# Animal HECT ubiquitin ligases: evolution and functional implications

**DOI:** 10.1186/1471-2148-10-56

**Published:** 2010-02-22

**Authors:** Ignacio Marín

**Affiliations:** 1 Instituto de Biomedicina de Valencia, Consejo Superior de Investigaciones Científicas (IBV-CSIC), Valencia, Spain

## Abstract

**Background:**

HECT ubiquitin ligases (HECT E3s) are key components of the eukaryotic ubiquitin-proteasome system and are involved in the genesis of several human diseases. In this study, I analyze the patterns of diversification of HECT E3s since animals emerged in order to provide the right framework to understand the functional data available for proteins of this family.

**Results:**

I show that the current classification of HECT E3s into three groups (NEDD4-like E3s, HERCs and single-HECT E3s) is fundamentally incorrect. First, the existence of a "Single-HECT E3s" group is not supported by phylogenetic analyses. Second, the HERC proteins must be divided into two subfamilies (Large HERCs, Small HERCs) that are evolutionarily very distant, their structural similarity being due to convergence and not to a common origin. Sequence and structural analyses show that animal HECT E3s can be naturally classified into 16 subfamilies. Almost all of them appeared either before animals originated or in early animal evolution. More recently, multiple gene losses have occurred independently in some lineages (nematodes, insects, urochordates), the same groups that have also lost genes of another type of E3s (RBR family). Interestingly, the emergence of some animal HECT E3s precedes the origin of key cellular systems that they regulate (TGF-β and EGF signal transduction pathways; p53 family of transcription factors) and it can be deduced that distantly related HECT proteins have been independently co-opted to perform similar roles. This may contribute to explain why distantly related HECT E3s are involved in the genesis of multiple types of cancer.

**Conclusions:**

The complex evolutionary history of HECT ubiquitin ligases in animals has been deciphered. The most appropriate model animals to study them and new theoretical and experimental lines of research are suggested by these results.

## Background

Ubiquitination is a critical process in all eukaryotic organisms. It is involved in multiple essential functions, from its best-known role in the regulation of protein levels to additional, precise roles in endocytosis, cellular signaling, DNA repair or the regulation of gene expression [1-3]. Ubiquitin ligases (E3s), a numerous and highly diverse group of enzymes able to transfer ubiquitin to target proteins, are fundamental components of the ubiquitination system [3]. E3 enzymes can be classified into a few groups, depending on whether they are single proteins or multiprotein complexes and on structural features of the proteins involved. One of the most characteristic types of E3s is the proteins of the HECT family. HECT E3s are enzymes that have a C-terminal HECT domain, involved in both accepting ubiquitin from a ubiquitin-conjugating protein and catalyzing its transfer to the protein to be ubiquitinated [4]. Exceptionally, one HECT enzyme, HERC5, has been shown to be involved in attaching the ubiquitin-like protein ISG15, instead of ubiquitin, to its substrates [5,6]. Because they are involved in the regulation of several basic cellular mechanisms (signal transduction pathways, protein trafficking, DNA damage, etc), HECT proteins have attracted a considerable attention and interest in them is growing since they have been found to be involved in several human diseases (reviewed in [4,7-9]). Of particular interest is that multiple HECT E3s have been associated to a broad variety of types of cancer [7,8,10].

HECT family proteins have been divided into three classes: 1) Those containing tryptophan-tryptophan (WW) domains, called NEDD4-like E3s; 2) Those containing RCC1-like domains, known as HERCs; and, 3) those lacking any of those domains (Single-HECT E3s) [4,7,8]. However, whether this simple classification makes sense from an evolutionary point of view has not been hitherto investigated. Currently, the evolutionary history of HECT ubiquitin ligases is unknown. Consequently, there are many basic questions that remain unanswered. For instance, we cannot establish when HECT E3s acquired their current roles or whether HECT E3s that perform similar functions are evolutionary closely related or not. This situation not only implies a general lack of understanding of this group of proteins but also generates practical problems, such as to make difficult to determine the model organisms that may be used to improve our understanding of the functions of the HECT E3s involved in human pathologies. In this work, I performed a comprehensive set of analyses to determine the relationships among HECT E3s, obtaining significant advances in our understanding of their evolution and also novel hints about how they acquired their current functions.

## Methods

A first step in this study was the generation of a comprehensive database of HECT proteins. To obtain that database, I performed TBLASTN and BLASTP searches, using multiple HECT domain sequences as queries, against the nr, htgs, gss, est and wgs databases of the National Center for Biotechnology Information http://www.ncbi.nlm.nih.gov/. After eliminating duplicates, sequences with identity ≥ 99% and partial sequences (size < 300 amino acids), a final database of 1081 protein sequences was obtained. From it, I selected 594 derived from metazoan genomes or from the choanoflagellate *Monosiga brevicollis*. This species is a good outgroup to establish which genes were already present when animals emerged, given that choanoflagellates are the closest extant relatives of animals [11]. The 594 sequences were then aligned using Clustal X 2.07 [12] and the alignment was manually edited with GeneDoc 2.7 [13] to correct for minor mistakes. For phylogenetic analyses, dendrograms were obtained using three different methods of tree reconstruction (neighbor-joining [NJ], maximum parsimony [MP] and maximum likelihood [ML]). The methods used to obtain these dendrograms were essentially the same described in [14]. The only two minor improvements on that paper were that the number of tied trees saved in the MP procedure was here 100, instead of 20, and that 200 bootstrap replicates (and not just 100) were performed in the ML analyses.

The searches performed to generate this database were not exhaustive. With the methods that I used, some members of the HECT family (e. g. those partially sequenced) could have passed undetected. Thus, in order to obtain a more precise picture of the patterns of appearance and loss of HECT E3s, I performed a second, more focused, set of searches to detect all the HECT proteins present in 14 model species with fully or almost fully sequenced genome. These species were the choanoflagellate *Monosiga brevicollis*, the placozoan *Trichoplax adhaerens*, the cnidarian *Nematostella vectensis*, six insects (two dipterans, *Drosophila melanogaster *and *Anopheles gambiae*, two hymenopterans, *Apis mellifera *and *Nasonia vitripennis*, the beetle *Tribolium castaneum *and the hemipteran *Acyrthosiphon pisum*), two nematodes such as *Caenorhabditis elegans *and *Brugia malayi*, the echinoderm *Strongylocentrotus purpuratus*, the urochordate *Ciona intestinalis*, and, finally, our own species, the vertebrate *Homo sapiens*. Moreover, I also searched for all HECT proteins and protein fragments present in a wide set of lophotrochozoan species (i. e. invertebrates such as molluscs, platyhelminthes, annelids, etc), for which the set of HECT E3s is still quite incomplete.

Within a protein family, subfamilies can be defined as sets of proteins present in multiple species that are both evolutionary closely related -- as indicated by sequence-based analyses -- and share structural features, such as the presence of subfamily-specific protein domains. In previous works, we successfully used a classification into subfamilies to analyze the long-term evolution of another family of E3s, named RBR [14-17]. Here, I used similar methods. In order to establish subfamilies within the HECT family, I combined the results of phylogenetic and structural analyses. Thus, on one hand, branches used to establish the proteins that belong to a subfamily must be significantly supported by bootstrap analyses. On the other hand, proteins in those branches must be structurally very similar or identical. Structural analyses were performed with InterProScan [18]. All the HECT proteins in humans, the placozoan *Trichoplax adhaerens*, the cnidarian *Nematostella vectensis *and the choanoflagellate *Monosiga brevicollis *were analyzed. The proteins of the three model animal species were identical unless otherwise indicated.

Finally, the functional data regarding the substrates of HECT E3s were obtained from the current literature (up to the end of September 2009) by searching the PubMed database http://www.ncbi.nlm.nih.gov/sites/entrez?db=PubMed, using the names and synonyms described in the NCBI Entrez Gene database http://www.ncbi.nlm.nih.gov/sites/entrez?db=gene for each mammalian HECT gene. TBLASTN analyses were used to determine whether the substrates of particular HECT E3s in mammals had orthologs in *Monosiga *or *Trichoplax *species. For these analyses, human proteins were used as query sequences.

## Results

### A natural classification of the HECT family

**Figure 1:**
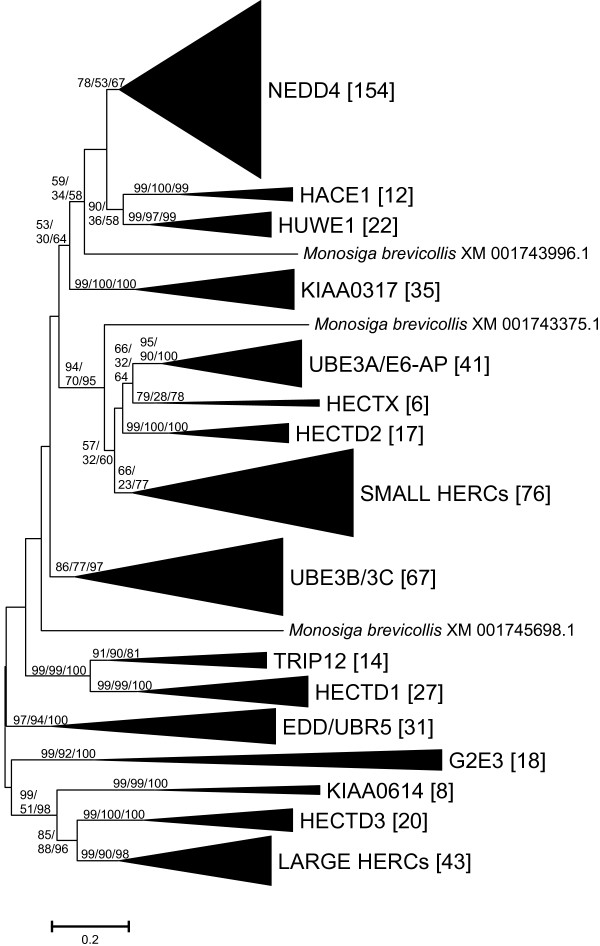
**HECT E3 subfamilies**. The results of all the phylogenetic analyses were highly congruent, so they are depicted in a single tree. Number above branches refer to bootstrap support (NJ/MP/ML; in percentages; only consistently high bootstrap values are indicated). The number of sequences included in each subfamily is also indicated (in brackets). All the families except the one that I have named HECTX contain at least one human protein. Thus, I used the names of the human proteins to call the corresponding subfamilies.

**Figure 2:**
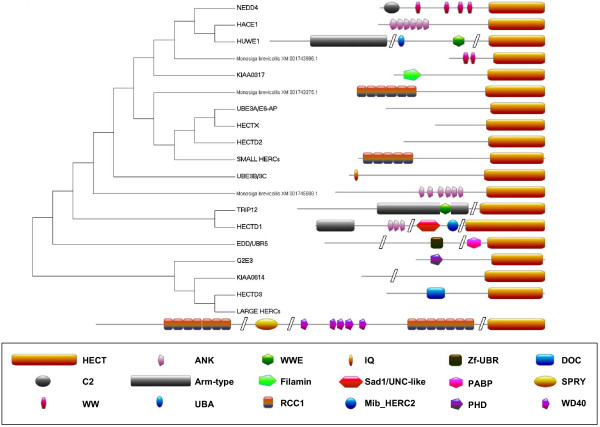
**Structures of typical members of the subfamilies**. To obtain this figure, all the HECT proteins present in humans, the placozoan *Trichoplax adhaerens*, the cnidarian *Nematostella vectensis *and the choanoflagellate *Monosiga brevicollis *were structurally analyzed using InterProScan [18]. The structures from orthologous proteins in the three animals were generally identical, and therefore all but four of the proteins shown here are from humans. The four exceptions derive from *Monosiga brevicollis*. Three of them are detailed in the figure. The fourth belongs to the HECTX subfamily, which is not present in vertebrates. Minimal variations were detected in some NEDD4 subfamily proteins (e. g. lack of the C2 domain; lack of one of the WW domains). Also, this figure shows the structure of just one of the Large HERC proteins, HERC1, but does not depict the structure of HERC2 (described before by e. g. [31]).

The three methods of phylogenetic reconstruction yielded very similar results and it was possible to subdivide the HECT family in groups highly supported by bootstrap analyses. Figure 1 shows a summary of the trees obtained (see details in Additional files 1 and 2). As shown in that figure, 99.5% (591/594) of the proteins were ascribed to 16 "natural" groups, which were fully confirmed using structural data: Each group contained proteins with identical or nearly identical structures (Figure 2). Variations were only detected in a few NEDD4-like proteins (e. g. lack of the C2 domain; lack of one of the WW domains). Following the conventions used to classify the RBR family of ubiquitin ligases [14-17], I have called these groups as subfamilies. Therefore, each subfamily is an evolutionary independent unit and the members of a particular subfamily have both similar sequences and a characteristic protein structure. As detailed in the Material and methods section, given that the searches performed were not exhaustive, there was the possibility of additional subfamilies being missed. However, although searches in multiple model species (see details in Material and methods) indeed unearthed some additional sequences, no new subfamilies were detected. Table 1 summarizes the details of the genes included in each subfamily

**Table 1:** Number of genes of the different subfamilies present in model organism or deduced for key ancestors of current groups from data of multiple species.

Subfamily	*Monosiga brevicollis*	Ancestor of all animals	*Trichoplax adhaerens*	Cnidarian ancestor	Ancestor of protostomes, deuterostomes	*Caenorhabditis elegans*	*Homo sapiens*
NEDD4	4	4	4	4	4	3	9 (*NEDD4, NEDD4L, WWP1, WWP2, ITCH, SMURF1, SMURF2, NEDL2, NEDL1*)
HACE1	0	0	0	0	0	0	1
HUWE1	1	1	1	1	1	1	1
KIAA0317	0	1	2	1	1	0	1
UBE3A/E6-AP	0	1	1	1	1	0	1
HECTX	1	1	1	1	1	0	0
HECTD2	0	1	1	1	1	0	1
Small HERCs	1	1	1	1	1	1	4 (*HERC3, HERC4, HERC5, HERC6*)
UBE3B/3C	1	2	2	2	2	2	2 (*UBE3B, UBE3C*)
TRIP12	1	1	1	1	1	0	1
HECTD1	1	1	1	1	1	1	1
EDD/UBR5	1	1	1	1	1	1	1
G2E3	0	0	0	0	0	0	1
KIAA0614	1	1	0	1	1	0	1
HECTD3	0	1	1	2	1	0	1
Large HERCs	1	2	2	2	2	0	2 (*HERC1, HERC2*)
Not assigned	4*	0	0	0	0	0	0
							
**TOTAL**	**17**	**19**	**19**	**20**	**19**	**9**	**28**
							
**No. subfamilies**	**10**	**14**	**13**	**14**	**14**	**6**	**15**

When several genes of the same family are present in humans, the names are indicated. Otherwise, the single human gene has the same name as the corresponding subfamily. *Monosiga *contains four genes that cannot be assigned to any subfamily (asterisk). Three of them are detailed in Figure 2. For the fourth (Accession number XM_001748120) only a partial HECT domain was obtained, precluding their inclusion in the phylogenetic analyses.

The classification obtained totally contradicts the currently accepted division of HECT E3s into three groups. There are two main incongruences. First, HERC E3s, one of the accepted classes of HECT ubiquitin ligases, are in fact divided into two subfamilies that are distantly related (Small HERCs, Large HERCs). This result agrees well with the fact that the RCC1-like domains in Small and Large HERCs are very different [19]. Actually, the distant relationship observed when analyzing their HECT domains indicates that the RCC1-like domains of these proteins have been acquired twice independently. Second, the postulated "Single HECT" group is meaningless from an evolutionary point of view. The proteins of that group can be naturally divided into 13 subfamilies, which are moreover interspersed in the trees with HERCs and NEDD4-like E3s. Therefore, the only group in the current classification of HECT E3s that is evolutionary supported is that of NEDD4-like E3s (NEDD4 subfamily; Figure 1). According to the results obtained, the NEDD4 subfamily is a monophyletic group which contained four members when animals emerged. Most animal groups have retained those 4 ancestral genes (that I have called *NEDD4a *- *NEDD4d*) while a substantial increase occurred in vertebrates, which have 9 NEDD4 subfamily genes (see details in Table 1). This increase was most likely associated to the genomic duplications that occurred in the vertebrate lineage. A caveat for the conclusion that the NEDD4 subfamily is monophyletic is that, given that the trees summarized in Figure 1 are unrooted, it is a formal possibility that the root was to be found within the NEDD4 group. This option seems however unlikely, given the high similarity in sequence and structure of all the proteins of this subfamily. A final important conclusion is that some subfamilies are significantly related. High bootstrap support for some inner branches of the trees, which include several subfamilies (e. g. HACE1-HUWE1; UBE3A-HECTX-HECTD2-Small HERCs; TRIP12-HECTD1; KIAA0614-HECTD3-Large HERCs), can be observed in Figure 1.

These conclusions imply a novel paradigm of how distinct HECT E3s relate to each other and therefore have significant functional implications, as a simple example may indicate: on one hand, these results mean that caution is required to compare functional data for Small HERC and Large HERC subfamily proteins, given that their similarity is due to convergence. On the other hand, they also unsuspectedly suggest that data from HECTD3 subfamily proteins may provide clues of how the Large HERC subfamily proteins function, given their close evolutionary relationships (Figure 2). In fact, HECTD3 and the Large HERC protein HERC1 are the only HECT E3s that contain DOC domains [20], indicating an ancient functional similarity.

### Patterns of emergence and loss of HECT ubiquitin ligases in animals

**Figure 3:**
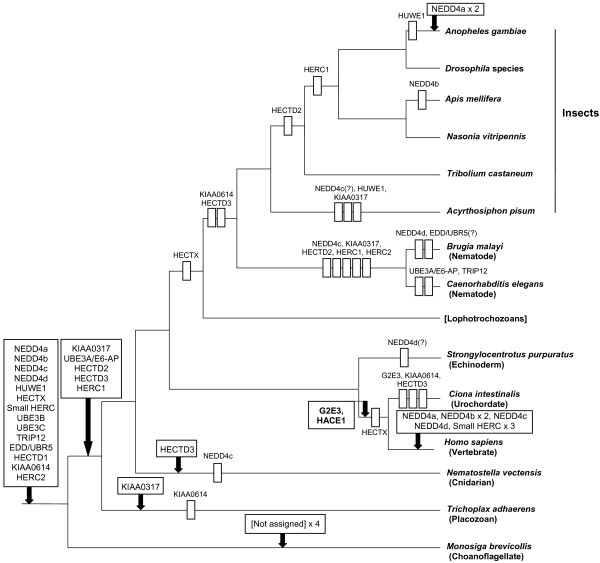
**Most parsimonious reconstruction of the patterns of emergence and loss of HECT E3s in animals**. Arrows indicate the emergence of new genes (indicated in the boxes) and rectangles, a gene loss. Question marks indicate cases in which it is unclear that a gene is present or not, due to partial data. The names *NEDD4a-d *refer to the four ancestral genes of the NEDD4 subfamily that still exist in many animals (Table 1) and that emerged before the metazoa/choanoflagellata split. In humans, the genes *NEDD4 *and *NEDD4L *derive from a duplication of *NEDD4a*, *WWP1*, *WWP2 *and *ITCH *derive from *NEDD4b*, *Smurf1 *and *Smurf2 *from *NEDD4c *and *NEDL1 *and *NEDL2 *from *NEDD4d*. The partial data available for lophotrochozoans (in brackets) allows concluding that all genes are present in at least one species of this group of organisms, and therefore they were all present in their common ancestor. However, losses in particular lineages may have occurred.

Considering all the data obtained together, it was possible to formulate the most parsimonious hypothesis for the diversification of HECT E3s in animals, which is summarized in Figure 3. This hypothesis is based on minimizing the number of evolutionary events that must have occurred to explain the current patterns of presence/absence of HECT-encoding genes. Three main conclusions can be drawn from Figure 3. First, it turns out that almost all HECT subfamilies, and in general the great majority of HECT-encoding genes currently detected in all species, originated either before the choanoflagellata/metazoa split or just after that split. In fact, the number of subfamilies that existed before the origin of metazoans may be underestimated by these analyses, given that it is possible that the lineage that gave rise to the single choanoflagellate analyzed lost some HECT genes. In any case, we can safely conclude that the first animals had a diversity of HECT E3s (19 genes from 14 subfamilies) that was quite similar to that found now in living animals. This is made especially clear when the number and variety of HECT E3s in a few key organisms is detailed (Table 1). The second conclusion is that there has been a reduction in the number of HECT E3s in particular metazoan lineages, such as nematodes, insects and urochordates. Their sets of HECT E3s are in some cases (see e. g. *Caenorhabditis elegans *data) much simpler than the one that can be deduced for the ancestor of all animals (Table 1, Figure 3). Finally, the third conclusion is that the number of HECT proteins largely increased in the vertebrate lineage. The known genomic duplications in that lineage may explain this pattern. However, that increase is concentrated on just two subfamilies (NEDD4 and Small HERCs; Figure 3), indicating that many other duplicates have been lost. In the NEDD4 subfamily, finding only 2-3 genes of each orthology group (NEDD4a-NEDD4d), instead of 4, implies that some losses have also occurred.

### Correlations between diversification and function of HECT proteins

**Figure 4:**
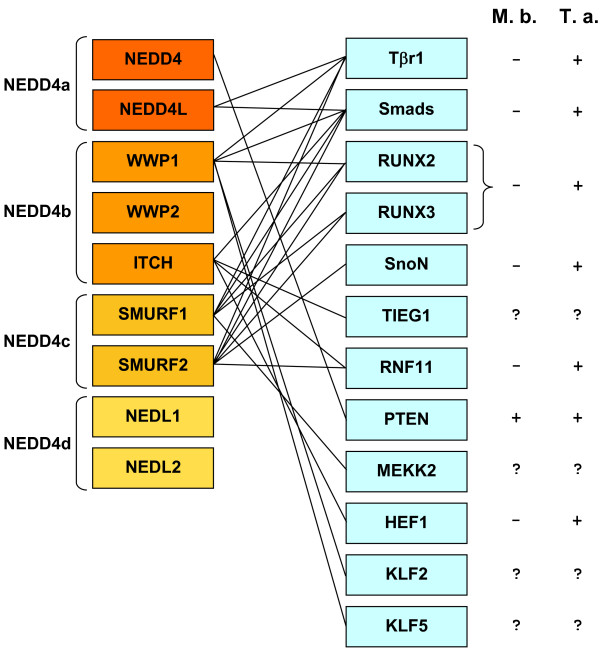
**Known substrates of HECT E3s that are related to the TGF-β signaling pathway**. Lines connect the enzymes and their corresponding substrates. The presence (+) or absence (-) of genes encoding those substrates in the choanoflagellate *Monosiga brevicollis *(M. b.) and the placozoan *Trichoplax adhaerens *(T. a.) is also indicated. *NEDD4a *- *NEDD4d *refer to the four genes already present in early animals. A question mark indicates that one or more related genes are detected, but it is unclear whether they are true orthologs of the corresponding human genes. It is clear from this data that NEDD4 genes of different origin ubiquitinate now the same substrates.

Elucidating the evolution of the HECT family of E3s may allow for a more precise understanding of how the current functions of these proteins emerged. Although the functional information for animal HECT E3s is largely restricted to mammalian proteins, it is still possible to obtain some ideas of how several functions originated by comparing the patterns of presence/absence of genes encoding HECT proteins and genes encoding their substrates. More precisely, the question that can be tackled is when HECT E3s started to ubiquitinate the substrates that we observe today. Of particular interest are three basic systems present in all animals that are known to be regulated by HECT E3s. The first of them is the transforming growth factor β (TGF-β) signaling pathway, one of the main signal transduction systems in animals, involved in multiple cellular and developmental processes [21]. The TGF-β pathway is precisely regulated by ubiquitination [22,23]. Particularly, it is well established that several members of the NEDD4 subfamily of HECT E3s control, directly or indirectly, TGF-β signaling [4,22,23]. Figure 4 summarizes the known substrates of NEDD4 E3s that are known to be related to the TGF-β signaling pathway. They include from one of the units of the TGF-β receptor (Tβr1) and multiple critical mediators of the pathway (e. g. several Smads) to downstream targets of this signaling system, such as transcription factors that are regulated by TGF-β signaling (e. g. RUNX2, RUNX3) or the protein phosphatase PTEN, a well-known tumor suppressor (see further details in the legend of Figure 4). Figure 4 also includes whether these TGF-β-related proteins are detected in choanoflagellates (*Monosiga*) or in placozoans (*Trichoplax*). This allows comparing the origin of the NEDD4 subfamily genes with the origin of their substrates. The picture that emerges is very complex. On one hand, and as it could be expected, some proteins that appeared recently, from vertebrate-specific duplications, have often the same substrates (e. g. the proteins encoded by *Smurf1 *and *Smurf2*, originated from a duplication of the ancestral NEDD4c gene; Figures 3, 4). However, on the other hand, and surprisingly, proteins encoded by genes that emerged from duplications that occurred before animals originated also often share substrates. For example, the proteins encoded by *NEDD4L*, *WWP1 *and the *Smurf *genes, which respectively derive from the *NEDD4a*, *NEDD4b *and *NEDD4c *ancestral genes, have multiple common substrates (Figure 4). This similarity is even more surprising, considering that the four ancestral NEDD4 genes (*NEDD4a *- *NEDD4d*) originated before the emergence of the TGFβ signaling pathway, which is absent in choanoflagellates ([11]; see also Figure 4). Therefore, it can be deduced that several HECT E3s have - independently and long after they arose -- acquired the ability to modify the same substrates.

**Figure 5:**
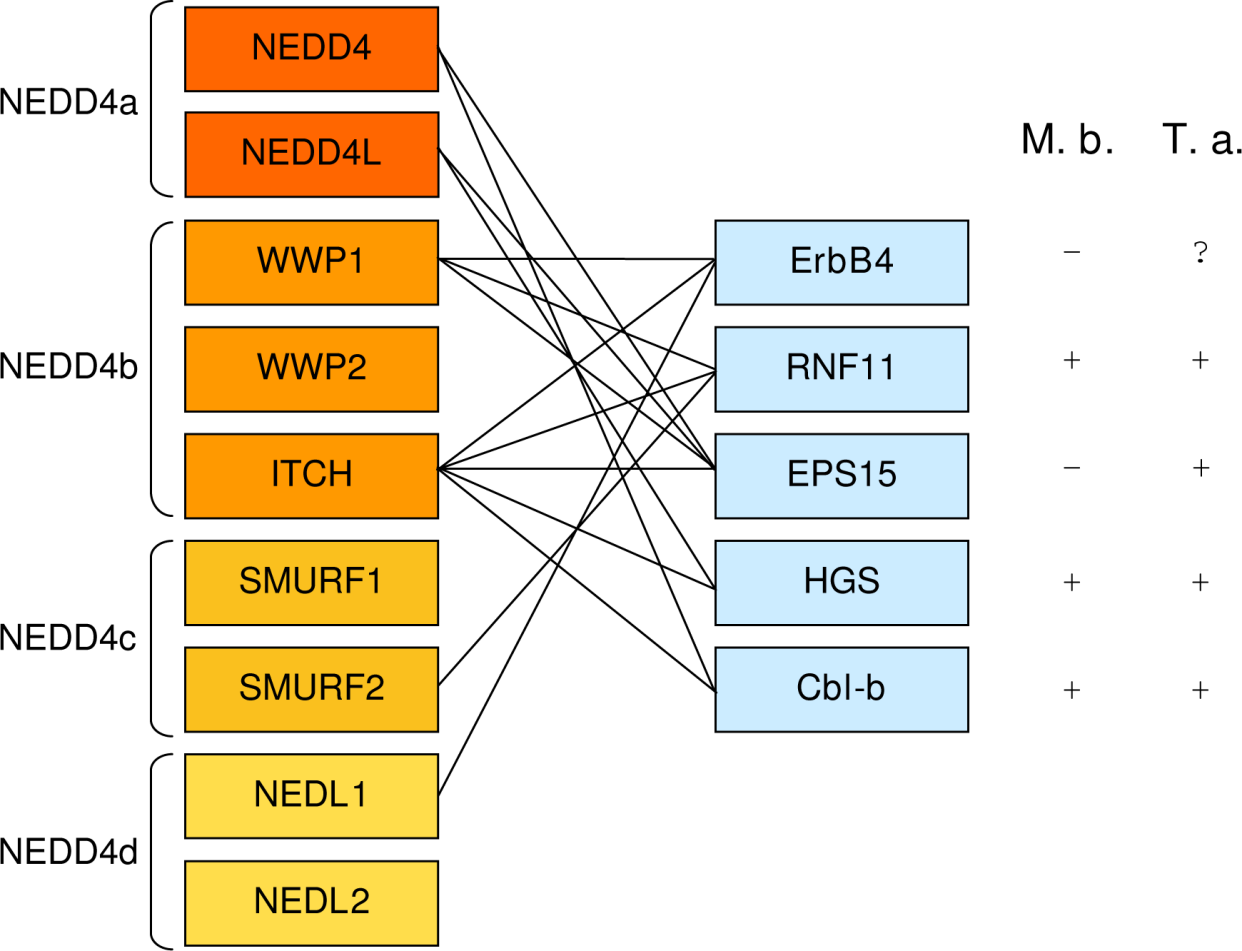
**Substrates of HECT E3s that are related to EGFR metabolism**. Conventions as in Figure 4.

**Figure 6:**
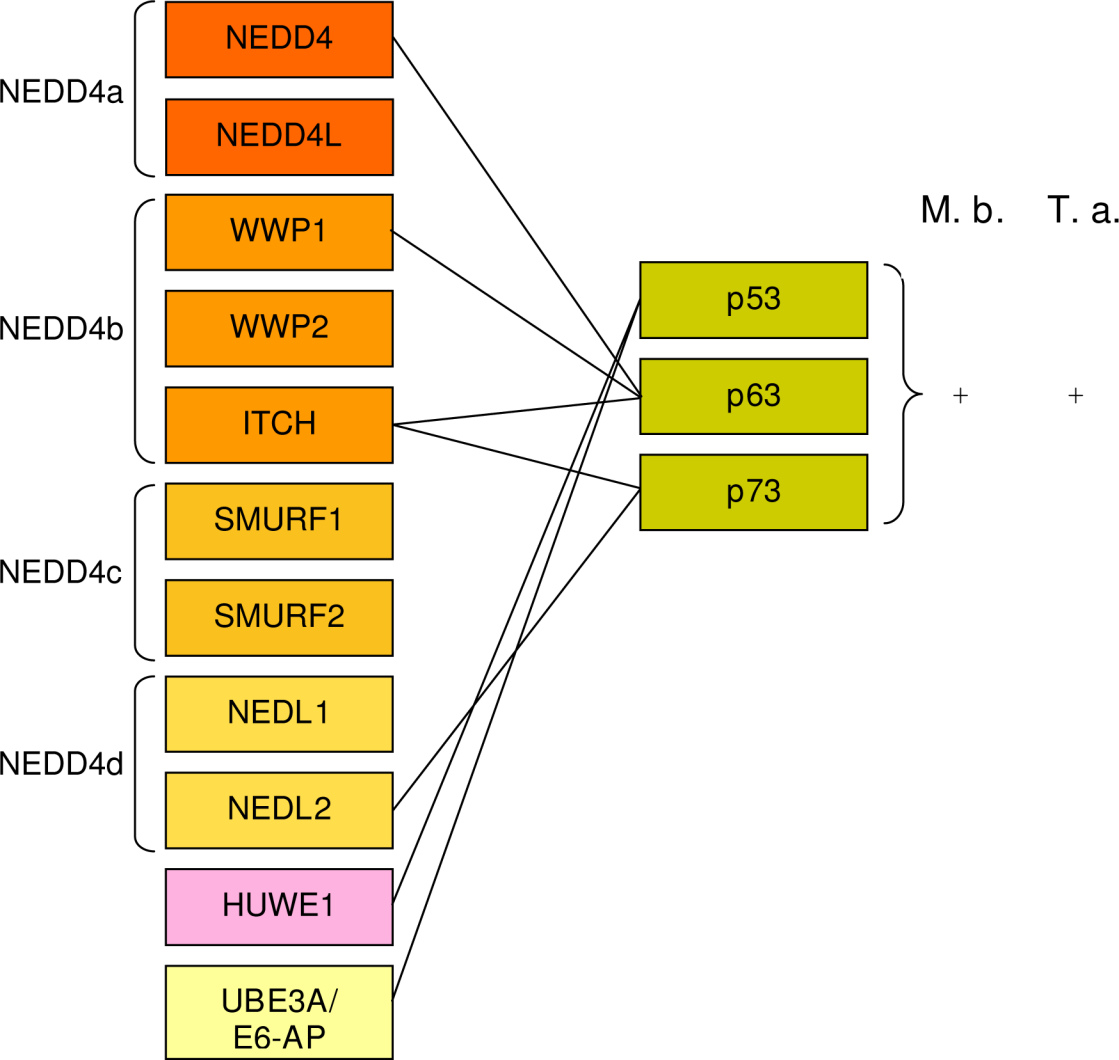
**Details of the HECT ubiquitin ligases that are known to ubiquitinate p53 family proteins**. See Figure 4 and main text for the details.

Interestingly, a similar situation is found when several proteins involved in the endocytosis of epidermal growth factor receptors (EGFRs) are considered. Both one of the proteins of the EGFR family (ErbB4) and several mediators and regulators of the endocytosis of EGFRs are ubiquitinated by NEDD4 subfamily proteins (Figure 5; [10]). It turns out that all the proteins related to EGFR metabolism are ubiquitinated by 2 - 4 NEDD4 subfamily members that derive from at least two different ancestral genes (Figure 5). Finally, the same complex pattern is found for members of the p53 family of transcription factors, which are also ubiquitinated by HECT E3s ([4,10]; Figure 6). In this case, not only NEDD4 subfamily proteins but also HUWE1 are known to ubiquitinate p53 or p53-related proteins. Moreover, UBE3A/E6-AP (member a third subfamily) has been found also to ubiquitinate p53, albeit only when complexed with the papillomavirus protein E6 [24]. It is obvious that all these proteins originated well before the diversification of the p53 family: although a p53-related protein is already present in choanoflagellates, the three members that we found in vertebrates (p53, p63 and p73) emerged by duplication of the single gene present before the cephalochordate/vertebrate split [25]. Again, independent cooptions of the same substrates by very different HECT E3s have occurred.

## Discussion and Conclusions

This study is focused on the characterization of the origin and evolutionary history of HECT ubiquitin ligases. The combination of sequence and structural analyses (Figures 1 and 2) allows establishing a natural classification of these proteins. This classification turns to be totally different from the one hitherto assumed. The division in sixteen subfamilies - groups of proteins that have very similar sequences and structures -- is much more precise than the classification into three groups proposed before [4,7,8]. Moreover, I have found that at least two of the three groups defined in previous studies are not monophyletic, and therefore, not being real evolutionary units, should not be used for classification purposes. Therefore, this work provides a new evolutionary paradigm for the HECT family.

Twelve of the sixteen subfamilies generally group single orthologous genes in all animal species in which they are present, while the other four (NEDD4, UBE3B/3C, Large HERCs and Small HERCs) include several (up to nine genes for the NEDD4 subfamily in vertebrates). However, the results obtained also hint to broader classification patterns that put together members of different subfamilies (as indicated above: HACE1-HUWE1; UBE3A-HECTX-HECTD2-Small HERCs; TRIP12-HECTD1; KIAA0614-HECTD3-Large HERCs). Many of the genes and proteins of these subfamilies have been so far barely explored. Thus, to known of these cryptic relationships may be useful to design experiments based on what is understood for members of closely related subfamilies.

The precise analysis of the patterns of presence/absence in multiple model organisms allowed establishing the most parsimonious hypothesis for the evolution of HECT genes in animals (Figure 3). Notably, most HECT subfamilies arose before the emergence of animals or very early in metazoan evolution. The number of novel subfamilies emerged later is very low, just two of them (G2E3 and HACE1) appeared after the chordate/echinoderm split (Figure 3). I conclude that, since the origin of animals, HECT genes have generally been either maintained or, in some lineages such as insects, nematodes and urochordates, lost. Only vertebrates have a number of HECT genes much higher than the one that can be deduced for early animals, due to specific duplications of both the NEDD4 and the small HERC subfamily genes (Table 1; Figure 3). It is striking that all these patterns of diversification and streamlining are virtually identical, lineage by lineage, to the ones that I recently described for RBR ubiquitin ligases [14]. This suggests the presence of underlying selective forces acting on the evolution of the animal ubiquitination system as a whole, sometimes leading to its simplification by the progressive loss of E3s. The deep reasons that explain the parallelism observed for RBR and HECT ubiquitin ligases are a mystery. To discover what controls the patterns of duplication, conservation and loss of E3 proteins is another promising line of research.

The comparison of the emergence of the HECT genes and the genes that encode known substrates of HECT proteins (Figures 4, 5 and 6) follows a complex pattern that cannot be simply explained by the typical "textbook" processes that we know often follow after gene duplications, such as neofunctionalization leading to the acquisition of new functions by one of the duplicates or subfunctionalization that divides the functions of the original gene between its duplicates. This is especially obvious for the members of the TGF-β signaling pathway regulated by NEDD4 subfamily HECTs (Figure 4). I have found that there were four NEDD4 genes before animals emerged (Figure 3). It is also well established that the TGF-β system is not present in choanoflagellates ([11] and analyses presented above). Therefore, the simplest expectation would be that, in early animal history, a single NEDD4 protein was co-opted to regulate the novel signaling system. However, the current mammalian data shows that multiple, distantly related NEDD4 subfamily proteins are involved in the regulation of many (and often the same) proteins of the TGFβ pathway. This must be interpreted as evidence for multiple independent cooptions of HECT proteins to perform similar roles in the TGF-β pathway. The caveat that many of these results have been obtained *in vitro *or by overexpressing the E3s in cell culture assays, and often through directed searches devised to test multiple related NEDD4 proteins (e. g. [26]), must be acknowledged. It is therefore possible that not all the interactions described so far actually occur in whole organisms, However, if the pattern shown in Figure 4 is basically correct (and the patterns shown in Figures 5 and 6 for other systems indeed have similar features), then it is a strong indication that enzymes that are part of complex families, with many members, may act on functionally related substrates in ways that do not follow any simple pattern and therefore may be largely unpredictable. This type of results indicates that there may be significant shortcomings in our current models of how duplicated genes differentiate, evolve new functions and are preserved in the genomes (see discussion in [27]). Often, simplistic expectations are not fulfilled and what is really happening can only be understood by the combination of detailed phylogenetic analyses and functional data, as it has been shown here and also, recently, in other related studies (e. g. [28-30]).

At present, data for substrates of HECT proteins have largely restricted to members of the NEDD4 family. Out of 131 substrates that I have found in the literature, 92 were described for NEDD4 proteins (Additional file 3). Therefore, it is still impossible to have a well-defined picture of all the different roles of the HECT E3s as a whole. However, the available results clearly point to a general involvement in the control of many key pathways that impinge on the regulation of gene expression [4]. This is obvious from the data that I have shown in Figures 4, 5 and 6, and it is reinforced by the large set of additional results showing that HECT E3s also regulate proteins that are either part of other signal transduction pathways (e. g. Notch, TrkA, Insulin-like growth factor, interleukin receptors, etc.) or directly involved in gene expression and its regulation (RNA pol II, histones, TopBP1, c-Jun, etc.). These results may contribute to explain one of the main features associated to this family, the fact that many of its members have been found in different ways to be associated to multiple types of cancer [7,8,10]. Results shown here demonstrate that these members are not necessarily closely related, but often belong to different subfamilies. Further experimental results may contribute to clarify whether there is some subfamily specificity, in which members of different subfamilies have clearly distinct roles and primarily affect different cell types, or whether the roles of distant members of the HECT family may be indeed substantially overlapping, as the data currently available suggest. In this direction of future research, the discovery of some model species in which the number of HECT E3s has been largely reduced (e. g. *Caenorhabditis elegans*, in which there are only 9 HECT genes; Table 1) may be especially interesting, given that it may help to sort out more easily the roles of particular HECT ubiquitin ligases.

## Authors' contributions

Single-authored paper.

## Supplementary Material

Additional file 1 **Summary of HECT sequences**. Microsoft Word (.doc) file with the 594 animal sequences, aligned, in FASTA format. Click here for file

Additional file 2 **Phylogenetic reconstruction of HECT protein relationships**. MEGA 4 (.mts) file corresponding to the NJ tree on which is based Figure 1. Click here for file

Additional file 3 **Substrates of the HECT ubiquitin ligases**. List of known substrates of HECT ubiquitin ligases and references that describe these substrates. Microsoft Excel (.xls) file. Click here for file
