# Supplementary material for: Animal HECT ubiquitin ligases: evolution and functional implications
Source: BMC Evol Biol. 2010 Feb 22;10:56. doi: 10.1186/1471-2148-10-56 (PMC2837046; doi:10.1186/1471-2148-10-56)
Supplement: Additional file 1 — Summary of HECT sequences. Microsoft Word (.doc) file with the 594 animal sequences, aligned, in FASTA format. [file 1471-2148-10-56-S1.DOC]

>Pan_troglodytes_AACZ02038620.1 .

INVSRQTSFEDSFQQIMALK----------------PYDLR-RCLYVIFR-----GEEGL

---DYGGLAREWLFLLSHEVLNP-------------------------------------

------------------------------------------------------------

------------------------------------------------------------

------------------------------------------------------------

------------------------------------------------------------

--------------------MYCLFEYVGKNN----------------------------

------------YSLQINPAS---------TINPDHLSYFCFIG--RFIAMALFHG----

-----KLIDTGFSL-PFYKHMLSKK-----------------------------------

----------------------------------------LTIKDLE---SIDTEFY---

NSLIWIRDNNIE------------------------------------------ECGLEL

YFSVDMES-----------------------------WGKVTSHDLKLGV----SNILVT

EENK--DEYIGLMTEWHFSRG-----VQEQTKAFLDG---------------FNGVV---

--PHQWL-QYFNEKELEVTLCGTQQ--------------------------VDLADWQRN

TVYQH----------------YTRNSKQIIRFWQFVK-ETDNEV----RMLLLQFVTGTC

RLSLGGFAELMGSN----GPQKFCIEKVDK---------------------------DTW

LP-RSHTCFN-----LLDLPP-YKSYEQ--LKEKLLFAIEET-EG-

>Homo_sapiens_AC234261.2 .

INVSRQTLFEDSFQQIMALK----------------PYDLR-RCLYVIFR-----GEEGL

---DYGGLAREWLFLLSHEVLNP-------------------------------------

------------------------------------------------------------

------------------------------------------------------------

------------------------------------------------------------

------------------------------------------------------------

--------------------M-CLFEYVGKNN----------------------------

------------YSLQINPAS---------TINPDHLSYYCFIG--RFIAMALFHG----

-----KLIDTGFSL-PFYKHVLSKK-----------------------------------

----------------------------------------LTIKDLE---SIDTEFY---

NSLIWIRDNNIE------------------------------------------ECGLEL

YFSVDMES-----------------------------WGKVTSHDLKLGV----SNILVT

EENK--DEYIGLMTEWHFSRG-----VQEQTKAFLDG---------------FNGVV---

--PHQWL-QYFNEKELEVTLCGTQQ--------------------------VDLADWQRN

TVYQH----------------YTRNSKQIIRFWQFVK-ETDNEV----RMLLLQFVTGTC

RLSLGGFAELMGSN----GPQKFCIEKVGK---------------------------DTW

LP-RSHTCFN-----LLDLPP-YKSYEQ--LKEKLLFAIEET-EG-

>Gorilla_gorilla_CABD01085931.1. .

---------------IMALK----------------PYDLR-RXL-VIFR-----GEEGL

---DYGGLAREWLFLLSHEVLNP-------------------------------------

------------------------------------------------------------

------------------------------------------------------------

------------------------------------------------------------

------------------------------------------------------------

--------------------MYCLFEYVGKNN----------------------------

------------YCLQINPAS---------TINPDHLSYFCFIG--RFIAMALFHG----

-----KLIDTGFSL-PFYKHMLSKK-----------------------------------

----------------------------------------LTIKDLE---SIDTEFY---

NSLIWIRDNNIE------------------------------------------ECGLEL

YFSVDMES-----------------------------WGKVTSHDLKLGV----SNILVT

EENK--DEYIGLMTEWHFSRG-----VQEQTKAFLDG---------------FNGVV---

--PHQWL-QYFNEKELEVTLCGMQE--------------------------VDLADWQRN

TVYQH----------------YTRNSKQIIRFWQFVK-ETDNEV----RMLLLQFVTGTC

RLSLGGFAELMGSN----GPQKFCIEKAGK---------------------------DTW

LP-RSHTCFN-----LLDLPP-YKSYEQ--LKEKLLFAIEET-EG-

>Pongo_abelii_ABGA01207039.1. .

INVSRQTLFEDSFQQIMALK----------------PYDLR-RCLYVIFR-----GEEGL

---DYGGLAREWLFLLSHEVLNT-------------------------------------

------------------------------------------------------------

------------------------------------------------------------

------------------------------------------------------------

------------------------------------------------------------

--------------------MYCLFEYAGKNN----------------------------

------------YCLQINPAS---------TINPDHLSYFCFIG--RFIATALFHG----

-----KFIDTDFSL-PFYKRMLSEK-----------------------------------

----------------------------------------LTIKDLE---SIDTEFY---

NSLIWIRDNNIE------------------------------------------ECGLEP

YFSVDMES-----------------------------WGKVTLHDPKLGV----SNILVT

EENK--DEYIGLMTEWHFSRG-----VQEQTKTFLDG---------------FNGVV---

--PRQRL-QYFDEKDLEVMLG-TQE--------------------------ADLADWQRN

TLYQH----------------YTRNSKQIIRFRQFVK-ETDNEV----RMLLSQFVTGTC

RLSLGGFAELMGSN----GTQKFCIEKVGK---------------------------DTW

LP-RSHTCFN-----LLDLPP-YKIYKQ--LKEKLLFAIEET-EGF

>Choloepus_hoffmanni_ABVD01008253.1 .

ISVSWQTVLEDSFQQIMALK----------------PYDLR-RRLYVILR-----G-EGL

---DYGGLAREF-FLLSHEVLNP-------------------------------------

------------------------------------------------------------

------------------------------------------------------------

------------------------------------------------------------

------------------------------------------------------------

--------------------MYCLFEYAGKNS----------------------------

------------YSLQINPAS---------TINPDHLSYFCFIG--CFITVALFCR----

-----KFIDTGFSL-PFYKCLLSKK-----------------------------------

----------------------------------------FTVKELE---SIDTEFY---

NSLIWIRDNNIE-------------------------------------------CGLEM

YFSVDMEI-----------------------------LGKVTSRNLKLGG----SNILVT

EENK--DEYIGLMTE-HFSQG-----VQEQTKAFLDG---------------FNEVV---

--LLQWL-QYFDEKELEVMLCGMQE--------------------------TNLADWQRN

TVYWH----------------YTRNSKQIIWFWQFVK---DNEV----RM-LLQFVTGTC

CLPLGGFAELMGSN----GSQKFCIEKVGK---------------------------DTW

LP-RSHMCFN-----CLDLPP-YKSYEQ--LKEKLLFAIERQ-RDL

>Canis_familiaris_XM_856135.1 .

INVSRQTLFEDSFQQIMALK----------------PYDLR-RRLYVIFR-----GEEGL

---DYGGLAREWFFLLSHEVLNP-------------------------------------

------------------------------------------------------------

------------------------------------------------------------

------------------------------------------------------------

------------------------------------------------------------

--------------------MYCLFEYAGKNN----------------------------

------------YCLQINPAS---------TINPDHLSYFCFIG--RFIAMALFHG----

-----KFIDTGFSL-PFYKRMLSKK-----------------------------------

----------------------------------------LTIKDLE---SIDTEFY---

NSLIWIRDNNIE------------------------------------------ECGLEM

YFSVDMEI-----------------------------LGKVTSHDLKLGG----SNILVT

EENK--DEYIGLMTEWRFSRG-----VQEQTKAFLDG---------------FNEVV---

--PLQWL-QYFDEKELEVMLCGMQE--------------------------VDLADWQRN

TVYRH----------------YTRNSKQIIWFWQFVK-ETDNEV----RMRLLQFVTGTC

RLPLGGFAELMGSN----GPQKFCIEKVGK---------------------------DTW

LP-RSHTCFN-----RLDLPP-YKSYEQ--LKEKLLFAIEET-EGF

>Pan_troglodytes_XM_001135743.1 .

INVSRQTLFEDSFQQIMALK----------------PYDLR-RRLYVIFR-----GEEGL

---DYGGLAREWFFLLSHEVLNP-------------------------------------

------------------------------------------------------------

------------------------------------------------------------

------------------------------------------------------------

------------------------------------------------------------

--------------------MYCLFEYAGKNN----------------------------

------------YCLQINPAS---------TINPDHLSYFCFIG--RFIAMALFHG----

-----KFIDTGFSL-PFYKRMLSKK-----------------------------------

----------------------------------------LTIKDLE---SIDTEFY---

NSLIWIRDNNIE------------------------------------------ECGLEM

YFSVDMEI-----------------------------LGKVTSHDLKLGG----SNILVT

EENK--DEYIGLMTEWRFSRG-----VQEQTKAFLDG---------------FNEVV---

--PLQWL-QYFDEKELEVMLCGMQE--------------------------VDLADWQRN

TVYRH----------------YTRNSKQIIWFWQFVK-ETDNEV----RMRLLQFVTGTC

RLPLGGFAELMGSN----GPQKFCIEKVGK---------------------------DTW

LP-RSHTCFN-----RLDLPP-YKSYEQ--LKEKLLFAIEET-EGF

>Equus_caballus_XM_001488288.2 .

INVSRQTLFEDSFQQIMALK----------------PYDLR-RRLYVIFR-----GEEGL

---DYGGLAREWFFLLSHEVLNP-------------------------------------

------------------------------------------------------------

------------------------------------------------------------

------------------------------------------------------------

------------------------------------------------------------

--------------------MYCLFEYAGKNN----------------------------

------------YCLQINPAS---------TINPDHLSYFCFIG--RFIAMALFHG----

-----KFIDTGFSL-PFYKRMLNKK-----------------------------------

----------------------------------------LTIKDLE---SIDTEFY---

NSLIWIRDNNIE------------------------------------------ECGLEM

YFSVDMEI-----------------------------LGKVTSHDLKSGG----SNILVT

EDNK--DEYIGLMTEWRFSRG-----VQEQTKAFLDG---------------FNEVV---

--PLQWL-QYFDEKELEVMLCGMQE--------------------------VDLADWQRN

AVYRH----------------YTRNSKQIIWFWQFVK-ETDNEV----RMRLLQFVTGTC

RLPLGGFASCLGSN----GPQKFCIEKVGK---------------------------DTW

LP-RSHTCFN-----RLDLPP-YKSYEQ--LKEKLLFAIEET-EGF

>Ornithorhynchus_anatinus_XM_001506900.1 .

INVSRQTLFEDSFQQIMALK----------------PYDLR-RRLYVIFR-----GEEGL

---DYGGLAREWFFLLSHEVLNP-------------------------------------

------------------------------------------------------------

------------------------------------------------------------

------------------------------------------------------------

------------------------------------------------------------

--------------------MYCLFEYAGKSN----------------------------

------------YCLQINPAS---------TINPDHLSYFCFIG--RFIAMALFHG----

-----KFIDTGFSL-PFYKRMLSKK-----------------------------------

----------------------------------------LTIKDLE---SIDTEFY---

NSLIWIRDNNIE------------------------------------------ECGLEM

YFSVDMEI-----------------------------LGKVTSHDLKLGG----SNILVT

EENK--EEYIGLMAEWRFSRG-----VQEQTKAFLDG---------------FNEVV---

--PLQWL-QYFDEKELEVMLCGMQE--------------------------VDLADWQRN

TVYRH----------------YTRNSKQIIWFWQFVK-ETDNEV----RMRLMQFVTGTC

RLPLGGFAELMGSN----GPQKFCIEKVGK---------------------------ETW

LP-RSHTCFN-----RLDLPP-YKSYEQ--LKEKLLFAIEET-EGF

>Taeniopygia_guttata_XM_002199809.1 .

INVSRQTLFEDSFQQIMALK----------------PYDLR-RRLYVIFR-----GEEGL

---DYGGLAREWFFLLSHEVLNP-------------------------------------

------------------------------------------------------------

------------------------------------------------------------

------------------------------------------------------------

------------------------------------------------------------

--------------------MYCLFEYAGKSN----------------------------

------------YCLQINPAS---------TINPDHLSYFCFIG--RFIAMALFHG----

-----KFIDTGFSL-PFYKRMLSKK-----------------------------------

----------------------------------------LTIKDLE---SIDTEFY---

NSLIWIRDNNIE------------------------------------------ECNLEM

YFCVDMEL-----------------------------LGKVTSHELKSGG----SNILVT

EENK--EEYIGLMAEWRFSRG-----VREQTKAFLDG---------------FNEVV---

--PLQWL-HYFDEKELEVMLCGMQE--------------------------VDLADWQRN

TVYRH----------------YTRNSKQIIWFWQFVK-ETDNEV----RMRLLQFVTGTC

RLPLGGFAELMGSN----GPQKFCIEKVGK---------------------------ETW

LP-RSHTCFN-----RLDLPP-YKSYEQ--LKEKLLFAIEET-EGF

>Xenopus_tropicalis_NM_001097375.1 .

ITVSRQTLFEDSFQQIMALK----------------PYDLR-RRLYVMFR-----GEEGL

---DYGGLAREWFFLLSHEVLNP-------------------------------------

------------------------------------------------------------

------------------------------------------------------------

------------------------------------------------------------

------------------------------------------------------------

--------------------MYCLFEYAGKSN----------------------------

------------YCLQINPAS---------TINPDHLSYFCFIG--RFIAMALFHG----

-----KFIDTGFSL-PFYKRMLSKK-----------------------------------

----------------------------------------LTIKDLE---SIDPEFY---

NSLIWIRDNNIE------------------------------------------ECNLEM

YFSVDMEI-----------------------------LGKVTSHDLKPEG----SNILVT

EENK--EEYIGLMAEWRFSRG-----VEEQTKAFLDG---------------FNAVV---

--PLQWL-QYFDEKELEVMLCGMQE--------------------------VDLSDWQRN

TVYRH----------------YTRNSKQIIWFWQFVK-EMDNEV----RLRLLQFVTGTC

RLPLGGFAELMGSN----GPQKFCIEKVGK---------------------------ETW

LP-RSHTCFN-----RLDLPP-YKSYEQ--LKEKLLFAIEET-EGF

>Danio_rerio_XM_001920534.1 .

INVSRKTLFEDSFQQIMSFS----------------AQDLR-RRLWIIFP-----GEEGL

---DYGGVARQWFFLLSHEVLNP-------------------------------------

------------------------------------------------------------

------------------------------------------------------------

------------------------------------------------------------

------------------------------------------------------------

--------------------MYCLFEYAGKDN----------------------------

------------YCLQINPAS---------YINPDHLKYFKFIG--RFIAMALFHG----

-----KFIDTGFSL-PFYKRILNKP-----------------------------------

----------------------------------------LTLKDLE---SIDPEFY---

NSLIWIKDNNIE------------------------------------------ECGLEM

FFSVDKEI-----------------------------LGEVSTHELKPDG----GNIQVT

EENK--EEYIRLVAEWRLSRG-----VEEQTQAFFEG---------------FNEVL---

--PQQYL-QYFDAKELEVMLCGMQE--------------------------IDLNDWQRN

TIYRH----------------YTSSSKQIMWFWQFIK-EMDNEK----RMRLLQFVTGTC

RLPVGGFADLMGSN----GPQKFCIEKVGK---------------------------ENW

LP-RSHTCFN-----RLDLPP-YRSYEQ--LKEKLMFAIEET-EGF

>Gasterosteus_aculeatus_DN679946.1 .

--VTRKTLFEDSFQQIMSFN----------------AQDLR-RRLWIIFP-----GEEGL

---DYGGVAREWFFLLSHEVLNP-------------------------------------

------------------------------------------------------------

------------------------------------------------------------

------------------------------------------------------------

------------------------------------------------------------

--------------------MYCLFEYAGKDN----------------------------

------------YCLQINPAS---------YINPDHLKYFKFIG--RFIAMALFHG----

-----KFIDTGFSL-PFYKRILNKP-----------------------------------

----------------------------------------LALKDLE---SIDPEFY---

NSLMWIKDNNIE------------------------------------------ECGLEL

FFSVDKEI-----------------------------LGEVSTHELKPDG----GELQVT

EENK--EEYIRLVAEWRMSRG-----VEEQTQAFFEG---------------FNEVL---

--PQQYL-QYFDAKELEVMLCGMQE--------------------------IDLVDWQRS

TIYRH----------------YARNSKQIVWFWQFIK-EMDNXK----RMRLLQFVTGTC

RLPVGSFADLMGKH----GPQKFCIEKGGK---------------------------RNW

LP-RSQRAS------QTGLPP-YRA---------------------

>Xenopus_tropicalis_NM_001079269.1 .

ITVNRKTLFEDSFQQIMSFN----------------AQDLR-RRLWIIIP-----GEEGL

---DYGGVAREWFFLLSHEVMNP-------------------------------------

------------------------------------------------------------

------------------------------------------------------------

------------------------------------------------------------

------------------------------------------------------------

--------------------MYCLFEYAGKDN----------------------------

------------YCLQINPAS---------YINPDHLRYFRFIG--RFIAMALFHG----

-----KFIDTGFSL-PFYKRILNKP-----------------------------------

----------------------------------------VGLKDLE---SVDPEFY---

NSLIWIKDNNIE------------------------------------------ECGLEM

FFSVDKEI-----------------------------LGDVKSHDLKPDG----SNIQVT

EENK--EEYIRLVAEWRLSRG-----VEEQTQAFFEG---------------FNEIL---

--PQQYL-QYFDAKELEVLLCGMQE--------------------------IDLNDWQRN

TIYRH----------------YTRTSKQIIWFWQFVK-EIDNEK----RMRLLQFVTGTC

RLPVGGFADLMGSN----GPQKFCIEKVGK---------------------------ENW

LP-RSHTCFN-----RLDLPP-YKSYEQ--LKEKLLFAIEET-EGF

>Gallus_gallus_XM_417330.2 .

ITVSRKTLFEDSFQQIMSFS----------------PQDLR-RRLWVIFP-----GEEGL

---DYGGVAREWFFLLSHEVLNP-------------------------------------

------------------------------------------------------------

------------------------------------------------------------

------------------------------------------------------------

------------------------------------------------------------

--------------------MYCLFEYAGKDN----------------------------

------------YCLQINPAS---------YINPDHLKYFRFIG--RFIAMALFHG----

-----KFIDTGFSL-PFYKRILNKP-----------------------------------

----------------------------------------VGLKDLE---SVDPEFY---

NSLIWVKENDIE------------------------------------------ECGLEM

FFSVDKEI-----------------------------LGEIKSHDLKPNG----SNILVT

EENK--EEYIRLVAEWRLSRG-----VEEQTQAFFEG---------------FNEIL---

--PQQYL-QYFDAKELEVLLCGMQE--------------------------IDLNDWQRH

TIYRH----------------YTRTSRQILWFWQFVK-EIDNEK----RMRLLQFVTGTC

RLPVGGFADLMGSN----GPQKFCIEKVGK---------------------------ENW

LP-RSHTCFN-----RLDLPP-YKNYEQ--LKEKLLFAIEET-EGF

>Erinaceus_europaeus_AANN01709963.1 .

ITVTRKTLFGDFFQQIMNFS----------------PQDIG-GHLWVIFP-----GEEGL

---DYGGIAREWCFLLSHKVLNP-------------------------------------

------------------------------------------------------------

------------------------------------------------------------

------------------------------------------------------------

------------------------------------------------------------

--------------------MYCLFEYAGKDN----------------------------

------------YCLQINPAS---------YINPDHLKYFRFVG--RFIAMTLFHE----

-----KFIDTGFSL-PFYKHILNKP-----------------------------------

----------------------------------------VGLKDLE---IL--------

------------------------------------------------------------

-------------------------------------------------G----GNILVT

EENK--E-YIRMVAEWRLSRG-----VEEQTQAFFEG---------------FNEIL---

--PQKYL-QYFDAKELEVLLCGMQE--------------------------IALNDWQRH

AIYHH----------------YTRTSKQIMWFWQFVK-ETD-KK----RMQLLQFVTGTC

LLPVGRFADLMGSK----GPQKFYVEKVGK---------------------------K-W

LP-RS---FN-----HLDLPP-YKSYEQ--LKEKFSFAIEET-EGF

>Monodelphis_domestica_XM_001381351.1 .

ITVTRKTLFEDSFQQIMSFN----------------PQDLR-RRLWVIFP-----GEEGL

---DYGGVAREWFFLLSHEVLNP-------------------------------------

------------------------------------------------------------

------------------------------------------------------------

------------------------------------------------------------

------------------------------------------------------------

--------------------MYCLFEYAGKDN----------------------------

------------YCLQINPAS---------YINPDHLKYFRFIG--RFIAMALFHG----

-----KFIDTGFSL-PFYKRILNKP-----------------------------------

----------------------------------------VGLKDLE---SIDPEFY---

NSLIWVKENNIE------------------------------------------ECGLEM

YFSVDKEI-----------------------------LGEIKSHELKPNG----SNILVT

EENK--EEYIRMVAEWRLSRG-----VEEQTQAFFEG---------------FNEIL---

--PQQYL-QYFDAKELEVLLCGMQE--------------------------IDLNDWQRH

AIYRH----------------YTRTSKQIMWFWQFVK-EIDNEK----RMRLLQFVTGTC

RLPVGGFADLMGSN----GPQKFCIEKVGK---------------------------ENW

LP-RSHTCFN-----RLDLPP-YKSYEQ--LKEKLLFAIEET-EGF

>Macaca_fascicularis_AB169137.1 .

ITVTRKTLFEDSFQQIMSLS----------------PQDLR-RRLWVIFP-----GEEGL

---DYGGVAREWFFLLSHEVLNP-------------------------------------

------------------------------------------------------------

------------------------------------------------------------

------------------------------------------------------------

------------------------------------------------------------

--------------------MYCLFEYAGKDN----------------------------

------------YCLQINPAS---------YINPDHLKYFRFIG--RFIAMALFHG----

-----KIIDTSFSL-PFYKRILSKP-----------------------------------

----------------------------------------VGLKDLE---SIDPEFY---

NSLIWVKENNIE------------------------------------------ECGLEM

YFSVDKEI-----------------------------LGEIKSHDLKPNG----GNILVT

EENK--EEYIRMVAEWRLSRG-----VEEQTQAFFEG---------------FNEIL---

--PQQYV-QYFDAKELEVLLCGMQE--------------------------IDLNDWQRH

AIYRH----------------YTRTSKQIMWFWQFVK-EIDNEK----RMRLLQFVTGTC

RLPLGGFADLMGSN----GPQKFCIEKVGK---------------------------ENW

LP-RSHTCFN-----RLDLPP-YKSYEQ--LKEKLLFAIEET-EGF

>Canis_familiaris_XM_858627.1 .

ITVTRKTLFEDSFQQIMSFN----------------PQDLR-RRLWVIFP-----GEEGL

---DYGGVAREWFFLLSHEVLNP-------------------------------------

------------------------------------------------------------

------------------------------------------------------------

------------------------------------------------------------

------------------------------------------------------------

--------------------MYCLFEYAGKDN----------------------------

------------YCLQINPAS---------YINPDHLKYFRFIG--RFIAMALFHG----

-----KFIDTGFSL-PFYKRILNKP-----------------------------------

----------------------------------------VGLKDLE---SIDPEFY---

NSLIWVKENNIE------------------------------------------ECGLEM

YFSVDKEI-----------------------------LGEIKSHDLKPNG----GNILVT

EENK--EEYIRMVAEWRLSRG-----VEEQTQAFFEG---------------FNEIL---

--PQQYL-QYFDAKELEVLLCGMQE--------------------------IDLNDWQRH

AIYRH----------------YTRTSKQIMWFWQFVK-EIDNEK----RMRLLQFVTGTS

--------EAERSN----GPQKFCIEKVGK---------------------------ENW

LP-RSHTCFN-----RLDLPP-YKSYEQ--LKEKLLFAIEET-EGF

>Pan_troglodytes_XR_024621.1 .

ITVTRKTLFEDSFQQIMSFS----------------PQDLR-RRLWVIFP-----GEEGL

---DYGGVAREWFFLLSHEVLNP-------------------------------------

------------------------------------------------------------

------------------------------------------------------------

------------------------------------------------------------

------------------------------------------------------------

--------------------MYCLFEYAGKDN----------------------------

------------YCLQINPAS---------YINPDHLKYFRFIG--RFIAMALFHG----

-----KFIDTGFSL-PFYKRILNKP-----------------------------------

----------------------------------------VGLKDLE---SIDPEFY---

NSLIWVKENNIE------------------------------------------ECDLEM

YFSVDKEI-----------------------------LGEIKSHDLKPNG----GNILVT

EENK--EEYIRMVAEWRLSRG-----VEEQTQAFFEG---------------FNEIL---

--PQQYL-QYFDAKELEVLLCGMQE--------------------------IDLNDWQRH

AIYRH----------------YARTSKQIMWFWQFVK-EIDNEK----RMRLLQFVTGTC

RLPVGGFADLMGSN----GPQKFCIEKVGK---------------------------ENW

LP-RSHTCFN-----RLDLPP-YKSYEQ--LKEKLLFAIEET-EGF

>Bos_taurus_DAAA01013200.1 .

ITVTRKTLFEDSFQQIMSFS----------------PQDLR-R-LWVIFP-----GEEGL

---DYGGVAREWFFLLSHEVLNP-------------------------------------

------------------------------------------------------------

------------------------------------------------------------

------------------------------------------------------------

------------------------------------------------------------

--------------------MYCLFEYAGKDN----------------------------

------------YCLQINPAS---------YINPDHLKYFRFIC--RFIAMALFHG----

-----KFIDTGFSL-PFYKRILNKP-----------------------------------

----------------------------------------VGLKDLE---SIDPEFY---

NSLIWVKENNIE------------------------------------------ECDLEM

YFSIDKEI-----------------------------LGEIKSHDLKPNG----GNILVT

EENK--EEYIRIVAEWRFSRG-----VEEQTQAFFEG---------------FNEIL---

--PQQYL-QYFDAKELEVLLCGIQE--------------------------IDLNDRQRH

AIYHH----------------YTRTSKQIMWFWQFVK-EIDNEK----RMRLLQFVTGTC

RLSVGGFADLMGSN----GPQKFCIEKVGK---------------------------ENW

LP-RRHTCFN-----RVDLPP-YKSYEQ--LKEKLLFVIEET-EGF

>Salmo_salar_BT059089.1 .

ISVSRQTLFEDSFQQIMNVK----------------PYDLR-RRLYIIMR-----GEEGL

---DYGGIAREWFFLLSHEVLNP-------------------------------------

------------------------------------------------------------

------------------------------------------------------------

------------------------------------------------------------

------------------------------------------------------------

--------------------MYCLFEYAGKNN----------------------------

------------YCLQINPAS---------SINPDHLTYFRFIG--RFIAMALYHG----

-----KFIDTGFTL-PFYKRMLNKK-----------------------------------

----------------------------------------PTLKDLE---SIDPEFY---

NSIMWVKDNSLE------------------------------------------EGGVEL

YFAQDMEI-----------------------------LGKVSTHQLKDDG----EDELVT

TENK--EEYISLLTDWRFTRG-----VEEQTKAFLDG---------------FNEVV---

--PLEWL-RYFDEKELELMLCGMQE--------------------------IDLSDWQKN

TIYRH----------------YTKNSKQIHWFWQVVK-EMDNEK----RIRLLQFVTGTC

RLPVGGFAELIGSN----GPQKFCIDKVGK---------------------------ETW

LP-RSHTCFN-----RLDLPP-YKSLEQ--LREKLLFAIEET-EGF

>Salmo_salar_BT059315.1 .

ISVSRQTLFEDSFQQIMNVK----------------PYDLR-RRLYIIMR-----GEEGL

---DYGGIAREWFFLLSHEVLNP-------------------------------------

------------------------------------------------------------

------------------------------------------------------------

------------------------------------------------------------

------------------------------------------------------------

--------------------MYCLFEYAGKNN----------------------------

------------YCLQINPAS---------SINPDHLTYFRFIG--RFIAMALYHG----

-----KFIDTGFTL-PFYKRMLNKK-----------------------------------

----------------------------------------PTLKDLE---SIDPEFY---

NSIMWVKDNCLE------------------------------------------ECGVEL

YFAQDMEI-----------------------------LGKVSTHQLKDDG----ENELVT

SENK--EEYISLLTDWRFMRG-----VEEQTKAFLDG---------------FNEVV---

--PLEWL-RYFDEKELELMLCGMQE--------------------------IDLSDWQKN

TIYRH----------------YTKNSKQIHWFWQVVK-EMDNEK----RIRLLQFVTGTC

RLPVGGFAELIGSN----GPQKFCIDKVGK---------------------------ETW

LP-RSHTCFN-----RLDLPP-YRSLEQ--LKEKLMFAIEET-EGF

>Danio_rerio_NM_001099448.1 .

ISVSRQTLFEDSFQQIMNMK----------------PYDLR-RRLYIIMR-----GEEGL

---DYGGIAREWFFLLSHEVLNP-------------------------------------

------------------------------------------------------------

------------------------------------------------------------

------------------------------------------------------------

------------------------------------------------------------

--------------------MYCLFEYAGKNN----------------------------

------------YCLQINPAS---------SINPDHLTYFRFIG--RFIAMALYHG----

-----KFIDTGFTL-PFYKRMLNKK-----------------------------------

----------------------------------------PTLKDLE---SIDPEFY---

NSIMWVKENDLE------------------------------------------ECGVEL

YFAQDMEI-----------------------------LGKVTTHQLKDDG----ENELVT

QDNK--EEYIGLLTDWRFTRG-----VEEQTKAFLDG---------------FNEVV---

--PLEWL-RYFDEKELELMLCGMQE--------------------------IDLNDWQKN

TIYRH----------------YTKNSKQIHWFWQVVK-EMDNEK----RIRLLQFVTGTC

RLPVGGFAELIGSN----GPQKFCIDKVGK---------------------------ETW

LP-RSHTCFN-----RLDLPP-YKNLEQ--LREKLLFAIEET-EGF

>Xenopus_tropicalis_NM_001097372.1 .

ISVSRQTLFEDSFQQIMNMK----------------PYDLR-RRLYIIMR-----GEEGL

---DYGGIAREWFFLLSHEVLNP-------------------------------------

------------------------------------------------------------

------------------------------------------------------------

------------------------------------------------------------

------------------------------------------------------------

--------------------MYCLFEYAGKNN----------------------------

------------YCLQINPAS---------SINPDHLTYFRFIG--RFIAMALYHG----

-----KFIDTGFTL-PFYKRMLNKK-----------------------------------

----------------------------------------PTLRDLE---SIDPEFY---

NSIMWIKDNNLE------------------------------------------ECELEL

YFVQDMEI-----------------------------LGEVTSHKLKEGG----ENIRVT

EENK--EEFISLLTDWRFTRG-----VEEQTNAFLDG---------------FKEVV---

--PLEWL-RYFDEKELELMLCGMQE--------------------------IDIADWQKN

TIYRH----------------YTKNSKQVQWFWQVVK-EMDNEK----RIRLLQFVTGTC

RLPVGGFVELIGSN----GPQKFCIDRVGK---------------------------DTW

LP-RSHTCFN-----RLDLPP-YKSYEQ--LKEKLLFAIEET-EGF

>Mus_musculus_AK159248.1 .

ISVSRQTLFEDSFQQIMNMK----------------PYDLR-RRLYIIMR-----GEEGL

---DYGGIAREWFFLLSHEVLNP-------------------------------------

------------------------------------------------------------

------------------------------------------------------------

------------------------------------------------------------

------------------------------------------------------------

--------------------MYCLFEYAGKNN----------------------------

------------YCLQINPAS---------SINPDHLTYFRFIG--RFIAMALYHG----

-----KFIDTGFTL-PFYKRMLNKR-----------------------------------

----------------------------------------PTLKDLE---SIDPEFY---

NSIVWIKENNLE------------------------------------------ECGLEL

FFIQDMEI-----------------------------LGKVTTHELKEGG----ENIRVT

EENK--EEYIMLLTDWRFTRG-----VEEQTKAFLDG---------------FNEVA---

--PLEWL-RYFDEKELELMLCGMQE--------------------------IDMSDWQKN

AIYRH----------------YTKSSKQIQWFWQVVK-EMDNEK----RIRLLQFVTGTC

RLPVGGFAELIGSN----GPQKFCIDRVGK---------------------------ETW

LP-RSHTCFN-----RLDLPP-YKSYEQ--LKEKLLYAIEET-EGF

>Pan_troglodytes_XM_511070.2 .

ISVSRQTLFEDSFQQIMNMK----------------PYDLR-RRLYIIMR-----GEEGL

---DYGGIAREWFFLLSHEVLNP-------------------------------------

------------------------------------------------------------

------------------------------------------------------------

------------------------------------------------------------

------------------------------------------------------------

--------------------MYCLFEYAGKNN----------------------------

------------YCLQINPAS---------SINPDHLTYFRFIG--RFIAMALYHG----

-----KFIDTGFTL-PFYKRMLNKR-----------------------------------

----------------------------------------PTLKDLE---SIDPEFY---

NSIVWIKENNLE------------------------------------------ECGLEL

YFIQDMEI-----------------------------LGKVTTHELKEGR----ESIRVT

-----------LLTDWRFTRG-----VEEQTKAFLDG---------------FNEVA---

--PLEWL-RYFDEKELELMLCGMQE--------------------------IDMSDWQKS

TIYRH----------------YTKNSKQIQWFWQVVK-EMDNEK----RIRLLQFVTGTC

RLPVGGFAELIGSN----GPQKFCIDKVGK---------------------------ETW

LP-RSHTCFN-----RLDLPP-YKSYEQ--LREKLLFAIEET-EGF

>Macaca_mulatta_XM_001101869.1 .

ISVSRQTLFEDSFQQIMNMK----------------PYDLR-RRLYIIMR-----GEEGL

---DYGGIAREWFFLLSHEVLNP-------------------------------------

------------------------------------------------------------

------------------------------------------------------------

------------------------------------------------------------

------------------------------------------------------------

--------------------MYCLFEYAGKNN----------------------------

------------YCLQINPAS---------SINPDHLTYFRFIG--RFIAMALYHG----

-----KFIDTGFTL-PFYKRMLNKR-----------------------------------

----------------------------------------PTLKDLE---SVDPEFY---

NSIVWIKENNLE------------------------------------------ECGLEL

YFIQDMEI-----------------------------LGKVTTHELKEGG----ESIRVT

EENK--EEYIMLLTDWRFTRG-----VEEQTKAFLDG---------------FNEVA---

--PLEWL-RYFDEKELELMLCGMQE--------------------------IDMSDWQKS

TIYRH----------------YTKNSKQIQWFWQVVK-EMDNEK----RIRLLQFVTGTC

RLPIGGFAELIGSN----GPQKFCIDKVGK---------------------------ETW

LP-RSHTCFN-----RLDLPP-YKSYEQ--LREKLLYAIEET-EGF

>Bos_taurus_NM_001083414.1 .

ISVSRQTLFEDSFQQIMNMK----------------PYDLR-RRLYIIMR-----GEEGL

---DYGGIAREWFFLLSHEVLNP-------------------------------------

------------------------------------------------------------

------------------------------------------------------------

------------------------------------------------------------

------------------------------------------------------------

--------------------MYCLFEYAGKNN----------------------------

------------YCLQINPAS---------SINPDHLTYFRFIG--RFIAMALYHG----

-----KFIDTGFTL-PFYKRMLNKR-----------------------------------

----------------------------------------PTLKDLE---SIDPEFY---

NSIVWIKENNLE------------------------------------------ECGLEL

YFIQDMEI-----------------------------LGKVTTHELKEGG----ESIRVT

EENK--EEYIMLLTDWRFTRG-----VEEQTKAFLDG---------------FNEVA---

--PLEWL-RYFDEKELELMLCGMQE--------------------------IDLSDWQKH

TIYRH----------------YTKNSKQIQWFWQVVK-EMDNEK----RIRLLQFVTGTC

RLPVGGFTELIGSN----GPQKFCIDKVGK---------------------------ETW

LP-RSHTCFN-----RLDLPP-YKSYEQ--LKEKLLYAVEET-EGF

>Monodelphis_domestica_XM_001367005.1 .

ISVSRQTLFEDSFQQIMNMK----------------PYDLR-RRLYIIMR-----GEEGL

---DYGGIAREWFFLLSHEVLNP-------------------------------------

------------------------------------------------------------

------------------------------------------------------------

------------------------------------------------------------

------------------------------------------------------------

--------------------MYCLFEYAGKNN----------------------------

------------YCLQINPAS---------SINPDHLTYFRFIG--RFIAMALYHG----

-----KFIDTGFTL-PFYKRMLNKR-----------------------------------

----------------------------------------PTLKDLE---SIDPEFY---

NSIVWIKENNLE------------------------------------------ECGLEL

YFIQDMEI-----------------------------LGKVTSHELKEGG----ESIRVT

EENK--EEYIMLLTDWRFTRG-----VEEQTKAFLDG---------------FNEVA---

--PLEWL-RYFDEKELELMLCGMQE--------------------------IDMADWQKN

TIYRH----------------YTKNSRPIQWFWQVVK-EMDNEK----RIRLLQFVTGTC

RLPVGGFAELIGSN----GPQKFCIDKVGK---------------------------ETW

LP-RSHTCFN-----RLDLPP-YKSYEQ--LKEKLLYAIEET-EGF

>Ornithorhynchus_anatinus_XM_001509995.1 .

ISVSRQTLFEDSFQQIMNMK----------------PYDLR-RRLYIIMR-----GEEGL

---DYGGIAREWFFLLSHEVLNP-------------------------------------

------------------------------------------------------------

------------------------------------------------------------

------------------------------------------------------------

------------------------------------------------------------

--------------------MYCLFEYAGKNN----------------------------

------------YCLQINPAS---------SINPDHLTYFRFIG--RFIAMALYHG----

-----KFIDTGFTL-PFYKRMLNKR-----------------------------------

----------------------------------------PTLKDLE---SIDPEFY---

NSIVWIKENNLE------------------------------------------ECGLEL

YFIQDMEI-----------------------------LGKVTTHELKEGG----ESIRVT

EENK--EEYIMLLTDWRFTRG-----VEEQTKAFLDG---------------FNEVA---

--PLEWL-RYFDEKELELMLCGMQE--------------------------IDMNDWQKN

TIYRH----------------YTKNSKQIQWFWQVIK-EMDNEK----RIRLLQFVTGTC

RLPVGGFAELIGSN----GPQKFCIDKVGK---------------------------ETW

LP-RSHTCFN-----RLDLPP-YKSYEQ--LKEKLLYAIEET-EGF

>Taeniopygia_guttata_XR_054448.1 .

ISVSRQTVFEDSFQQIMNMK----------------PYDLR-RRLYIIMR-----GEEGL

---DYGGIAREWFFLLSHEVLNP-------------------------------------

------------------------------------------------------------

------------------------------------------------------------

------------------------------------------------------------

------------------------------------------------------------

--------------------MYCLFEYAGKNN----------------------------

------------YCLQINPAS---------SINPDHLTYFRFIG--RFIAMALYHG----

-----KFIDTGFTL-PFYKRMLNKR-----------------------------------

----------------------------------------PTLKDLE---SIDPEFY---

NSIVWTKENSLE------------------------------------------ECGLEL

YFIQDMEI-----------------------------LGKVTTHELKEGG----ESIRVT

EENK--EEYIMLLTDWRFTRG-----VEEQTKAFLDG---------------FNEVV---

--PLEWL-RYFDEKELELMLCGMQE--------------------------IDMNDWQKN

TIYRH----------------YTKNSKQIQWFWQVVK-EMDNEK----RIRLLQFVTGTC

RLPVGGFAELIGSN----GPQKFCIDKVGK---------------------------ETW

LP-RSHTCFN-----RLDLPP-YKSYEQ--LKEKLLYAIEET-EGF

>Ciona_intestinalis_XM_002131701.1 .

INVSRQSIFEDSFSQIMHLQ----------------AYDLR-RRLYIMFK-----GEEGL

---DYGGVAREWFFLVSHEVLNP-------------------------------------

------------------------------------------------------------

------------------------------------------------------------

------------------------------------------------------------

------------------------------------------------------------

--------------------MYCLFEYAGSTN----------------------------

------------YTLQINPAS---------TINPDHMHYFRFVG--RFIAMALYHG----

-----KFIDTGFSL-PFYKRMLNRK-----------------------------------

----------------------------------------LTIKDIE---SVDEEFY---

NSLVWIRDNNIE------------------------------------------ECGLEL

DFTMDFEV-----------------------------LGKIDTIELKDGG----EDIPVT

EENK--EEYIRLMIDWRFSRG-----VEKQTKGFLDG---------------FNEVV---

--PLQWL-QYFDERELELMLCGMQE--------------------------FDVEDWSRH

SIYRN----------------YTKNSKQVLWFWQYIR-EIDNEK----RARLLQFVSGTC

RIPVGGFAELLGSN----GPQKFCIEKVGK---------------------------ETW

LP-RSHTCFN-----RLDLPP-YKSYEQ--LKEKLTMAIEET-EGF

>Nematostella_vectensis_XM_001641340.1 .

ITVSRTNLFEESFQQVMRYQ----------------PHDLR-RRLYITFK-----GEEGL

---DYGGVAREWFFLLSHEVLNP-------------------------------------

------------------------------------------------------------

------------------------------------------------------------

------------------------------------------------------------

------------------------------------------------------------

--------------------MYCLFEYANKNN----------------------------

------------YSLQINAAS---------SVNPDHLMYFKFIG--RFIAMALYHG----

-----KFIDRGFTL-PFYKRMLNKK-----------------------------------

----------------------------------------LLMKDLE---TIDPEFY---

NSLVWVKENNIE------------------------------------------ECGLEM

FFTVDMEL-----------------------------LGKVTSHDLKPGG----TDIAVT

EENK--EEYISLMTEWRLNRG-----IEEQTRAFLEG---------------MHEVL---

--PLYWI-QYFDERELELMLCGMQE--------------------------IDVEDWQQN

TVYRH----------------YTRNSKQVMWFWQAVK-AYDNEK----RIRLLQFVTGTC

RLPVGGFTELMGDN----GPQKFCIEKVGK---------------------------ETW

LP-RSHTCFN-----RLDLPP-YKSYEQ--LVEKLTFAIEET-EGF

>Nematostella_vectensis_XM_001641341.1 .

ITVSRTNLFEESFQQVMRYQ----------------PHDLR-RRLYITFK-----GEEGL

---DYGGVAREWFFLLSHEVLNP-------------------------------------

------------------------------------------------------------

------------------------------------------------------------

------------------------------------------------------------

------------------------------------------------------------

--------------------MYCLFEYANKNN----------------------------

------------YSLQINAAS---------SVNPDHLMYFKFIG--RFIAMALYHG----

-----KFIDRGFTL-PFYKRMLNKK-----------------------------------

----------------------------------------LLMKDLE---TIDPEFY---

NSLVWVKENNIE------------------------------------------ECGLEM

FFTVDMEL-----------------------------LGKVTSHDLKPGG----TDIAVI

EENK--EEYISLMTEWRLNRG-----IEEQTRAFLEG---------------MHEVL---

--PLYWI-QYFDERELELMLCGMQE--------------------------IDVEDWQQN

TVYRH----------------YTRNSKQVMWFWQAVK-AYDNEK----RIRLLQFVTGTC

RLPVGGFTELMGSN----GPQKFCIEKVGK---------------------------ETW

LP-RSHT---------------------------------------

>Branchiostoma_floridae_XM_002245239.1 .

ISVSRQTVFEDSFHQIMRLQ----------------PFDLR-RRLYIIFR-----GEEGL

---DYGGIAREWFFMLSHEVLNP-------------------------------------

------------------------------------------------------------

------------------------------------------------------------

------------------------------------------------------------

------------------------------------------------------------

--------------------MYCLFEYASKNN----------------------------

------------YSLQINPAS---------SVNPDHLMYFRFIG--RFIAMALYHG----

-----KFIDNGFTL-PFYKQMLSKK-----------------------------------

----------------------------------------LTMKDLE---SVDPEFY---

NSLQFIKENDID------------------------------------------ELDMEL

YFCADFEV-----------------------------LGKITTVDLKEDG----SNIRVT

EENK--DEYIILMVNWRFQRG-----VEEQTKAFLDG---------------FNDVV---

--PLQWL-QYFDERELELMLCGMQE--------------------------FDINDWQRN

TIYRH----------------YARNSKQVQWFWQYVK-ELDNEK----RARLLQFVTGTC

RLPVGGFAELMGIN----GPQRFCIEKVGK---------------------------ETW

LP-RSHTCFN-----RLDLPP-YKSYEQ--LVEKLNYAIEET-EGF

>Strong._purpuratus_XM_001195336.1 .

ISVTRNTLFEDSFHQIMRLQ----------------AFDLR-RRLYIIFR-----GEEGL

---DYGGVAREWFFMLSHEVLNP-------------------------------------

------------------------------------------------------------

------------------------------------------------------------

------------------------------------------------------------

------------------------------------------------------------

--------------------MYCLFEYANKNN----------------------------

------------YCLQINPAS---------SVNPDHLQYFRFVG--RFIAMALYHG----

-----KFIYSGFTM-PFYKRMLNKP-----------------------------------

----------------------------------------LSLRDLE---SIDPEFY---

NSLVWIKDNDID------------------------------------------ELDMEM

AFVADFEI-----------------------------LGKVETVDLKEGG----KDIDVS

EENK--EEYIHLMTQFRFNRG-----IEEQTKAFLEG---------------MNEVV---

--PLQWL-QYFDERELELMLCGMQE--------------------------FDVDDWYRC

TIYRH----------------YTRESKQVQWFWRAVR-EMDNEK----RARLLQFVTGTC

RLPVGGFTELMGSN----GPQKFCIEKVGK---------------------------ETW

LP-RSHTCFN-----RLDLPP-YKSYEQ--LTEKLTFAIEET-EGF

>Acyrthosiphon_pisum_XM_001943104.1 .

ITVSRQTLFEDSYHQIMRSA----------------AYEMR-KRLYIVFR-----GEEGL

---DYGGVSREWFFLLSHEVLNP-------------------------------------

------------------------------------------------------------

------------------------------------------------------------

------------------------------------------------------------

------------------------------------------------------------

--------------------MYCLFEYANKNN----------------------------

------------YSLQINPAS---------YVNPDHLLYFKFIG--RFIAMALYHG----

-----RFIYSGFTM-PFYKRMLNKK-----------------------------------

----------------------------------------LTMKDIE---SIDPEFY---

NSLVWIRDNNLE------------------------------------------ESDIEM

YFGVDFEV-----------------------------LGQVVHHELIENG----DKVKVT

DTNK--DEYIKLMTEWRMTRG-----IEEQTQALLDG---------------FNEVV---

--ALEWL-KYFDERELELMLCGMQE--------------------------IDVEDWQQH

TIYRH----------------YNRTSKQINWFWQFVK-QADNEK----RARLLQFVTGTC

RVPVGGFAELMGSN----GAQRFCIEKVGK---------------------------ETW

LP-RSHTCFN-----RLDLPP-YKSYDQ--LVEKLNYAIEET-EGF

>Tribolium_castaneum_AAJJ01000056.1 .

IQVSRQTLFEDSFHTIMRLP----------------AYELR-RRLYIIFK-----GEEGL

---DYGGVSREWFFLVSHEALNP-------------------------------------

------------------------------------------------------------

------------------------------------------------------------

------------------------------------------------------------

------------------------------------------------------------

--------------------MYCLFEYANKNN----------------------------

------------YSLQINPAS---------YVNPEHLTYFKFIG--RFIAMALYHG----

-----RFIYSGFTM-PFYKRMLGKK-----------------------------------

----------------------------------------LVMKDIE---SIDPEFY---

NSLVWIKENNID------------------------------------------ECGLEL

YYSVDFEV-----------------------------LGQVVHHELKKNG----DKEKVT

EENK--EEYLTLMTEWRMTRG-----IEQQTQAFLDG---------------FNEVV---

--PIEWL-KYFDERELELLLCGMQE--------------------------IDVDDWQRH

TIYRH----------------YTRSSKPVVWFWQFVR-QSDNEK----RARLLQFVTGTC

RVPVGGFAELMGSN----GPQKFCIEKVGK---------------------------ESW

LP-RSHTCFN-----RLDLPP-YKSYEQ--LVEKLTYAIEET-DTF

>Nasonia_vitripennis_XM_001603300.1 .

ISVSRQTLFEDSYHQIMNAE----------------AFALR-RRLYIIFK-----GEEGL

---DYGGVSREWFFLLSHEVLNP-------------------------------------

------------------------------------------------------------

------------------------------------------------------------

------------------------------------------------------------

------------------------------------------------------------

--------------------MYCLFEYANKSN----------------------------

------------YSLQINPAS---------YVNPDHLNYFKFIG--RFIAMALYHG----

-----RFIYSGFTM-PFYKRMLNKK-----------------------------------

----------------------------------------LIMKDIE---SIDPEFY---

KSLVWIKDNNID------------------------------------------ECGLEL

YYSVDFEI-----------------------------LGQVIHHELKEGG----DKVKVV

EDNK--EEYIRLMTEWRMTRG-----IEEQTKAFLEG---------------FNSVV---

--PLEWL-KYFDERELELMLCGMQE--------------------------IDVDDWQRN

TIYRH----------------YTRNSKQVLWFWQFVR-AADNEK----RARLLQFVTGTC

RVPVGGFAELMGSN----GPQRFCIEKVGK---------------------------DTW

LP-RSHTCFN-----RLDLPP-YKSYDQ--MVEKLNYAIEET-EGF

>Drosophila_mojavensis_XM_002004023.1 .

ITVTRQTLFEDSYHQIMRLP----------------AYELR-RRLYIIFR-----GEEGL

---DYGGVSREWFFLLSHEVLNP-------------------------------------

------------------------------------------------------------

------------------------------------------------------------

------------------------------------------------------------

------------------------------------------------------------

--------------------MYCLFEYANKNN----------------------------

------------YSLQINPAS---------YVNPDHLQYFKFIG--RFIAMALYHG----

-----RFIYSGFTM-PFYKRMLNKK-----------------------------------

----------------------------------------LTIKDIE---TIDPEFY---

NSLIWVRDNNID------------------------------------------ECGLEL

WFSVDFEV-----------------------------LGQIIHHELKENG----EKERVT

EENK--EEYITLMTEWRMTRG-----IEQQTKTFLEG---------------FNEVV---

--PLEWL-KYFDERELELILCGMQD--------------------------VDVEDWQRN

TIYRH----------------YNRNSKQVVWFWQFVR-ETDNEK----RARLLQFVTGTC

RVPVGGFAELMGSN----GPQRFCIEKVGK---------------------------ETW

LP-RSHTCFN-----RLDLPP-YKSYDQ--LVEKLTFAIEET-EGF

>Drosophila_persimilis_XM_002020540.1 .

FTVTRQTLLEDSYHQIMRLP----------------AYELR-RRLYIIFR-----GEEGL

---DYGGVSREWFFLLSHEVLNP-------------------------------------

------------------------------------------------------------

------------------------------------------------------------

------------------------------------------------------------

------------------------------------------------------------

--------------------MYCLFEYANKNN----------------------------

------------YSLQINPAS---------YVNPDHLQYFKFIG--RFIAMALYHG----

-----RFIYSGFTM-PFYKRMLNKK-----------------------------------

----------------------------------------LTIKDIE---TIDPEFY---

NSLIWVKDNNID------------------------------------------ECGLEL

WFSVDFEV-----------------------------LGQIIHHELKENG----EKERVN

EENK--EEYITLMTEWRMTRG-----IEQQTKTFLEG---------------FNEVV---

--PLEWL-KYFDERELELILCGMQD--------------------------VDVEDWQRN

TIYRH----------------YNRNSKQVVWFWQFVR-ETDNEK----RARLLQFVTGTC

RVPVGGFAELMGSN----GPQRFCIEKVGK---------------------------ETW

LP-RSHTCFN-----RLDLPP-YKSYDQ--LVEKLTFAIEET-EGF

>Anopheles_gambiae_XM_319824.3 .

ITLTRQTLFEDSYHQIMRLP----------------AYELR-RRLYIIFR-----GEEGL

---DYGGVSREWFFLLSHEVLNP-------------------------------------

------------------------------------------------------------

------------------------------------------------------------

------------------------------------------------------------

------------------------------------------------------------

--------------------MYCLFEYANKNN----------------------------

------------YSLQINPAS---------YVNPDHLQYFKFIG--RFIAMALYHG----

-----RFIYSGFTM-PFYKRMLNKK-----------------------------------

----------------------------------------LTTKDIE---TIDPEFY---

NSLIWVRDNNID------------------------------------------ECGLEL

WFSVDFEV-----------------------------LGQIIHHELKDNG----DKERVT

EENK--EEYISLMTEWRMTRG-----IEEQTKTFLDG---------------FNEVV---

--PLEWL-KYFDERELELMLCGMQE--------------------------IDVDDWQRN

SIYRH----------------YNRNSKQVVWFWQFVR-ETDNEK----RARLLQFVTGTC

RVPVGGFAELMGSN----GPQRFCIEKVGK---------------------------DTW

LP-RSHTCFN-----RLDLPP-YKSYDQ--LVEKLNYAIEET-EGF

>Culex_quinquefasciatus_XM_001845843.1 .

ITLTRQTLFEDSYHQIMRLP----------------AYELR-RRLYIIFR-----GEEGL

---DYGGVSREWFFLLSHEVLNP-------------------------------------

------------------------------------------------------------

------------------------------------------------------------

------------------------------------------------------------

------------------------------------------------------------

--------------------MYCLFEYANKNN----------------------------

------------YSLQINPAS---------YVNPDHLQYFKFIG--RFIAMALYHG----

-----RFIYSGFTM-PFYKRMLNKK-----------------------------------

----------------------------------------LTTKDIE---TIDPEFY---

NSLIWVRDNNID------------------------------------------ECGLEL

WFSVDFEV-----------------------------LGQIIHHELKEEG----DKEKVS

EENK--EEYISLMTEWRMTRG-----IEEQTKTFLEG---------------FNEVV---

--PLEWL-KYFDERELELMLCGMQE--------------------------IDVDDWQRN

SIYRH----------------YNRTSKQVVWFWQFVR-ETDNEK----RARLLQFVTGTC

RVPVGGFAELMGSN----GPQRFCIEKVGK---------------------------DTW

LP-RSHTCFN-----RLDLPP-YKSYDQ--LVEKLNYAIEET-EGF

>Brugia_malayi_XM_001901449.1 .

IAVSRNNLFEDSYQEIMRKN----------------PVDLR-RRLYIQFR-----GEEGL

---DYGGVA---------------------------------------------------

------------------------------------------------------------

------------------------------------------------------------

------------------------------------------------------------

------------------------------------------------------------

--------------------SYVLFLVKAN------------------------------

-------------------------------------IFWTCIG--RFIAMALFHG----

-----KFIYSGFTM-PFYKKMLRKK-----------------------------------

----------------------------------------FTLKDLE---SVDAEFY---

NSLMWIKENNVD------------------------------------------ECDMEL

YFVADYEL-----------------------------LGEIRTHELKDGG----AELKVC

EENK--EEYIELLMEWRFNRG-----IEQQTRAFFTG---------------FNSVF---

--PLEWL-QYFDERELELLLCGMQD--------------------------VDVDDWQRN

TVYRH----------------YAPQSKQVIWFWQFVR-SLDQVK----RSRLLQFVTGTC

RVPVGGFSELIGST----GPQLFCIERVGK---------------------------ENW

LP-RSHTCFN-----RLDLPP-YRSYEQ--LAEKLSRAIDET-EGF

>Caenorhabditis_elegans_NM_171831.2 .

ITVSRNNVFEDSFQEIMRKN----------------AVDLR-RRLYIQFR-----GEEGL

---DYGGVAREWFFLLSHEVLNP-------------------------------------

------------------------------------------------------------

------------------------------------------------------------

------------------------------------------------------------

------------------------------------------------------------

--------------------MYCLFMYAGNNN----------------------------

------------YSLQINPAS---------FVNPDHLKYFEYIG--RFIAMALFHG----

-----KFIYSGFTM-PFYKKMLNKK-----------------------------------

----------------------------------------IVLKDIE---QVDSEIY---

NSLMWIKDNNID------------------------------------------ECDMEL

YFVADYEL-----------------------------LGELKTYELKEGG----TEIAVT

EENK--LEYIELLVEWRFNRG-----VEQQTKAFFTG---------------FNSVF---

--PLEWM-QYFDERELELLLCGMQD--------------------------VDVDDWQRN

TVYRH----------------YAPQSKQVTWFWQWVR-SLDQEK----RARLLQFVTGTC

RVPVGGFSELMGST----GPQLFCIERVGK---------------------------ENW

LP-RSHTCFN-----RLDLPP-YRSYDQ--LVEKLSMAIEMT-EGF

>Caenorhabditis_briggsae_XM_001667457.1 .

ITVSRNNVFEDSFQEIMRKN----------------AVDLR-RRLYIQFR-----GEEGL

---DYGGVAREWFFLLSHEVLNP-------------------------------------

------------------------------------------------------------

------------------------------------------------------------

------------------------------------------------------------

------------------------------------------------------------

--------------------MYCLFMYAGNNN----------------------------

------------YSLQINPAS---------FVNPDHLKYFEYIG--RFIAMALFHG----

-----KFIYSGFTM-PFYKKMLNKK-----------------------------------

----------------------------------------IVLKDIE---QVDSEIY---

NSLMWIKDNNID------------------------------------------ECDMEL

YFVADYEL-----------------------------LGELKTYELKEGG----TDIAVT

EANK--LEYIELLVEWRFNRG-----VEQQTKAFFTG---------------FNSVF---

--PLEWM-QYFDERELELLLCGMQD--------------------------VDVDDWQRN

TVYRH----------------YAPQSKQVSWFWQWVR-SLDQEK----RARLLQFVTGTC

RVPVGGFSELMGST----GPQLFCIERVGK---------------------------ENW

LP-RSHTCFN-----RLDLPP-YRSYEQ--LVEKLSMAIEMT-EGF

>Trichoplax_adhaerens_XM_002111571.1 .

IAISRNTIFEDSYNAIISLK----------------PYDLR-KRLYIMFK-----GEDGL

---DYGGLAREWFFQLSHEMLNP-------------------------------------

------------------------------------------------------------

------------------------------------------------------------

------------------------------------------------------------

------------------------------------------------------------

--------------------MYCLFEYANQNN----------------------------

------------YSLQINAAS---------SVNPDHLKYFKFVG--RVIAMALYHG----

-----KFIDNGFTL-PFYKRLLNRG-----------------------------------

----------------------------------------VSINDLE---QVDPEFY---

NSLNWIKDNNID------------------------------------------ECDMEM

FFTADMEI-----------------------------FGEIKTYELKTGG----SDIKVT

DENK--EEYINLMSHWRFTRG-----VEDQTKAFMEG---------------FYEVV---

--PLRWL-EFFNEKELEMMLCGMQE--------------------------IDVDDWQQN

TVYKH----------------YTKNSKQVMWFWQFVR-DRKNEQ----RIRLLQFITGTC

RVPIGGFSHLMGSN----GPQKFCIEKVGK---------------------------ESW

LP-RSHTCFN-----RLDLPP-YKSYDQ--LVEKLNFAIEET-EGF

>Monosiga_brevicollis_XM_001743985.1 .

MPIRRSSLFQDSFDCIMSEHP---------------DETGF-RRLFITFQ-----GEQGL

---DYGGVAREWFFLISHEMLDP-------------------------------------

------------------------------------------------------------

------------------------------------------------------------

------------------------------------------------------------

------------------------------------------------------------

--------------------MYCLFEYATANN----------------------------

------------YQLQINPNS---------HVNPEHLQYFRFVG--RVVALAIYHK----

-----KFIDNGFTL-PFYKRLLNKK-----------------------------------

----------------------------------------LVLQDLE---TVDPDFY---

KNLYWLLNNEID------------------------------------------DLELGL

VFTADSNE-----------------------------FGAVKEVELKAGG----KDIEVT

DANK--QEYVELMANFRLKRG-----VEEQTEAFLMG---------------FHEIL---

--PHQAI-EFFDEREMELLLIGMAE--------------------------FDVDAWEKH

TIYRN----------------YRKKDRQVAWFWEVVR-EFTQEQ----RARLLQFVTGSC

RLPVGGFAELQGSN----GPQPFCIERYND---------------------------HGA

LP-RSHTCFN-----RLDLPP-YKTKEA--MKQKLTMAIEET-EGF

>Caenorhabditis_briggsae_XM_001667683.1 .

MVVHRDTLFEDSYRHIMDKK----------------DYDLR-NKLWIEFF-----GETGL

---DYGGVTREWFFLLSHQIFNP-------------------------------------

------------------------------------------------------------

------------------------------------------------------------

------------------------------------------------------------

------------------------------------------------------------

--------------------YYGLFEYSATDN----------------------------

------------YTLQINPHSE--------ACNPEHLSYFHFIG--RIIGMAIYHG----

-----KLLDAFFIR-PFYKMMLGKK-----------------------------------

----------------------------------------ITLFDME---SVDNAYY---

NSLIYVKDND--------------------------------------------PADLEL

TFSLDDSI-----------------------------FGETQNVELIPGG----ANVAVT

EENK--EEYIEAVV----------------------------------------------

--PSNLL-RLFDANELELLMCGLQK--------------------------IDVKDWKAN

TIYKG---------------GYGPSSQVVHNFWKCIL-SFDNEM----RARVLQFVSGTS

RVPMNGFRELYGSN----GLQKFTIERWGS---------------------------ADM

LP-RAHTCFN-----RLDLPP-YTTFKE--LKSKLLTAIENS-EIF

>Caenorhabditis_brenneri_ABEG01020224.1 .

MVVHRDTLFEDSYRHIMDKK----------------DYDLR-NKLWIEFF-----GETGL

---DYGGVTREWFFLLSHQIFNP-------------------------------------

------------------------------------------------------------

------------------------------------------------------------

------------------------------------------------------------

------------------------------------------------------------

--------------------YYGLFEYSATDN----------------------------

------------YTLQINPHSE--------ACNPEHLSYFHFIG--RIIGMAIYHG----

-----KLLDAFFIR-PFYKMMLGKK-----------------------------------

----------------------------------------ITLFDME---SVDNAYY---

NSLIYVKDND--------------------------------------------PADLEL

TFSLDDSI-----------------------------FGETQNVELVPGG----ANIAVT

EENK--EEYIEAVISWRFVNR-----IEKQMNQILKGVQEVRNVISSKRTFVVLQVV---

--PSNLL-RLFDANELELLMCGLQK--------------------------IDVKDWKAN

TIYKG---------------GYGPSSQVVHNFWKCIL-SFDNEM----RARVLQFVSGTS

RVPMNGFRELYGSN----GLQKFTIERWGS---------------------------ADM

LP-RAHT---------------------------------------

>Caenorhabditis_elegans_NM_058464.5 .

MVVHRDTLFEDSYRHIMDKK----------------DYDLR-NKLWIEFF-----GETGL

---DYGGVTREWFFLLSHQIFNP-------------------------------------

------------------------------------------------------------

------------------------------------------------------------

------------------------------------------------------------

------------------------------------------------------------

--------------------YYGLFEYSATDN----------------------------

------------YTLQINPHSE--------ACNPEHLSYFHFIG--RIIGMAIYHG----

-----KLLDAFFIR-PFYKMMLGKK-----------------------------------

----------------------------------------ITLFDME---SVDNEYY---

NSLIYVKDND--------------------------------------------PADLEL

TFSLDDSI-----------------------------FGETQNIELIPNG----ANVPVT

EDNK--EEYIEAVV----------------------------------------------

--PSNLL-RLFDANELELLMCGLQK--------------------------IDVKDWKAN

TIYKG---------------GYGPSSQVVHNFWKCIL-SFDNEM----RARVLQFVSGTS

RVPMNGFRELYGSN----GLQKFTIERWGS---------------------------ADM

LP-RAHTCFN-----RLDLPP-YTTFKE--LKSKLLTAIENS-EIF

>Brugia_malayi_XM_001901127.1 .

ITVRRIQLFEDSYRQIMQLS----------------PTLLR-AKLWIEFE-----NETGL

---DYGGVAREWFYLLSHDIFSP-------------------------------------

------------------------------------------------------------

------------------------------------------------------------

------------------------------------------------------------

------------------------------------------------------------

--------------------YYGLFEYSATDN----------------------------

------------YTLQINPHSE--------TCNPEHLSYFHFIG--RVIGIAIYHG----

-----KLLDAFFIR-PFYKMMLGKP-----------------------------------

----------------------------------------ITLNDME---SVDNEYF---

NSLIYIKDNN--------------------------------------------PEDLDL

HFAVDEDV-----------------------------FGKMNSVELRDGG----AEEKVT

DANK--DEYIDLIIKWRFVSR-----VEEQMKALMKG---------------VHELI---

--PPNLL-SIFDPNELELLVCGLQK--------------------------IDVKDWKDN

TLYKG---------------GYSPSHPVIQNFWKCLL-AFNNEM----RARLLQFVTGTS

RVPMNGFRELYGSN----GPQKFTIERWGS---------------------------ADM

LP-RAHTCFN-----RIDLPP-YTSFHE--MKERLTTAIENS-EIF

>Trichoplax_adhaerens_XM_002110879.1 .

IRVKRSHILEDSFRSISAVKK---------------PDLLK-TRLWIEFD-----QESGL

---DYGGLAREWFYLLSHEIFNP-------------------------------------

------------------------------------------------------------

------------------------------------------------------------

------------------------------------------------------------

------------------------------------------------------------

--------------------YYGLFEYSANDN----------------------------

------------YTLQINPNSG--------LCNENHLAYFKFAG--RVAGMAVFHG----

-----KLLDAFFIA-PFYKMMLGKP-----------------------------------

----------------------------------------ITLDDME---AVDTEYY---

NSLQYIMEND--------------------------------------------PSELDL

LFSVDEET-----------------------------LGKVNQIDLKPNG----KDIPVT

EKNK--KEYIDLVIKWRFASR-----IKSQMDKFLEG---------------FRELV---

--SLERL-RIFDEREIELLMCGMGD--------------------------IDVHDWRRN

TNYKN---------------GYGDQHLVIQWFWQVVY-ALEKES----RLRLLQFVTGTS

RVPMNGFSELYGIN----GPQRFTIERWGK---------------------------FDQ

LP-RAHTCFN-----RIDLPE-YKSYQD--LHDKLIMAIECT-QGY

>Anopheles_gambiae_ABKQ02010606.1 .

IKVRRASILEDSYRVINSVTR---------------LDLLK-TKLWVEFE-----GETGL

---DYGGLAREWFYLLSKEMFNP-------------------------------------

------------------------------------------------------------

------------------------------------------------------------

------------------------------------------------------------

------------------------------------------------------------

--------------------YYGLFEYSAMDN----------------------------

------------YTLQINPNSG--------LCNEEHLNYFRFIG--RIAGMAIYHG----

-----KLLDAFFIR-PFYKMMLQKS-----------------------------------

----------------------------------------IDLKDME---AVDTEYY---

NSLLYIKEND--------------------------------------------PSTLML

TFSVDEES-----------------------------FGTTNQRELKPNG----ADLEVS

NENK--DEYIRLVIDWRFEAR-----VKDQMQAFLEG---------------VSSLV---

--PLHLL-KIFDENELELLMCGIQS--------------------------IDVNDWKKN

TMYKG---------------DYYANHAVVQWFWRAVL-SFNNEM----RSRLLQFVTGTS

RVPMNGFKELYGSN----GPQLFTIEKWGT---------------------------VNN

FP-RAHT---------------------------------------

>Anopheles_gambiae_XM_312819.4 .

IKVRRASILEDSYRVINSVTR---------------LDLLK-TKLWVEFE-----GETGL

---DYGGLAREWFYLLSKEMFNP-------------------------------------

------------------------------------------------------------

------------------------------------------------------------

------------------------------------------------------------

------------------------------------------------------------

--------------------YYG-FEYSAMDN----------------------------

------------YTLQINPNSG--------LPATRGL-INLFIG--RIAGMAIYHG----

-----KLLDAFFIR-PFYKMMLQKS-----------------------------------

----------------------------------------IDLKDME---AVDTEYY---

NSLLYIKEND--------------------------------------------PSTLML

TFSVDEES-----------------------------FGTTNQRELKPNG----ADLEVS

NENK--DEYIRLVIDWRFEAR-----VKDQMQAFLEG---------------VSSLV---

--PLHLL-KIFDENELELLMCGIQS--------------------------IDVNDWKKN

TMYKG---------------DYYANHAVVQWFWRAVL-SFNNEM----RSRLLQFVTGTS

RVPMNGFKELYGSN----GPQLFTIEKWGT---------------------------VNN

FP-RAHTCFN-----RLDLPP-YESYAQ--LKEKLISAIEGS-QGF

>Aedes_aegypti_XM_001653159.1 .

IKVRRASILEDSYRIINSVTK---------------VELLK-TKLWVEFE-----SEAGL

---DYGGLAREWFYLLSKEMFNP-------------------------------------

------------------------------------------------------------

------------------------------------------------------------

------------------------------------------------------------

------------------------------------------------------------

--------------------YYGLFEYSAMDN----------------------------

------------YTLQINPYSG--------LCNEEHLNYFKFIG--RIAGMAVYHG----

-----KLLDAFFIR-PFYKMMLQKQ-----------------------------------

----------------------------------------IDLKDME---SVDTEYY---

NSLLYIKEND--------------------------------------------PSELML

TFSVDEES-----------------------------FGTTSQRDLKPNG----ANIEVT

NDNK--DEYIKLVIDWRFVAR-----VKSQMHAFLEG---------------FGSLV---

--PLHLL-KIFDENELELLMCGIQS--------------------------IDVSDWKKN

TLYKG---------------DYYANHVVVQWFWRAVL-SFNNEM----RSRLLQFVTGTS

RVPMNGFKELYGSN----GPQMFTIEKWGT---------------------------TDN

FP-RAHTCFN-----RLDLPP-YESYSH--LKDKLVKAIEGS-QGF

>Culex_quinquefasciatus_XM_001862489.1 .

IKIRRTSILEDSYRIINSVTK---------------VDLLK-TKLWVEFE-----GEAGL

---DYGGLAREWFYLLSKEMFNP-------------------------------------

------------------------------------------------------------

------------------------------------------------------------

------------------------------------------------------------

------------------------------------------------------------

--------------------YYGLFEYSAMDN----------------------------

------------YTLQINPFSG--------LCNEEHLNYFKFIG--RIAGMAVYHG----

-----KLLDAFFIR-PFYKMMLQKQ-----------------------------------

----------------------------------------IDLRDME---SVDTEYY---

NSLLYIKEND--------------------------------------------PSELML

TFSVDEES-----------------------------FGTTSQRELKPDG----ANIEVT

NENK--DEYIRLVIEWRFVAR-----VKSQMQAFLEG---------------FGSLV---

--PLNLL-KIFDENELELLMCGIQS--------------------------IDVNDWKRN

TLYKG---------------DFYANHVVVQWFWRAVL-SFNNEM----RARLLQFVTGTS

RVPMNGFKELYGSN----GPQMFTIEKWGT---------------------------PAN

YP-RAHTCFN-----RLDLPP-YESYSQ--LKDKLVKAIEGT----

>XP_001862524.1 .

----------------------------------------------------------GL

---DYGGLAREWFYLLSKEMFNP-------------------------------------

------------------------------------------------------------

------------------------------------------------------------

------------------------------------------------------------

------------------------------------------------------------

--------------------YYGLFEYSAMDN----------------------------

------------YTLQINPFSG--------LCNEEHLNYFKFIG--RIAGMAVYHG----

-----KLLDAFFIR-PFYKMMLQKQ-----------------------------------

----------------------------------------IDLRDMES---VDTEYY---

NSLLYIKENDPS--------------------------------------------ELML

TFSVDEES-----------------------------FGTTSQRELKPDG----ANIEVT

NENK--DEYIRLVIEWRFVAR-----VKSQMQAFLEG---------------FGSLV---

--PLNLL-KIFDENELELLMCGIQS--------------------------IDVNDWKRN

TLYKG---------------DFYANHVVVQWFWRAVL-SFNNEM----RARLLQFVTGTS

RVPMNGFKELYGSN----GPQMFTIEKWGT---------------------------PAN

YP-RAHTCFN-----RLDLPP-YESYSQ--LKDKLVKAIEGTLREE

>Aedes_aegypti_XM_001655428.1 .

IKVRRVSILEDSYRIINSITK---------------TELLK-TKLWIEFE-----GEAGL

---DYGGLAREWFYLLSKEMFNP-------------------------------------

------------------------------------------------------------

------------------------------------------------------------

------------------------------------------------------------

------------------------------------------------------------

--------------------YYGLFEYSAMDN----------------------------

------------YTLQINPFSG--------LCNEDHLHYFKFIG--RVAGMAVYHG----

-----KLLDAFFIR-PFYKMMLQKP-----------------------------------

----------------------------------------IDLKDME---SVDMEYY---

NSLLWIKEND--------------------------------------------PSELML

TFCVDEET-----------------------------FGYTSQRELKPNG----ADIEVT

NENK--DEYIKLVIEWRFVAR-----VKDQMSAFLDG---------------FGQIV---

--PLNLL-KIFDENELELLMCGIQS--------------------------IDVKDWKRN

TLYKG---------------DYYANHVIIQWFWKAVL-SFSNEM----RSRLLQFVTGTS

RVPMNGFKELYGSN----GPQMFTIEKWGT---------------------------PEN

YP-RAHTCFN-----RLDLPP-YESYLA--LKDRLIKAIEGS-QGF

>Culex_quinquefasciatus_XM_001865114.1 .

IKVRRASILEDSYRIINSITK---------------VDLLK-TKLWIEFE-----GEAGL

---DYGGLAREWFYLLSKEMFNP-------------------------------------

------------------------------------------------------------

------------------------------------------------------------

------------------------------------------------------------

------------------------------------------------------------

--------------------YYGLFEYSAMDN----------------------------

------------YTLQINPFSG--------LCNEDHLHYFKFIG--RVAGMAVYHG----

-----KLLDAFFIR-PFYKMMLQKP-----------------------------------

----------------------------------------IDLKDME---AVDMEYY---

NSLLWIKEND--------------------------------------------PSELML

TFCVDEET-----------------------------FGYTSQRELKPNG----ADIEVT

NDNK--DEYIKLVIEWRFVAR-----VKDQMQAFLEG---------------FGQIV---

--PLNML-KIFDENELELLMCGIQS--------------------------IDVKDWKRN

TLYKG---------------DYFANHVIIQWFWRAVL-SFSNEM----RARLLQFVTGTS

RVPMNGFKELYGSN----GPQMFTIEKWGT---------------------------PEN

YP-RAHT---------------------------------------

>Anopheles_gambiae_XM_311327.4 .

IKVRRASIMEDSYRIINSVTR---------------LDLLK-TKLWIEFE-----GEAGL

---DYGGLAREWFYLLSKEMFNP-------------------------------------

------------------------------------------------------------

------------------------------------------------------------

------------------------------------------------------------

------------------------------------------------------------

--------------------YYGLFEYSAMDN----------------------------

------------YTLQINPYSE--------LCNEDHLLYFRFIG--RIAGMAVYHG----

-----KLLDAFFIR-PFYKMMLQKP-----------------------------------

----------------------------------------IDLKDME---AVDTEYY---

NSLVWIKEND--------------------------------------------PSELML

TFCVDEET-----------------------------FGQTTQHELLPNG----AAIDVT

NENK--DEYIRLVIQWRFVSR-----VQVQMQAFLDG---------------FGSLV---

--PLNLL-KIFDEHELELLMCGIQN--------------------------IDLRDWKRN

TLYKG---------------DYYPNHVVIQWFWRAVL-SFSNEM----RARLLQFVTGTS

RVPMNGFKELYGSN----GPQMFTIEKWGT---------------------------PEN

YP-RAHTCFN-----RLDLPP-YESYQQ--LKDRLIQAVEGS-QGF

>Drosophila_melanogaster_ACN62428.1 .

--------------------------------------------LWVEFE-----GETGL

---DYGGLAREWFYLLSKEMFNP-------------------------------------

------------------------------------------------------------

------------------------------------------------------------

------------------------------------------------------------

------------------------------------------------------------

--------------------YYGLFEYSAMDN----------------------------

------------YTLQINNGSG--------LCNEEHLSYFKFIG--RIAGMAVYHG----

-----KLLDAFFIR-PFYKMMLQKP-----------------------------------

----------------------------------------IDLKDMES---VDTEYY---

NSLMWIKENDPR--------------------------------------------ILEL

TFCLDEDV-----------------------------FGQKSQHELKPGG----ANIDVT

NENK--DEYIKLVIEWRFVAR-----VKEQMSSFLDG---------------FGSII---

--PLNLI-KIFDEHELELLMCGIQN--------------------------IDVKDWREN

TLYKG---------------DYHMNHIIIQWFWRAVL-SFSNEM----RSRLLQFVTGTS

RVPMNGFKELYGSN----GPQMFTIEKWGT---------------------------PNN

FP-RAHTCFN-----RLDLPP-YEGYLQ--LKDKLIKAIEGS----

>Drosophila_willistoni_XM_002068913.1 .

IRIRRTSILEDSYRIISSVTK---------------TDLLK-TKLWVEFE-----GETGL

---DYGGLAREWFYLLSKEMFNP-------------------------------------

------------------------------------------------------------

------------------------------------------------------------

------------------------------------------------------------

------------------------------------------------------------

--------------------YYGLFEYSAMDN----------------------------

------------YTLQINNGSG--------LCNEEHLSYFKFIG--RIAGMAVYHG----

-----KLLDAFFIR-PFYKMMLQKP-----------------------------------

----------------------------------------IDLKDME---SVDTEYY---

NSLMWIKEND--------------------------------------------PRILEL

TFCLDEDV-----------------------------FGQKSQHELKTGG----ANIEVT

NENK--DEYIKLVIEWRFVAR-----VKEQMSAFLDG---------------FGSII---

--PLNLI-KIFDEHELELLMCGIQN--------------------------IDVKDWREN

TLYKG---------------DYHMNHIIIQWFWRAVL-SFSNEM----RSRLLQFVTGTS

RVPMNGFKELYGSN----GPQMFTIEKWGT---------------------------PNN

FP-RAHTCFN-----RLDLPP-YEGYLQ--LKDKLIKAIEGS-QGF

>Drosophila_mojavensis_XM_002007416.1 .

IRIRRTSILEDSYRIISSVTK---------------TDLLK-TKLWVEFE-----GETGL

---DYGGLAREWFYLLSKEMFNP-------------------------------------

------------------------------------------------------------

------------------------------------------------------------

------------------------------------------------------------

------------------------------------------------------------

--------------------YYGLFEYSAMDN----------------------------

------------YTLQINNGSG--------LCNEEHLSYFKFIG--RIAGMAVYHG----

-----KLLDAFFIR-PFYKMMLQKS-----------------------------------

----------------------------------------IDLKDME---SVDTEYY---

NSLMWIKEND--------------------------------------------PRILEL

TFCLDEDV-----------------------------FGQKSQHELKAGG----ANIEVS

NENK--DEYIKLVIEWRFVAR-----VKDQMTAFLDG---------------FGSII---

--PLNLI-KIFDEHELELLMCGIQN--------------------------IDVKDWREN

TLYKG---------------DYHMNHIIIQWFWRAVL-SFSNEM----RSRLLQFVTGTS

RVPMNGFKELYGSN----GPQMFTIEKWGT---------------------------PNN

FP-RAHTCFN-----RLDLPP-YEGYLQ--LKDKLIKAIEGS-QGF

>Drosophila_ananassae_XM_001957650.1 .

IRIRRTSILEDSYRIISSVTK---------------TDLLK-TKLWVEFE-----GETGL

---DYGGLAREWFYLLSKEMFNP-------------------------------------

------------------------------------------------------------

------------------------------------------------------------

------------------------------------------------------------

------------------------------------------------------------

--------------------YYGLFEYSAMDN----------------------------

------------YTLQINNGSG--------LCNEEHLSYFKFIG--RIAGMAVYHG----

-----KLLDAFFIR-PFYKMMLQKP-----------------------------------

----------------------------------------IDLKDME---SVDTEYY---

NSLMWIKEND--------------------------------------------PRILEL

TFCLDEDV-----------------------------FGQKSQHELKPGG----ANIDVT

NENK--DEYIKLVIEWRFVAR-----VKEQMSVFLDG---------------FGSII---

--PLNLI-KIFDEHELELLMCGIQN--------------------------IDVRDWREN

TLYKG---------------DYHMNHIIIQWFWRAVL-SFSNEM----RSRLLQFVTGTS

RVPMNGFKELYGSN----GPQMFTIEKWGT---------------------------PNN

YP-RAHTCFN-----RLDLPP-YEGYLQ--LKDKLIKAIEGS-QGF

>Drosophila_grimshawi_XM_001983800.1 .

IRIRRTSILEDSYRIISSVTK---------------TDLLK-TKLWVEFE-----GETGL

---DYGGLAREWFYLLSKEMFNP-------------------------------------

------------------------------------------------------------

------------------------------------------------------------

------------------------------------------------------------

------------------------------------------------------------

--------------------YYGLFEYSAMDN----------------------------

------------YTLQINNGSG--------LCNEEHLSYFKFIG--RIAGMAVYHG----

-----KLLDAFFIR-PFYKMMLQKP-----------------------------------

----------------------------------------IDLKDME---SVDTEYY---

NSLMWIKEND--------------------------------------------PRTLEL

TFCLDEDV-----------------------------FGQKSQHELKPGG----ANIDVT

NDNK--DEYIKLVIEWRFVAR-----VKDQMSSFLDG---------------FGSII---

--PLNLI-KIFDEHELELLMCGIQN--------------------------IDVKDWREN

TLYKG---------------DYHMNHIIIQWFWRAVL-SFSNEM----RSRLLQFVTGTS

RVPMNGFKELYGSN----GPQMFTIEKWGT---------------------------PNN

FP-RAHTCFN-----RLDLPP-YEGYLQ--LKDKLIKAIEGS-QGF

>Drosophila_pseudoobscura_XM_001354098.2 .

IRIRRTSILEDSYRIISSVTK---------------TDLLK-TKLWVEFE-----GETGL

---DYGGLAREWFYLLSKEMFNP-------------------------------------

------------------------------------------------------------

------------------------------------------------------------

------------------------------------------------------------

------------------------------------------------------------

--------------------YYGLFEYSAMDN----------------------------

------------YTLQINNGSG--------LCNEEHLSYFKFIG--RIAGMAVYHG----

-----KLLDAFFIR-PFYKMMLQKP-----------------------------------

----------------------------------------IDLKDME---SVDTEYY---

NSLMWIKEND--------------------------------------------PRILEL

TFCLDDDV-----------------------------LGQKSQHDLKPGG----ANIDVT

NENK--DEYIKLVIEWRFVAR-----VKEQMSSFLDG---------------FGSII---

--PLNLI-KIFDEHELELLMCGIQN--------------------------IDVKDWREN

TLYKG---------------DYHMNHIIIQWFWRAVL-SFSNEM----RSRLLQFVTGTS

RVPMNGFKELYGSN----GPQMFTIEKWGT---------------------------PNN

FP-RAHTCFN-----RLDLPP-YEGYLQ--LKDKLIKAIEGS-QGF

>Pediculus_humanus_EEB13366.1 .

----------------------------------------------------------GL

---DYGGLAREWFFLLSKEMFNP-------------------------------------

------------------------------------------------------------

------------------------------------------------------------

------------------------------------------------------------

------------------------------------------------------------

--------------------YYGLFEYSATDN----------------------------

------------YTLQINPLSG--------MCNEEHLNYFKFIG--RIAGMAVYHG----

-----KLLDAFFIR-PFYKMMLEKP-----------------------------------

----------------------------------------IDLKDMES---VDSEYY---

NSLLWIKENDPS--------------------------------------------ELEL

TFSIDEDS-----------------------------LGMTSQRELKPGG----ANIAVT

DENK--DEYINLVIKWRFVSR-----VQEQMNAFLEG---------------FSGLV---

--PLAFI-KIFDENELELLMCGIQN--------------------------IDVKDWKQN

TLYKG---------------DYHPNHIVVQWFWRVVL--TFNKEM---RARLLQFTTGTS

RVPMNGFKELYGSN----GPQLFTIEKWGT---------------------------PDN

YP-RAHTCFN-----RIDLPP-YESYQQ--LREKL-----------

>Acyrthosiphon_pisum_XM_001949884.1 .

IKVRRSNILEDSFRIINSMH----------------PDKLR-AKLWVEFE-----QEVGL

---DYGGLAREWFFLLSKEMFNP-------------------------------------

------------------------------------------------------------

------------------------------------------------------------

------------------------------------------------------------

------------------------------------------------------------

--------------------YYGLFEYSAMDN----------------------------

------------YTLQINAMSG--------LCNEEHLHYFKFIG--TVAGMAVYHG----

-----KLLDAFFIR-PFYKMMLEKP-----------------------------------

----------------------------------------IELKDME---SVDSEYY---

NSLLWIKEND--------------------------------------------PSELDL

TFSLDEDS-----------------------------LGHTSHRELKPDG----ANIHLT

QENK--DEYISLVIQWRFVSR-----IQDQMNAFLQG---------------FGSIV---

--PLSYI-KIFDENEMELLMCGIQN--------------------------IDVKDWKEN

THYKG---------------DYSPNNIVIQWFWRGVL-SFNNEM----RSRLLQFVTGTS

RVPMNGFKELYGSN----GPQLFTIERWGT---------------------------KEN

YP-RAHTCFN-----RLDLPP-YESYHE--LRNKLIKAIEGS-QGF

>Tribolium_castaneum_XM_969846.2 .

IKVKRRSILEDSFRVITSVPR---------------VELLK-TKLWIEFE-----GEVGL

---DYGGLAREWFYLLSKEMFNP-------------------------------------

------------------------------------------------------------

------------------------------------------------------------

------------------------------------------------------------

------------------------------------------------------------

--------------------YYGLFEYSAMDN----------------------------

------------YTLQINPFSG--------LCNEEHLNYFKFIG--RVAGMAVYHG----

-----KLLDAFFIR-PFYKMMLGKT-----------------------------------

----------------------------------------IDLKDME---SVDSEYY---

KSLLWIKEND--------------------------------------------PSGLDL

TFSVDEES-----------------------------LGHTTVHELIEGG----ANIPLD

NTNK--DDYIKCIIQWRFVGR-----VQEQMNAFLSG---------------FNDLI---

--PLSIV-KIFDEHELELLMCGIQH--------------------------IDVKDWKQN

TLYKG---------------DYHANHIVIQWFWRVVL-SFSNEM----RARLLQFVTGTS

RVPMNGFKELYGSN----GPQLFTIEKWGT---------------------------PEN

FP-RAHTCFN-----RLDLPP-YESYQH--LKDKLIKAIEGS-QGF

>Nasonia_vitripennis_NM_001159858.1 .

IKVGRNNILEDSYRIISSVNR---------------VEILK-TKLWVEFE-----GEVGL

---DYGGLAREWFFLLSKEMFNP-------------------------------------

------------------------------------------------------------

------------------------------------------------------------

------------------------------------------------------------

------------------------------------------------------------

--------------------YYGLFEYSAMDN----------------------------

------------YTLQINPFSG--------VCNEEHLNYFKFIG--RIAGMAVYHG----

-----KLLDAFFIR-PFYKMMLSKT-----------------------------------

----------------------------------------IDLKDME---SVDTEYY---

NSLLWIKEND--------------------------------------------PSELEL

TFCVDEES-----------------------------FGHTSQRELKTNG----ANVPVT

NENK--DEYISLVIQWRFVSR-----VQEQMNAFLEG---------------FNALV---

--PLTLV-KIFDENELELLMCGIQH--------------------------IDVKDWKQN

TLYKG---------------DYHANHITVQWFWRVVL-SFNNEM----RARLLQFVTGTS

RVPMNGFKELYGSN----GPQLFTIEKWGT---------------------------PDN

YP-RAHTCFN-----RIDLPP-YESYQQ--LRDKLIKAIEGS-QGF

>Apis_mellifera_XM_395191.3 .

IKVGRNNILEDSYRIISSVNR---------------VEILK-TKLWVEFE-----GEVGL

---DYGGLAREWFFLLSKEMFNP-------------------------------------

------------------------------------------------------------

------------------------------------------------------------

------------------------------------------------------------

------------------------------------------------------------

--------------------YYGLFEYSATDN----------------------------

------------YTLQINPFSG--------VCNEEHLNYFKFIG--RIAGMAVYHG----

-----KLLDAFFIR-PFYKMMLGKS-----------------------------------

----------------------------------------IDLKDME---SVDSEYY---

NSLLWIKEND--------------------------------------------PSELEL

TFCVDEES-----------------------------FGHTSQRELKPDG----ANIPLT

DENK--DEYIALVIQWRFVSR-----VQEQMNAFLEG---------------FNALI---

--PPTLV-KIFDEHELELLMCGIQH--------------------------IDVRDWKQN

TLYKG---------------DYHANHIVVQWFWRVVL-SFSNEM----RSRLLQFVTGTS

RVPMNGFKELYGSN----GPQLFTIEKWGT---------------------------PEN

YP-RAHTCFN-----RIDLPP-YESYQQ--LREKLIKAIEGS-QGF

>Ixodes_scapularis_XP_002412402.1 .

-------------------------------------------------D-----GEEVL

---DYGGASREFFYLLSREMFNP-------------------------------------

------------------------------------------------------------

------------------------------------------------------------

------------------------------------------------------------

------------------------------------------------------------

--------------------YYGLFEYSAADN----------------------------

------------YTLQINPCSG--------MCNEDHLSYFKFIG--RMAGMAVYHG----

-----KLLDAFFIR-PFYKMMLGKP-----------------------------------

----------------------------------------ITIKDMES---VDTEYY---

NSLRWIMDND--------------------------------------------PADLDL

RFSVDEDL-----------------------------FGQMQQRELTAGG----ADLAVT

QENK--GRYVDLVIQWRFASR-----VRPQMNAFLEG---------------LNELV---

--PLALL-RLFDEHELELLMCGIGQ--------------------------IDVRDWRRH

TVYKG---------------GYHANHVVVQWFWRLVL-SFSNEM----RSRLLQFVTGTS

RVPMNGFAELHGSN----GPQPFTLERWGS---------------------------PSN

LP-RSHTCFN-----RLDLPM-YESYQD--LREKLVQAIEGS----

>Nematostella_vectensis_XM_001638084.1 .

IHIRRRNLMEDSYRAVQLSIVK--------------PEILK-SRLWIVFD-----GETGL

---DYGGLQREWFYLLSKEVFNP-------------------------------------

------------------------------------------------------------

------------------------------------------------------------

------------------------------------------------------------

------------------------------------------------------------

--------------------YYGLFEYSASDN----------------------------

------------YTLQINPNSG--------LCNEEHLSYFKFIG--RVAGMAVYHG----

-----KLLDAFFIR-PFYKMMLGRP-----------------------------------

----------------------------------------ITLIDME---SVDSEYY---

NSLNWILEND--------------------------------------------PEDLDL

HFCVDEEL-----------------------------FGILSVKDLKPNG----SQTNVT

NENK--REYINLVIKWRFVSR-----VEDQMRAFMEG---------------FCDLI---

--PHNLI-QIFDERELELLMCGLGE--------------------------IDTVDWRKN

SNYRG---------------EYHDKHIVIQWFWKAVN-SFDIET----RARLLQFVTGTS

RVPMNGFSELYGSN----GPQRFTIEPWGT---------------------------PHS

LP-RAHTCFN-----RLDLPR-YRSYYE--LRERLRIAIENT-QGF

>Hydra_magnipapillata_XM_002166686.1 .

IHVRRNNLFQDSYRFVLHSCTN--------------VELLK-TKLWIVFD-----GETGL

---DYGGVARXWFYLLSKEMFNP-------------------------------------

------------------------------------------------------------

------------------------------------------------------------

------------------------------------------------------------

------------------------------------------------------------

--------------------YYGLFEYSATDN----------------------------

------------YTLQINSNSG--------IANEQHLSYFTFIG--RVAGMAVYHG----

-----KLLDAFFIR-PFYKMMLGKK-----------------------------------

----------------------------------------ITLKDME---SVDSEYH---

ESLNWLLSHD--------------------------------------------PTELDV

MFVLDYES-----------------------------FGTIVSKELITNG----ANIPVT

NENK--KQYIDLVIKWRFMDR-----VSEQMNAFMKG---------------FEDII---

--PRTAI-QVFDERELEYLLCGLGE--------------------------IDMEDWRKN

TQYRS---------------GYHDKHVVIQWFWKAVQ-TFDDEM----KARLLQFVTGTS

RVPMNGFAELYGSN----GPQKFTIERWGN---------------------------THQ

LP-RSHTCFN-----RIDLPP-YHSYHE--LREKLRLAIENT-EGF

>Branchiostoma_floridae_XM_002221727.1 .

MKLHRNSILEDSYRTIMACKK---------------ADNLK-AKLWIDFE-----GEKGL

---DYGGVAREWFYLLSHEMFNP-------------------------------------

------------------------------------------------------------

------------------------------------------------------------

------------------------------------------------------------

------------------------------------------------------------

--------------------YYGLFEYSANDN----------------------------

------------YTLQINPNSG--------LCNEEHLSYFKFIG--RVAGMAVYHG----

-----KLLDAFFIR-PFYKMMLKKP-----------------------------------

----------------------------------------ITLKDME---SVDSEYY---

NSLVWITEND--------------------------------------------PEDLDL

RFCVEEDQ-----------------------------FGQMVTKNLKANG----EDILVT

NSNK--KEYIDLVIKWRFSSR-----VQEQMKALMDG---------------FNELV---

--QQELL-SIFDEREVELLMCGLGD--------------------------IDVNDWRKH

TAYRG---------------DYSDKHPIIQWFWKAVI-LMDPET----RVRLLQFVTGTS

RVPMNGFAELWGSN----GPQKFTIEKWGN---------------------------PDQ

LP-RAHTCFN-----RVDLPP-YRSFQE--LWDKLKIAIENT-EGF

>Strong._purpuratus_XM_001193848.1 .

LKVHRNNLLEDSYRGISSIRS---------------ADLLK-ARLWIEFT-----GETGL

---DYGGVAREWFFLLSKEMFNP-------------------------------------

------------------------------------------------------------

------------------------------------------------------------

------------------------------------------------------------

------------------------------------------------------------

--------------------YYGLYEYSAMDN----------------------------

------------YTLQINPDSG--------ICNEDHISYFKFIG--RVAGMAVFHG----

-----KLLDAFFIR-PFYKMMAGKP-----------------------------------

----------------------------------------ITLRDME---SVDSEYH---

SSLQWITDND--------------------------------------------PVDLDL

TFAVDEES-----------------------------LGQTKTTELKPHG----ADIPLT

NENK--AEYIQLVIEWRFVNR-----VRKQMDAFMDG---------------FTSLV---

--PRELL-TMFDENEIELLLSGLGD--------------------------INVNDWRTN

TAYRG---------------GYHANHIVIQWFWKAVL-AMGAEM----RARLLQFVTGTS

RVPMNGFAHLYGSN----GPQLFTIEKWGS---------------------------ANA

LP-RAHT---------------------------------------

>Ciona_intestinalis_XM_002125038.1 .

MKVDRRTILNDSYRVISRVKK---------------PEFLK-SRLWIEFN-----KEKGL

---DYGGVAREWFYLLSKEMFNP-------------------------------------

------------------------------------------------------------

------------------------------------------------------------

------------------------------------------------------------

------------------------------------------------------------

--------------------YYGLFEYSATDN----------------------------

------------YTLQINPNSG--------MCNEDHFDWFRFIG--RVAGMAVYHG----

-----KLLDAFFIR-PFYKMMLGKP-----------------------------------

----------------------------------------ITLRDME---SVDSEYY---

NSLKWILEND--------------------------------------------PTDLDL

CFTVDEEL-----------------------------FGQMKVNELKPGG----ADIKVN

NENK--REYIQLVIKWRFVSR-----VQEQMKSFLQG---------------FNELI---

--PSNLV-KIFDENEVELLMCGLGD--------------------------VDVNDWRRN

TNYKG---------------DYSANHIVIQWFWRAVL-LMDPEL----RVRFLQFVTGTS

RVPMNGFGELWGSN----GPQLFTIEKWGT---------------------------PEK

LP-RAHTCFN-----RLDLPP-YKSFEE--LRKKLLLAIEST-QGF

>Pan_troglodytes_AY410992.1 .

---------EESYRRIMSVKR---------------PDVLK-ARLWIEFE-----SEKGL

---DYGGVAREWXXLLSKEMFNP-------------------------------------

------------------------------------------------------------

------------------------------------------------------------

------------------------------------------------------------

------------------------------------------------------------

--------------------YYGLFEYSATDN----------------------------

------------YTLQINPNSG--------LCNEDHLSYFTFIG--RVAGLAVFHG----

-----KLLDXXFIR-PXXKXMLGKQ-----------------------------------

----------------------------------------ITXNDME---SVDSEYY---

NSLKWILEND--------------------------------------------PTELDL

MFCIDEEN-----------------------------FGQTYQVDLKPNG----SEIMVT

NENK--REYIDLVIQWRFVNR-----VQKQMNAFLEG---------------FTELL---

--PIDLI-KIFDENELELLMCGLGD--------------------------VDVNDWRQH

SIYKN---------------GYCPNHPVIQWFWKAVL-LMDAEK----RIRLLQFVTGTS

RVPMNGFAELYGSN----GPQLFTIEQWGS---------------------------PEK

LP-RAHTCFN-----RLDLPP-YETFED--LREKLLMAVENA-QGF

>Macaca_mulatta_XM_001088661.1 .

MKLHRNNIFEESYRRIMSVKR---------------PDVLK-ARLWIEFE-----SEKGL

---DYGGVAREWFFLLSKEMFNP-------------------------------------

------------------------------------------------------------

------------------------------------------------------------

------------------------------------------------------------

------------------------------------------------------------

--------------------YYGLFEYSATDN----------------------------

------------YTLQINPNSG--------LCNEDHLSYFTFIG--RVAGLAVFHG----

-----KLLDGFFIR-PFYKMMLGKQ-----------------------------------

----------------------------------------ITLNDME---SVDSEYY---

NSLKWILEND--------------------------------------------PTELDL

MFCIDEEN-----------------------------FGQTYQVDLKPNG----SEIMVT

NENK--REYIDLVIQWRFVNR-----VQKQMNAFLEG---------------FTELL---

--PIDLI-KIFDENELELLMCGLGD--------------------------VDVNDWRQH

SIYKN---------------GYCPNHPVIQWFWKAVL-LMDAEK----RIRLLQFVTGTS

RVPMNGFAELYGSN----GPQLFTIEQWGS---------------------------PEK

LP-RAHTCFN-----RLDLPP-YETFED--LREKLLMAVENA-QGF

>Oryctolagus_cuniculus_AAGW01364483.1 .

--------------------R---------------LDVLK-ARLWIEFE-----SEKGL

---DYGGVAREWFFLLSKEMFNP-------------------------------------

------------------------------------------------------------

------------------------------------------------------------

------------------------------------------------------------

------------------------------------------------------------

--------------------YYGLFEYSATDN----------------------------

------------YTLQINPNSG--------LCNEDHLSYFTFIG--RVAGLAVFHG----

-----KLLDGFFIR-PFYKMMLGKQ-----------------------------------

----------------------------------------ITLNDME---SVDSEYY---

NSLKWILEND--------------------------------------------PTELDL

MFCIDEEN-----------------------------FGQTYQVDLKPNG----SEIMVT

NENK--REYIDLVIQWRFVNR-----VQKQMNAFLEG---------------FTELL---

--PIDLI-KIFDENELELLMCGLGD--------------------------VDVNDWRQH

SIYKN---------------GYCPNHPVIQWFWKAVL-LMDAEK----RIRLLQFVTGTS

RVPMNGFAELYGSS----GPQLFTIEQWGS---------------------------PEK

LP-RAHTCFN-----R------------------------------

>Gallus_gallus_XM_424462.2 .

MKLHRNNIFEESYRRIMSVKR---------------PDVLK-ARLWIEFE-----SEKGL

---DYGGVAREWFFLLSKEMFNP-------------------------------------

------------------------------------------------------------

------------------------------------------------------------

------------------------------------------------------------

------------------------------------------------------------

--------------------YYGLFEYSATDN----------------------------

------------YTLQINPNSG--------LCNEDHLSYFTFIG--RVAGLAVYHG----

-----KLLDGFFIR-PFYKMMLGKP-----------------------------------

----------------------------------------ITLKDME---SVDSEYY---

NSLKWILEND--------------------------------------------PTELDL

MFCIDEEN-----------------------------FGQTYQVDLKPNG----SEIMVT

NENK--REYIDLVIQWRFVNR-----VQKQMNAFLEG---------------FTELL---

--PIDLI-KIFDENELELLMCGLGD--------------------------VDVNDWRQH

TIYKN---------------GYCPNHPVIQWFWKAVL-LMDAEK----RIRLLQFVTGTS

RVPMNGFAELYGSN----GPQLFTIEQWGT---------------------------PDK

LP-RAHTCFN-----RLDLPL-YESFDD--LREKLLMAVENA-QGF

>Danio_rerio_XM_682997.3 .

MKLHRNNIFEESYRRIMSLKR---------------PDSLK-ARLWIEFE-----SEKGL

---DYGGVAREWFFLLSKEMFNP-------------------------------------

------------------------------------------------------------

------------------------------------------------------------

------------------------------------------------------------

------------------------------------------------------------

--------------------YYGLFEYSATDN----------------------------

------------YTLQINPNSG--------LCNEDHLSYFKFIG--RVAGMAVYHG----

-----KLLDGFFIR-PFYKMMLGKQ-----------------------------------

----------------------------------------ITLNDME---SVDSEYY---

NSLKWILEND--------------------------------------------PTELDL

RFCIDEDN-----------------------------FGQTYQVDLKPSG----SDMVVT

NDNK--KEYIDLVIQWRFVNR-----VQKQMNAFLEG---------------FTELI---

--PIDLI-KIFDENELELLMCGLGD--------------------------VDVNDWRQH

TVYKN---------------GYCPNHPVIQWFWKAVL-LMDAEK----RIRLLQFVTGTS

RVPMNGFAELYGSN----GPQLFTIEQWGT---------------------------PDK

LP-RAHTCFN-----RLDLPM-YETFED--LREKLLMAVENA-QGF

>Xenopus_tropicalis_NM_001006726.1 .

MKLHRNNIFEESYRRIMSVKR---------------PDVLK-ARLWIEFE-----SEKGL

---DYGGVAREWFFLLSKEMFNP-------------------------------------

------------------------------------------------------------

------------------------------------------------------------

------------------------------------------------------------

------------------------------------------------------------

--------------------YYGLFEYSATDN----------------------------

------------YTLQINPNSG--------LCNEDHLSYFTFIG--RIAGLAVFHG----

-----KLLDGFFIR-PFYKMMLGKQ-----------------------------------

----------------------------------------ITLKDME---SVDSEYY---

NSLKWILEND--------------------------------------------PTELDL

RFCIDEEN-----------------------------FGQTYQVDLKPNG----SEMVVT

NDNK--REYIDLVIQWRFVNR-----VQKQMNAFLEG---------------FTELI---

--PIDLI-KIFDENELELLMCGLGD--------------------------VDVNDWRQH

TLYKN---------------GYCPNHPAIQWFWKAVL-LMDAEK----RIRLLQFVTGTS

RVPMNGFAELYGSN----GPQLFTIEQWGS---------------------------PDK

LP-RAHTCFN-----RLDLPP-YDSFED--LREKLLMAVENA-QGF

>Danio_rerio_NM_001034186.1 .

LSVRRNAVLEDSYRRILSVKR---------------SELLK-ARLWVEFE-----GEKGL

---DYGGVAREWFFLISKEMFNP-------------------------------------

------------------------------------------------------------

------------------------------------------------------------

------------------------------------------------------------

------------------------------------------------------------

--------------------YYGLFEYSATDN----------------------------

------------YTLQINPNSG--------LCNEDHLSYFKFIG--RVAGMAVYHG----

-----KLLDAFFIR-PFYKMMLQKP-----------------------------------

----------------------------------------ITLQDME---SVDSEYF---

NSLRWILEND--------------------------------------------PTDLDL

RFTIDEEL-----------------------------FGQTHQHELKPGG----ADIVVN

DTNK--KEYIHLVMQWRFVDR-----IQRQMTAFKEG---------------FYELI---

--PQDLI-KIFDENELELLMCGLGD--------------------------VDVNDWREN

TKYKN---------------GYNPNHPAIIWFWKTVL-LMDAEK----RIRLLQFVTGTS

RVPMNGFAELYGSN----GPQLFTIEQWGT---------------------------REK

LP-RAHTCFN-----RLDLPP-XESFEE--LREKLHMAIENA-QGF

>Taeniopygia_guttata_XM_002197579.1 .

MKIHRTTILEDSYRRIIAVKR---------------ADFLK-ARLWIEFD-----GEKGL

---DYGGVAREWFFLLSKEMFNP-------------------------------------

------------------------------------------------------------

------------------------------------------------------------

------------------------------------------------------------

------------------------------------------------------------

--------------------YYGLFEYSATDN----------------------------

------------YTLQINPNSG--------LCNEDHLSYFKFIG--RVAGMAVYHG----

-----KLLDAFFIR-PFYKMMLQKP-----------------------------------

----------------------------------------ITLHDME---SVDSEYY---

NSLRWILEND--------------------------------------------PTELDL

RFIVDEEL-----------------------------FGQTHQHELKSGG----SEIVVT

NKNK--RDYIHLVIQWRFVSR-----VQKQMTAFKEG---------------FFELI---

--PQDLI-KIFDENELELLMCGLGD--------------------------VDVADWKLH

TKYKN---------------GYNINHQVIQWFWKAVL-MMDSEK----RIRLLQFVTGTS

RVPMNGFAELYGSN----GPQLFTVEQWGT---------------------------PEK

LP-RAHTCFN-----RLDLPP-YDSFED--LWDKLLLAIENT-QGF

>Gallus_gallus_XM_001231786.1 .

MKIHRTTILEDSYRRIIAVKR---------------ADFLK-ARLWIEFD-----GEKGL

---DYGGVAREWFFLLSKEMFNP-------------------------------------

------------------------------------------------------------

------------------------------------------------------------

------------------------------------------------------------

------------------------------------------------------------

--------------------YYGLFEYSATDN----------------------------

------------YTLQINPNSG--------LCNEDHLSYFKFIG--RVAGMAVYHG----

-----KLLDAFFIR-PFYKMMLQKP-----------------------------------

----------------------------------------ITLHDME---SVDSEYY---

NSLRWILEND--------------------------------------------PAELDL

RFIVDEEL-----------------------------FGQTHQHELKSGG----SEIVVT

NKNK--RDYIHLVIQWRFVSR-----VQKQMAAFKEG---------------FFELI---

--PQDLI-KIFDENELELLMCGLGD--------------------------VDVADWKLH

TKYKN---------------GYSVNHQVIQWFWKAVL-MMDSEK----RIRLLQFVTGTS

RVPMNGFAELYGSN----GPQLFTVEQWGT---------------------------PEK

LP-RAHTCFN-----RLDLPP-YDSFED--LWDKLLLAIENT-QGF

>Ornithorhynchus_anatinus_XM_001511147.1 .

MKLRRTAILEDSYRRIIAVKR---------------ADFLK-ARLWIEFD-----GEKGL

---DYGGVAREWFFLISKEMFNP-------------------------------------

------------------------------------------------------------

------------------------------------------------------------

------------------------------------------------------------

------------------------------------------------------------

--------------------YYGLFEYSATDN----------------------------

------------YTLQINPNSG--------LCNEDHLSYFKFIG--RVAGMAVYHG----

-----KLLDGFFIR-PFYKMMLHKP-----------------------------------

----------------------------------------ITLHDME---SVDSEYY---

NSLQWIIEND--------------------------------------------PTELDL

RFTVDEEL-----------------------------FGQTHQHELKADG----SEIVVT

NKNK--KEYIHLVIQWRFVNR-----VQKQMAAFKEG---------------FFELI---

--PQDLI-KIFDENELELLMCGLGD--------------------------VDVNDWREH

TKYKN---------------GYNVNHQVIQWFWKAVL-MMDSEK----RIRLLQFVTGTS

RVPMNGFAELYGSN----GPQFFTVEQWGS---------------------------PDK

LP-RAHTCFN-----RLDLPP-YESFED--LWDKLHVAIENT-QGF

>Mus_musculus_BC138813.1 .

MKLRRANILEDSYRRIMGVKR---------------ADLLK-ARLWIEFD-----GEKGL

---DYGGVAREWFFLISKEMFNP-------------------------------------

------------------------------------------------------------

------------------------------------------------------------

------------------------------------------------------------

------------------------------------------------------------

--------------------YYGLFEYSATDN----------------------------

------------YTLQINPNSG--------LCNEDHLSYFKFIG--RVAGMAVYHG----

-----KLLDGFFIR-PFYKMMLQKL-----------------------------------

----------------------------------------ITLHDME---SVDSEYY---

SSLRWILEND--------------------------------------------PTELDL

RFIIDEEL-----------------------------FGQTHQHELKTGG----SEIVVT

NKNK--KEYIYLVIQWRFVNR-----IQKQMAAFKEG---------------FFELI---

--PQDLI-KIFDENELELLMCGLGD--------------------------VDVNDWREH

TKYKN---------------GYSMNHQVIHWFWKAVW-MMDSEK----RIRLLQFVTGTS

RVPMNGFAELYGSN----GPQSFTVEQWGT---------------------------PDK

LP-RAHTCFN-----RLDLPP-YESFDE--LWDKLQMAIENT-QGF

>Rattus_norvegicus_U50842.1 .

MKLRRANILEDSYRRIMGVKR---------------ADFLK-ARLWIEFD-----GEKGL

---DYGGVAREWFFLISKEMFNP-------------------------------------

------------------------------------------------------------

------------------------------------------------------------

------------------------------------------------------------

------------------------------------------------------------

--------------------YYGLFEYSATEDN---------------------------

------------YTLQINPNSG--------LCNEDHLSYFKFIG--RVAGMAVYHG----

-----KLLDGFFIR-PFYKMMLQKL-----------------------------------

----------------------------------------ITLHDME---SVDSEYY---

SSLRWILEND--------------------------------------------PTELDL

RFIIDEEL-----------------------------FGQTHQHELKTGG----SEVVVT

NKNK--KEYIYLVIQWRFVNR-----IQKQMAAFKEG---------------FFELI---

--PQDLI-KIFDENELELLMCGLGD--------------------------VDVNDWREH

TKYKN---------------GYSLNHQVIHWFWKAVL-MMDSEK----RIRLLQFVTGTS

RVPMNGFAELYGSN----GPQSFTVEQWGT---------------------------PDK

LP-RAHTCFN-----RLDLPP-YESFDE--LWDKLQMAIENT-QGF

>Equus_caballus_XM_001500779.1 .

MKLRRATVLEDSYRRIMGVKR---------------ADFLK-ARLWIEFD-----GEKGL

---DYGGVAREWFFLISKEMFNP-------------------------------------

------------------------------------------------------------

------------------------------------------------------------

------------------------------------------------------------

------------------------------------------------------------

--------------------YYGLFEYSATDN----------------------------

------------YTLQINPNSG--------LCNEDHLSYFKFIG--RVAGMAVYHG----

-----KLLDGFFIR-PFYKMMLHKP-----------------------------------

----------------------------------------ITLHDME---SVDGEYY---

NSLRWILEND--------------------------------------------PTELDL

RFVIDEEL-----------------------------FGQTHQHELKNGG----SEIVVT

NKNK--KEYIYLVIQWRFVNR-----IQKQMAAFKEG---------------FFELI---

--PQDLI-KIFDENELELLMCGLGD--------------------------VDVNDWREH

TKYKN---------------GYNVNHPVIQWFWKAVL-MMDSEK----RIRLLQFVTGTS

RVPMNGFAELYGSN----GPQSFTVEQWGT---------------------------PEK

LP-RAHTCFN-----RLDLPP-YESFEE--LWDKLQMAIENT-QGF

>Macaca_mulatta_XM_001088005.1 .

MKLRRATVLEDSYRRIMGVKR---------------ADFLK-ARLWIEFD-----GEKGL

---DYGGVAREWFFLISKEMFNP-------------------------------------

------------------------------------------------------------

------------------------------------------------------------

------------------------------------------------------------

------------------------------------------------------------

--------------------YYGLFEYSATDN----------------------------

------------YTLQINPNSG--------LCNEDHLSYFKFIG--RVAGMAVYHG----

-----KLLDGFFIR-PFYKMMLHKP-----------------------------------

----------------------------------------ITLHDME---SVDSEYY---

NSLRWILEND--------------------------------------------PTELDL

RFIIDEEL-----------------------------FGQTHQHELKNGG----SEIVVT

NKNK--KEYIYLVIQWRFVNR-----IQKQMAAFKEG---------------FFELI---

--PQDLI-KIFDENELELLMCGLGD--------------------------VDVNDWREH

TKYKN---------------GYSANHQVIQWFWKAVL-MMDSEK----RIRLLQFVTGTS

RVPMNGFAELYGSN----GPQSFTVEQWGT---------------------------PEK

LP-RAHTCFN-----RLDLPP-YESFEE--LWDKLQMAIENT-QGF

>Homo_sapiens_AL832359.1 .

MKLRRATVLEDSYRRIMGVKR---------------ADFLK-ARLWIEFD-----GEKGL

---DYGGVAREWFFLISKEMFNP-------------------------------------

------------------------------------------------------------

------------------------------------------------------------

------------------------------------------------------------

------------------------------------------------------------

--------------------YYGLFGYSATDS----------------------------

------------YTLQINPNSG--------LCNEDHLSYFKFIG--RVAGMAVYHG----

-----KLLDGFFIR-PFCKMMLHKP-----------------------------------

----------------------------------------ITLHDME---SVDSEYY---

NSLRWILEND--------------------------------------------PTELDL

RFIIDEEL-----------------------------FGQTHQHELKNGG----SEIVVT

NKNK--KEYIYLVIQWRFVNR-----IQKQMAAFKGG---------------FFELI---

--PQDLI-KIFDENELELLMCGLGD--------------------------VDVNDWREH

TKYKN---------------GYSANHQVIQWFWKAVL-MMDSEK----RIRLLQFVTGTS

RVPMNGFAELYGSN----GPQSFTVEQWGT---------------------------PEK

LP-KAHTCFN-----RLDLPP-YESFEE--LWDKLQMAIENT-QGF

>Trichoplax_adhaerens_XM_002109434.1 .

IEVTRSDVFEASYNQIMKMR----------------PKDLK-KRLTVKFK-----GEEGL

---DYGGVAREWLHLLSHEMLNP-------------------------------------

------------------------------------------------------------

------------------------------------------------------------

------------------------------------------------------------

------------------------------------------------------------

--------------------SYGLFTFSDDDM----------------------------

------------CCLQINQDS---------SINTNHLSYFHFVG--RVMGMGVFHG----

-----HHIDGTFPT-PFYKQLLNKA-----------------------------------

----------------------------------------CTIEDLE---SVDPGFY---

RSLCWLLNNDI-------------------------------------------TDDLEQ

NFCVEHQS-----------------------------FGEIVEYDLKPNG----SAIRVT

NDNK--YEYAELLVNWKLTHG-----IDEQLQALKKG---------------FYEIV---

--PTYLL-KNFHEKELELIIGGLKK--------------------------IDIQDWKAN

TRLKH----------------CTPSTDVVKWFWQIVD-SYCEEE----RMRLLQFVTGSS

RVPLQGFEALQGSLRDATGSRLFTINVVDIN--------------------------TDC

LP-KAHTCFN-----RLDFPP-YENYDK--MLQKLTCAIEET-CGF

>Drosophila_virilis_XM_002049819.1 .

LEVSRSEIFEESYRLIMKMR----------------AKDMR-KRLMVKFK-----GEEGL

---DYGGVAREWLHLLSREMLNP-------------------------------------

------------------------------------------------------------

------------------------------------------------------------

------------------------------------------------------------

------------------------------------------------------------

--------------------QYGLFQYSRDDH----------------------------

------------YTLQINPDS---------SVNPDHLSYFHFVG--RTLGIAVFHG----

-----HCLDGGFTT-PFYKQLLNKP-----------------------------------

----------------------------------------ITLGDIE---GVDPELH---

RSLTWMLESNI-------------------------------------------SGIIES

TFSVENNS-----------------------------FGALVVHELKPGG----ASIAVT

EENK--REYVKLYVNYRFMRG-----IEQQFLALQKG---------------FCELV---

--PSHLL-RPFDERELELVIGGISS--------------------------IDVNDWRNN

TRMKH----------------CTNETPQVLWFWQVVE-SYSSEM----RARLLQFVTGSS

RVPLQGFRALQGSTGAV-GPRLFTIHLTADVP-------------------------TQN

LP-KAHTCFN-----RIDLPP-YENYQL--LCDKLTQAVEET-CGF

>Drosophila_grimshawi_XM_001987208.1 .

LEVSRSEIFEESYRLIMKMR----------------AKDMR-KRLMVKFK-----GEEGL

---DYGGVAREWLHLLSREMLNP-------------------------------------

------------------------------------------------------------

------------------------------------------------------------

------------------------------------------------------------

------------------------------------------------------------

--------------------QYGLFQYSRDDH----------------------------

------------YTLQINPDS---------SVNPDHLSYFHFVG--RTLGIAVFHG----

-----HCLDGGFTT-PFYKQLLNKP-----------------------------------

----------------------------------------ITLGDIE---GVDPELH---

RSLTWMLESNI-------------------------------------------SGFFES

TFSVENNS-----------------------------FGALVVHELKPGG----ASIAVT

EENK--REYVKLYVNYRFMRG-----IEQQFLALQKG---------------FCELI---

--PSHLL-RPFDERELELVIGGISS--------------------------IDVNDWRNN

TRMKH----------------CTNETPQVLWFWQVVE-SYSFEM----RARLLQFVTGSS

RVPLQGFRALQGSTGAV-GPRLFTIHLTVDVP-------------------------TQN

LP-KAHTCFN-----RIDLPP-YENYQL--LCDKLTQAVEET-CGF

>Drosophila_yakuba_XM_002092020.1 .

LEVSRNEIFEESYRLIMKMR----------------AKDMR-KRLMVKFK-----GEEGL

---DYGGVAREWLHLLSREMLNP-------------------------------------

------------------------------------------------------------

------------------------------------------------------------

------------------------------------------------------------

------------------------------------------------------------

--------------------QYGLFQYSRDDH----------------------------

------------YTLQINPDS---------GVNPDHLSYFHFVG--RTLGIAVFHG----

-----HCLDGGFTT-PFYKQLLNKP-----------------------------------

----------------------------------------ITLGDIE---GVDPELH---

RSLTWMLESNI-------------------------------------------SGIIES

TFSVENNS-----------------------------FGALVVHELKPGG----ASIPVT

EENK--REYVKLYVNYRFMRG-----IEQQFLALQKG---------------FCELI---

--PSHLL-RPFDERELELVIGGISS--------------------------IDVNDWRNN

TRLKH----------------CTNETTQVLWFWQVVE-SYSSEM----RARLLQFVTGSS

RVPLQGFRALQGSTGAV-GPRLFTIHLTADVP-------------------------TQN

LP-KAHTCFN-----RIDLPP-YETYQL--LCDKLTQAVEET-CGF

>Drosophila_mojavensis_XM_002005956.1 .

LEVSRSEIFEESYRLIMKMR----------------AKDMR-KRLMVKFK-----NEEGL

---DYGGVAREWLHLLSREMLNP-------------------------------------

------------------------------------------------------------

------------------------------------------------------------

------------------------------------------------------------

------------------------------------------------------------

--------------------HYGLFQYSRDDH----------------------------

------------YTLQINPDS---------SINPDHLSYFHFVG--RILGIAVFHG----

-----HCLDGGFTT-PFYKQLLNKP-----------------------------------

----------------------------------------ITLGDIE---GVDPELH---

RSLTWMLESNI-------------------------------------------SGIIES

TFSVENNS-----------------------------FGALVVHELKPGG----ASIPVT

EENK--REYVKLYVNYRFMRG-----IEQQFLALQKG---------------FCELI---

--PSHLL-RPFDERELELVIGGISS--------------------------IDVNDWRSN

TRMKH----------------CTNETPQVQWFWQVVE-SYSSEM----RARLLQFVTGSS

RVPLQGFRALQGSTGAV-GPRLFTIHLTADVP-------------------------TQN

LP-KAHTCFN-----RIDLPP-YESYQL--LCDKLTQAVEET-CGF

>Culex_quinquefasciatus_XM_001846596.1 .

LEVSRHEIFEESYRLIMKMR----------------QRDMR-KRLMVKFK-----GEEGL

---DYGGVAREWLHLLSREMLNP-------------------------------------

------------------------------------------------------------

------------------------------------------------------------

------------------------------------------------------------

------------------------------------------------------------

--------------------QYGLFQYSGDDR----------------------------

------------YSLQINPDS---------GVNPDHLSYFHFVG--RILGIAVFHN----

-----HVLDGGFTL-PFYKQLLNKP-----------------------------------

----------------------------------------ITLSDIE---DVDPELH---

RSLTWMLENNI-------------------------------------------NGVLDS

TFSVENNS-----------------------------FGALKVHELKPNG----AQLVVT

EENK--REYVKLYVNYRFMRG-----IEQQFLALSKG---------------FGELI---

--PSHLL-RPFDERELELVVGGISK--------------------------IDVIDWKTH

TRLKQ----------------CTPETPQIVWFWQIVE-SYSPEM----RAQLLQFVTGSC

RVPLQGFRALQGSTGAV-GPRLFTIHLTADAP-------------------------IQN

LP-KAHTCFN-----RIDLPM-YDSYQL--MYDKLTQAVEET-CGF

>Anopheles_gambiae_XM_309403.3 .

LEVSRNEIFEESYRLIMKMR----------------PKDMR-KRLMVKFK-----GEEGL

---DYGGVAREWLYLLSHEMLNP-------------------------------------

------------------------------------------------------------

------------------------------------------------------------

------------------------------------------------------------

------------------------------------------------------------

--------------------QYGLFQYSRDDH----------------------------

------------YSLQINPDS---------AINPEHLSYFHFVG--RILGIAVFHN----

-----HVLDGGFTL-PFYKQLLNKP-----------------------------------

----------------------------------------ITLSDIE---DVDPDLH---

RSLTWILENNI-------------------------------------------TGIIDS

TFSVENNS-----------------------------FGVLKVHELKPNG----ASIAVT

EDNK--REYVKLYVNYRFMRG-----IEQQFLALSKG---------------FGELI---

--LSHLL-RPFDERELELLISGISK--------------------------IDVNDWKAN

TRLKQ----------------CTADTPQIVWFWQIVE-SYSPEM----RAQLLQFVTGSC

RVPLQGFRALQGSTGAV-GPRLFTIHLTADVP-------------------------LQN

LP-KAHTCFN-----RLDLPM-YDSYQL--MYDKLTQAVEET-CGF

>Tribolium_castaneum_XM_961336.2 .

LEVSRNEVFEESYRLIMKMR----------------PKDMR-KRLMVKFK-----GEEGL

---DYGGVAREWLHLLSREMLNP-------------------------------------

------------------------------------------------------------

------------------------------------------------------------

------------------------------------------------------------

------------------------------------------------------------

--------------------QYGLFQYSRDDH----------------------------

------------YTLQINPDS---------AVNPEHLSYFHFVG--RILGIAVFHN----

-----HQLEGGFTL-PFYKQLLNKP-----------------------------------

----------------------------------------ITLQDIE---GVDPELH---

RSLTWMLENNI-------------------------------------------DGVLDT

TFSVENNS-----------------------------FGIVKVHELKPSG----ATIPVT

EDNK--REYVKLYVNYRFMRG-----IEQQFLALQKG---------------FTELI---

--PPSLL-RPFDERELELVISGIGS--------------------------IDIADWRSH

TRLKH----------------CTPETPVVQWFWQVVE-SYSEEM----RARLLQFVTGSS

RVPLQGFKALQGSTGAA-GPRLFTIHCIDCS--------------------------PQN

LP-KAHTCFN-----RIDIPP-YDSYQT--LADKLTQAVEET-CGF

>Apis_mellifera_XM_396318.3 .

LEVSRNEIFEESYRLIMKMR----------------PKDMR-KRLMVKFR-----GEEGL

---DYGGVAREWLYLLSHEMLNP-------------------------------------

------------------------------------------------------------

------------------------------------------------------------

------------------------------------------------------------

------------------------------------------------------------

--------------------QYGLFQYSRDDN----------------------------

------------YTLQINPDS---------GINPEHLSYFHFAG--RIIGIAVFHG----

-----HHIDGGFTT-PFYKMLLNKA-----------------------------------

----------------------------------------ITLTDIE---GVDPELH---

RSLTWMLENSI-------------------------------------------DGVLDA

TFSVEHSS-----------------------------FGVLKNHELKPGG----KDIPVT

EENK--KEYVRLYVNYRFMRG-----IEQQFLALQKG---------------FHELI---

--PPQLL-RPFDERELELVIGGLGT--------------------------IDINDWKMH

TRLKH----------------CTPDTPVVKWFWQIVE-SYGEEM----RARLLQFVTGSS

RVPLQGFKALQGAA----GPRLFTIHAVDAP--------------------------SEN

LP-KAHTCFN-----RIDIPESYPSYQK--MLDKLTQAVEET-CGF

>Nasonia_vitripennis_XM_001606551.1 .

LEVSRNEIFEESYRLIMKMR----------------PKDMR-KRLMVKFR-----GEEGL

---DYGGVAREWLYLLSHEMLNP-------------------------------------

------------------------------------------------------------

------------------------------------------------------------

------------------------------------------------------------

------------------------------------------------------------

--------------------QYGLFQYSRDDN----------------------------

------------YTLQINADS---------GINPEHLSYFHFAG--RIIGIAVFHG----

-----HHVDGGFTT-PFYKMLLNKA-----------------------------------

----------------------------------------ITLSDIE---GVDPELH---

RSLTWMLENSI-------------------------------------------DGVLDA

TFSVEHSS-----------------------------FGVLKNHELKPGG----KDIPVT

EENK--KEYVRLYVNYRFMRG-----IEQQFLALQKG---------------FHELI---

--PSQLL-RPFDERELELVIGGLGT--------------------------IDISDWKQN

TRLKN----------------CTPDTPVVKWFWQIVE-SYGEEM----RARLLQFVTGSS

RVPLQGFKALQGSTGAA-GPRLFTIHAVDAP--------------------------SEN

LP-KAHTCFN-----RIDIPQTYPNYKK--MLDKLTQAVEET-CGF

>Bombyx_mori_NM_001146252.1 .

IEVSRNEILEESYRLVMKLR----------------GKELR-KRLLVKFR-----GEEGL

---DYGGVAREWLHLLGRELFNP-------------------------------------

------------------------------------------------------------

------------------------------------------------------------

------------------------------------------------------------

------------------------------------------------------------

--------------------HYGLFQYANAGEDR--------------------------

------------YALQINSDS---------GVNPEHLSYFHFAG--RILGVALFHG----

-----HQLDAAFTA-PFYKQLLGRP-----------------------------------

----------------------------------------ITLRDIK---DVDPELH---

RSLSWMLDNSI-------------------------------------------QGVIDT

TFSVECSS-----------------------------FGAVRSVELRPGG----ATEPVT

DGNK--REYVRLYVAHRFTRG-----AERQWLALQRG---------------LADVV---

--PPQLL-RPLSARDLQPLLAGRAD--------------------------LDPTDWRRH

TRLKH----------------LAPDSALAQWFWEIVD-EFDQEM----RARLLQFVTGSR

RVPLAGFRALQGSTGVA-APRLFTLHLVADAT-------------------------PDS

LP-KAHTCFN-----RLDLPP-YPTKQK--LHDKLKQAILET-AGF

>Hydra_magnipapillata_XM_002164072.1 .

IEVSRDDIFEQSYQSIKKTK----------------ARELR-KRLVVIFK-----NEVGL

---DFGGIAREWLYILSQEMFNP-------------------------------------

------------------------------------------------------------

------------------------------------------------------------

------------------------------------------------------------

------------------------------------------------------------

--------------------YYGLFKYSKDSQ----------------------------

------------YTLEVNPDS---------GVNPDHLSYFHFVG--RIVGIAVFHH----

-----HYLDGGFTM-PFYKQLLGKP-----------------------------------

----------------------------------------NTLEDLE---SVDPELY---

RSLKWVAENKI-------------------------------------------NDIIFQ

TFAVEHLS-----------------------------FGKTTLYDLKKDG----SQIPVT

DDNK--KEFVKLYVNYRLRHG-----VEMQFKAFMKG---------------FNELV---

--PQHII-KMFDERELELLICGLGK--------------------------IDIADWKAN

TRLKH----------------CSKDHNIVQWFWEIVD-SYDEEK----RARLLQFVTGSS

RVPVQGFKALQGSTGSN-GPRLFTISLINAD--------------------------IAS

LP-KSHTCFN-----RIDLPK-YESKSQ--LYEKLTLAIEET-CGF

>Hydra_magnipapillata_XM_002166314.1 .

-----------SYQSIMKTK----------------ARELR-KRLVVKFK-----NEVGL

---DFGGIAREWLYILSQEMFNP-------------------------------------

------------------------------------------------------------

------------------------------------------------------------

------------------------------------------------------------

------------------------------------------------------------

--------------------YYGLFKYSKDSQ----------------------------

------------YTLEVNPDS---------GVNPDHLSYFHFVG--RIVGIAVFHH----

-----HYLDGGFTM-PFYKQLLGKP-----------------------------------

----------------------------------------NTLEDLE---SVDPELY---

RSLKWVAENKI-------------------------------------------KDIIFH

TFAVEHLS-----------------------------FGKTTLYDLKKDG----SQIPVT

DDNK--KEFVKLYVNYRLRHG-----VEMQFKAFMKG---------------FNELV---

--PQHII-KMFDERELELLICGLGK--------------------------IDIADWKAN

TRLKH----------------CSKDHNIVQWFWEIVD-SYDEEK----RARLLQFVTGSS

RVPVQGFKALQGSTGSN-GPRLF-------------------------------------

--------FN-----RIDLPK-YESKSQ--LYEKLTLAIEET-CGF

>Strong._purpuratus_XR_026213.1 .

IEVGRENIFEESYRQIMKLK----------------PKDLR-KRLAIKFK-----SEEGL

---DYGGITREWLYLLSHEMLNP-------------------------------------

------------------------------------------------------------

------------------------------------------------------------

------------------------------------------------------------

------------------------------------------------------------

--------------------YYGLFQYSRDDI----------------------------

------------YTLQINPDS---------GVNPEHLSYFHFVG--RVIGLAIFHG----

-----HYIDGGFTM-PFYKQLLGKP-----------------------------------

----------------------------------------VSLEDME---TVDPEVY---

RSLVWILENDI-------------------------------------------TGILEN

TFAVEHNS-----------------------------FGQMQIHELKPNG----IDIPVT

EENK--KKYVKLFVQWRFLRG-----IEPQFLALQKG---------------FHELI---

--PPRLL-KPFDERELELLINGLGK--------------------------IDVEDWKNN

TRLKN----------------CSLETAIVQWFWQAVD-SFDEEK----RARLLQFVTGTS

KVPLEGFKALQGST----GALSPRLFTIHQIDAN-----------------------TDL

LP-KAHTCFN-----RLDLPP-YENYSK--FFDKLTCAIEET-CGF

>Ciona_intestinalis_XM_002127351.1 .

LEIKRSEAFEQSYSLIMKMK----------------PKDLK-KRLMVKFT-----GEDGL

---DYGGLAREWLYILSHEMLNP-------------------------------------

------------------------------------------------------------

------------------------------------------------------------

------------------------------------------------------------

------------------------------------------------------------

--------------------YYGLFQYSREDI----------------------------

------------YTLQINADS---------HINPDHLSYFHFVG--RILGMAVYHG----

-----HYIDGGFTM-LFYKQLLGKP-----------------------------------

----------------------------------------ISLEDME---EVDPALY---

KSMKWILQNDI-------------------------------------------NGVLDH

TFCVDQDS-----------------------------FGERVTHELKPNG----VNIPVT

EQNK--REYVKLYVNWRFLRG-----IEAQFLSLSKG---------------FYELI---

--PQHLL-RPFDERELELIIGGLGK--------------------------IDLIDWKKH

TKLKH----------------CSQDSNIVKWFWQAVE-SFDEEK----RARVLQFVTGSS

RVPLQGFKALQGSTGTQ-GPRLFTIQFVDNK--------------------------TDC

LP-KAHTCFN-----RIDLPP-YESYKK--LLEKLTCAVENT-CGF

>Branchiostoma_floridae_XM_002206560.1 .

IEVSREEIFEESYRQVMKMR----------------PKDLR-KRLMVKFK-----GEEGL

---DYGGVAREWLYLLSHEMLNP-------------------------------------

------------------------------------------------------------

------------------------------------------------------------

------------------------------------------------------------

------------------------------------------------------------

--------------------YYGLFQYSRESD----------------------------

------------YTLQINPDS---------GVNPEHLSYFHFVG--RIIGMAIFHG----

-----HYIDGGFTL-PLYKQLLGKP-----------------------------------

----------------------------------------ISLDDME---SVDPDLH---

RSLIWILENDI-------------------------------------------TGILDN

SFCVEQDS-----------------------------FGALQTHELKPNG----SDIPVT

EDNK--KEYVKLYVQWRFLHG-----IEAQFLSLQKG---------------LQEII---

--PPHLL-RPFDEKELELMVTGLGK--------------------------IDIDDWKAH

TRLKH----------------CTPDSTVVKWFWRAVE-SFDDER----RARLLQFVTGSS

RVPLQGFKALQGSTGAA-GPRLFTIHQIDAS--------------------------TDN

LP-KAHTCFN-----RIDIPP-YENYEK--LLEKLTCAVEET-CGF

>Mus_musculus_NM_001038627.1 .

IEVSREEIFEESYRQIMKMR----------------PKDLK-KRLMVKFR-----GEEGL

---DYGGVAREWLYLLCHEMLNP-------------------------------------

------------------------------------------------------------

------------------------------------------------------------

------------------------------------------------------------

------------------------------------------------------------

--------------------YYGLFQYSTDNI----------------------------

------------YTLQINPDS---------SINPDHLSYFHFVG--RIMGLAVFHG----

-----HYINGGFTV-PFYKQLLGKP-----------------------------------

----------------------------------------IQLSDLE---SVDPELH---

KSLVWILENDI-------------------------------------------TPVLDH

TFCVEHNA-----------------------------FGRILQHELKPNG----RNVPVT

EENK--KEYVRLYVNWRFMRG-----IEAQFLALQKG---------------FNELI---

--PQHLL-KPFDQKELELIIGGLDK--------------------------IDLNDWKSN

TRLKH----------------CVADSNIVRWFWQAVE-TFDEER----RARLLQFVTGST

RVPLQGFKALQGSTGAA-GPRLFTIHLIDAN--------------------------TDN

LP-KAHTCFN-----RIDIPP-YESYEK--LYEKLLTAVEET-CGF

>Homo_sapiens_DQ051601.1 .

IEVSREEIFEESYRQIMKMR----------------PKDLK-KRLMVKFR-----GEEGL

---DYGGVA---------------------------------------------------

------------------------------------------------------------

------------------------------------------------------------

------------------------------------------------------------

------------------------------------------------------------

------------------------------------------------------------

----------------------------------DHLSYFHFVG--RIMGLAVFHG----

-----HYINGGFTV-PFYKQLLGKP-----------------------------------

----------------------------------------IQLSDLE---SVDPELH---

KSLVWILENDI-------------------------------------------TPVLDH

TFCVEHNA-----------------------------FGRILQHELKPNG----RNVPVT

EENK--KEYVRLYVNWRFMRG-----IEAQFLALQKG---------------FNELI---

--PQHLL-KPFDQKELELIIGGLDK--------------------------IDLNDWKSN

TRLKH----------------CVADSNIVRWFWQAVE-TFDEER----RARLLQFVTGST

RVPLQGFKALQGSTGAA-GPRLFTIHLIDAN--------------------------TDN

LP-KAHTCFN-----RIDIPP-YESYEK--LYEKLLTAVEET-CGF

>Taeniopygia_guttata_XM_002190224.1 .

IEVSREEIFEESYRQIMKMR----------------PKDLK-KRLMVKFR-----GEEGL

---DYGGVAREWLYLLCHEMLNP-------------------------------------

------------------------------------------------------------

------------------------------------------------------------

------------------------------------------------------------

------------------------------------------------------------

--------------------YYGLFQYSTDNI----------------------------

------------YMLQINPDS---------SINPDHLSYFHFVG--RIMGLAVFHG----

-----HYINGGFTV-PFYKQLLGKP-----------------------------------

----------------------------------------IQLSDLE---SVDPELH---

KSLVWILENDI-------------------------------------------TPVLDH

TFCVEHNA-----------------------------FGRILQHELKPNG----RNIPVT

EENK--KEYVRLYVNWRFMRG-----IEAQFLALQKG---------------FNELI---

--PQHLL-KPFDQKELELIIGGLDK--------------------------IDLNDWKSN

TRLKH----------------CMADSNIVKWFWQAVE-TFDEER----RARLLQFVTGST

RVPLQGFKALQGAA----GPRLFTIHLIDAN--------------------------TDN

LP-KAHTCFN-----RIDIPP-YESYEK--LYEKLLTAVEET-CGF

>Xenopus_laevis_NM_001088470.1 .

VEVSREEIFEESYRQIMKMR----------------PKDLK-KRLMVKFR-----GEEGL

---DYGGVAREWLYLLCHEMLNP-------------------------------------

------------------------------------------------------------

------------------------------------------------------------

------------------------------------------------------------

------------------------------------------------------------

--------------------YYGLFQYSTDNI----------------------------

------------YTLQINPDS---------SINPDHLSYFHFVG--RIMGLAVFHG----

-----HYINGGFTV-PFYKQLLGKP-----------------------------------

----------------------------------------IQLSDLE---SVDPELH---

KSLVWILENDI-------------------------------------------TSVLDH

TFCVEHNA-----------------------------FGRLLQHELKPNG----KNLQVT

EENK--KEYVRLYVNWRFMRG-----IEAQFLALQKG---------------FNELI---

--PQHLL-KPFEQKELELIIGGLDK--------------------------IDISDWKAN

TRLKH----------------CLANSNIVQWFWQAVE-SFDEER----RARLLQFVTGST

RVPLQGFKALQGSTGAA-GPRLFTIHLIDAN--------------------------TDN

LP-KAHTCFN-----RIDIPP-YESYEK--LYEKLLTAVEET-SGF

>Danio_rerio_NM_001001943.1 .

IEVSREEIFEESYRQIMKMR----------------PKDLK-KRLMVKFR-----GEEGL

---DYGGVAREWLYLLCHEMLNP-------------------------------------

------------------------------------------------------------

------------------------------------------------------------

------------------------------------------------------------

------------------------------------------------------------

--------------------YYGLFQYSTDNI----------------------------

------------YTLQINPDS---------SINPDHLSYFHFVG--RIMGLAVFHG----

-----HYINGGFTL-PFYKQLLGKP-----------------------------------

----------------------------------------IQLCDLE---TVDPELH---

KSLVWILENDI-------------------------------------------TSVLDH

TFCVEHNA-----------------------------FGKFLQHELKPNG----KNIPVT

EENK--KEYVRLYVNWRFMRG-----IEAQFLALQKG---------------FNELI---

--PQHLL-KPFDNKELELIIGGLGK--------------------------IDLNDWKAN

TRLKH----------------CVADSNIVKWFWQAVE-SFDEER----RGRLLQFVTGST

RVPLQGFKALQGSTGSA-GPRLFTIHLIDAN--------------------------TDN

LP-KAHTCFN-----RIDIPP-YESYEK--LYEKLLTAVEET-CGF

>Xenopus_tropicalis_BC081337.1 .

IEVSREEIFEESYRQVMKMR----------------PKDLW-KRLMIKFR-----GEEGL

---DYGGVAREWLYLLSHEMLNP-------------------------------------

------------------------------------------------------------

------------------------------------------------------------

------------------------------------------------------------

------------------------------------------------------------

--------------------YYGLFQYSRDDI----------------------------

------------YTLQINPDS---------AVNPEHLSYFHFVG--RIMGMAVFHG----

-----HYIDGGFTL-PFYKQLLGKP-----------------------------------

----------------------------------------ITLDDME---SVDPDLH---

NSLVWILENDI-------------------------------------------TGVLDH

TFCVEHSA-----------------------------YGELIQHELKPSG----KSIPVT

EETK--KEYVRLYVNWRFLRG-----IEAQFLALQKG---------------FNEVI---

--PQHLL-KAFDEKELELIICGLGK--------------------------IDVNDWKSN

TRLKH----------------CTADSNIVKWFWKAVE-SFDEER----RARLLQFVTGSS

RVPLQGFKALQGAA----GPRLFTIHQIDAS--------------------------TNN

LP-KAHTCFN-----RIDIPP-YETYEK--LYEKLLTAIEET-CGF

>Xenopus_laevis_NM_001088813.1 .

IEVSREEIFEESYRQVMKMR----------------PKDLW-KRLMIKFR-----GEEGL

---DYGGVAREWLYLLSHDMLNP-------------------------------------

------------------------------------------------------------

------------------------------------------------------------

------------------------------------------------------------

------------------------------------------------------------

--------------------YYGLFQYSRDDI----------------------------

------------YTLQINPDS---------AVNPEHLSYFHFVG--RIMGMAVFHG----

-----HYIDGGFTL-PFYKQLLGKP-----------------------------------

----------------------------------------ITLDDME---SVDPDLH---

NSLVWILENDI-------------------------------------------TGVLDH

TFCVEHNA-----------------------------YGELIQHELKPSG----KSIPVT

EDTK--KEYVRLYVNWRFLRG-----IEAQFLALQKG---------------FNEVI---

--PQHLL-KAFDEKELELIICGLGK--------------------------IDVSDWKSN

TRLKH----------------CTTDSNIVKWFWKAVE-SFDEER----RARLLQFVTGSS

RVPLQGFKALQGAA----GPRLFTIHQIDAS--------------------------TNN

LP-KAHTCFN-----RIDIPP-YETYEK--LYEKLLTAIEET-CGF

>Danio_rerio_NM_001114426.1 .

IEVSREEIFEESYRQVMKMR----------------PKDLW-KRLMVKFR-----GEEGL

---DYGGVAREWLYLLSHEMLNP-------------------------------------

------------------------------------------------------------

------------------------------------------------------------

------------------------------------------------------------

------------------------------------------------------------

--------------------YYGLFQYSRDDI----------------------------

------------YTLQINPDS---------AVNPEHLSYFHFVG--RIMGMAVFHG----

-----HYIDGGFTL-PFYKQLLGKP-----------------------------------

----------------------------------------ITLDDME---SVDPDLH---

NSLVWILDNDI-------------------------------------------TGVLDH

TFCVEHNA-----------------------------YGEIIQHELKPNG----KSIPVT

QDTK--KEYVRLYVNWRFLRG-----IEAQFLALQKG---------------FNEVI---

--PQHLL-KAFDEKELELIVCGLGK--------------------------IDINDWKSN

TRLKH----------------CTPDSNIVKWFWRAVE-SYDEER----RARLLQFVTGSS

RVPLQGFKALQGAA----GPRLFTIHQIDAS--------------------------TNN

LP-KAHTCFN-----RIDIPP-YESYDK--LYDKLLTAIEET-CGF

>Equus_caballus_XM_001917106.1 .

IEVSREEIFEESYRQVMKMR----------------PKDLW-KRLMIKFR-----GEEGL

---DYGGVAREWLYLLSHEMLNP-------------------------------------

------------------------------------------------------------

------------------------------------------------------------

------------------------------------------------------------

------------------------------------------------------------

--------------------YYGLFQYSRDDI----------------------------

------------YTLQINPDS---------AVNPEHLSYFHFVG--RIMGMAVFHG----

-----HYIDGGFTL-PFYKQLLGKS-----------------------------------

----------------------------------------ITLDDME---LVDPDLH---

NSLVWILENDI-------------------------------------------TGVLDH

TFCVEHNA-----------------------------YGEIIQHELKPNG----KSIPVT

EENK--KEYVRLYVNWRFLRG-----IEAQFLALQKG---------------FNEVI---

--PQHLL-KTFDEKELELIICGLGK--------------------------IDVNDWKVN

TRLKH----------------CTPDSNVVKWFWKAVE-FFDEER----RARLLQFVTGSS

RVPLQGFKALQGAA----GPRLFTIHQIDAC--------------------------TNN

LP-KAHTCFN-----RIDIPP-YESYEK--LYEKLLTAIEET-CGF

>Ornithorhynchus_anatinus_XM_001510676.1 .

IEVSREEIFEESYRQVMKMR----------------PKDLW-KRLMIKFR-----GEEGL

---DYGGVAREWLYLLSHEMLNP-------------------------------------

------------------------------------------------------------

------------------------------------------------------------

------------------------------------------------------------

------------------------------------------------------------

--------------------YYGLFQYSRDDI----------------------------

------------YTLQINPDS---------AVNPEHLSYFHFVG--RIMGMAVFHG----

-----HYIDGGFTL-PFYKQLLGKP-----------------------------------

----------------------------------------ITLDDME---LVDPDLH---

NSLVWILENDI-------------------------------------------TGVLDH

TFCVEHNA-----------------------------YGEIIQHELKPNG----KSIPVT

EENK--KEYVRLYVNWRFLRG-----IEAQFLALQKG---------------FNEVI---

--PQHLL-KTFDEKELELIICGLGK--------------------------IDVSDWKVN

TRLKH----------------CTPDSNIVKWFWKAVE-LFDEER----RARLLQFVTGSS

RVPLQGFKALQGAA----GPRLFTIHQIDAS--------------------------TNN

LP-KAHTCFN-----RIDIPP-YESYEK--LYEKLLTAIEET-CGF

>Monodelphis_domestica_XM_001379266.1 .

IEVSREEIFEESYRQVMKMR----------------PKDLW-KRLMIKFR-----GEEGL

---DYGGVAREWLYLLSHEMLNP-------------------------------------

------------------------------------------------------------

------------------------------------------------------------

------------------------------------------------------------

------------------------------------------------------------

--------------------YYGLFQYSRDDI----------------------------

------------YTLQINPDS---------AVNPEHLSYFHFVG--RIMGMAVFHG----

-----HYIDGGFTL-PFYKQLLGKS-----------------------------------

----------------------------------------INLDDME---LVDPDLH---

NSLVWILENDI-------------------------------------------TGVLDH

TFCVEHNA-----------------------------YGEIIQHELKPNG----KSISVT

EDNK--KEYVRLYVNWRFLRG-----IEAQFLALQKG---------------FNEVI---

--PQHLL-KTFDEKELELIICGLGK--------------------------IDVSDWKVN

TRLKH----------------CTPDSNIVKWFWKAVE-FFDEER----RARLLQFVTGSS

RVPLQGFKALQGAA----GPRLFTIHQIDAS--------------------------TNN

LP-KAHTCFN-----RIDIPP-YESYEK--LYEKLLTAIEET-CGF

>Monosiga_brevicollis_XM_001750727.1 .

----------------MRLQ----------------RKDLR-RRLMVQFH-----GEGGL

---DYGGLAREWFYLLGIEVFDP-------------------------------------

------------------------------------------------------------

------------------------------------------------------------

------------------------------------------------------------

------------------------------------------------------------

--------------------RLGMFSYCNEQD----------------------------

------------YLLQINPSS---------SADQDHTLFFHFTG--RLIGLAILHR----

-----HFLDVTFVS-SFYKQILGQS-----------------------------------

----------------------------------------ITLADLQ---DSDPDVH---

RSLIWILENDV-------------------------------------------SEVPDL

TFSTDEDE-----------------------------LGDIRTHELVPGG----ANKAVT

EENK--FEFAKLMVEWKLIKS-----SSRQMCALLTG---------------LNEVI---

--PIENF-RTFTVKELRFLISGSHE--------------------------YDLEDWKRN

TEYKG----------------YESNDQIIEWLWEIVE-AWDHDN----QARFLQFCTGSS

RVPIEGFQALQGSD----GPRRFCIQKLED---------------------------LTR

LP-SAHTCFN-----RLDLPE-FPVRRM--LEERLALALKNA-QGF

>Caenorhabditis_briggsae_XM_001665099.1 .

WKVSRDRLLDDAFRVILSVDP---------------FVLKK-SRLHIRFE-----GELAL

---DYGGLSREFFILLSRELFHP-------------------------------------

------------------------------------------------------------

------------------------------------------------------------

------------------------------------------------------------

------------------------------------------------------------

--------------------NNGYFEYEGND-----------------------------

------------YHLQLRPRG---------CESEKERKWLTLCG--KVLALAIIHR----

-----CYIDVFFSN-IFYKCLQRKP-----------------------------------

----------------------------------------IDLSDFQ---ESDADFY---

KSMNWLLEND--------------------------------------------VDALEM

SFVYSSMV--------------NGKASDFKQ-NDLLVFFQLTEQELLNGG----DKKIVT

NSNK--TEFVELMCQKKSTRG-----IEKPLEIILES---------------FSQIL---

--KADIL-NGMCSSELKRILSGSLE--------------------------LDLNDWRIN

TIYKG---------------GYSDCHIVIEWFWEVIE-TMTNQE----RFDLLLFVTGSS

SVPFEGFSALRGND----EISKFCIEKWGD---------------------------ATS

LP-RAHTCFN-----RLQLPS-YNTKHN--LKSKLQQAISNG-MSY

>Caenorhabditis_elegans_NM_065296.2 .

WKVSRDRLLDDAFRIILNVDP---------------FVLKK-SRLHIRFE-----GELAL

---DYGGLSREFFILLSRELFHP-------------------------------------

------------------------------------------------------------

------------------------------------------------------------

------------------------------------------------------------

------------------------------------------------------------

--------------------KNGYFEYEGND-----------------------------

------------YHLQLRPRG---------CETEKEKKWLILCG--RVLALAVIHR----

-----CYIDVFFTN-VFYKSLQKRP-----------------------------------

----------------------------------------VTLMDFK---ESDAEFY---

KSMNWLLEND--------------------------------------------VVDLEM

SFVYSSMV--------------NGKVSDLTLSSIVSVALQLAEQELVPGG----ESQMVT

EANK--AEFIDLMCQKKAIRG-----VEKPLEILLTS---------------FNQIL---

--NDNLL-NSLESSDLKRILSGSLE--------------------------LDLNDWRTN

TIYKG---------------GYSDCHIVVEWFWEVIE-TMTNQE----RFDLLLFVTGSS

SVPFEGFSALRGNE----EISKFCIEKWGD---------------------------ATS

FP-RAHTCFN-----RLQLPS-YNTKQQ--LKSKLQQAIVNG-MSY

>Trichoplax_adhaerens_XM_002110698.1 .

----------------------------------------K-NKLEIQFV-----GEDGL

---DYGGLTREFFFLLSRELFNP-------------------------------------

------------------------------------------------------------

------------------------------------------------------------

------------------------------------------------------------

------------------------------------------------------------

--------------------VCGLFEYSANDT----------------------------

------------YTIQVSPKS---------TYVDHWQDWFRFCG--RVIGMAIVHR----

-----YLLDVFFAR-PFYKALLERP-----------------------------------

----------------------------------------CNIGDLE---FVDEQYH---

RSITWIMDNEI-------------------------------------------TDDLAV

PFCISYDV-----------------------------FGEKREYELKPNG----RNIYVT

ESDK--KEYVELFLKWRLEQS-----ISEQIKPILKG---------------FRDII---

--GPEML-STFDANELELLICGTHD--------------------------IDVDDWIKN

TEYRS---------------GYSEDHQVIVWFWEAVR-IMSNEK----RLRLLQFITGTS

SLPVEGFAALRGSS----GLRKFCIDKLSS---------------------------TDK

LP-RAHTCFN-----RLDLPA-YPSYKV--LYKKLVLAIEET-DNF

>Nematostella_vectensis_XM_001641396.1 .

----------------------------------------R-NRLEIQFA-----GEEGL

---DYGGPAREFFFLISRQIFNP-------------------------------------

------------------------------------------------------------

------------------------------------------------------------

------------------------------------------------------------

------------------------------------------------------------

--------------------YYGLFEYSANDT----------------------------

------------YTVQVSPVS---------LYVDNSHEWFRFCG--RIVGLVIIHQ----

-----HLLDAFFTR-TFYKALLRSL-----------------------------------

----------------------------------------CDLSDVE---ALDALFH---

QSMTWVIENDI-------------------------------------------EDVLDL

TFSVSEEI-----------------------------FGQVTERELIPGG----KDIAVT

EQNK--GDYVAEMVKWRVERG-----VSEQMESIVRG---------------FNEVI---

--DPALV-NIFDARELELVISGTAD--------------------------IDIKDWRRN

TEYRS---------------GYHDNHKVVKWFWKAVS-SFDNEQ----RLRLLQFVTGTS

SIPYEGFAALRGST----GPRKFSIERWGD---------------------------YTK

LP-RAHTCFN-----RLDMPI-YRSYDE--LLEKLTYAVEET-GSF

>Hydra_magnipapillata_XM_002163250.1 .

LKIRRDHVLQDAFEQVMKLSP---------------RALHK-EKLYIKFT-----GEEGL

---DYGGPAREFFFMISRELFNP-------------------------------------

------------------------------------------------------------

------------------------------------------------------------

------------------------------------------------------------

------------------------------------------------------------

--------------------YYGLFEYSASDT----------------------------

------------YTLQVSPAS---------MYCENAQNWFRFIG--RIISLALIHQ----

-----HLLDVFFTR-TIYKALLREK-----------------------------------

----------------------------------------WDLSDLE---TLDEEYY---

QSLKWMLENDI-------------------------------------------TDILDL

TFSVNEEV-----------------------------FGQVSERELKPNG----KNITVT

EGNK--HEYVDMMVKWKIERG-----MGEQMEQIIKG---------------FTDAL---

--DLKMI-SLFDPKELELVIAGTVD--------------------------IDIEDWRKN

TEYRS---------------GYHDSHPIVEWFWKAVG-SFTNER----RLRLLQFVTGTS

SIPYEGFSALRGSS----GLKKFTIDCWGS---------------------------EEM

LP-RAHTCFN-----RLDLPP-YKSYDK--LFEKLLFAIEES-STF

>Ciona_intestinalis_XM_002121914.1 .

LTVHRDNLLEDAFRKVMLLPR---------------KQLQR-SKLFVSFA-----GEEGL

---DYSGPSREFFFLISRELFNP-------------------------------------

------------------------------------------------------------

------------------------------------------------------------

------------------------------------------------------------

------------------------------------------------------------

--------------------YYGLFEYSAVDT----------------------------

------------YTVQISPLS---------TFADSPHEWFRFAG--RIIGLALVHH----

-----CLLDAFFTR-PLYKMLLRSK-----------------------------------

----------------------------------------CDLSDLR---YEDEQFY---

QSIMWIKDNDI-------------------------------------------TDVLDL

TFSVNEEM-----------------------------FGKIEERELKPNG----KNIAVT

EKNK--KEYIERMVKWRVVRG-----TREQTNMLIRG---------------FNEVI---

--DLRLV-SVFDANELELVICGTAD--------------------------IDLNDWRQH

TEYRG---------------GYYDQHPVVINFWEALD-QFDNER----RLRLLQFVTGTS

SIPYEGFAALRGPN----GPKRFCIEKWGK---------------------------PDC

LP-RTHTCFN-----RLDLPP-YDTFNL--LWEKLVIAIEET-NTF

>Anopheles_gambiae_XM_311340.4 .

LEVRRAHLVEDAFERIMAASR---------------RDLQR-CRLNIVWD-----TEDGL

---DYGGPSREFFYLLSRQLFSP-------------------------------------

------------------------------------------------------------

------------------------------------------------------------

------------------------------------------------------------

------------------------------------------------------------

--------------------YRNMFEYSANDI----------------------------

------------YTVQIAPERP--------TDRDDILEWYRFAG--RVLGLALVHQ----

-----YLLDAFFTR-PFYKALLRLP-----------------------------------

----------------------------------------VSLSDLE---SLDSSFH---

QSLLWIRDNNMDN-----------------------------------------CGELGL

NFTVTEER----------------------------SDGTSIDIELKPNG----RNITVS

ERNK--RDYLDRIIKWRLERG-----VLEQTEWLVRG---------------FNEVV---

--DHRLV-AVFDASELELVISGTVE--------------------------IDVHDWRAN

TEYRG---------------GYHDTHHVIMWFWAVIE-RMSNEQ----RLRLLQFVTGTS

SIPHDGFAGLRGSN----GLRRFCIEKWGK---------------------------ANA

LP-RSHTCFN-----RLDLPP-YPTPDV--LYEKLVLAVEET-NTF

>Drosophila_willistoni_XM_002071000.1 .

LHIRRSHLLEDAFRRIMSANK---------------KDLQR-GRLAVLWD-----TEEGL

---DYGGPSREFFFLLSRELFNP-------------------------------------

------------------------------------------------------------

------------------------------------------------------------

------------------------------------------------------------

------------------------------------------------------------

--------------------YYGLFEYSANDT----------------------------

------------YTVQVSPLS---------AFVDNCHDWFRFSG--RVLGLALVHQ----

-----YLLDAFFTR-PFYKALLRLP-----------------------------------

----------------------------------------VALSDLE---SLDNEFH---

QSLQWIRDNDIGT-----------------------------------------GIDLGL

TFCVTEEL-----------------------------LGRVVERELKPGG----KNMIVN

EKNK--KEYLERMIKWRLERG-----VQEQTESLVRG---------------FYEVV---

--DSRLV-SVFDARELELVIAGTAE--------------------------IDTNDWRLN

TEYRS---------------GYHDNHQVIVWFWQVIE-RFSNEQ----RLRLLQFVTGTS

SIPYEGFSALRGST----GPRRFCIEKWGK---------------------------PNA

LP-RAHTCFN-----RLDLPP-YPTPEL--LYEKLLLAVEET-NTF

>Drosophila_mojavensis_XM_002010268.1 .

LHIRRSHLLEDAFRRIMSANK---------------KDLQR-GRLAVLWD-----TEEGL

---DYGGPSREFFFLLSRELFNP-------------------------------------

------------------------------------------------------------

------------------------------------------------------------

------------------------------------------------------------

------------------------------------------------------------

--------------------YYGLFEYSANDT----------------------------

------------YTVQVSPLS---------AFVDNCHDWFRFSG--RVLGLALVHQ----

-----YLLDAFFTR-PFYKALLRLP-----------------------------------

----------------------------------------VALSDLE---SLDNEFH---

QSLQWIRDNDIGT-----------------------------------------GVDLGL

TFCVTEEL-----------------------------LGRVVERELKPGG----KNLIVN

EKNK--KEYLERMIKWRLERG-----VQEQTESLVRG---------------FYEVV---

--DSRLV-SVFDARELELVIAGTAE--------------------------IDTNDWRLN

TEYRS---------------GYHDNHQVIIWFWQVIE-RFSNEQ----RLRLLQFVTGTS

SIPYEGFSALRGST----GPRRFCIEKWGK---------------------------PNA

LP-RAHTCFN-----RLDLPP-YPTPEL--LYEKLLLAVEET-NTF

>Drosophila_yakuba_AAEU02000954.1 .

LHIRRSHLLEDAFRRIMSANK---------------KDLQR-GRLAVLWD-----TEEGL

---DYGGPSREFFFLLSRELFNP-------------------------------------

------------------------------------------------------------

------------------------------------------------------------

------------------------------------------------------------

------------------------------------------------------------

--------------------YYGLFEYSANDT----------------------------

------------YTVQVSPLS---------AFVDNCHDWFRFSG--RVLGLALVHQ----

-----YLLDAFFTR-PFYKALLRLP-----------------------------------

----------------------------------------VALSDLE---SLDNEFH---

QSLQWIRDNDIGT-----------------------------------------GVDLGL

TFCVTEEL-----------------------------LGRVVERELKPGG----KNIIVN

EKNK--KEYLERMIKWRLERG-----VQEQTESLVRG---------------FYEVV---

--DSRLV-SVFDARELELVIAGTAE--------------------------IDTNDWRLN

TEYRS---------------GYHDNHQVIVWFWQVIE-RFSNEQ----RLRLLQFVTGTS

SIPYEGFSALRGST----GPRRFCIEKWGK---------------------------PNA

LP-RAHTCFN-----RLDLPP-YPTPEL--LYEKLLLAVEET-NTF

>Drosophila_melanogaster_BT058014.1 .

LHIRRSHLLEDAFRRIMSANK---------------KDLQR-GRLAVLWD-----TEEGL

---DYGGPSREFFFLLSRELFNP-------------------------------------

------------------------------------------------------------

------------------------------------------------------------

------------------------------------------------------------

------------------------------------------------------------

--------------------YYGLFEYSANDT----------------------------

------------YTVQVSPLS---------AFVDNCHDWFRFSG--RVLGLALVHQ----

-----YLLDAFFTR-PFYKALLRLP-----------------------------------

----------------------------------------VALSDLE---SLDNEFH---

QSLQWIRDNDIGT-----------------------------------------GVDLGL

TFCVTEEL-----------------------------LGSVVDRELKPGG----KNIIIN

EKNK--KEYLERMIKWRLERG-----VQEQTESLVRG---------------FYEVI---

--DSRLV-SVFDARELELVIAGTAE--------------------------IDTNDWRLN

TEYRS---------------GYHDNHQVIVWFWQVIE-RFSNEQ----RLRLLQFVTGTS

SIPYEGFSALRGST----GPRRFCIEKWGK---------------------------PNA

LP-RAHTCFN-----RLDLPP-YPTPEL--LYEKLLLAVEET-NTF

>Drosophila_grimshawi_XM_001990948.1 .

LHIRRSHLLEDAFRRIMSANK---------------KDLQR-GRLAVLWD-----TEEGL

---DYGGPSREFFFLLSRELFNP-------------------------------------

------------------------------------------------------------

------------------------------------------------------------

------------------------------------------------------------

------------------------------------------------------------

--------------------YYGLFEYSANDT----------------------------

------------YTVQVSPLS---------AFVDNCHDWFRFSG--RVLGLALVHQ----

-----YLLDAFFTR-PFYKALLRLP-----------------------------------

----------------------------------------VALSDLE---SLDNEFH---

QSLQWIRDNDIGT-----------------------------------------GIDLGL

TFCVTEEL-----------------------------LGRVVERELKPGG----RNVIVH

EKNK--KEYLERMIKWRLERG-----VQEQTESLVRG---------------FYEVV---

--DSRLV-SVFDARELELVIAGTAE--------------------------IDTNDWRLN

TEYRS---------------GYHDNHQVIIWFWQVIE-RFSNEQ----RLRLLQFVTGTS

SIPYEGFSALRGST----GPRRFCIEKWGK---------------------------PNS

LP-RAHTCFN-----RLDLPP-YPTPEL--LYEKLLLAVEET-NTF

>Aedes_aegypti_XM_001651397.1 .

LHIRRSHLLEDAFRRIMSANK---------------KDLQR-GRLAVLWD-----TEEGL

---DYGGPSREFFFLLSRELFNP-------------------------------------

------------------------------------------------------------

------------------------------------------------------------

------------------------------------------------------------

------------------------------------------------------------

--------------------YYGLFEYSANDT----------------------------

------------YTVQVSPLS---------AFVDNSHDWFRFSG--RVLGLALVHQ----

-----YLLDAFFTR-PFYKALLRLP-----------------------------------

----------------------------------------VALSDLE---SLDNEFH---

QSLQWIRDNDIGS-----------------------------------------GTALGL

TFCVTEEL-----------------------------LGRVVERELKPGG----KNIPVT

EKNK--REYLERMVKWRLERG-----VQEQTESLVRG---------------FYEVV---

--DPRLV-SVFDARELELVIAGTAE--------------------------IDLNDWRIN

TEYRS---------------GYHDGHQVIVWFWHVIE-KFSNEQ----RLRLLQFVTGTS

SIPYEGFAALRGST----GPRRFCIEKWGK---------------------------PNA

LP-RAHTCFN-----RLDLPP-YPTPDI--LYEKLLLAVEET-NTF

>Culex_quinquefasciatus_XM_001868164.1 .

LHIRRSHLLEDAFRRIMSANK---------------KDLQR-GRLAVLWD-----TEEGL

---DYGGPSREFFFLLSRELFNP-------------------------------------

------------------------------------------------------------

------------------------------------------------------------

------------------------------------------------------------

------------------------------------------------------------

--------------------YYGLFEYSANDT----------------------------

------------YTVQVSPLS---------AFVDNSHDWFRFSG--RVLGLALVHQ----

-----YLLDAFFTR-PFYKALLRLP-----------------------------------

----------------------------------------VALSDLE---SLDNEFH---

QSLQWIRDNDIGS-----------------------------------------GVSLGL

TFCVTEEL-----------------------------LGRVVERELKPGG----KNIPVT

EKNK--REYLERMVKWRLERG-----VQEQTESLVRG---------------FYEVV---

--DPRLV-SVFDARELELVIAGTAE--------------------------IDLNDWRIN

TEYRS---------------GYHDGHQVIVWFWHVIE-KFSNEQ----RLRLLQFVTGTS

SIPYEGFAALRGST----GPRRFCIEKWGK---------------------------PNA

LP-RAHTCFN-----RLDLPP-YPTPDI--LYEKLLLAVEET-NTF

>Tribolium_castaneum_XM_968154.1 .

LHIRREHLLEDAFRRIMSANK---------------KELQK-GKLCVVWD-----NEEGL

---DYGGPSREFFFLLSRELFNP-------------------------------------

------------------------------------------------------------

------------------------------------------------------------

------------------------------------------------------------

------------------------------------------------------------

--------------------YYGLFEYSANDT----------------------------

------------YTVQISPMS---------AFVDNYHDWFRFSG--RVLGLALVHQ----

-----YLLDAFFTR-PFYKALLRLP-----------------------------------

----------------------------------------VALSDLE---SLDFEFH---

QSLQWIREHDVSM-----------------------------------------QGELEL

TFAVTEEV-----------------------------FGQVLERELKPGG----RNVPVT

EKNK--KEYLERIVRWRLERG-----VSEQTESLVRG---------------FYEVV---

--DPRLV-SVFDARELELVIAGTAE--------------------------IDLADWRQN

TEYRG---------------GYHDQHPVVVWFWQAIE-RFSNEQ----RLRLLQFVTGTS

SIPFEGFSALRGSI----GPRKFCIEKWGK---------------------------PNS

LP-RAHTCFN-----RLDLPP-YPTSEV--LYEKLLLAVEET-NTF

>Apis_mellifera_XM_392900.3 .

LHIRREHFLEDAFTRIMAASK---------------KDLQK-SKLVIMFD-----EEEGV

---DYGGPSREFFFHLSRELFNP-------------------------------------

------------------------------------------------------------

------------------------------------------------------------

------------------------------------------------------------

------------------------------------------------------------

--------------------YYGLFEYSANDT----------------------------

------------YTVQVSPMS---------AFVDNYHDWFRFSG--RVLGLALVHQ----

-----YLLDAFFTR-PFYKALLRIP-----------------------------------

----------------------------------------ASLSDLE---SLDQEFH---

QSLMWIKERDIS------------------------------------------IEPLEL

TFSVTEEL-----------------------------LGRVAERELKPGG----RNIAVT

EKNK--KEYLERVVRWRLERG-----IAEQTESLVRG---------------FYEVV---

--DPRLV-SVFDARELELVIAGAAE--------------------------IDLNDWRTH

TEYRS---------------GYHDAHPVVEWFWSSIS-RFTNEQ----RLRLLQFVTGTS

SIPYEGFAALRGST----GPRKFCIEKWGR---------------------------PNS

LP-RAHTCFN-----RLDLPP-YPTPEI--LYEKLLLAVEET-NTF

>Nasonia_vitripennis_XM_001606781.1 .

LHIRREHLLEDAFTHIMAVSK---------------KDLQK-GKLVVIFD-----REEGL

---DYGGPSREFFFHLSRELFNP-------------------------------------

------------------------------------------------------------

------------------------------------------------------------

------------------------------------------------------------

------------------------------------------------------------

--------------------YYGLFEYSANDT----------------------------

------------YTVQVSPMS---------AFVDNYHDWFRFSG--RVLGLALVHQ----

-----YLLDVFFTR-PFYKALLRIP-----------------------------------

----------------------------------------ASLSDLE---SLDQEFH---

QSLMWIKEKDIT------------------------------------------IEPLEL

TFSVTEEL-----------------------------LGRVAERELKPGG----RNIPVT

EKNK--KEYLERILRWRLERG-----VAEQTESLVRG---------------FYEVV---

--DPRLV-SVFDARELELVIAGAAE--------------------------IDLNDWRAH

TEYRS---------------GYHDAHPVIEWFWSSIS-RFSNEQ----RLRLLQFVTGTS

SIPYEGFAALRGST----GPRKFCIEKWGR---------------------------PNS

LP-RAHTCFN-----RLDLPP-YPTPEI--LYEKLLLAVEET-NTF

>Acyrthosiphon_pisum_XM_001944719.1 .

IDVRRDHLLQDAYNKIMSMPK---------------KDLQK-CKLSAQFD-----NEEGL

---DYGGPSREFFFLISRELFNP-------------------------------------

------------------------------------------------------------

------------------------------------------------------------

------------------------------------------------------------

------------------------------------------------------------

--------------------YYGLFEYSANDT----------------------------

------------YTVQISPMSA--------AFVNNKEDWFRFSG--RVLGLALVHQ----

-----YLLDAFFTR-PFYKTLLKLP-----------------------------------

----------------------------------------MSLSDLE---SVDREFH---

QSLCWVQERDIS------------------------------------------GEMLDL

TFSVTEEV-----------------------------FGQTVEKELKPGG----QRIFVT

DKNK--KEYIDKIVKWRLERG-----VTEQTGALVQG---------------FYDVV---

--DPRLV-SVFDAQELELVIAGTVE--------------------------IDLSDWRNN

TEYRS---------------GYHDGHQVIRWFWSSIE-QFTNEQ----RLRLLQFVTGTS

SIPYEGFSALRGST----SPRKFCIEKWGK---------------------------ANS

LP-RAHTCFN-----RLDLPP-YTTQNI--LCEKLLLAIDES-NTF

>Branchiostoma_floridae_XM_002243269.1 .

LNIRRDHLLSDTFNKIMGTTR---------------RDLQR-NKLYVTFV-----GEEGL

---DYGGPSREFFFLLSRELFNP-------------------------------------

------------------------------------------------------------

------------------------------------------------------------

------------------------------------------------------------

------------------------------------------------------------

--------------------YYGLFEYSANDQ----------------------------

------------YTVQVSPMS---------AFVDNAHDWFRFSG--RILGLALIHQ----

-----YLLDAFFTR-PFYKALLRTP-----------------------------------

----------------------------------------CDINDLQ---AVDEEFY---

ASLQWIKDNDI-------------------------------------------TDILEL

TFSVDEEV-----------------------------FGQVTERELITNG----KNVPVT

EKNK--MNYIERVVKWRLERG-----VAEQTESLVRG---------------FYEVI---

--DTRLV-SVFDARELELVIAGTAE--------------------------IDIVDWRKN

TEYRS---------------GYHDRHPVIQWFWTAVE-RFDNER----RLRLLQFVTGTS

SIPYEGFAALRGSN----GPRKFCIEKWGK---------------------------ISA

LP-RAHTCFN-----RLDLPP-YPSYAM--LYEKLIIAVEET-STF

>Rana_catesbeiana_GO472865.1 .

-----------AFNQIMGYSR---------------KDLQR-NKLYVTFV-----GEEGL

---DYSGPSREFFFLVSRELFNP-------------------------------------

------------------------------------------------------------

------------------------------------------------------------

------------------------------------------------------------

------------------------------------------------------------

--------------------YYGLFEYSANDT----------------------------

------------YTVQISPMS---------AFVDNHHEWFRFSG--RILGLALIHQ----

-----YLLDAFFTR-PFYKALLRIL-----------------------------------

----------------------------------------CDLSDLE---YLDEEFH---

QSLQWMKDNDI-------------------------------------------HDMLDL

TFMVNEEV-----------------------------FGQITERELKPGG----ANIPVT

EKNK--KEYIEKMVKWRIERG-----VVQQTESLVRG---------------FYEVV---

--DARLV-SVFDARELELVIAGTAE--------------------------IDLGDWRNN

TEYRG---------------GYHDNHIVIRWFWAAVE-RFNNEQ----RLRLLQFVTGTS

SIPYEGFASLRGSN----GPRRFCVEKWGK---------------------------SQ-

----------------------------------------------

>Mus_musculus_NM_001001883.3 .

LIIRRDHLLEDAFNQIMGYSR---------------KDLQR-NKLYVTFV-----GEEGL

---DYSGPSREFFFLVSRELFNP-------------------------------------

------------------------------------------------------------

------------------------------------------------------------

------------------------------------------------------------

------------------------------------------------------------

--------------------YYGLFEYSANDT----------------------------

------------YTVQISPMS---------AFVDNHHEWFRFSG--RILGLALIHQ----

-----YLLDAFFTR-PFYKALLRIL-----------------------------------

----------------------------------------CDLSDLE---YLDEEFH---

QSLQWMKDNDI-------------------------------------------HDILDL

TFTVNEEV-----------------------------FGQITERELKPGG----ANIPVT

EKNK--KEYIERMVKWRIERG-----VVQQTESLVRG---------------FYEVV---

--DARLV-SVFDARELELVIAGTAE--------------------------IDLNDWRNN

TEYRG---------------GYHDNHIVIRWFWAAVE-RFNNEQ----RLRLLQFVTGTS

SIPYEGFASLRGSN----GPRRFCVEKWGK---------------------------ITA

LP-RAHTCFN-----RLDLPP-YPSFSM--LYEKLLTAVEET-STF

>Macaca_mulatta_XR_010263.1 .

------------------------------------------------FV-----GEEGL

---DYSGPSREFFFLVSRELFNP-------------------------------------

------------------------------------------------------------

------------------------------------------------------------

------------------------------------------------------------

------------------------------------------------------------

--------------------YYGLFEYSANDT----------------------------

------------YTVQISPMS---------AFVDNHHEWFRFSG--RILGLALIHQ----

-----YLLDAFFTR-PFYKALLRIL-----------------------------------

----------------------------------------CDLSDLE---YLDEEFH---

QSLQWMKDNDI-------------------------------------------HDILDL

TFTVNEEV-----------------------------FGQITERELKPGG----ANIPVT

EKNK--KEYIERMVKWRIERG-----VVQQTESLVRG---------------FYEVV---

--DARLV-SVFDARELELVIAGTAE--------------------------IDLSDWRNN

TEYRG---------------GYHDNHIVIRWFWAAVE-RFNNEQ----RLRLLQFVTGTS

SIPYEGFASLRGSN----GPRRFCVEKWGK---------------------------ITA

LPSLSHTCLN-----SIDLPP-YPSFSM--LYEKLLTAVEET-STF

>Danio_rerio_XM_001922463.1 .

LIIRRDHLLEDAFNQIMCYSR---------------KDLQR-SKLYVSFV-----GEEGL

---DYSGPSREFFFLVSRELFNP-------------------------------------

------------------------------------------------------------

------------------------------------------------------------

------------------------------------------------------------

------------------------------------------------------------

--------------------YYGLFEYSANDT----------------------------

------------YTVQISPMS---------AFVDNHHEWFRFSG--RILGLALIHQ----

-----YLLDAFFTR-PFYKGLLRIP-----------------------------------

----------------------------------------CDLSDLE---YLDEEFH---

QSLQWMKDNDI-------------------------------------------EDMLDL

TFTVNEEV-----------------------------FGQITERELKPGG----ANIPVS

EKNK--KEYIERMVKWRIERG-----VVQQTESLVRG---------------FYEVV---

--DARLV-SVFDARELELVIAGTAE--------------------------IDLGDWRNN

TEYRG---------------GYHDNHIVIRWFWAAVE-RFNNEQ----RLRLLQFVTGTS

SIPYEGFASLRGSN----GPRRFCVEKWGK---------------------------ITS

LP-RAHTCFN-----RLDLPP-YPSFSM--LYEKMLTAVEET-STF

>Danio_rerio_XM_001922405.1 .

LIIRRDHLLEDAFNQIMCYSR---------------KDLQR-SRLYVSFV-----GEEGL

---DYSGPSREFFFLVSRELFNP-------------------------------------

------------------------------------------------------------

------------------------------------------------------------

------------------------------------------------------------

------------------------------------------------------------

--------------------YYGLFEYSANDT----------------------------

------------YTVQISPMS---------AFVDNHHEWFRFSG--RILGLALIHQ----

-----YLLDAFFTR-PFYKGLLRIP-----------------------------------

----------------------------------------CDLSDLE---FLDEEFH---

QSLQWMKDNDI-------------------------------------------EDMLDL

TFTVNEEV-----------------------------FGQITERELKPGG----SGIAVS

DKNK--KEYIERMVKWRIERG-----VAQQTESLVRG---------------FYEVV---

--DVRLV-SVFDARELELVIAGTAE--------------------------IDLSDWRNN

TEYRG---------------GYHDNHIVIRWFWAAVE-RFNNEQ----RLRLLQFVTGTS

SIPYEGFASLRGSN----GPRRFCVEKWGK---------------------------VTS

LP-RAHTCFN-----RLDLPP-YPSFSM--LYEKMVTAVEET-STF

>Danio_rerio_XM_001920639.1 .

LIVRREHLLEGTFNQVMAYSR---------------KELQR-NKLYITFV-----GEEGL

---DYSGPSREFFFLLSQELFNP-------------------------------------

------------------------------------------------------------

------------------------------------------------------------

------------------------------------------------------------

------------------------------------------------------------

--------------------YYGLFEYSANDT----------------------------

------------YTVQISPMS---------AFVENHLEWFRFSG--RILGLALIHQ----

-----YLLDAFFTR-PFYKALLRL------------------------------------

------------------------------------------------------------

------------------------------------------------------------

----------------------------------------VTERELKSGG----SNIQVT

EKNK--KDYIERMARWRVERG-----VMQQAEALVRG---------------FYEVV---

--DSRLV-SVFDARELELVIAGTAE--------------------------IDLSDWRSN

TEYRG---------------GYHDGHMVMRWFWAAVE-RFNNEQ----RLRLLQFVTGTS

SVPYEGFAALRGSN----GLRRFCIEKWGK---------------------------VTS

LP-RAHTCFN-----RLDLPP-YPSYTM--LYEKLLTAVEET-STF

>Danio_rerio_NM_001145764.1 .

LIVRRDHLLEGTFNQVMAYSR---------------KELQR-NKLYITFV-----GEEGL

---DYSGPSREFFFLLSQELFNP-------------------------------------

------------------------------------------------------------

------------------------------------------------------------

------------------------------------------------------------

------------------------------------------------------------

--------------------YYGLFEYSANDT----------------------------

------------YTVQISPMS---------AFVENHLEWFRFSG--RILGLALIHQ----

-----YLLDAFFTR-PFYKALLRLP-----------------------------------

----------------------------------------TDLSDLE---YLDEEFH---

QSLQWMKENDI-------------------------------------------TDVLDL

TFTVNEEV-----------------------------FGQVTERELKSGG----TNVQVT

EKNK--KEYIERMVKWRVERG-----VVQQTQALVRG---------------FYEVV---

--DSRLV-SVFDARELELVIAGTAE--------------------------IDLNDWRNN

TEYRG---------------GYHDGHIVIRWFWGAVE-RFNNEQ----RLRLLQFVTGTS

SVPYEGFTALRGSN----GLRRFCIEKWGK---------------------------ITS

LP-RAHTCFN-----RLDLPP-YPSYTM--LYEKLLIAVEET-STF

>Xenopus_tropicalis_NM_001130278.1 .

LIIRRDHLLEGTFNQVMAYSR---------------KELQR-NKLYITFV-----GEEGL

---DYSGPSREFFFLLSQELFNP-------------------------------------

------------------------------------------------------------

------------------------------------------------------------

------------------------------------------------------------

------------------------------------------------------------

--------------------YYGLFEYSANDT----------------------------

------------YTVQISPMS---------AFVENHLEWFRFSG--RILGLALIHQ----

-----YLLDAFFTR-PFYKALLRLP-----------------------------------

----------------------------------------CDLSDLE---YLDEEFH---

QSLQWMKDNDI-------------------------------------------TDILDL

TFTVNEEV-----------------------------FGQVTERELKSGG----ANIQVS

EKNK--KEYIEKMVKWRVERG-----VVQQTEALVRG---------------FYEVV---

--DSRLV-SVFDARELELVIAGTAE--------------------------IDLNDWRNN

TEYRG---------------GYHDGHIVIRWFWAAVE-RFNNEQ----RLRLLQFVTGTS

SVPYEGFAALRGSN----GLRRFCIEKWGK---------------------------ITS

LP-RAHTCFN-----RLDLPP-YPSYSM--LHEKLLIAVEET-STF

>Taeniopygia_guttata_XM_002196572.1 .

LIIRRDHLLEGTFNQVMAYSR---------------KELQR-NKLYVTFV-----GEEGL

---DYSGPSREFFFLLSQELFNP-------------------------------------

------------------------------------------------------------

------------------------------------------------------------

------------------------------------------------------------

------------------------------------------------------------

--------------------YYGLFEYSANDT----------------------------

------------YTVQISPMS---------AFVENHLEWFRFSG--RILGLALIHQ----

-----YLLDAFFTR-PFYKALLRLP-----------------------------------

----------------------------------------CDLSDLE---YLDEEFH---

QSLQWMKDNNI-------------------------------------------TDILDL

TFTVNEEV-----------------------------FGQVTERELKSGG----ANTAVT

EKNK--KEYIERMVKWRVERG-----VVQQTEALVRG---------------FYEVV---

--DSRLV-SVFDARELELVIAGTAE--------------------------IDLNDWRNN

TEYRG---------------GYHDGHIVIRWFWAAVE-RFNNEQ----RLRLLQFVTGTS

SVPYEGFAALRGSN----GLRRFCIEKWGK---------------------------ITS

LP-RAHTCFN-----RLDLPP-YPSYSM--LYEKLLTAVEET-STF

>Equus_caballus_XM_001915708.1 .

LIIRRDHLLEGTFNQVMAYSR---------------KELQR-SKLYITFV-----GEEGL

---DYSGPSREFFFLLSQELFNP-------------------------------------

------------------------------------------------------------

------------------------------------------------------------

------------------------------------------------------------

------------------------------------------------------------

--------------------YYGLFEYSANDT----------------------------

------------YTVQISPMS---------AFVENHLEWFRFSG--RILGLALIHQ----

-----YLLDAFFTR-PFYKALLRLP-----------------------------------

----------------------------------------CDLSDLE---YLDEEFH---

QSLQWMKDNNI-------------------------------------------TDILDL

TFTVNEEV-----------------------------FGQVTERELKSGG----ANTQVT

EKNK--KEYIERMVKWRVERG-----VVQQTEALVRG---------------FYEVV---

--DSRLV-SVFDARELELVIAGTAE--------------------------IDLNDWRNH

TEYRG---------------GYHDGHLVIRWFWAAVE-RFNNEQ----RLRLLQFVTGTS

SVPYEGFAALRGSN----GLRRFCIEKWGK---------------------------ITS

LP-RAHTCFN-----RLDLPP-YPSYSM--LYEKLLTAVEET-STF

>Rattus_norvegicus_NM_001106117.1 .

LIIRRDHLLEGTFNQVMAYSR---------------KELQR-NKLYITFV-----GEEGL

---DYSGPSREFFFLLSQELFNP-------------------------------------

------------------------------------------------------------

------------------------------------------------------------

------------------------------------------------------------

------------------------------------------------------------

--------------------YYGLFEYSANDT----------------------------

------------YTVQISPMS---------AFVENYLEWFRFSG--RILGLALIHQ----

-----YLLDAFFTR-PFYKGLLKLP-----------------------------------

----------------------------------------CDLSDLE---YLDEEFH---

QSLQWMKDNNI-------------------------------------------TDILDL

TFTVNEEV-----------------------------FGQVTERELKSGG----ANTQVT

EKNK--KEYIERMVKWRVERG-----VVQQTEALVRG---------------FYEVV---

--DSRLV-SVFDARELELVIAGTAE--------------------------IDLNDWRNN

TEYRG---------------GYHDGHLVIRWFWAAVE-RFNNEQ----RLRLLQFVTGTS

SVPYEGFAALRGSN----GLRRFCIEKWGK---------------------------ITS

LP-RAHTCFN-----RLDLPP-YPSYST--LYEKLLTAVEET-STF

>Mus_musculus_AB083710.1 .

LIIRRDHLLEGTFNQVMAYSR---------------KELQR-NKLYITFV-----GEEGL

---DYSGPSREFFFLLSQELFNP-------------------------------------

------------------------------------------------------------

------------------------------------------------------------

------------------------------------------------------------

------------------------------------------------------------

--------------------YYGLFEYSANDT----------------------------

------------YTVQISPMS---------AFVENYLEWFRFSG--RILGLALIHQ----

-----YLPDAFFTR-PFYKGLLKLP-----------------------------------

----------------------------------------CDLSDLE---YLDEEFH---

QSLQWMKDNNI-------------------------------------------TDILDL

TFTVNEEV-----------------------------FGQVTERELKSGG----ANTQVT

EKNK--KEYIERMVKWRVERG-----VVQQTEALLRG---------------FYEVV---

--DSRLV-SVFDARELELVIAGTAE--------------------------IDLNDWRNN

TEYRG---------------GYHDGHLVIRWFWAAVE-RFNNEQ----RLRLLQFVTGTS

SMPYEGFAALRGSN----GLRRFCIEKWGK---------------------------ITS

LP-RAHTCFN-----RLDLPP-YPSYSM--LYEKLLTAVEET-STF

>Bos_taurus_XM_605958.4 .

LLVHRDSIFRSSCEVVSKAN----------------CAKLK-QGIAVRFH-----GEEGM

----GQGVVREWFDILSNEIVNP-------------------------------------

------------------------------------------------------------

------------------------------------------------------------

------------------------------------------------------------

------------------------------------------------------------

--------------------DYALFTQSADG-----------------------------

------------TTFQPNSNS---------SVNPDHLNYFRFAG--QILGLALNHR----

-----QLVNIYFTR-SFYKHILGIP-----------------------------------

----------------------------------------VNYQDVA---SIDPEYA---

KNLQWILDNDIS------------------------------------------DLGLEL

TFSVETDV-----------------------------FGAMEEVPLKPGG----GSILVT

QNNK--AEYVQLVTELRMTRA-----IQPQINAFLQG---------------FHMFI---

--PPSLI-QLFDEYELELLLSGMPE--------------------------IDVSDWIKN

TEYTS---------------GYEREDPVIQWFWEVVE-DITPEE----RVLLLQFVTGSS

RVPHGGFANIMGGS----GLQNFTIAAVPYT--------------------------PNL

LP-TSSTCIN-----MLKLPE-YPSKEI--LKDRLLVAL-------

>Canis_familiaris_XM_863239.1 .

LLVHRDSIFRSSCEVVSKAN----------------CAKLK-QGIAVRFH-----GEEGM

----GQGVVREWFDILSNEIVNP-------------------------------------

------------------------------------------------------------

------------------------------------------------------------

------------------------------------------------------------

------------------------------------------------------------

--------------------DYALFTQSADG-----------------------------

------------TTFQPNSNS---------YVNPDHLNYFRFAG--QILGLALNHR----

-----QLVNIYFTR-SFYKHILGIP-----------------------------------

----------------------------------------VNYQDVA---SIDPEYA---

KNLQWILDNDIS------------------------------------------DLGLEL

TFSVETDV-----------------------------FGAMEEVPLKPGG----GSILVT

QNNK--------------------------------------------------------

----------------ELLLSGMPE--------------------------IDVSDWIRN

TEYTS---------------GYEREDPVIQWFWEVVE-GITPEE----RVLLLQFVTGSS

RVPHGGFANIMGGS----GLQNFTIAAVPYT--------------------------PNL

LP-TSSTCIN-----MLKLPE-YPSKEI--LKDRLLVAL-------

>Rattus_norvegicus_NM_001108539.3 .

LLVHRDSIFRSSCEIVSKAN----------------CAKLK-QGIAVRFH-----GEEGM

----GQGVVREWFDILSNEIVNP-------------------------------------

------------------------------------------------------------

------------------------------------------------------------

------------------------------------------------------------

------------------------------------------------------------

--------------------DYALFTQSADG-----------------------------

------------TTFQPNSNS---------YVNPDHLNYFRFAG--QILGLALNHR----

-----QLVNIYFTR-SFYKHILGIP-----------------------------------

----------------------------------------VNYQDVA---SIDPEYA---

KNLQWILDNDIS------------------------------------------DLGLEL

TFSVETDV-----------------------------FGAMEEVPLKPGG----GSILVT

QNNK--AEYVQLVTELRMTRA-----IQPQINAFLQG---------------FHMFI---

--PPSLI-QLFDEYELELLLSGMPE--------------------------IDVNDWIKN

TEYTS---------------GYEREDPVIQWFWEVVE-DMTQEE----RVLLLQFVTGSS

RVPHGGFANIMGGS----GLQNFTIAAVPYT--------------------------PNL

LP-TSSTCIN-----MLKLPE-YPSKEI--LKDRLLVAL-------

>Monodelphis_domestica_XM_001368006.1 .

LLVHRDSIFRSSCEVVSKAN----------------CAKLK-QGIAVRFH-----GEEGM

----GQGVVREWFDILSNEIVNP-------------------------------------

------------------------------------------------------------

------------------------------------------------------------

------------------------------------------------------------

------------------------------------------------------------

--------------------DYALFTQSADG-----------------------------

------------TTFQPNSNS---------YVNPDHLNYFRFAG--QILGLALNHR----

-----QLVNIYFTR-SFYKHILGIP-----------------------------------

----------------------------------------VNYQDVA---SIDPEYA---

KNLQWILDNDIS------------------------------------------DLGLEL

TFSVETDV-----------------------------FGAMEEVPLKPGG----ASILVT

QDNK--AEYVQLVTELRMTRA-----IQPQINAFLQG---------------FHMFI---

--PPSLI-QLFDEYELELLLSGMPE--------------------------IDVSDWIKN

TEYTS---------------GYERDDPVVQWFWEVVE-SITQEE----RVLLLQFVTGSS

RVPHGGFANIMGGS----GLQNFTIAAVPYT--------------------------PNL

LP-TSSTCIN-----MLKLPE-YPSKEI--LKDRLLVAL-------

>Ornithorhynchus_anatinus_XM_001505995.1 .

LLVHRDSIFRSSCEVVSKAS----------------CAKLK-QGIAVRFH-----GEEGM

----GQGVVREWFDILSNEIVNP-------------------------------------

------------------------------------------------------------

------------------------------------------------------------

------------------------------------------------------------

------------------------------------------------------------

--------------------DYALFTQSADG-----------------------------

------------TTFQPNSNS---------YVNPDHLNYFRFAG--QILGLALNHR----

-----QLVNIYFTR-SFYKHILGIP-----------------------------------

----------------------------------------VSYQDVA---SIDPEYA---

KNLQWILDNDIS------------------------------------------DLGLEL

TFSVETDV-----------------------------FGAMEEVPLKPGG----ASILVT

QDNK--AEYVQLVTELRMTRA-----IQPQINAFLQG---------------FHMFI---

--PPSLI-QLFDEYELELLLSGLPE--------------------------IDVNDWIKN

TEYTS---------------GYEREDPVIQWFWEVVE-DITQEE----RVLLLQFVTGSS

RVPHGGFANIMGGS----GLQNFTIAAVPYT--------------------------PNL

LP-TSSTCIN-----MLKLPE-YPSKEI--LKDRLLVAL-------

>Taeniopygia_guttata_XM_002196401.1 .

LLVHRDSIFRSSCEVVSKAN----------------CAKLK-QGIAVRFH-----GEEGM

----GQGVVREWFDILSSEIVNP-------------------------------------

------------------------------------------------------------

------------------------------------------------------------

------------------------------------------------------------

------------------------------------------------------------

--------------------DYALFTQSADG-----------------------------

------------TTFQPNSNS---------SVNPDHLNYFRFAG--QILGLALNHR----

-----QLVNIYFTR-SFYKHILGIP-----------------------------------

----------------------------------------VNYQDVA---SIDPEYA---

KNLQWILDNDIS------------------------------------------DLGLEL

TFSVETDV-----------------------------FGAMEEVPLKPGG----ASILVT

QENK--AEYVQLVTELRMTRA-----IQPQINAFLQG---------------FHMFI---

--PPSLI-QLFDEYELELLLSGMPE--------------------------IDVNDWLKN

TEYTS---------------GYERGDQVIQWFWDVVE-ELTQEE----RVLLLQFVTGSS

RVPHGGFAHIMGGS----GLQNFTIAAVPYT--------------------------ANL

LP-TSSTCIN-----MLKLPE-YPSKEI--LKDRLLVAL-------

>Pan_troglodytes_DQ033965.1 .

------------------------------------------------------------

------GVVREWFDILSNEIVNP-------------------------------------

------------------------------------------------------------

------------------------------------------------------------

------------------------------------------------------------

------------------------------------------------------------

--------------------DYALFTQSADG-----------------------------

------------TTFQPNSNS---------YVNPDHLNYFRFAG--QILGLALNHR----

-----QLVNIYFTR-SFYKHILGIP-----------------------------------

----------------------------------------VNYQDVA---SIDPEYG---

KNLQWILDNDIS------------------------------------------DLGLEL

TFSVETDV-----------------------------FGAMEEVPLKPGG----GSILVT

QNNK--AEYVQLVTELRMTRA-----IQPQINAFLQG---------------FHMFI---

--PPSLI-QLFDEYELXLLLXGMPE--------------------------IDVSDWIKN

TEYTS---------------GYEREDPVIQWFWEVVE-DITQEE----RVLLLQFVTGSS

RVPHGGFANIMGGS----GLQNFTIAAVPYT--------------------------PNL

LP-TSSTCIN-----MLKLPE-YPSKEI--LKDRLLVAL-------

>Xenopus_tropicalis_NM_001045804.1 .

---------------------------------------LK-QGIAVRFH-----GEEGM

----GQGVVREWFDILSSEIINP-------------------------------------

------------------------------------------------------------

------------------------------------------------------------

------------------------------------------------------------

------------------------------------------------------------

--------------------DYALFTQSADG-----------------------------

------------TTFQPNSNS---------SVNPDHLNYFRFAG--EILGLALYHR----

-----QLVNIYFTR-SFYKHILGIP-----------------------------------

----------------------------------------VNYQDVA---SIDPEYA---

KNLQWILDNDIS------------------------------------------DLGLEL

TFSVETDV-----------------------------FGAMEEVPLKPGG----ASILVT

QENK--AEYVQLVTELRMTRA-----IQPQINGFLQG---------------FHMFI---

--PPSLI-QLFDEYELELLLSGMPE--------------------------IDVNDWMKN

TEYTS---------------GYERDDQVIQWFWEVVQ-ELTQEE----RVLLLQFVTGSS

RVPHGGFAYIMGGS----GLQNFTIAAVAYT--------------------------PNL

LP-TSSTCIN-----MLKLPE-YPSKEI--LKDRLLVAL-------

>Danio_rerio_XM_696143.3 .

---------------------------------------LK-QGIAVRFH-----GEEGM

----GQGVVREWFDILSNEIINP-------------------------------------

------------------------------------------------------------

------------------------------------------------------------

------------------------------------------------------------

------------------------------------------------------------

--------------------DYALFTQSADG-----------------------------

------------TTFQPNSNS---------SVNPDHLNYFRFAG--QILGLALYHR----

-----QLVNIYFTR-SFYKHILGIP-----------------------------------

----------------------------------------VSYQDVS---SIDPEYA---

KNLQWILDNDIS------------------------------------------DLGLEL

TFSVETDV-----------------------------FGTMEEVPLKPGG----TTIQVT

QDNK--EEYVQLVTELRMTRA-----IQPQINAFLQG---------------FHTFI---

--PPSLI-QLFDEYELELLLSGMPE--------------------------IDVMDWKRN

TEYTS---------------GYDLQEPVIQWFWEVVE-NLTQEE----RVLLLQFVTGSS

RVPHGGFAFLMGGS----GLQKFTVAAVPYT--------------------------SNL

LP-TSSTCIN-----MLKLPE-YPSKDV--LRDRLLVAL-------

>Branchiostoma_floridae_XM_002217655.1 .

---------DSSCKIVTKMN----------------SEKLK-ENIAVKFT-----GEEGM

----GQGVVREWFDILSKEILNP-------------------------------------

------------------------------------------------------------

------------------------------------------------------------

------------------------------------------------------------

------------------------------------------------------------

--------------------DYALFTMSADG-----------------------------

------------STFQPNSNS---------AVNPDHLNYFRFAG--GIMGLALYHR----

-----QLLNVYFTR-SFYKHILGIP-----------------------------------

----------------------------------------VNYHDVA---SIDPEYA---

KNLQWILDHDIS------------------------------------------DLGLEL

TFSVETDV-----------------------------FGAMEEVELKPDG----KNIQVT

EANK--AEYVQLVTELRMTRA-----IQPQINAFLQG---------------FHAFI---

--PCSLV-QLFDEYELELMLSGLPE--------------------------VDVEDFERN

TDYNS---------------GYTADCPVIK----TVR-DFPQQE----RVLLLQFVTGSS

RVPHGGFAYLPGGS----GMQKITISPVTYT--------------------------PNL

LP-TASTCIN-----LLKLPE-YRSREE--LRERLKVALQH-----

>Ciona_intestinalis_AABS01000468.1 .

LQVRRDKVFEDSCHELKKIN----------------QSNLK-HKFSVQFS-----NEEGI

----GDGVFREWFSVLSNEILNP-------------------------------------

------------------------------------------------------------

------------------------------------------------------------

------------------------------------------------------------

------------------------------------------------------------

--------------------EYGLFIQSFDG-----------------------------

------------CSFQPNSRS---------SINPDHLSYFEFAG--KILSVALYHK----

-----QLINGSLTS-SFYKHLLGRK-----------------------------------

----------------------------------------VDYRDVA---SIDPEYA---

TNLQWILDNDIT------------------------------------------EIGLEL

SFVVETDV-----------------------------FGRMEEIELTPGG----SKVAVT

EENK--QEYVQLVTELRMTRA-----IQPQLDAFIRG---------------FNEII---

--PSYLI-RIFTEDEMDLMFTGCKD--------------------------VDVGYWKSI

TEYSG---------------CYNQHHQVILWFWECVG-KMDTED----RSSLLHFATGRS

RLPS---PSVKLSN----TFVICNMSSQ-----------------------------KNL

LP-SASTCMS-----MLRLPE-YDSYDV--LETKLLTAI-------

>Ciona_savignyi_AACT01001556.1 .

-QVRREKVFEDSCQEFGKIS----------------AENLK-GNFMVQFT-----NEEGM

----GNGVLREWFSVLSNEILNP-------------------------------------

------------------------------------------------------------

------------------------------------------------------------

------------------------------------------------------------

------------------------------------------------------------

--------------------EYGLFVPSFDG-----------------------------

------------CSFQPNSRS---------SINPDHLSYFEFAG--KILSVALYHK----

-----HLINASLTS-SFYKHLLGRK-----------------------------------

----------------------------------------ISYEDVE---SIDPEYA---

TNLQWILDNDIS------------------------------------------DIGLNL

NFTVETDV-----------------------------FGRMEEIELTPGG----SRLSVT

EENK--QEYVQLVTELKMTSA-----IQRQLESFIRG---------------FNSII---

--PSYLI-RIFTPEEMDLMLTGCRE--------------------------IDVEIWKSI

TEYSG---------------CYNPTHQVIQWFWECVE-RMQNDD----RSLLLHFSTGRS

RLPA---PNVIFSN----KFMITSTPP------------------------------SDL

LP-SASTCMS-----LLKLPE-YDSYET--LETKLLTAI-------

>Monosiga_brevicollis_XM_001744158.1 .

LRIRRDYLFEDSYQRVMQLN----------------AGELR-GRLNVQFQ-----GEEGI

---DAGGLLREWYYTISQSIMNP-------------------------------------

------------------------------------------------------------

------------------------------------------------------------

------------------------------------------------------------

------------------------------------------------------------

--------------------NYALFCQSTPGS----------------------------

------------ETYQPNQHS---------SINVDHLRYFQFCG--RVVAKAIFDH----

-----QLLDCHFTR-AFYKQILGMH-----------------------------------

----------------------------------------VSWRDLA---AVDSSLY---

KNLLFILENDVT------------------------------------------PFEGDF

TFSLDVDR-----------------------------FGKLETIDLKPGG----RDLNVT

EENK--KEYVRLVADMKLTEA-----IKDQIKAFQKG---------------FYEVI---

--PQTDI-ALFNESELELLISGLPE--------------------------VDIDDLRAN

TDYHS---------------GLSASTPVIQWFWRAVR-SFSRDE----RIKLIQFVTGTG

RIPVGGFSKLVGMS----GPQKFNIQKDRSG--------------------------PQR

LP-QAHTCFN-----QLDLPE-YESYEQ--LREALKLAIMEASEGF

>Schistosoma_japonicum_AY814324.1 .

ITVSRDRIFEDSYARLHRRS----------------VSEWK-HKFVIRFQ-----NEEGQ

---DAGGPLREWFLLMSREIFNP-------------------------------------

------------------------------------------------------------

------------------------------------------------------------

------------------------------------------------------------

------------------------------------------------------------

--------------------NYCLFRVSPADR----------------------------

------------VTYTINPSS---------YINSNHLSYFKFVG--RFIAKAINDN----

-----KLLECYFTR-AFYKHILGVP-----------------------------------

----------------------------------------VRCSDLE---SEDYEFF---

KGLEFLLSHNVS------------------------------------------DLGYEL

TFSTEINE-----------------------------FGKTDTRDLIENG----RNVTVT

ESNK--KEYVRLVCQERMTGA-----IRQQLDAFLRG---------------FYDII---

--PKRMI-SIFNEQELELLISGLPN--------------------------IDIVDLKAN

TTYSK----------------YQLNSPQIEWFWQALE-SFDQED----LARFLQFVTGTS

KVPLGGFANLEGMH----GPTKFQISRASVSS-------------------------TNH

LP-SAHTCFN-----TLVLPA-YESFEQ--LRSRLLTAIRECSEGY

>Caenorhabditis_remanei_AAGD02012443.1 .

VQVSRNRVFSDSFRELFRLR----------------PSEWK-NRFYIIFQ-----GEEGQ

---DAGGLLREWFSVITREIFNP-------------------------------------

------------------------------------------------------------

------------------------------------------------------------

------------------------------------------------------------

------------------------------------------------------------

--------------------NYALFITAPGDM----------------------------

------------VTYMINKAS---------YINPEHLDYFKFVG--RLIAKSVFEH----

-----KYLDCYFTR-AFYKHILNLP-----------------------------------

----------------------------------------VRYQDLE---SEDPAFF---

KSLDFLLQNPID------------------------------------------DLGLDL

TFSTEVEE-----------------------------FGVRSVRDLKPNG----RKIDVN

DANK--DEYVKLVCQMKMTGS-----IRKQLDAFLTG---------------FYEII---

--PKDLI-SMFNEQELELLISGLPT--------------------------VDIDDMAAN

TDYKG----------------FQKTSTHIQWFWRALR-SFEKED----KAKFLQFVTGTS

KVPLQGFASLEGMN----GVQKFSIHMDSRG--------------------------GDR

LP-AAHT---------------------------------------

>Caenorhabditis_brenneri_ABEG01014746.1 .

VQVSRNRVFSDSFRELFRLR----------------PSEWK-NRFYIIFQ-----GEEGQ

---DAGGLLREWFSVITREIFNP-------------------------------------

------------------------------------------------------------

------------------------------------------------------------

------------------------------------------------------------

------------------------------------------------------------

--------------------NYALFITAPGDM----------------------------

------------VTYMINKAS---------YINPEHLDYFKFVG--RLIAKSVFEH----

-----KYLDCYFTR-AFYKHILNLP-----------------------------------

----------------------------------------VRYQDLE---SEDPAFF---

KSLDFLLQNPID------------------------------------------DLGLDL

TFSTEVEE-----------------------------FGVRSVRDLKQNG----RKIEVN

DANK--DEYVKLVCQMKMTGS-----IRKQLDAFLTG---------------FYEII---

--PKDLI-SMFNEQELELLISGLPT--------------------------VDIDDMAAN

TEYKG----------------FQKTSTHIQWFWRALR-SFEKED----KAKFLQFVTGTS

KVPLQGFASLEGMN----GVQKFSIHMDSRG--------------------------GDR

LP-AAHT---------------------------------------

>Caenorhabditis_elegans_NM_067883.3_eel1 .

VQVSRNRVFSDSFRELFRLR----------------PSEWK-NRFYIIFQ-----GEEGQ

---DAGGLLREWFSVITREIFNP-------------------------------------

------------------------------------------------------------

------------------------------------------------------------

------------------------------------------------------------

------------------------------------------------------------

--------------------NYALFITAPGDM----------------------------

------------VTYMINKAS---------YINPEHLDYFKFVG--RLIAKSVFEH----

-----KYLDCYFTR-AFYKHILNLP-----------------------------------

----------------------------------------VRYQDLE---SEDPAFF---

KSLDFLLQNPID------------------------------------------DLALDL

TFSTEVEE-----------------------------FGVRSVRDLKPNG----RKIEVN

DANK--DEYVKLVCQMKMTGS-----IRKQLDAFLTG---------------FYEII---

--PKDLI-SMFNEQELELLISGLPT--------------------------VDIDDMAAN

TDYKG----------------FQKTSTHIQWFWRALR-SFEKED----KAKFLQFVTGTS

KVPLQGFASLEGMN----GVQKFSIHMDSRG--------------------------GDR

LP-AAHTCFN-----QLDLPQ-YESYEK--LRQSLLLAIRECTEGF

>Caenorhabditis_briggsae_XM_001670716.1 .

VQVSRNRVFSDSFRELFRLR----------------PSDWK-NRFYIIFQ-----GEEGQ

---DAGGLLREWFSVITREIFNP-------------------------------------

------------------------------------------------------------

------------------------------------------------------------

------------------------------------------------------------

------------------------------------------------------------

--------------------NYALFITAPGDM----------------------------

------------VTYMINKAS---------YINPEHLDYFKFVG--RLIAKSVFEH----

-----KYLDCYFTR-AFYKHILNLP-----------------------------------

----------------------------------------VRYQDLE---SEDPAFF---

KSLDFLLQNSID------------------------------------------DLDLDL

TFSTEVEE-----------------------------FGVRSVRDLKPNG----RKIEVN

DANK--DEYVKLVCQMKMTGS-----IRKQLDAFLTG---------------FYEII---

--PKDLI-SMFNEQELELLISGLPT--------------------------VDIDDMAAN

TDYKG----------------FQKTSTHIQWFWRALR-SFEKED----KAKFLQFVTGTS

KVPLQGFASLEGMN----GVQKLSIHLDSRG--------------------------GDR

LP-AAHTCFN-----QLDLPQ-YDSYEK--LRQSLLLAIRECTEGF

>Brugia_malayi_XM_001897730.1 .

VRIRRSHLFSDSFRELFRLR----------------GPEWK-ARFYIIFE-----GEEGQ

---DAGGLLREWFSIITREIFNP-------------------------------------

------------------------------------------------------------

------------------------------------------------------------

------------------------------------------------------------

------------------------------------------------------------

--------------------NYALFITSPGDR----------------------------

------------VTYMINKSS---------YINPEHLEYFKFVG--RIIAKAIYEN----

-----KLLECYFTR-AFYKHILSVP-----------------------------------

----------------------------------------VRAQDLE---SEDPSFY---

KSLEFLLNNPIE------------------------------------------DLGTEL

TFSLEVEE-----------------------------FGVRKMRMLKENG----SSVPVT

DGNK--EEYVKLVCQMKMTGS-----INQQLNAFLEG---------------FYEII---

--PKHLI-SIFNEQELELLISGLPN--------------------------VDIDDLYAN

TEYKT----------------YTKSSSQIQWFWKALR-SFEQED----RAKFLQFVTGTS

KVPLQGFAALEGMN----GTQKFSIHLDSRS--------------------------SDR

LP-TAHTCFN-----QLDLPQ-YETYDK--LRDMLLLAVRECTEGF

>Trichoplax_adhaerens_XM_002111836.1 .

IRVRRTHVFEDSFKELSRRT----------------ADQMK-SKMFIHFE-----GEDGQ

---DAGGLLREWYLIISREIFNP-------------------------------------

------------------------------------------------------------

------------------------------------------------------------

------------------------------------------------------------

------------------------------------------------------------

--------------------NYALFTTSSGDR----------------------------

------------VTYIINSSS---------HVNHYHLEYFKFVG--RMIAKALFDN----

-----KLLDVYFSR-SMYKHILGSA-----------------------------------

----------------------------------------VHYSDIE---SEDYTFY---

QSMKYLLEHNIS------------------------------------------EIDNEL

TYSTDVCE-----------------------------FGKSEIRDLKPNG----RNISVT

EANK--LEYVHLMCQMKMTGA-----VRKQIASFLEG---------------FYEII---

--PKRLI-SIFDPQELELLISGLPT--------------------------IDIDDLKAN

TDYSK----------------YTKDSIQIQWFWRALR-SYDQAS----RAKFLQFVTGTS

RVPLQGFAALEGMN----GPQKFSIHRDDMS--------------------------TDR

LP-SAHTCFN-----QLDLPA-YETYDK--LNSLLSTAINECPEGF

>Ciona_intestinalis_XR_052983.1 .

IRVQRDHLFEDSFRELHRRT----------------PAELR-SRLYVVFD-----GEDGQ

---DAGGVLREWYLVISREIFNP-------------------------------------

------------------------------------------------------------

------------------------------------------------------------

------------------------------------------------------------

------------------------------------------------------------

--------------------MYALFRTSPGDH----------------------------

------------GTYTINPLS---------YINPNHLSYFKFVG--RIVAKAIYDN----

-----KLLECYFTR-SFYKHILGKP-----------------------------------

----------------------------------------VKYTDME---ADDYEFS---

QGLRYLLEHDIT------------------------------------------SLGTEL

FFSVEIEE-----------------------------FGKTEVRDLKENG----RNLPVS

EKNK--REYAHLVCQEKMTGA-----IKKQLAAFLEG---------------FYEII---

--PKRLI-SIFDEQELELLISGLPN--------------------------VDIDDLRQN

TEYHK----------------YQPNSPQIQWFWRALR-SFDQAE----RAKFLQFVTGTS

KVPLQGFSTLEGMT----GVQKFQIHRDDRS--------------------------TAR

LP-CAHTCFN-----QLDLPA-YENYDK--LRERLLLAITECTEGF

>Hydra_magnipapillata_XM_002157573.1 .

VHIRREHIFEDSYRELHRRT----------------AEELK-NRLYVVFD-----GEEGQ

---DAGGLLREWYGIMAREMFNP-------------------------------------

------------------------------------------------------------

------------------------------------------------------------

------------------------------------------------------------

------------------------------------------------------------

--------------------NYALFTNSQGEK----------------------------

------------STYLPNQHS---------HCNPNHLSYFKFAG--RIVAKAIYDN----

-----KLLDCYVQ-----------------------------------------------

------------------------------------------------------------

------------------------------------------------------------

-------V-----------------------------FGMNEIHDLKPNG----RNIPVT

EDTK--REYVKLVCQEKMTGS-----IRQQIGSFLEG---------------FYEII---

--PKRLI-SIFDEQELELLIAGLPT--------------------------IDIEDLKMN

TEYHK----------------YTENSLQIQWLWRALR-SFDQAD----RAKFLQFVTGTS

KVPLQGFVSLEGMN----GPQKFQIHRDDRS--------------------------TDR

LP-CAHTCFN-----QLDLPA-YETYDK--LHSQMLKAINECPEGF

>Drosophila_ananassae_XM_001966528.1 .

VSVRRVTVFEDSFRVLYRLG----------------PEEWK-NRFYIVFE-----DEEGQ

---DAGGLLREWYVIISREIFNP-------------------------------------

------------------------------------------------------------

------------------------------------------------------------

------------------------------------------------------------

------------------------------------------------------------

--------------------MYALFCVSPGDR----------------------------

------------VTYMINPSS---------HANPNHLSYFKFVG--RVIAKAVHDN----

-----KLLECYFTR-SFYKHILGKQ-----------------------------------

----------------------------------------VKHTDME---SQDYEFY---

KGLDYLMKNDIS------------------------------------------TLGYEL

TFSTEVQE-----------------------------FGVTQIRDLKPNG----RDIAVT

EENK--FEYVQLVCQLKMSGS-----IRQQLDAFLEG---------------FYDII---

--PKHLI-SIFNEQELELLISGLPD--------------------------IDIEDLKAN

TEYHK----------------YTSKSAQIQWFWRALR-SFDQAD----RAKFLQFVTGTS

KVPLQGFGSLEGMN----GIQKFQIHRDDRS--------------------------TDR

LP-CAHTCFN-----QLDLPM-YKSYDK--LRSCLLKAIHECSEGF

>Drosophila_pseudoobscura_XM_002133748.1 .

VSVRRVTVFEDSFRVLYRLG----------------PEEWK-NRFYIVFE-----DEEGQ

---DAGGLLREWYVIISREIFNP-------------------------------------

------------------------------------------------------------

------------------------------------------------------------

------------------------------------------------------------

------------------------------------------------------------

--------------------MYALFCVSPGDR----------------------------

------------VTYMINPSS---------HANPNHLSYFKFVG--RVIAKAVHDN----

-----KLLECYFTR-SFYKHILGKQ-----------------------------------

----------------------------------------VKHTDME---SQDYEFY---

KGLDYLMKNDIS------------------------------------------NLGYEV

TFSTEVQE-----------------------------FGVTQIRDLKTNG----RDIPVT

EDNK--FEYVQLVCQLKMSGS-----IRQQLDAFLEG---------------FYDII---

--PKHLI-SIFNEQELELLISGLPD--------------------------IDIEDLKAN

TEYHK----------------YTSKSAQIQWFWRALR-SFDQAD----RAKFLQFVTGTS

KVPLQGFGSLEGMN----GIQKFQIHRDDRS--------------------------TDR

LP-CAHTCFN-----QLDLPM-YKSYDK--LRSCLLKAIHECSEGF

>Drosophila_grimshawi_AAPT01018882.1 .

--------------------------------------------------------EEGQ

---DAGGLLREWYVIISREIFNP-------------------------------------

------------------------------------------------------------

------------------------------------------------------------

------------------------------------------------------------

------------------------------------------------------------

--------------------MYALFCVSPGDR----------------------------

------------VTYMINPSS---------HANPNHLSYFKFVG--RVIAKAVHDN----

-----KLLECYFTR-SFYKHILGKQ-----------------------------------

----------------------------------------VKHTDME---SQDYEFY---

KGLDYLMKNDIS------------------------------------------TLGYEL

TFSTEVQE-----------------------------FGVTQIRDLKPNG----RDVAVT

EENK--FEYVQLVCQLKMSGS-----IRQQLDAFLEG---------------FYDII---

--PKHLI-SIFNEQELELLISGLPD--------------------------IDIEDLKAN

TEYHK----------------YTSKSAQIQWFWRALR-SFDQAD----RAKFLQFVTGTS

KVPLQGFGSLEGMN----GIQKFQIHRDDRS--------------------------TDR

LP-CAHTXFN-----QLDLPM-YKSYDK--LRSCLLKAIHECSEGF

>Salmo_salar_BT072724.1 .

VHVRRDHVFEDSYRELHRKS----------------PEDMK-NRLYIVFE-----GEEGQ

---DAGGLLREWYMIISREMFNA-------------------------------------

------------------------------------------------------------

------------------------------------------------------------

------------------------------------------------------------

------------------------------------------------------------

--------------------MYALFRTSPGDR----------------------------

------------VTYTINPSS---------HCNPNHLSYFKFVG--RVVAKAVYDN----

-----RLLECYFTR-SFYKHILGKS-----------------------------------

----------------------------------------VRYTDME---SEDYPFF---

QGLVYLLENNVS------------------------------------------TLGYEL

TFSTEVQE-----------------------------FGVCEVRDLKPNG----GNIIVT

EENK--KEYVHLVCQMKMTGA-----IRKQLSAYLEG---------------FYEII---

--PKRLI-SIFTEQELELLISGLPT--------------------------IDIDDLKAN

TEYHK----------------YQSSSIQIQWFWRALR-SFDQAD----RAKFLQFVTGTS

KVPLQGFAALEGMN----GIQKFQIHRDDRS--------------------------TDR

LP-SAHTCFN-----QLDLPA-YESYEK--LRHMLLLAIQECSEGF

>Danio_rerio_XM_001923865.1 .

VHVRRDHVFEDSYRELHRKS----------------PEDMK-NRLYIVFE-----GEEGQ

---DAGGLLREWYMIISREMFNP-------------------------------------

------------------------------------------------------------

------------------------------------------------------------

------------------------------------------------------------

------------------------------------------------------------

--------------------MYALFRTSPGDR----------------------------

------------VTYTINPSS---------HCNPNHLSYFKFVG--RVVAKAVYDN----

-----RLLECYFTR-SFYKHILGKS-----------------------------------

----------------------------------------VRYTDME---SEDYPFF---

QGLVYLLENDVS------------------------------------------TLGYEL

TFSTEVQE-----------------------------FGVCEVRDLKANG----ANILVT

EENK--KEYVHLVCQMKMTGA-----IRKQLAAFLEG---------------FYEII---

--PKRLI-SIFTEQELELLISGLPT--------------------------IDIDDLKAN

TEYHK----------------YQSSSIQIQWFWRALR-SFDQAD----RAKFLQFVTGTS

KVPLQGFAALEGMN----GIQKFQIHRDDRS--------------------------TDR

LP-SAHTCFN-----QLDLPA-YESYEK--LRHMLLLAIQECSEGF

>Homo_sapiens_AK223167.1 .

VHVRRDHVFEDSYRELHRKS----------------PEEMK-NRLYIVFE-----GEEGQ

---DAGGLLREWYMIISREMFNP-------------------------------------

------------------------------------------------------------

------------------------------------------------------------

------------------------------------------------------------

------------------------------------------------------------

--------------------MYALFRTSPGDR----------------------------

------------VTYTINPSS---------HCNPNHLSYFKFVG--RIVAKAVYDN----

-----RLLECYFTR-SFYKHILGKS-----------------------------------

----------------------------------------VRYTDME---SEDYHFY---

QGLVYLLENDVS------------------------------------------TLGYDL

TFSTEVQE-----------------------------FGVCEVRDLKPNG----ANILVT

EENK--KEYVHLVCQMRMTGA-----IRKQLAAFLEG---------------FYEII---

--PKRLI-SIFTEQELELLISGLPT--------------------------IDIDDLKSN

TEYHK----------------YQSNSIQIQWFWRALR-SFDQAD----RAKFLQFVTGTS

KVPLQGFAALEGMN----GIQKFQIHRDDRS--------------------------TDR

LP-SAHTCFN-----QLDLPA-YESFEK--LHHMLLLAIQECSEGF

>Mus_musculus_BC079665.1 .

------------------------------------------------------------

-------ILREWYMIISREMFNP-------------------------------------

------------------------------------------------------------

------------------------------------------------------------

------------------------------------------------------------

------------------------------------------------------------

--------------------MYALFRTSPGDR----------------------------

------------VTYTINPSS---------HCNPNHLSYFKFVG--RIVAKAVYDN----

-----RLLECYFTR-SFYKHILGKS-----------------------------------

----------------------------------------VRYTDME---SEDYHFY---

QGLVYLLENDVS------------------------------------------TLGYDL

TFSTEVQE-----------------------------FGVCEVRDLKPNG----ANILVT

EENK--KEYVHLVCQMRMTGA-----IRKQLAAFLEG---------------FYEII---

--PKRLI-SIFTEQELELLISGLPT--------------------------IDIDDLKSN

TEYHK----------------YQSNSIQIQWFWRALR-SFDQAD----RAKFLQFVTGTS

KVPLQGFAALEGMN----GIQKFQIHRDDRS--------------------------TDR

LP-SAHTCFN-----QLDLPA-YESFEK--LRHMLLLAIQECSEGF

>Strong._purpuratus_XR_026553.1 .

IHVRREHVFEDSYRELHRRT----------------PEQWK-NRFYVVFE-----GEEGQ

---DAGGLLREWYLIISKEIFNQ-------------------------------------

------------------------------------------------------------

------------------------------------------------------------

------------------------------------------------------------

------------------------------------------------------------

--------------------MYALFRTSPGDR----------------------------

------------VTYIPNPSS---------HCNSNHLSYFKFVG--RIMAKAIYDN----

-----KLLECYFSR-SFYKHILGKP-----------------------------------

----------------------------------------VKYTDME---SEDYAFY---

QGLVFLLEHDVS------------------------------------------ELGYDL

TFSTEIEE-----------------------------FGVTEARDLKPNG----SNLIVT

EDNK--QEYVHLVCQMKMTSA-----IRKQIDSFLEG---------------FYDII---

--PKKLI-GIFNEQELELLIAGLPT--------------------------IDVDDLKAN

TEYHK----------------YQSNSLQIQWFWRALR-SFDQAT----RAKFVQFVTGTS

KVPLQGFASLEGMN----GPQKFQIHRDDRS--------------------------TDR

LP-TAHTCFN-----QLDLPA-YETYDK--LRRMLLLAIEECTEGF

>Branchiostoma_floridae_XM_002235531.1 .

VHVRRDHVFEDSFRELHRRT----------------PEELK-NRLYIVFE-----GEEGQ

---DAGGLLREWYLIISREIFNP-------------------------------------

------------------------------------------------------------

------------------------------------------------------------

------------------------------------------------------------

------------------------------------------------------------

--------------------NYALFTTSPGDR----------------------------

------------VTYRPNPSS---------HCNPNHLSYFKFVG--RVIGKAIYDN----

-----KLLECYFTR-SFYKHILGKN-----------------------------------

----------------------------------------VKYTDME---SEDYQFY---

QGLTFLLENNIE------------------------------------------ESGLEL

TFSTEIQE-----------------------------FGVTEVRDLKQNG----RNITVT

EDHK--HEYVKLVCQLKMTGS-----IRKQIDAFLEG---------------FYEII---

--PKRLI-SIFNEQELELLISGLPN--------------------------IDLDDLKAN

SEYHK----------------YQSNSLQIQWFWRALR-SYDQAD----RAKFLQFVTGTS

KVPLQGFSHLEGMN----GTQKFQIHRDDRS--------------------------TDR

LP-SAHTCFN-----QLDLPP-YETYEK--LHYMLKIAIQECSEGF

>Ixodes_scapularis_ABJB010915401.1 .

VHVRREHVFEDSFRELHRRP----------------PEEWK-NRFYIVFE-----GEEGQ

---DAGGLLREWYTIISREIFNP-------------------------------------

------------------------------------------------------------

------------------------------------------------------------

------------------------------------------------------------

------------------------------------------------------------

--------------------MYALFTTSPGDR----------------------------

------------VTYMINPAS---------HCNSNHLSYFKFVG--RVIAKAVYDN----

-----KLLECYFTR-SFYKHILGKP-----------------------------------

----------------------------------------VKYTDME---SEDYSFY---

QGLVFLLEHGVR------------------------------------------ALGYEL

TFSVEVQE-----------------------------FGVTEVRDLKPGG----RHLPVT

EETT--QEYVRLVCQEKMTGA-----IRRQLNAFLEG---------------FYEII---

--PKRLI-AIFNEQELELLISGLPS--------------------------IDVDDLRAH

TEYHK----------------YQPNSLQIQWFWRALR-SLDQAD----RAKFLQFVTGTS

KVPLQGFVALEGMN----GVQRFQIHRDDRS--------------------------TDR

LP-SAHT---------------------------------------

>Tribolium_castaneum_XM_963912.1 .

VHVRRSNIFEDSFRELYRRT----------------PEEWK-NRFYIVFE-----DEEGQ

---DAGGLLREWYVIISRDIFNP-------------------------------------

------------------------------------------------------------

------------------------------------------------------------

------------------------------------------------------------

------------------------------------------------------------

--------------------MYALFTVSPGDR----------------------------

------------VTYMINSAS---------HYNPNHLCYYKFVG--RVIAKAIYDN----

-----KLLECYFTR-SFYKHILGIP-----------------------------------

----------------------------------------VKYTDME---SEDYSFY---

RGLVYLMENNIN------------------------------------------NLGLDL

TFSTEINE-----------------------------FGVTETRDLIVNG----RHVPVT

EETK--MEYIRLSCQMKMTGA-----IKQQLNAFLDG---------------FYDII---

--PMRLI-SIFNEQELELLISGLPN--------------------------VDIDDLKAN

TEYHK----------------YQANSLQIQWFWRALR-SFDQAD----RAKFLQFVTGTS

KVPLQGFAALEGMN----GVQKFQIHRDDRS--------------------------TDR

LP-SAHTCFN-----QLDLPV-YETYDK--LRSYLLKAIHECSEGF

>Apis_mellifera_XM_001120794.1 .

VHVRRNNVFEDSFRELHRRN----------------ADEWK-NRFYIVFE-----GEEGQ

---DAGGLLREWYVIISREIFNP-------------------------------------

------------------------------------------------------------

------------------------------------------------------------

------------------------------------------------------------

------------------------------------------------------------

--------------------MYALFTVSPGDR----------------------------

------------VTYMINSSS---------HCNPNHLCYYKFVG--RVIAKAIYDN----

-----KLLECYFTR-SFYKHILGIL-----------------------------------

----------------------------------------VKHTDME---SEDYSFY---

KGLVYLTEHNIA------------------------------------------DLGYEL

TFSTEVNE-----------------------------FGVNDVRDLIPNG----RNIIVT

EETK--LEYIRLVCQMKMTGA-----IRKQLNAFLEG---------------FYDII---

--PKRLI-SIFNEQELELLISGLPN--------------------------VDIEDLK--

----------------------------IQWFWRALR-GFDQAD----RAKFLQFVTGTS

KVPLQGFAALEGMN----GIQKFQIHREDRS--------------------------TDR

LP-SAHTCFN-----QLDLPV-YETYDK--LRTNLLKAIHECSEGF

>Hydra_magnipapillata_XM_002155916.1 .

---------------------------------------------------------EGI

---DYGGLTREFIQLLLSELLHK-------------------------------------

------------------------------------------------------------

------------------------------------------------------------

------------------------------------------------------------

------------------------------------------------------------

--------------------KGALFRKFNDDDSQ--------------------------

------------ALIHPNPFR---------EKTYQNIKLYEFLG--LLIGKCLVECSF--

-GQHFASDDKLYYT-GKIKYILENNPECLEIYFTEDVYDNDN------------------

----------------------------------------KFIKHFA---SDDKLYY--T

GKIKYILENN--------------------------------------------PECLEI

YFTEDVYD---------------------------NDNKFIKVVDLVTNG----SQIQVV

DSNK--EIYLQRLAEFRLHES-----VSEEIDSFLKD---------------FSTAS---

--GLDVIP-IT-------------------------------------------------

---------------------------VMSWFWSILS-SFSQEE----LSRLLQFTTGCG

QLPPEGFAGLYPNF----QITRAGL--------------------------------TDS

LP-TAHTCFN-----NLCLPL-YTSREE--MKKKLIIAMNEGSEGF

>Trichoplax_adhaerens_XM_002115757.1 .

----------------------------------------------VSFE-----LEEAL

---DWGGVRRELFEILNTELFTPS------------------------------------

------------------------------------------------------------

------------------------------------------------------------

------------------------------------------------------------

------------------------------------------------------------

--------------------ENGLFTTFDIENKQ--------------------------

------------ALIHPNTKR----------ADSYPLSFYELCG--KIVGKCLYDSAQ--

GEAYHLLVKARFSR-SFLAQMLGLR-----------------------------------

----------------------------------------VSYDYFS---SDDPELY--K

TKIKYILDND--------------------------------------------VTELNL

TFAEEEYS---------------------------SSGNVVKVVDLKSNG----RFITVT

EENK--IEYLNLLSQHRLAKK-----FTSEMDHFLKG---------------VHLLI---

--PENLLA-IFDESELELLMCGIGN--------------------------ISISDMKLN

CIATD---------------SSRSFGKILRWFWAIVG-TFSQEE----LARLLQFVTGSS

QLPPGGFKELRPSL----QISPALV--------------------------------QNG

LP-TSHTCFN-----QLCLPE-YDSSAE--LKRCLILAINEGSEGF

>Nematostella_vectensis_XM_001622318.1 .

LKINRSNLLESSYVACK-------------------NVDWL-KMFNVTFE-----GEEGL

---DWGGVRREWFELLCIALFGR-------------------------------------

------------------------------------------------------------

------------------------------------------------------------

------------------------------------------------------------

------------------------------------------------------------

--------------------DSELFTRFKGDDPQ--------------------------

------------APVHPNPRR----------PPHLNLKYYKFAG--QVVSKCIYESAI--

SNARRQNVKAKFTR-SFLAQLLGLR-----------------------------------

----------------------------------------VNYKYLE---SDDKDLY--R

SKVQLIEKND--------------------------------------------PADLEL

KFTEEEYN---------------------------SSGQLEKVVELLPGG----RLIDVT

EQNK--CEYLNLMARYKLVES-----VQKEVEAFIQG---------------LNELV---

--PDNLLG-MFDENELELLMCGTGH--------------------------ISVADMKAH

HHVLA--------------GGGQRFTKIMDWFWTIVS-SLTQEE----LARLLQFVTGSS

QLPPGGFAELSPQL----QISYIAT--------------------------------SQA

LP-TAHTCFN-----QLCLPD-FPSFNE--MQRKLLLAVNEGCEGF

>Trichoplax_adhaerens_XM_002109081.1 .

VSISRQHALRDSMKQLKNAS----------------ASDWC-RPFQIKFQ-----GEQAE

---DLGGVLREWFHVLSRALFAP-------------------------------------

------------------------------------------------------------

------------------------------------------------------------

------------------------------------------------------------

------------------------------------------------------------

--------------------SNQLFVGFRDDNQ---------------------------

------------ALVRPNNAR---------PPEFAKIKYFEFAG--KIVGKCLLESAI--

TRDSNCYIRACFAR-SFLAQILGLR-----------------------------------

----------------------------------------VHYKHFE---NDDPEFF--T

NKIQYILSND--------------------------------------------IADLSL

VFAEEIYS---------------------------SDGKLEKIIELKPNG----KNCVVN

NDNK--IEYLDLLAQQRLCKE-----IQEEKNAFLKG---------------LNDLI---

--PDSLLS-IFDENELELLICGTCE--------------------------YDINDLKLN

HAVIG---------------RTSEFEKILSWFWSTIA-NFTQQE----MARLLQFVTGCS

QLPPEGFSELSPKF----QIVNAMV--------------------------------TGT

LP-TAHTCFN-----ELCLPI-YDSLDD--MQHSLLIAINEGSEGF

>Ciona_intestinalis_XM_002122587.1 .

VNVDRDDLLNSSYKATKHFS----------------TSDWC-RQFVVAFK-----GETGL

---DWGGVNREWIHLLCKIFFQSKDA----------------------------------

------------------------------------------------------------

------------------------------------------------------------

------------------------------------------------------------

------------------------------------------------------------

--------------------GCGIFRSMKEDAQ---------------------------

------------ALVLPASDS------------EVKIKHFEFAG--KLVGKCLLESAV--

GGEYARQVTARFAR-SFLAQLIGLP-----------------------------------

----------------------------------------ATFAHFE---SDDPDLY--M

SKVKYILEHD--------------------------------------------VTDAEL

TFSEEQFT---------------------------SSGSLSKVIDLVPNG----ANVAVT

NDNK--IKYLNRIAQYRLSES-----IKNELALFIKG---------------LTSIV---

--PDHLLS-VFDENELELVMCGSSK--------------------------ISPDDFKLH

CVVNS--------------GMDPTFQKILSWFWSLIS-TFTQDE----LARLLQFTTGCS

QLPPDGFKALEPKF----KISSIEYK-------------------------------TGG

LP-MAHTCFN-----ELCLPN-YESYDD--LHKMLKISITEGITG-

>Strong._purpuratus_XM_785947.2 .

LVIDRHNLLESSMKATKSLS----------------TSEWC-KKFDIHFV-----NEEGQ

---DWGGLMREWVNLLCVSLFDP-------------------------------------

------------------------------------------------------------

------------------------------------------------------------

------------------------------------------------------------

------------------------------------------------------------

--------------------ENKLFKRFEDDNNQ--------------------------

------------ALVHPNPNR---------PTYLSKLKYYEFAG--KLIGKCLFESSVPT

ATASQMVVKARFTR-SFLAQLIGLR-----------------------------------

----------------------------------------VTHKYFE---TDDSEFF--T

TKVRYIRDNE--------------------------------------------VESMEL

NFSEEVYD---------------------------AEGHLEQVVELLPGG----SSLQVT

EDNK--MQYLDLLAQYRLSTS-----VQEEIDAFLKG---------------LNDLI---

--PDNLLS-MFDENELELLICGTCC--------------------------FDLADLKAH

HILAG---------------AGPQFRKMIEWFWIVIA-SFTQEE----MSRLLQFTTGCS

QLPPGGFAELRPKF----QLVAAPT--------------------------------HGI

LP-TAHTCFN-----QLCLPT-YDTIEH--LQKSIVLAITEGNVGF

>Branchiostoma_floridae_XM_002219137.1 .

VKVNRRDLLDSSMKSTKHFS----------------SSDWG-KTFVVSFY-----GEEGL

---DWGGLSREWFQLVTETLFRP-------------------------------------

------------------------------------------------------------

------------------------------------------------------------

------------------------------------------------------------

------------------------------------------------------------

--------------------DTQLFRRFKDDNQ---------------------------

------------GLVHPNPDR---------PLPVTKPKYYEFAG--KVVGKCLFESAK--

GGSSRQLVKARFSR-SFLAQLIGLR-----------------------------------

----------------------------------------VTYKHFA---ADDPEFY--K

TKVHYIETND--------------------------------------------VEDMEL

TFTEEVYD---------------------------VSGKLVKTVELVPGG----AHMQVT

NANK--LQYLDSLAQYRLAAT-----VQEELEHFLKG---------------LNELI---

--PDNLLS-IFDENELELLICGTGD--------------------------YSIADFKQH

CEIQG--------GAWGFEKYWGKHLNVLDWFWTIVA-SFTQEE----VARLLQFTTGSS

QLPPGGFAELNPRF----QICSVPV--------------------------------RGI

LP-TAHTCFN-----QLCLPD-YDSCEQ--LHKMLTLAITEGSQGF

>Drosophila_simulans_AAGH01016301.1 .

LKVQREKILESSMKAAKGFS----------------VSDWC-GNFEVTFQ-----GEQGI

---DWGGLRREWFELVCSALFDA-------------------------------------

------------------------------------------------------------

------------------------------------------------------------

------------------------------------------------------------

------------------------------------------------------------

--------------------RGGLFCTFHDKHQ---------------------------

------------ALVHPNPTR----------PAHLKLKHFEFAG--KMVGKCLFESAL--

GGTYRQLVRARFSR-SFLAQLIGLRVHYKVSRQKRNNKSSYLNAF---------------

----------------------------------------LTYQYFE---QDDPDLY--L

SKIKYILDTDLDA-----------------------------------------TDTLEL

YFVEEMYD--------------------------SSSGQLSKTIELIPNG----AKTRVT

NATK--NQYLDALAQQRLCNN-----VKDEVDSFLKG---------------LNSII---

--PDNLLS-IFDENELELLMCGTGE--------------------------YSISDFKAH

HIANG---------------NSAEFRRVLAWFWAGVS-NFSQTE----MARLLQFTTGCS

QLPPGGFQELNPQF----QITAAPT--------------------------------FGN

LP-TAHT---------------------------------------

>D_melanogaster_NM_001103583.1_CG4238 .

LKVQREKILESSMKAVKGFS----------------VSDWC-GNFEVTFQ-----GEQGI

---DWGGLRREWFELVCSALFDA-------------------------------------

------------------------------------------------------------

------------------------------------------------------------

------------------------------------------------------------

------------------------------------------------------------

--------------------RGGLFCTFHDKHQ---------------------------

------------ALVHPNPTR----------PAHLKLKHFEFAG--KMVGKCLFESAL--

GGTYRQLVRARFSR-SFLAQLIGLR-----------------------------------

----------------------------------------VHYKYFE---QDDPDLY--L

SKIKYILDTDLDA-----------------------------------------TDTLEL

YFVEEMYD--------------------------SSSGQLSKTIELIPNG----AKTRVT

NATK--NQYLDALAQQRLCNN-----VKDEVDSFLKG---------------LNSII---

--PDNLLS-IFDENELELLMCGTGE--------------------------YSISDFKAH

HIANG---------------NSAEFRRVLAWFWAGVS-NFSQTE----MARLLQFTTGCS

QLPPGGFQELNPQF----QITAAPT--------------------------------FGN

LP-TAHTCFN-----QLCLPD-YESYEQ--FEKSLLLAISEGSEGF

>Drosophila_pseudoobscura_XM_001356913.2 .

LKVQREKILESSMKATKGFS----------------VSDWC-GNFEVTFQ-----GEQGI

---DWGGLRREWFELVCSSLFDA-------------------------------------

------------------------------------------------------------

------------------------------------------------------------

------------------------------------------------------------

------------------------------------------------------------

--------------------RGGLFCTFHDKHQ---------------------------

------------ALVHPNPTR----------AAHLKLKHFEFAG--KMVGKCLFESAL--

GGSYRQLVRARFSR-SFLAQLIGLR-----------------------------------

----------------------------------------VHYKYFE---QDDPDLY--L

SKIKYILDTDLDA-----------------------------------------TDTLEL

YFVEELYD--------------------------ASNGQLSKTIELIPNG----AKTRVT

NASK--NQYLDSLAQQRLCNS-----VKDEVDSFLKG---------------LNSII---

--PDNLLS-IFDENELELLMCGTGE--------------------------YSISDFKSH

HIANG---------------NSAEFRRVLAWFWAGVS-NFSQTE----MARLLQFTTGCS

QLPPGGFQELNPQF----QITAAPT--------------------------------FGN

LP-TAHTCFN-----QLCLPD-YESYEQ--FEKSLLLAISEGSEGF

>Drosophila_ananassae_XM_001962088.1 .

LKVQRDKILESSMKSTKGFS----------------VSDWC-GNFEVTFQ-----GEQGI

---DWGGLRREWFELVCSSLFDA-------------------------------------

------------------------------------------------------------

------------------------------------------------------------

------------------------------------------------------------

------------------------------------------------------------

--------------------RGGLFCTFHDKHQ---------------------------

------------ALVHPNPTR----------PAHLKLKHFEFAG--KMVGKCLFESAL--

GGSYRQLVRARFSR-SFLAQLIGLR-----------------------------------

----------------------------------------VHYKYFE---QDDPDLY--L

SKIKYILDTDLDA-----------------------------------------TDTLEL

YFVEELYD--------------------------ASSGQLSKTIELIPNG----ARTRVT

NASK--NQYLDALAQQRLCNS-----VKDEVDSFLKG---------------LNSII---

--PDNLLS-IFDENELELLMCGTGE--------------------------YSISDFKSH

HIANG---------------NSAEFRRVLAWFWAGVS-NFSQTE----MARLLQFTTGCS

QLPPGGFQELNPQF----QITAAPT--------------------------------FGN

LP-TAHTCFN-----QLCLPD-YESYEQ--FEKSLLLAISEGSEGF

>Drosophila_willistoni_XM_002064708.1 .

LKVQRDKILESSMKAAKGFS----------------VSDWC-GNFEVTFQ-----GEQGI

---DWGGLRREWFELVCSSLFDA-------------------------------------

------------------------------------------------------------

------------------------------------------------------------

------------------------------------------------------------

------------------------------------------------------------

--------------------RGGLFCTFHDKHQ---------------------------

------------ALVHPNPTR----------PPQLKLKHFEFAG--KMVGKCLFESAL--

GGSYRQLVRARFSR-SFLAQLIGLR-----------------------------------

----------------------------------------VHYKYFE---QDDPDLY--L

SKIKYILDTDLDA-----------------------------------------TDTLEL

YFVEEVYD---------------------------GSGQLSKTVELIPNG----AKTRVT

NATK--NQYLDSLAQQRLCNS-----VKDEVDSFLKG---------------LNSII---

--PDNLLS-IFDENELELLMCGTGE--------------------------YSISDFKSH

HIANG---------------NSAEFRRVLGWFWAGVS-NFSQTE----MARLLQFTTGCS

QLPPGGFQELNPQF----QITAAPT--------------------------------FGN

LP-TAHTCFN-----QLCLPD-YESYEQ--FEKSLLLAISEGSEGF

>Drosophila_grimshawi_XM_001996446.1 .

LKVNREKILESSMKSTKGFS----------------VSDWC-GNFEVTFQ-----GEQGI

---DWGGLRREWFELVCSSLFDS-------------------------------------

------------------------------------------------------------

------------------------------------------------------------

------------------------------------------------------------

------------------------------------------------------------

--------------------RGGLFCTFHDKHQ---------------------------

------------ALVHPNPTR----------PAHLKLKYFEFAG--KMVGKCLFESAL--

GGSYRQLVRARFSR-SFLAQLIGLR-----------------------------------

----------------------------------------VHYKYFE---QDDPDLY--L

SKIKYILDTDLDS-----------------------------------------TDTLEL

YFVEDLYD---------------------------ISGQLSKTIELIPNG----AKTRVT

NASK--NQYLDALAQQRLCNS-----VKDEVDSFLKG---------------LNAII---

--PDNLLS-IFDENELELLMCGTGE--------------------------YSISDFKSH

HIANG---------------NSSEFRRVLAWFWAGVS-NFSQTE----MARLLQFTTGCS

QLPPGGFQELNPQF----QITAAPT--------------------------------FGN

LP-TAHTCFN-----QLCLPD-YESYEQ--FEKSLLLAISEGSEGF

>Drosophila_mojavensis_XM_002004021.1 .

LKVNREKILESSMKATKGFS----------------VSDWC-GNFEVTFQ-----GEQGI

---DWGGLRREWFELVCSALFDS-------------------------------------

------------------------------------------------------------

------------------------------------------------------------

------------------------------------------------------------

------------------------------------------------------------

--------------------RSGLFCTFHDKHQ---------------------------

------------ALVHPNPTR----------PSHLKLKYFEFAG--KIVGKCLFESAL--

GGSYRQLVRARYSR-SFLAQLIGLR-----------------------------------

----------------------------------------VHYKYFE---QDDPDLY--L

SKIKYILDTDLDG-----------------------------------------TDTLEL

YFVEDVYD---------------------------TSGQLIKSIELIPNG----AKTRVT

NATK--NQYLDALAQQRLCNS-----VKDEVDSFLKG---------------LNAII---

--PDNLLS-IFDENELELLMCGTGE--------------------------YSITDFKSH

HITNG---------------NSAEFRRVLAWFWAAVS-NFSQTE----MARLLQFTTGCS

QLPPGGFQELNPQF----QITAAPT--------------------------------FGN

LP-TAHTCFN-----QLCLPD-YESYEQ--FEKSLLLAISEGSEGF

>Anopheles_gambiae_XM_310176.4 .

LKVQRDKILESSMKATKNFS----------------VSDWC-GNFEVTFQ-----GEQGI

---DWGGLRREWFELVCSALFDP-------------------------------------

------------------------------------------------------------

------------------------------------------------------------

------------------------------------------------------------

------------------------------------------------------------

--------------------RGGLFCTFHDKRQ---------------------------

------------ALVHPNPNR----------PPHLKLKHFEFAG--KVVGKCLYESAL--

GGTYRQLVRARFSR-SFLAQLIGLR-----------------------------------

----------------------------------------VHYKYFE---QDDPDLY--L

SKIKYILETDLDT-----------------------------------------SENLEL

YFVEEMYD---------------------------QSGQLQKTVELIPNG----AKVRVT

NATK--NQYLDALAQQRLCNN-----VREEIDSFLKG---------------LNGII---

--PDNLLS-IFDENELELLLCGTGE--------------------------YSIADFRAN

HIING---------------GSAEFRRVLGWFWAAVG-NFSQTE----MARLLQFTTGCS

QLPPGGFQELNPRF----QITAAPT--------------------------------FGN

LP-TAHTCFN-----QLCLPD-YESYEQ--FEKALMFAISEGTEGF

>Culex_quinquefasciatus_XM_001844029.1 .

VKVTREKILESSMKATKHFS----------------VSDWC-GNFEVTFQ-----GEQGI

---DWGGLRREWFELICSALFDP-------------------------------------

------------------------------------------------------------

------------------------------------------------------------

------------------------------------------------------------

------------------------------------------------------------

--------------------RGGLFCTFHDKRQ---------------------------

------------ALVHPNPNR----------PPHLKLKHFEFAG--KVVGKCLYESAL--

GGTYRQLVRARFSR-SFLAQLIGLR-----------------------------------

----------------------------------------VHYKYFE---QDDPDLY--L

SKIKYILDTDLDT-----------------------------------------SDNLEL

YFVEEIYD---------------------------TSGQLVKTVELIPNG----AKTRVT

NATK--NQYLDALAQQRLCNN-----VREEIDSFLKG---------------LNGII---

--PDNLLS-IFDENELELLLCGTGE--------------------------YSIADFRAN

HIVNG---------------GSPEFRRVLGWFWAAVG-NFSQTE----MARLLQFTTGCS

QLPPGGFQELNPRF----QITAAPT--------------------------------FGN

LP-TAHTCFN-----QLCLPD-YESYEQ--FERALVFATSEGTEGF

>Aedes_aegypti_XM_001651703.1 .

VKVQRDKILESSMKATKHFS----------------VSDWC-GNFEVTFQ-----GEQGI

---DWGGLRREWFELICSALFDP-------------------------------------

------------------------------------------------------------

------------------------------------------------------------

------------------------------------------------------------

------------------------------------------------------------

--------------------RGGLFCTFHDKRQ---------------------------

------------ALVHPNPNR----------PPHLKLKHFEFAG--KVVGKCLYESAL--

GGTYRQLVRARFSR-SFLAQLIGLR-----------------------------------

----------------------------------------VHYKYFE---QDDPDLY--L

SKIKYILDTDLDT-----------------------------------------SENLEL

YFVEEIYD---------------------------TSGQLVKTIELIPNG----AKIRVT

NATK--NQYLDALAQQRLCNN-----VREEIDSFLKG---------------LNGVI---

--PDNLLS-IFDENELELLLCGTGE--------------------------YSIADFRAN

HIVNG---------------GSPEFRRVLGWFWAAVG-NFSQTE----MARLLQFTTGCS

QLPPGGFQELNPRF----QITAAPT--------------------------------FGN

LP-TAHTC--------------------------------------

>Tribolium_castaneum_XM_968752.1 .

MKVNREKLLESSMKATKNFS----------------VSEWC-RNFEITFQ-----GEQGI

---DWGGLRREWFELICSQLFDA-------------------------------------

------------------------------------------------------------

------------------------------------------------------------

------------------------------------------------------------

------------------------------------------------------------

--------------------KNALFTSFHEGQQ---------------------------

------------SLVHPNPSR----------PSHLKLRHYEFAG--KVVGKCLYESAL--

GGSYRQLVRARFTR-SFLAQVIGLR-----------------------------------

----------------------------------------VHYKYFE---QDDPDLY--L

SKIKYLLENDID------------------------------------------QIDTEL

YFVEEQYD---------------------------TSGQLLKTVELIPNG----SKIRVR

NATK--LQYLDALAQYRLATS-----IKDEMEAFLKG---------------LNELI---

--PDNLLS-IFDENELELLLCGTGQ--------------------------YSIADFKQH

HVING---------------NSAEFRRIVGWFWAAVG-NFTQEE----MARLLQFTTGCS

QLPPGGFKELTPKF----QITAAPT--------------------------------FGN

LP-TAHTCFN-----QLCLPD-YDCYEH--FEKSLLLAISEGTEGF

>Nasonia_vitripennis_XM_001602558.1 .

MKVQRDKLLESSMKATKGFS----------------VSDWC-RNFEITFQ-----GEQGV

---DWGGVRREWFELICAALFDP-------------------------------------

------------------------------------------------------------

------------------------------------------------------------

------------------------------------------------------------

------------------------------------------------------------

--------------------GNGLFESFGESQQ---------------------------

------------ALVHPNSKR----------PPQLKLKHFEFAG--RIVGKCLYESAL--

GGSYRQLVRARFTR-SFLAQIIGLR-----------------------------------

----------------------------------------VHYKYFE---QDDPDLY--L

SKVKYILEND--------------------------------------------VEEMEL

FFVEEEYD---------------------------KDGQLTKLAELIPGG----SKVRVT

NDMK--LRYLDALAQHRLASS-----IRSEVDHFLRG---------------LNELI---

--PDNLLG-IFDENELELLLCGTGE--------------------------YSVTDLRAH

HIANG---------------SSPEFLRVLDWFWTAVS-NFTQEE----MARLLQFTTGCS

QLPPGGFQQLSPRF----QITAAPT--------------------------------FEN

LP-TAHTCFN-----QLCLPD-YECYDH--FEKALLLAISEGTEGF

>Apis_mellifera_XM_001121820.1 .

VKVQRDKLLESSMKSTKGFS----------------VSDWC-RNFEISFQ-----GEQGV

---DWGGVRREWFELICAALFDS-------------------------------------

------------------------------------------------------------

------------------------------------------------------------

------------------------------------------------------------

------------------------------------------------------------

--------------------GNGLFASFGESQQ---------------------------

------------ALVHPNSKR----------PSQLKLKHYEFAG--RIVGKCLYESAL--

GGSYRQLVRARFTR-SFLAQIIGLR-----------------------------------

----------------------------------------VHYKYFE---QDDPDLY--L

SKIKYILEND--------------------------------------------VEEMEL

YFVEEEYD---------------------------KDGQLLKVAELIPGG----SKIRVT

NDTK--LRYLDALAQHRLASS-----VRNEVEHFLRG---------------LNELI---

--PDNLLG-IFDENELELLLCGTGE--------------------------YSVADLRAH

HIANG---------------SSPEFLRVLDWFWTAVK-----------------------

-LPPGGFQQLSPRF----QITAAPT--------------------------------FAN

LP-TAHTCFN-----QLCLPD-YECYDH--FERALLLAISEGTEGF

>Xenopus_tropicalis_NP_001011393.1 .

LKVNRHSLLESSLRATRNFS----------------ASDWC-KNFEVVFQ-----DEEAL

---DWGGPRREWFELICKTLFDT-------------------------------------

------------------------------------------------------------

------------------------------------------------------------

------------------------------------------------------------

------------------------------------------------------------

--------------------NNQLFIRFSDSNQ---------------------------

------------GLVHPNPCR----------PPNIRVKLYEFAG--RVVGKCLFESSLG-

-GGCEQLVRARFTR-SFLAQIIGLR-----------------------------------

----------------------------------------MHYKYFE---TDDPDFFQ--

TKVQYILTND--------------------------------------------VIDTEL

SFAEEKYG---------------------------RAGQLEKVVELIPGG----SQILVT

NENK--VYYLNLLANHRLSNQ-----VREEVDHFLKG---------------LNELV---

---PDNLLGIFDENELELLMCGTGH--------------------------IAVQDFQAH

AVVIG--------------GSWHFREKVMSWFWAVVS-SLTQEE----LARLLQFTTGSS

QLPPGGFAALSPSF----QIIGSPT--------------------------------HGT

LP-TAHTCFN-----QLCLPT-YDSYEE--MHKMLKLAI-------

>Xenopus_laevis_NM_001095918.1 .

LKVNRHSLLESSLRATRNFS----------------ASDWC-KNFEVVFQ-----DEEAL

---DWGGPRREWFELICKALFDT-------------------------------------

------------------------------------------------------------

------------------------------------------------------------

------------------------------------------------------------

------------------------------------------------------------

--------------------NNQLFIRFSDSNQ---------------------------

------------SLVHPNPCR----------PPNIRVKLYEFAG--RVMGKCLFESSL--

GGGCEQLVRARFTR-SFLAQIIGLR-----------------------------------

----------------------------------------MHYKYFE---TDDPDFF--Q

SKVQYILTND--------------------------------------------VIDTEL

FFAEEKYG---------------------------RAGQLEKVVELIPGG----SQIPVT

NENK--VYYLNLLANHRLSNQ-----VREEVDHFLKG---------------LNELV---

--PDNLLG-IFDENELELLMCGTGH--------------------------IAVQDFQAH

AVVIG--------------GSWHFREKVMSWFWAVVS-SLTQEE----LARLLQFTTGSS

QLPSGGFAALSPSF----QIIGSPA--------------------------------HGT

LP-TAHTCFN-----QLCLPT-YDSYEE--MHKMLKLAISEGCEGF

>1_emb|CAG10645.1 .

LKITRHSILDSSLKATRNFS----------------VSDWS-KNFEVVFQ------DEEA

L--DWGGPRREWFELICKTLFDT-------------------------------------

------------------------------------------------------------

------------------------------------------------------------

------------------------------------------------------------

------------------------------------------------------------

--------------------SNQLFTRFSDNNQ---------------------------

------------GLVHPNADRP----------AHLRLKMYEFAG--RIVGKCLFESALG-

-GAYKQLVRARFTR-SFLAQIIGLR-----------------------------------

----------------------------------------MNYKYFE---TDDQEFYK--

TKVCFILNND--------------------------------------------VSEMDL

VFAEEKYS---------------------------KSGQLEKVVELISGG----AQIAVT

NENK--MHYLNLLAQYRLATQ-----VRDEVEHFLKV-------------RSLNELV---

---PENLLAIFDENELELLMCGTGD--------------------------INVQDFKAH

AVIVG--------------GSWHFREKVMKWFWAVVS-SFTQEE----LARLLQFTTGSS

QLPPGGFNTLCPSF----QIIAAPT--------------------------------HST

LP-TAHTCFN-----QLCLPT-YDSYEE--LHKMLKLAI-------

>XP_001921225.1 .

LKISRHSLLESSLKATRNFS----------------VSDWS-KNFEVVFQ------DEEA

L--DWGGPRREWFELVCKALFDT-------------------------------------

------------------------------------------------------------

------------------------------------------------------------

------------------------------------------------------------

------------------------------------------------------------

--------------------NNQLFTRFSDNNQ---------------------------

------------GLVHPNAERP----------PHLRVKMYEFAG--RVVGKCLYESALG-

-GSYKQLVRARFTR-SFLAQIIGLR-----------------------------------

----------------------------------------MNYKYFE---TDDEEFYK--

TKVCFILNND--------------------------------------------VSEMDL

LFAEEKYS---------------------------KSGHLEKVVELIPGG----AQIAVT

NENK--NHYLNLLAQYRLASQ-----VREEVEHFLKG---------------LNELV---

---PENLLAIFDENELELLMCGTGD--------------------------INVQDFKAH

AVIVG--------------GSWHFREKVMKWFWAVVS-SFTQEE----LARLLQFTTGSS

QLPPGGFNTLCPSF----QIIAAPT--------------------------------HST

LP-TAHTCFN-----QLCLPT-YDSYEE--LHKLLKLAI-------

>Danio_rerio_XM_001921190.1 .

LKISRHSLLESSLKATRNFS----------------VSDWS-KNFEVVFQ-----DEEAL

---DWGGPRREWFELVCKALFDT-------------------------------------

------------------------------------------------------------

------------------------------------------------------------

------------------------------------------------------------

------------------------------------------------------------

--------------------NNQLFTRFSDNNQ---------------------------

------------GLVHPNAER----------PPHLRVKMYEFAG--RVVGKCLYESAL--

GGSYKQLVRARFTR-SFLAQIIGLR-----------------------------------

----------------------------------------MNYKYFE---TDDEEFY--K

TKVCFILNND--------------------------------------------VSEMDL

LFAEEKYS---------------------------KSGHLEKVVELIPGG----AQIAVT

NENK--NHYLNLLAQYRLASQ-----VREEVEHFLKG---------------LNELV---

--PENLLA-IFDENELELLMCGTGD--------------------------INVQDFKAH

AVIVG--------------GSWHFREKVMKWFWAVVS-SFTQEE----LARLLQFTTGSS

QLPPGGFNTLCPSF----QIIAAPT--------------------------------HST

LP-TAHTCFN-----QLCLPT-YDSYEE--LHKLLKLAISEGSEGF

>Taeniopygia_guttata_XP_002200567.1 .

---------------TRNFS----------------VSDWS-KNFEVIFQ------DEEA

L--DWGGPRREWFELICKALFDT-------------------------------------

------------------------------------------------------------

------------------------------------------------------------

------------------------------------------------------------

------------------------------------------------------------

--------------------TSQLFTRFSDNNQ---------------------------

------------ALVHPNPGRP----------TYLRLKVYEFAG--RLVGKCLYESSLG-

-GAYKQLVRARFTR-SFLAQIIGLR-----------------------------------

----------------------------------------MHYKYFE---TDDPEFYK--

SKVCFILNND--------------------------------------------VSEMDL

VFAEEKYS---------------------------KTGQLEKVVELVAGG----AQVPVT

NENK--ILYLNLLAQYRLANQ-----VREEVDHFLKG---------------LNELV---

---PENLLAIFDENELELLMCGTGD--------------------------ISVCDFKAH

AVVVG--------------GSWHFREKVMRWFWTVVS-SFTQEE----LARLLQFTTGSS

QLPPGGFAALCPSF----QIIAAPT--------------------------------HST

LP-TAHTCFN-----QLCLPT-YDSYEE--VHKMLQLAI-------

>Gallus_gallus_XM_002200531.1 .

LKVSRHCLLESSFKATRNFS----------------VSDWS-KNFEVIFQ-----DEEAL

---DWGGPRREWFELICKALFDT-------------------------------------

------------------------------------------------------------

------------------------------------------------------------

------------------------------------------------------------

------------------------------------------------------------

--------------------TSQLFTRFSDNNQ---------------------------

------------ALVHPNPGR----------PTYLRLKVYEFAG--RLVGKCLYESSL--

GGAYKQLVRARFTR-SFLAQIIGLR-----------------------------------

----------------------------------------MHYKYFE---TDDPEFY--K

SKVCFILNND--------------------------------------------VSEMDL

VFAEEKYS---------------------------KTGQLEKVVELVAGG----AQVPVT

NENK--ILYLNLLAQYRLANQ-----VREEVDHFLKG---------------LNELV---

--PENLLA-IFDENELELLMCGTGD--------------------------ISVCDFKAH

AVVVG--------------GSWHFREKVMRWFWTVVS-SFTQEE----LARLLQFTTGSS

QLPPGGFAALCPSF----QIIAAPT--------------------------------HST

LP-TAHTCFN-----QLCLPT-YDSYEE--VHKMLQLAISEGCEGF

>Gallus_gallus_XM_421269.2 .

LKVSRHSLLESSFKATRNFS----------------VSDWS-KNFEVVFQ-----DEEAL

---DWGGPRREWFELICKALFDT-------------------------------------

------------------------------------------------------------

------------------------------------------------------------

------------------------------------------------------------

------------------------------------------------------------

--------------------TSQLFTRFSDNNQ---------------------------

------------ALVHPNPGR----------PTYVRLKLYEFAG--RLVGKCLYESSL--

GGAYKQLVRARFTR-SFLAQIIGLR-----------------------------------

----------------------------------------MHYKYFE---TDDPEFY--K

SKVCFILNND--------------------------------------------VSEMDL

VFAEEKYS---------------------------KTGQLEKVVELVTGG----AQVPVT

NENK--IFYLNLLAQYRLANQ-----VREEVDHFLKG---------------LNELV---

--PENLLA-IFDENELELLMCGTGD--------------------------ISVCDFKAH

AVVVG--------------GSWHFREKVMRWFWTVVS-SFTQEE----LARLLQFTTGSS

QLPPGGFAALCPSF----QIIAAPT--------------------------------HST

LP-TAHTCFN-----QLCLPT-YDSYEE--VHKMLQLAISEGCEGF

>Monodelphis_domestica_XM_001367963.1 .

LKVSRHTLLESSLKATRNFS----------------ISDWS-KNFEVIFQ-----DEEAL

---DWGGPRREWFELTCRALFDT-------------------------------------

------------------------------------------------------------

------------------------------------------------------------

------------------------------------------------------------

------------------------------------------------------------

--------------------TNQLFTRFSDNNQ---------------------------

------------ALVHPNPNR----------PPHLRLKVYEFAG--RLVGKCLYESSL--

GGAYKQLVRARFTR-SFLAQIIGLR-----------------------------------

----------------------------------------MHYKYFE---TDDPEFY--K

SKVCFILNND--------------------------------------------MSEMEL

VFAEEKYN---------------------------KSGQLDKVVELMNGG----AQVPVT

NSNK--TFYLNLLAQYRLASQ-----IKEEVEHFLKG---------------LNELV---

--PENLLA-IFDENELELLMCGTGD--------------------------INVSDFKAH

AVVVG--------------GSWHFREKVMRWFWTVVS-SLTQEE----LARLLQFTTGSS

QLPPGGFAALCPSF----QIIAAPT--------------------------------HST

LP-TAHTCFN-----QLCLPT-YDSYED--VHKMLQLAISEGCEGF

>Mus_musculus_AK147878.1 .

LKVSRHALLESSLKATRNFS----------------ISDWS-KNFEVVFQ-----DEEAL

---DWGGPRREWFELICKALFGT-------------------------------------

------------------------------------------------------------

------------------------------------------------------------

------------------------------------------------------------

------------------------------------------------------------

--------------------TSQLFARFTDSNQ---------------------------

------------ALVHPNPNR----------PAHLRLKMYEFAG--RLVGKCLYESSL--

GGAYKQLVRARFTR-SFLAQIIGLR-----------------------------------

----------------------------------------MHYKYFE---TDDPEFY--K

SKVCFILNND--------------------------------------------MSEMEL

VFAEEKYN---------------------------KSGQLDKIVELMTGG----AQTTVT

NANK--IFYLNLLAQYRLASQ-----VKEEVEHFLKG---------------LNELV---

--PENLLA-IFDENELELLMCGTGD--------------------------INVSDFKAH

AVVVG--------------GSWHFREKVMRWFWAVVS-SLTQEE----LARLLQFTTGSS

QLPPGGFAALCPSF----QIIAAPT--------------------------------HST

LP-TAHTCFN-----QLCLPT-YDSYEE--VHRMLQLAISEGCEGF

>Pan_troglodytes_AY410383.1 .

LKVSRHA----SLKATRNFS----------------ISDWS-KNFEVVFQ-----DEEAL

---DWGGPRREWFELICKALFDT-------------------------------------

------------------------------------------------------------

------------------------------------------------------------

------------------------------------------------------------

------------------------------------------------------------

--------------------TNQLFTRFSDNNQ---------------------------

------------AL------R----------PTHLRLKMYEFAG--RLVGKCLYESSL--

GGAYKQLVRARFTR-SFLAQIIGLR-----------------------------------

----------------------------------------MHYKYFE---TDDPEFY--K

SKVCFILNND--------------------------------------------MSEMEL

VFAEEKYN---------------------------KSGQLDKVVELMTGG----AQTPVT

NANK--IFYLNLLAQYRLASQ-----VKEEVEHFLKG---------------LNELV---

--PENLLA-IFDENELELLMCGTGD--------------------------ISVSDFKAH

AVVVG--------------GSWXXXXXVMRWFWTVVS-SLTQEE----LARLLQFTTGSS

QLPPGGFAALCPSF----QIIAAPT--------------------------------HST

LP-TAHTCFN-----QLCLPT-YDSYEE--VHRMLQLAISEGCEGF

>Sus_scrofa_XM_001926730.1 .

-----------------------------------------------------------L

---DWGGPRREWFELICKALFDT-------------------------------------

------------------------------------------------------------

------------------------------------------------------------

------------------------------------------------------------

------------------------------------------------------------

--------------------TNQLFTRFSDTNQ---------------------------

------------ALVHPNPNR----------PAHLRLKMYEFAG--RLVGKCLYESSL--

GGAYKQLVRARFTR-SFLAQIIGLR-----------------------------------

----------------------------------------MHYKYFE---TDDPEFY--K

SKVCFILNND--------------------------------------------MSEMEL

VFAEEKYN---------------------------KSGQLDKVVELMTGG----AQTPVT

NANK--IFYLNLLAQYRLASQ-----VKEEVEHFLKG---------------LNELV---

--PENLLA-IFDENELELLMCGTGD--------------------------ISVSDFKAH

AVVVG--------------GSWHFREKVMRWFWTVVS-SLTQEE----LARLLQFTTGSS

QLPPGGFAALCPSF----QIIAAPT--------------------------------HST

LP-TAHTCFN-----QLCLPT-YDSYEE--VHRMLQLAISEGCEGF

>Bos_taurus_NM_001076165.1 .

LKVSRHALLESSLKATRNFS----------------ISDWS-KNFEVVFQ-----DEEAL

---DWGGPRREWFELICKALFDT-------------------------------------

------------------------------------------------------------

------------------------------------------------------------

------------------------------------------------------------

------------------------------------------------------------

--------------------TNQLFTRFSDTNQ---------------------------

------------ALVHPNPNR----------PTHLRLKMYEFAG--RLVGKCLYESAL--

GGAYKQLVRARFTR-SFLAQIIGLR-----------------------------------

----------------------------------------MHYKYFE---TDDPEFY--K

SKVCFILNND--------------------------------------------MSEMEL

VFAEEKYN---------------------------KSGQLDKVVELMTGG----AQTPVT

NANK--IFYLNLLAQYRLASQ-----VKEEVEHFLKG---------------LNELV---

--PENLLA-IFDENELELLMCGTGD--------------------------ISVSDFKAH

AVVIG--------------GSWHFREKVMRWFWTVVS-SLTQEE----LARLLQFTTGSS

QLPPGGFAALCPSF----QIIAAPT--------------------------------HST

LP-TAHTCFN-----QLCLPT-YDSYEE--VHRMLQLAISEGCEGF

>Canis_familiaris_XM_547907.2 .

LKVSRHTLLESSLKATRNFS----------------ISDWS-KNFEVVFQ-----DEEAL

---DWGGPRREWFELICKALFDT-------------------------------------

------------------------------------------------------------

------------------------------------------------------------

------------------------------------------------------------

------------------------------------------------------------

--------------------TNQLFTRFSDNNQ---------------------------

------------ALVHPNPNR----------PAHLRLKMYEFAG--RLVGKCLYESSL--

GGAYKQLVRARFTR-SFLAQIIGLR-----------------------------------

----------------------------------------MHYKYFE---TDDPEFY--K

SKVCFILNND--------------------------------------------MSEMEL

VFAEEKYN---------------------------KSGQLDKVVELMTGG----AQTPVT

NANK--IFYLNLLAQYRLASQ-----VKEEVEHFLKG---------------LNELV---

--PENLLA-IFDENELELLMCGTGD--------------------------ISVSDFKAH

AVVVG--------------GSWHFREKVMRWFWTVVS-SLTQEE----LARLLQFTTGSS

QLPPGGFAALCPSF----QIIAAPT--------------------------------HST

LP-TAHTCFN-----QLCLPT-YDSYEE--VHRMLQLAISEGCEGF

>Homo_sapiens_AK296912.1 .

LKVSRHALLESSLKATRNFS----------------ISDWS-KNFEVVFQ-----DEEAL

---DWGGPRREWFELICKALFDT-------------------------------------

------------------------------------------------------------

------------------------------------------------------------

------------------------------------------------------------

------------------------------------------------------------

--------------------TNQLFTRFSDNNQ---------------------------

------------ALVHPNPNR----------PAHLRLKMYEFAG--RLVGKCLYESSL--

GGAYKQLVRARFTR-SFLAQIIGLR-----------------------------------

----------------------------------------MHYKYFE---TDDPEFY--K

SKVCFILNND--------------------------------------------MSEMEL

VFAEEKYN---------------------------KSGQLDKVVELMTGG----AQTPVT

NANK--IFYLNLLAQYRLASQ-----VKEEVEHFLKG---------------LNELV---

--PENLLA-IFDENELELLMCGTGD--------------------------ISVSDFKAH

AVVVG--------------GSWHFREKVMRWFWTVVS-SLTQEE----LARLLQFTTGSS

QLPPGGFAALCPSF----QIIAAPT--------------------------------HST

LP-TAHTCFN-----QLCLPT-YDSYEE--VHRMLQLAISEGCEGF

>Monosiga_brevicollis_XM_001743996.1 .

LVLQREGLMRDSWLAMTALP----------------VHQLQ-RRLFVSFE-----GEAGR

---DFGGLAREWMDLMCNGM----------------------------------------

------------------------------------------------------------

------------------------------------------------------------

------------------------------------------------------------

------------------------------------------------------------

--------------------SHHLPLALV-------------------------------

------------PTMTPTKEA---------------LDTIEFLG--RVVGLALFHG----

-----RLINPHFSL-IVYKMLLDQPC----------------------------------

----------------------------------------ASLDDLA---TVDPEMHH--

GYVLAICSN---------------------------------------------DVCRTD

LFSPRTVG----------------------------------------------STKPVT

DANK--RDYVDCLLQWRLNRG-----VAELLEAFKIG---------------LTSFI---

--PLSALA-GFTAPRLRLLVSG-LQQ-------------------------IDVREWRTA

TAYVN---------------GYGEDAIQVQWFWQWVD-QADEAQ----RAKLLTFCTGST

QLPAQGFHGLSGIH----GYCPFVIARVGD---------------------------VDR

YP-AAHTCVN-----RLDLPA-YASRDV--LHARLSFAVQET-EGF

>Monosiga_brevicollis_XM_001745698.1 .

--------------------------------------------LKVAFV-----GEAGI

----CLGPLRELFSVLPDSILTD-------------------------------------

------------------------------------------------------------

------------------------------------------------------------

------------------------------------------------------------

------------------------------------------------------------

--------------------TKLLTAATENNPRLV-------------------------

------------PTYFLNEHGACEA---ARPDSNARTAQFRALG--TLLGLAILHQ----

-----QTVNLPLAL-HVLAEMLSDPVE---------------------------------

----------------------------------------PNIDSLE---TLDGDLG---

RNLRWLLNRSID------------------------------------------DLDLDL

GYSVSVPP-----------------------------CAGAAPIEIELSR----DHASVT

DANK--KGYVQAVAQFHLVDK-----TSCEVRDLREG---------------FQSVL---

--PPDLLM-PFTAGELALTLEGVAT--------------------------IDVQAWQRH

TSYSN---------------EYDANHQVIRWFWQLVT-TLSDAE----KSLLLQFVTGVT

RLPPGGFADLRALG----GGSGMTITRGGD---------------------------VRH

LP-GASTCFN-----LLKLPP-YPTPAI--LRQKVLIAIRHGAHGF

>Danio_rerio_CU928229.4 .

------------------------------------------------------------

------GVYRECIVSLCKEGFAA-------------------------------------

------------------------------------------------------------

------------------------------------------------------------

------------------------------------------------------------

------------------------------------------------------------

--------------------EYGLFRQTTD------------------------------

------------GYVFPNSFSAIA------AGDPQHLQKIRFLG--AMVGRALRDG----

-----VLQDVPFAQ-HFRNAILGRR-----------------------------------

----------------------------------------NTLSNLK---SFDSELY---

HQLMSLTLLDEQELQ---------------------------------------AIGLTF

VYTVNSLG-------------------------------VTKEVELVRGG----AQMEVT

PRNC--LYYVHLVADFKLNRE-----AADQTRAFCAG---------------LHSVL---

--DSNRLV-FFDSNEVGKLFGGDESGG------------------------IDLQDWKAN

TVYDS---------------ADDAEKPQVRLFWDVVE-SLTRKQ----QSQLLKFATSMT

RPPLLGFSFLSPPF----KLQLLSPNVSG----------------------------GDH

LP-SAATCFS-----TLKLPP-YGDYAT--ARAKIIAAIEET-GTF

>Monosiga_brevicollis_ABFJ01001529.1 .

--------------------------------------------------------EAGV

---DGGGLFREFLQLICREGFDP-------------------------------------

------------------------------------------------------------

------------------------------------------------------------

------------------------------------------------------------

------------------------------------------------------------

--------------------TRGFFLESSS------------------------------

------------RELYPNPDMP--------LLDPDADRHYKFLG--TIVGRALRAE----

-----MLVDLTFCN-FFLTQILGGR-----------------------------------

----------------------------------------VSLNELR---DLDAELY---

QNLVQVKDYAGD------------------------------------------VSELDL

TFTVAGSE-----------------------------VTGHRLYNLVPDG----ANVAVT

NENR--IRYVYHMADFYLRRR-----TRQQIVAFQQG---------------LEQAV---

--PAALLR-LFAPAELRKLIRGEKQV-------------------------IDVEEFRRH

VVYNG----------------FRADDVPIQLFWSVVH-EMDNDA----REQLLRFITSCP

RPPILGFQAMHPRI----AIANSQD--------------------------------PSR

LP-SAATCMN-----LLKLPP-YTSREI--LKDRLYKAIYET-EGF

>Drosophila_mojavensis_XM_002009004.1 .

IVIHRERIVEDGYRQLAAQP----------------THALK-GVIRVRFINQQGLHEAGI

---DQDGVFKEFLEETIKKVFDP-------------------------------------

------------------------------------------------------------

------------------------------------------------------------

------------------------------------------------------------

------------------------------------------------------------

--------------------SLNLFKTTSD------------------------------

------------QRLYPSPIS---------YVQDNHLQLFEFVG--RMLGKAVYEG----

-----IVVDVPFAS-FFLSQLLGQTQQAL-------------------------------

---------------------------------------YSCMDELP---SLDNELY---

RSLTFIKHYKQD------------------------------------------VADLNL

TFSVDQDV-----------------------------MGKIVTHELHPGG----KARVVN

DHNK--LVYIHYMAYFHMNTQ-----IREQTQAFNRG---------------FRSIV---

--NPEWLS-LFSPPELQRLISGDTVP-------------------------LDLRDLRKH

TQYYG---------------GFHDSHRVVGWLWDILAKDFTEEE----RKLFLKFVTSCS

KPPLLGFAHLEPPF----SIRCVEVGDDEDTGDTIGSVIRGFFTIRKKDP-------LNR

LP-TSSTCFN-----LLKLPN-YQKKST--LRDKLRYAVSSN-TGF

>Drosophila_virilis_XM_002047828.1 .

IVIHRERIVEDGYRQLVAQP----------------THALK-GVIRVRFINQQGLHEAGI

---DQDGVFKEFLEETIKKVFDP-------------------------------------

------------------------------------------------------------

------------------------------------------------------------

------------------------------------------------------------

------------------------------------------------------------

--------------------SLNLFKTTSD------------------------------

------------QRLYPSPIS---------YVQDNHLQLFEFVG--RMLGKAVYEG----

-----IVVDVPFAS-FFLSQLLGQTQQAL-------------------------------

---------------------------------------YSCMDELP---SLDNELY---

RSLTFIKHYKQD------------------------------------------VADLNL

TFSVDQDV-----------------------------MGKIVTHELHPGG----KARVVN

DHNK--LVYIHYMAYFHMNTQ-----IREQTQAFNRG---------------FRSIV---

--NPEWLS-LFSPPELQRLISGDTVP-------------------------LDLKDLRKH

TQYYG---------------GFHDSHRVVGWLWDILAKDFTEDE----RKLFLKFVTSCS

KPPLLGFAHLEPPF----SIRCVEVGDDEDTGDTIGSVIRGFFTIRKKDP-------LNR

LP-TSSTCFN-----LLKLPN-YQKKST--LRDKLRYAVSSN-TAL

>Drosophila_ananassae_XM_001956806.1 .

IVIHRERIVEDGYRQLAAQP----------------TQALK-GVIRVRFINQQGLHEAGI

---DQDGVFKEFLEETIKKVFDP-------------------------------------

------------------------------------------------------------

------------------------------------------------------------

------------------------------------------------------------

------------------------------------------------------------

--------------------SLNLFKTTSD------------------------------

------------QRLYPSPIS---------YVQDNHKELFEFVG--RMLGKAVYEG----

-----IVVDVPFAS-FFLSQLLGQTQQAP-------------------------------

---------------------------------------YSCMDELP---SLDNELY---

RSLTFIKHYKQD------------------------------------------VADLNL

TFSVDQDV-----------------------------MGRIVTHELHPGG----KARVVN

DHNK--LVYIHYMAFFHMNTQ-----IREQTIAFNRG---------------FRSIV---

--NPEWLS-LFSPPELQRLISGDTVP-------------------------LDLRDLRKH

TQYYG---------------GFHDSHRVVGWLWDILAKDFTEEE----RKLFLKFVTSCS

KPPLLGFAHLEPPF----SIRCVEVGDDEDTGDTIGSVIRGFFTIRKKDP-------LNR

LP-TSSTCFN-----LLKLPN-YQKKST--LRDKLRYAVSSN-TGF

>Drosophila_persimilis_XM_002028008.1 .

IVIHRERIVEDGYRQLAAQP----------------TQALK-GVIRVRFINQQGLHEAGI

---DQDGVFKEFLEETIKKVFDP-------------------------------------

------------------------------------------------------------

------------------------------------------------------------

------------------------------------------------------------

------------------------------------------------------------

--------------------SLNLFKTTSD------------------------------

------------QRLYPSPIS---------YVQDNHLQLFEFVG--RMLGKAVYEG----

-----IVVDVPFAS-FFLSQLLGQTQQAL-------------------------------

---------------------------------------YSCMDELP---SLDAELY---

RSLTFIKHYKQD------------------------------------------VAELNL

TFSVDQDV-----------------------------MGKIVTHELHPGG----KGRVVN

DHNK--LVYIHYMAYFHMNTQ-----IREQTQAFNRG---------------FRSIV---

--NPEWLS-LFSPPELQRLISGDTAP-------------------------LDLRDLRKH

TQYYG---------------GFHDSHRVVGWLWDILGKDFTEEE----RKLFLKFVTSCS

KPPLLGFAHLEPPF----SIRCVEVGDDEDTGDTIGSVIRGFFTIRKKDP-------LNR

LP-TSSTCFN-----LLKLPN-YQKKST--LRDKLRYAVSSN-TGF

>Drosophila_melanogaster_NM_140022.1 .

IVIHRDRIVEDGYRQLAAQP----------------TQALK-GVIRVRFINQQGLHEAGI

---DQDGVFKEFLEETIKKVFDP-------------------------------------

------------------------------------------------------------

------------------------------------------------------------

------------------------------------------------------------

------------------------------------------------------------

--------------------SLNLFKTTSD------------------------------

------------QRLYPSPIS---------YVQDNHLELFEFVG--RMLGKAVYEG----

-----IVVDVPFAS-FFLSQLLGQTQQAL-------------------------------

---------------------------------------YSCMDELP---SLDNELY---

RSLTFIKHYKQD------------------------------------------VSDLNL

TFSVDQDV-----------------------------MGKIVTLALHPGG----KARVVN

DHNK--LVYIHYMAFFHMNTQ-----IREQTIAFNRG---------------FRSIV---

--NPEWLS-LFSPPELQRLISGDTSP-------------------------LDLKDLQKH

THYYG---------------GFHDTHQVVCWLWDILAKDFTEEE----RKLFLKFVTSCS

KPPLLGFAHLEPPF----SIRCVEVSDDEDTGDTIGSVIRGFFAIRKKDP-------LNR

LP-TSSTCFN-----LLKLPN-YQKKST--LRDKLRYAVSSN-TGF

>Drosophila_erecta_AAPQ01006547.1 .

IVIHRDRIVEDGYRQLAAQP----------------TQALK-GVIRVRFINQQGLHEAGI

---DQDGVFKEFLEETIKKVFDP-------------------------------------

------------------------------------------------------------

------------------------------------------------------------

------------------------------------------------------------

------------------------------------------------------------

--------------------SLNLFKTTSD------------------------------

------------QRLYPSPIS---------YVQDNHLQLFEFVG--RMLGKAVYEG----

-----IVVDVPFAS-FFLSQLLGQTQQAL-------------------------------

---------------------------------------YSCMDELP---SLDNELY---

RSLTFIKHYKQD------------------------------------------VSDLNL

TFSVDQDV-----------------------------MGKIVTLALHPGG----KSRVVN

DHNK--LVYIHYMAFFHMNTQ-----IREQTIAFNRG---------------FRSIV---

--NPEWLS-LFSPPELQRLISGDTSP-------------------------LDLKDLRKH

THYYG---------------GFHDSHRVVGWLWDILAKDFTEEE----RKLFLKFVTSCS

KPPLLGFAHLEPPF----SIRCVEVSDDEDTGDTIGSVIRGFFTIRKKDP-------LNR

LP-TSSTCFN-----LLKLPN-YQKKST--LRDKLRYAVSSN-TGF

>Drosophila_sechellia_XM_002029779.1 .

IVIHRDRIVEDGYRQLAAQP----------------TQALK-GVIRVRFINQQGLHEAGI

---DQDGVFKEFLEETIKKVFDP-------------------------------------

------------------------------------------------------------

------------------------------------------------------------

------------------------------------------------------------

------------------------------------------------------------

--------------------SLNLFKTTSD------------------------------

------------QRLYPSPIS---------YVQDNHLQLFEFVG--RMLGKAVYEG----

-----IVVDVPFAS-FFLSQLLGQTQQAL-------------------------------

---------------------------------------YSCMDELP---SLDNELY---

RSLTFIKHYKQD------------------------------------------VSDLNL

TFSVDQDV-----------------------------MGKIVTLALHPGG----KSRVVN

DHNK--LVYIHYMAFFHMNTQ-----IREQTIAFNRG---------------FRSII---

--NPEWLS-LFSPPELQRLISGDTSP-------------------------LDLKDLQKH

THYYG---------------GFHDSHCVVVWLWDILAKDFTEEE----RKLFLKFVTSCS

KPPLLGFAHLEPPF----SIRCVEVGDDEDTGDTIGSVIRGFFAIRKKDP-------LNR

LP-TSSTCFN-----LLKLPN-YQKKST--LRDKLRYAVSSN-TGF

>Aedes_aegypti_XM_001649776.1 .

VTVHRDRIVEDGYRQLAALP----------------PHALK-GVIRVRFVNQQGLDEAGI

---DQDGVFKEFLEETIKRVFDP-------------------------------------

------------------------------------------------------------

------------------------------------------------------------

------------------------------------------------------------

------------------------------------------------------------

--------------------SLNLFKTTTE------------------------------

------------QRLYPSPTS---------HMQENHLQLFEFVG--RMLGKAVYEG----

-----IVVDVPFAS-FFLSQVLGQTNQAL-------------------------------

---------------------------------------YSCMDELP---SLDKELY---

RSLTFIKHYHGD------------------------------------------VADLDL

TFSVDEDV-----------------------------MGKIVTHELYPGG----KARAVN

NDNK--INYIHYMAYFRMHTQ-----IRDQTAAFIRG---------------FRSIV---

--NPDWLA-LFSTPELQRLISGDTAP-------------------------LDLKDLRKH

TQYYG---------------GFHDGHRVVGWLWDILAKDFSEEE----KKLFLKFVTSCS

KPPLLGFAHLEPPF----SIRCVEVGDDEDIGDTVGSVIRGFFTIRKKDP-------LNR

LP-TSSTCFN-----LLKLPN-YQKKSM--LRDKLRYAISSN-TGF

>Anopheles_gambiae_XM_315926.3 .

VTVHRDRIVEDGYRQLAALP----------------PHGLK-GVIRVRFINQQGLDEAGI

---DQDGVFKEFLEETIKRVFDP-------------------------------------

------------------------------------------------------------

------------------------------------------------------------

------------------------------------------------------------

------------------------------------------------------------

--------------------SLNLFKTTTE------------------------------

------------QRLYPSPTS---------HMQENHLALFEFVG--RMLGKAVYEG----

-----IVVDVPFAS-FFLSQVLGQTQQAL-------------------------------

---------------------------------------YSCMDELP---SLDKELY---

RSLTFIKHYQGD------------------------------------------VADLDL

TFSVDEDV-----------------------------MGKIVTHELHPGG----RARAVN

NDNK--INYIHYMAYFRMHTQ-----IRDQTAAFIRG---------------FRCIV---

--NPDWLA-LFSTPELQRLISGDTSP-------------------------LDLKDLRKH

TQYYG---------------GFHDGHRVVGWLWDILAKDFTEEE----KKLFLKFVTSCS

KPPLLGFAHLEPPF----SIRCVEVGDDEDIGDTVGSVIRGFFTIRKKDP-------LNR

LP-TSSTCFN-----LLKLPN-YQKKSM--LRDKLRYAISSN-TGF

>Tribolium_castaneum_XM_965656.1 .

ITVHRSRIVEDGYRQLALLP----------------PQALK-GVIRVRFINEQGLDEAGI

---DQDGVFKEFLEESIKRIFDP-------------------------------------

------------------------------------------------------------

------------------------------------------------------------

------------------------------------------------------------

------------------------------------------------------------

--------------------SLNLFKVTSE------------------------------

------------ERLYPSPTS---------YLQDNHLQLFEFVG--RMLGKAVYEG----

-----IVVDVPFAS-FFLSQVSGQTAQAL-------------------------------

---------------------------------------YSCVDELP---SLDPELY---

RSLSYVKHYDGD------------------------------------------VSDLDL

TFSLDEEC-----------------------------MGKLVTHELVAGG----KAVPVT

NENK--INYIHLMAHFRMHVQ-----IKDQTSAFIRG---------------FRSII---

--NPDWLS-LFSTPELQRLISGDNVP-------------------------LDMKDLRKH

TQYYG---------------GFHDSHRVVGWLWDILEKDFTEEE----KGMFLKFVTSCS

KPPLLGFAHLEPPF----SIRCVEVGDDEDTGDTIGSVFRGFFTIRKKDP-------QNR

LP-TSSTCFN-----LLKLPN-YQKKST--LREKLRYAVTCN-TGF

>Apis_mellifera_XM_392283.3 .

IVVHRTRIVEDGYRQLAMLP----------------SQALK-GVIRVRFVNEQGLAEAGI

---DQDGVFKEFLEETIKKVFDP-------------------------------------

------------------------------------------------------------

------------------------------------------------------------

------------------------------------------------------------

------------------------------------------------------------

--------------------SLNLFKVTSE------------------------------

------------NRLYPSPTS---------SMQDNHLQLFEFVG--RMLGKAVYEG----

-----IVVDVPFAS-FFVSQFSGQTGGAL-------------------------------

---------------------------------------YSWLDELA---SLDRDLY---

RSLTLVKHYKGD------------------------------------------VRQLEL

TFSLDEDV-----------------------------LGKLVTHELIPGG----RAVPVT

NENK--INYIHLMAHFRMHMQ-----IKDQTAAFIKG---------------FRSII---

--NPEWLA-LFSTPELQRLISGDNVP-------------------------LDLRDLRRH

TQYYG---------------GFHDSHRVVCWLWDILEKDFSEEE----RGLFLKFVTSCS

KSPLLGFAHLEPPF----SIRCVEVGDDEDTGDTIGSVIRGFFTIRKKDP-------QNR

LP-TSSTCFN-----LLKLPN-YQKKST--LREKLRYAVTSN-TGF

>Strong._purpuratus_XM_781794.2 .

ITVHRSRLVEDGYQQLASVA----------------SQALK-GCIRVNFINEQGLDEAGI

---DQDGVFKEFLEEIIKTVFNP-------------------------------------

------------------------------------------------------------

------------------------------------------------------------

------------------------------------------------------------

------------------------------------------------------------

--------------------SLNLFKTTEE------------------------------

------------QRLYPSPTS---------YINENHLPLFEFVG--KMLGKAVYEG----

-----IVVEVPFAH-FFLSQILDHTHSTL-------------------------------

---------------------------------------YSPIDELP---SLDKELY---

KNLTFVKHYDGD------------------------------------------VADLDL

GFSLDEDF-----------------------------MGKLITHELIPGG----KAITVN

NQNK--IKYIHLMAHFRMCQQ-----IRDQTRAFKRG---------------FLSLV---

--NPLWLA-WFSGPELQRLISGDVNA-------------------------LDLNDLRKH

TQYYG---------------GFHNSHRVINWLFDILSNDFTFEE----RGLFLKFVTSCS

KPPLLGFAHLEPPF----SIRCVEVSDDQDTGDTVGSVLRGFLRIQRRDP-------VSR

LP-TSSTCFN-----LLKLPN-YQKKST--LKEKLRYAITSN-TGF

>Branchiostoma_floridae_XM_002220349.1 .

ITVHRSRMVEDGFQQLALLP----------------PQALK-GIIRVKFVNDLGLDEAGI

---DQDGVFKEFLEEIIKRVFDP-------------------------------------

------------------------------------------------------------

------------------------------------------------------------

------------------------------------------------------------

------------------------------------------------------------

--------------------SLNLFKMTSGE-----------------------------

------------ERLYPSPTS---------YIQEDHLTLFEFVG--KMLGKAVYEG----

-----IVVEVPFAS-FFLSQVLSHHHSAL-------------------------------

---------------------------------------YSSIDELP---SLDPELY---

KSLTFIKHYIEGD-----------------------------------------VRDLEL

TFSYAEDV-----------------------------MGKVVTHELMPGG----RAISVT

NDNK--IAYVHRVAHFRMHTQ-----IREQTAAFIRG---------------FRSII---

--SPDWLM-MFAAPELQKLISGDNME-------------------------IDLEDLKKY

TQYYG---------------GFHPAHRVVIWLWDILAKDFTNDE----RKAFLKFVTSCS

KPPLLGFAHLEPPF----CIRCVEVADDQDTGDTVGSVLRGFFTIRKRDP-------VNR

LP-TSSTCFN-----LLKLPN-YQKKST--LKEKLRYAISMN-TGF

>Danio_rerio_NM_001114682.1 .

ITIRRSRMLEDGYDQLRRLP----------------VNSIK-GVIRVKFVNDLGVDEAGI

---DQDGVFKEFLEEIIKKVFNP-------------------------------------

------------------------------------------------------------

------------------------------------------------------------

------------------------------------------------------------

------------------------------------------------------------

--------------------ALNLFKTTSGN-----------------------------

------------ERLYPSPTS---------SIHENHLQLFEFVG--KMLGKAMYEG----

-----IVVDVPFAS-FFLSQVLGHHHSTF-------------------------------

---------------------------------------YSSIDELP---SLDSEFY---

KNLTSIKRYDGD------------------------------------------VSDLGL

TLSYDEEV-----------------------------MGQLVCHELIPGG----KTMPVT

NENK--ISYIHLMAHFRMHTQ-----IKEQTAAFIRG---------------FRSII---

--NPEWLH-MFSTPEVQRLISGDNAE-------------------------IDLDDLKKH

TVYYG---------------GFHSSHRVILWLWDILSSDFTPDE----RAMFLKFVTSCS

RPPLLGFAYLKPPF----SIRCVEVSDDQDTGDTLGSVLRGFFTIRKKEP-------GGR

LP-TSSTCFN-----LLKLPN-YSKKSI--LRDKLRYAISMN-TGF

>Xenopus_tropicalis_NM_001079407.1 .

ITIRRSRMLEDGYDQLRQLS----------------QNAMK-GVIRVKFVNDLGVDEAGI

---DQDGVFKEFLEEIIKRVFDP-------------------------------------

------------------------------------------------------------

------------------------------------------------------------

------------------------------------------------------------

------------------------------------------------------------

--------------------ALNLFKTTSSG-----------------------------

------------DRLYPSPTS---------YIHENYLQLFEFVG--KMLGKAVYEG----

-----IVVDVPFAS-FFLSQLLGHHHSIF-------------------------------

---------------------------------------YSSVDELP---SLDSEFY---

KNLTSIKRYDGD------------------------------------------ISDLGL

TLSYDEDV-----------------------------MGQLVCHELVPGG----KSIPVT

NENK--ISYIHLMAHFRMHTQ-----IKNQTVAFISG---------------FRSII---

--RPEWIR-MFSAPELQRLISGDNAE-------------------------IDLDDLKKH

TVYYG---------------GFHGSHRVIIWLWDILANDFSSEE----KAMFLKFVTSCS

RPPLLGFAYLKPPF----SIRCVEVSDDQDTGDTLGSVLRGFFTIRKKEP-------GGR

LP-TSSTCFN-----LLKLPN-YSKKGI--LREKLRYAISMN-TGF

>Gallus_gallus_XM_423951.2 .

ITIRRSRMLEDGYEQLRQLS----------------QNAMK-GVIRVKFVNDLGVDEAGI

---DQDGVFKEFLEEIIKKVFDP-------------------------------------

------------------------------------------------------------

------------------------------------------------------------

------------------------------------------------------------

------------------------------------------------------------

--------------------ALNLFKTTSGD-----------------------------

------------ERLYPSPTS---------YIHENYLQLFEFVG--KMLGKAVYEG----

-----IVVDVPFAS-FFLSQLLGHHHSVF-------------------------------

---------------------------------------YSSVDELP---SLDSEFY---

KNLTSIKRYDGD------------------------------------------ISDLGL

TLSYDEDV-----------------------------MGQLVCHELVPGG----KTIPVT

NENK--ISYIHLMAHFRMHTQ-----IKSQTAALISG---------------FRSII---

--KPEWIR-MFSAPELQRLISGDNAE-------------------------IDLEDLKKH

TVYYG---------------GFHGSHRVIIWLWDILANDFSPEE----RAMFLKFVTSCS

RPPLLGFAYLKPPF----SIRCVEVSDDQDTGDTLGSVLRGFFTIRKKEP-------GGR

LP-TSSTCFN-----LLKLPN-YSKKSI--LREKLRYAISMN-TGF

>Monodelphis_domestica_XM_001378470.1 .

ITIRRSRMLEDGYEQLRQLS----------------QNAMK-GVIRVKFVNDLGVDEAGI

---DQDGVFKEFLEEIIKRVFDP-------------------------------------

------------------------------------------------------------

------------------------------------------------------------

------------------------------------------------------------

------------------------------------------------------------

--------------------ALNLFKTTSGD-----------------------------

------------ERLYPSPTS---------YIHENYLQLFEFVG--KMLGKAVYEG----

-----IVVDVPFAS-FFLSQLLGHHHSIF-------------------------------

---------------------------------------YSSVDELP---SLDAEFY---

KNLTSIKRYDGD------------------------------------------VSDLGL

TLSYDEDV-----------------------------MGQLVCHELVPGG----KTIPVT

NENK--ISYIHLMAHFRMHTQ-----IKSQTASLISG---------------FRSII---

--KWEWIR-MFSTPELQRLISGDNAE-------------------------IDLEDLKKH

TVYYG---------------GFHGSHRVIIWLWDILANDFTPEE----RAMFLKFVTSCS

RPPLLGFAYLKPPF----SIRCVEVSDDQDTGDTLGSVLRGFFTIRKKEP-------GGR

LP-TSSTCFN-----LLKLPN-YSKKSI--LREKLRYAISMN-TGF

>Canis_familiaris_XM_859027.1 .

ITIRRSRMLEDGYEQLRQLS----------------QHAMK-GVIRVKFVNDLGVDEAGI

---DQDGVFKEFLEEIIKRVFDP-------------------------------------

------------------------------------------------------------

------------------------------------------------------------

------------------------------------------------------------

------------------------------------------------------------

--------------------ALNLFKTTSGD-----------------------------

------------ERLYPSPTS---------YIHENYLQLFEFVG--KMLGKAVYER----

------------------------------------------------------------

------------------------------------------------------------

--------YDGD------------------------------------------IADLGL

TLSYDEDV-----------------------------MGQLVCHELIPGG----KTIPVT

NENK--ISYIHLMAHFRMHTQ-----IKNQTAALISG---------------FRSII---

--KPEWTR-MFSTPELQRLISGDNAE-------------------------IDLEDLKKH

TVYYG---------------GFHGSHRVIIWLWDILASDFTPDE----RAMFLKFVTSCS

RPPLLGFAYLKPPF----SIRCVEVSDDQDTGDTLGSVLRGFFTIRKREP-------GGR

LP-TSSTCFN-----LLKLPN-YSKKSV--LREKLRYAISMN-TGF

>Pan_troglodytes_XM_001137003.1 .

ITIRRSRMLEVSLAEWVSLQ----------------EHSVC-GVIRLKFVNDLGVDEAGI

---DQDGVFKEFLEEIIKRVFDP-------------------------------------

------------------------------------------------------------

------------------------------------------------------------

------------------------------------------------------------

------------------------------------------------------------

--------------------ALNLFKTTSGD-----------------------------

------------ERLYPSPTS---------YIHENYLQLFEFVG--KMLGKAVYEG----

-----IVVDVPFAS-FFLSQLLGHHHSVF-------------------------------

---------------------------------------YSSVDELP---SLDSEFY---

KNLTSIKRYDGD------------------------------------------IADLGL

TLSYDEDV-----------------------------MGQLVCHELIPGG----KTIPVT

NENK--ISYIHLMAHFRMHTQ-----IKNQTAALISG---------------FRSII---

--KPEWIR-MFSTPELQRLISGDNAE-------------------------IDLEDLKKH

TVYYG---------------GFHGSHRVIIWLWDILASDFTPDE----RAMFLKFVTSCS

RPPLLGFAYLKPPF----SIRCVEVSDDQDTGDTLGSVLRGFFTIRKREP-------GGR

LP-TSSTCFN-----LLKLPN-YSKKSV--LREKLRYAISMN-TGF

>Canis_familiaris_XM_859007.1 .

ITIRRSRMLEDGYEQLRQLS----------------QHAMK-GVIRVKFVNDLGVDEAGI

---DQDGVFKEFLEEIIKRVFDP-------------------------------------

------------------------------------------------------------

------------------------------------------------------------

------------------------------------------------------------

------------------------------------------------------------

--------------------ALNLFKTTSGD-----------------------------

------------ERLYPSPTS---------YIHENYLQLFEFVG--KMLGKAVYEG----

-----IVVDVPFAS-FFLSQLLGHHHSIF-------------------------------

---------------------------------------YSSVDELP---SLDSEFY---

KNLTSIKRYDGD------------------------------------------IADLGL

TLSYDEDV-----------------------------MGQLVCHELIPGG----KTIPVT

NENK--ISYIHLMAHFRMHTQ-----IKNQTAALISG---------------FRSII---

--KPEWTR-MFSTPELQRLISGDNAE-------------------------IDLEDLKKH

TVYYG---------------GFHGSHRVIIWLWDILASDFTPDE----RAMFLKFVTSCS

RPPLLGFAYLKPPF----SIRCVE-----------------------REP-------GGR

LP-TSSTCFN-----LLKLPN-YSKKSV--LREKLRYAISMN-TGF

>Homo_sapiens_BC144684.1 .

ITIRRSRMLEDGYEQLRQLS----------------QHAMK-GVIRVKFVNDLGVDEAGI

---DQDGVFKEFLEEIIKRVFDP-------------------------------------

------------------------------------------------------------

------------------------------------------------------------

------------------------------------------------------------

------------------------------------------------------------

--------------------ALNLFKTTSGD-----------------------------

------------ERLYPSPTS---------YIHENYLQLFEFVG--KMLGKAVYEG----

-----IVVDVPFAS-FFLSQLLGHHHSVF-------------------------------

---------------------------------------YSSVDELP---SLDSEFY---

KNLTSIKRYDGD------------------------------------------ITDLGL

TLSYDEDV-----------------------------MGQLVCHELIPGG----KTIPVT

NENK--ISYIHLMAHFRMHTQ-----IKNQTAALISG---------------FRSII---

--KPEWIR-MFSTPELQRLISGDNAE-------------------------IDLEDLKKH

TVYYG---------------GFHGSHRVIIWLWDILASDFTPDE----RAMFLKFVTSCS

RPPLLGFAYLKPPF----SIRCVEVSDDQDTGDTLGSVLRGFFTIRKREP-------GGR

LP-TSSTCFN-----LLKLPN-YSKKSV--LREKLRYAISMN-TGF

>Mus_musculus_BC026415.1 .

ITIRRSRMLEDGYEQLRQLS----------------QHAMK-GVIRVKFVNDLGVDEAGI

---DQDGVFKEFLEEIIKRVFDP-------------------------------------

------------------------------------------------------------

------------------------------------------------------------

------------------------------------------------------------

------------------------------------------------------------

--------------------ALNLFKTTSGD-----------------------------

------------ERLYPSPTS---------YIHENYLQLFEFVG--KMLGKAVYEG----

-----IVVDVPFAS-FFLSQMLGHHHSVF-------------------------------

---------------------------------------YSSVDELP---SLDSEFY---

KNLTSIKRYDGD------------------------------------------IADLGL

TLSYDEDV-----------------------------MGQLVCHELVPGG----KTIPVT

DENK--ISYIHLMAHFRMHTQ-----IKNQTAALISG---------------FRSII---

--KPEWIR-MFSTPELQRLISGDNAE-------------------------IDLEDLKKH

TVYYG---------------GFHGSHRVIIWLWDILASDFTPEE----RAMFLKFVTSCS

RPPLLGFAYLKPPF----SIRCVEVSDDQDTGDTLGSVLRGFFTIRKREP-------GGR

LP-TSSTCFN-----LLKLPN-YSKKSV--LREKLRYAISMN-TGF

>Rattus_norvegicus_NM_001143894.1 .

ITIRRSRMLEDGYEQLRQLP----------------QHAMK-GAIRVKFVSDLGVDEAGI

---DQDGVFKEFLEEIIKRVFDP-------------------------------------

------------------------------------------------------------

------------------------------------------------------------

------------------------------------------------------------

------------------------------------------------------------

--------------------ALNLFKTTSGD-----------------------------

------------ERLYPSPTS---------YIHENYLQLFEFVG--KMLGKAVYEG----

-----IVVDVPFAS-FFLSQMLGHHHSVF-------------------------------

---------------------------------------YSSVDELP---SLDSEFY---

KNLTSIKRYDGD------------------------------------------VADLGL

TLSYDEDV-----------------------------MGQLVCHELVPGG----KTIPVT

DENK--ISYIHLMAHFRMHTQ-----IKNQTAALISG---------------FRSII---

--KPEWIR-MFSTPELQRLISGDNAE-------------------------IDLEDLKKH

TVYYG---------------GFHGSHRVIVWLWDILASDFTPGE----RAMFLKFVTSCS

RPPLLGFAYLKPPF----SIRCVEVSDDQDTGDTLGSVLRGFFTIRKREP-------GGR

LP-TSSTCFN-----LLKLPN-YSKKSV--LREKLRYAISMN-TGF

>Nematostella_vectensis_XM_001639412.1 .

ITAHRNRLLEDGYDQLSVLP----------------AHILK-GLIRVRFINEQGLPEAGI

---DQDGVFKEFLEETIKHAFDP-------------------------------------

------------------------------------------------------------

------------------------------------------------------------

------------------------------------------------------------

------------------------------------------------------------

--------------------AFNLFKVTTGEE----------------------------

------------QRLYPSPTS---------FIHDNHLLLYEFVG--KMLGKAVYEG----

-----IVVEVPFAF-FFLNHVLGNQHSNL-------------------------------

---------------------------------------YSSIDELP---SLDQELY---

KSLTFIKHYDGD------------------------------------------IRDLDL

TFSFDEDV-----------------------------LGKVVTHQLKPGG----TAIVVS

DENK--ISYVHLMAHYRMCIQ-----IREQTRAFIRG---------------FKSIV---

--HNDWLR-MFSAPELQRLISGDNTA-------------------------LDLSDLRVH

TRYYG---------------GYHGGHRVVVWLWEILDKEFNDQE----KSLFLKFVTSCS

KPPLLGFEHLEPPF----SIRCVECPDDEDDGDTVGSVLRGFLNIRRRNARET----VSR

LP-TASTCFN-----LLKLPN-YRKKST--LRDKLRYAIQCN-AGF

>Acyrthosiphon_pisum_XM_001950454.1 .

---------------VSELP----------------LRRFK-GVIRVKFINEQGLDEAGI

---DQDGVFKEFLEETIKRVFDP-------------------------------------

------------------------------------------------------------

------------------------------------------------------------

------------------------------------------------------------

------------------------------------------------------------

--------------------SLNLFRTTDE------------------------------

------------QRLYPSPSS---------HIQENHLNLFEFVG--RILGKAVYEG----

-----IVVDVPFAS-FFLSQLLGQTQELL-------------------------------

---------------------------------------YSSMDELP---SLDNELY---

RNLTSVKHYEGD------------------------------------------VSDLDL

TFSVVDNH-----------------------------LGELTTHDLIPGG----RVISVT

NQNK--INYVHLMAHYVMHTQ-----IKAQTAAFIKG---------------FKYVI---

--NPEWLS-LFSTPELQKLISGDNAP-------------------------IDLLDLRRT

TQYYG---------------GFHDSHRVILWLWEVLQKDFTEKE----RSLFLKFVTSCS

KPPLLGFAYLEPPF----SIRCVQVADDEDFGDTLVSVIRGFFTIRKKDP-------NLR

LP-SASTCFN-----LLKLPN-YQKKSI--LRDKLRYAISSN-TGF

>Hydra_magnipapillata_XM_002164353.1 .

ISINRSHLLENGYEQLCRYT----------------GTQLK-GIIRVKFVNEHGLDEVGI

---DQDGVFKEFLEDVITEAFNP-------------------------------------

------------------------------------------------------------

------------------------------------------------------------

------------------------------------------------------------

------------------------------------------------------------

--------------------ELNLFKMTCGEE----------------------------

------------QRLYPSPTS---------FLHENHLQLFEFVG--KMLGKALYEG----

-----ILVDVPFAS-FFLNYMLQHKHSPM-------------------------------

---------------------------------------YSSIDELP---SLDPEMY---

KNLNFIKTYNGD------------------------------------------VSDLEL

VFAFDEDV-----------------------------LGQMVTHDLKPGG----RFVSVT

NSNK--MTYIHLMARYRLHTQ-----IKQQSAAFVQG---------------FLSII---

--SPEWLR-IFSAPQLQRLISGDTEN-------------------------FDLADLKNY

TRYYG---------------GYHASHQTIVWLWDILSNDFTLQE----KKLFLKFVTSCS

NPPLLGFKHLEPPF----SIRFVDSSDEDDNGDTPTSIVRGLFSLRKRGTTS-----SAR

LP-TSSTCFN-----LLKLPC-YKKKSI--LKEKLKYAIKSG-AGF

>Brugia_malayi_XM_001894625.1 .

ITVERNRLLADGYRQLSLLS----------------PTALK-ATIRVKFINQQGLDEAGI

---DQDGVFKEFLELTLKHVFNP-------------------------------------

------------------------------------------------------------

------------------------------------------------------------

------------------------------------------------------------

------------------------------------------------------------

--------------------DLNLFKCSPN------------------------------

------------RQLYPSPTS---------DLHENHLDLFQFVG--RMLAKAVYEG----

-----IVTEVHLAP-VLLAAVLGRKL----------------------------------

----------------------------------------CAFDELS---QLDPDLY---

KSLIYVKHYSDSD-----------------------------------------VADLSL

TFSIDEDI-----------------------------LGHVRTTDLIPGG----HAMHVT

NENK--IAYVHKMAQYRVFNQ-----TKEQCRAFVSG---------------FLSVL---

--NSNWLS-LFAPHELQYLISGQSTD-------------------------IDLHDLRKH

VQYYG---------------GFHNNHRVIKWLWQILENDFTAEE----RRLFLKFVTSCS

RAPLLGFAYLEPPF----SIRCVEVSDDQDQGDTLASVVRGFLAIKRRQS-------PSR

LP-TASTCFN-----LLKLPN-YGKKSV--LLQKLRYAVHSE-TGF

>Caenorhabditis_briggsae_XM_001665845.1 .

ITVQRNRIIEDGFHHLSKVS----------------LNQLK-STIRVKFVNEQGLDEAGI

---DQDGVFKEFLELTLKKVFDP-------------------------------------

------------------------------------------------------------

------------------------------------------------------------

------------------------------------------------------------

------------------------------------------------------------

--------------------QMNLFSTTST------------------------------

------------GVLYPSPTS---------SLHDDHLALFTFVG--RMLGKAVYEG----

-----IVVDVTLAP-VLLAAVLGNHRL---------------------------------

----------------------------------------CAFDELS---QLDQELY---

RSLTFVKRYEGD------------------------------------------MADLSL

TFSVDEDF-----------------------------MDKISTVDLVLSG----RTIPVT

NENK--IDYVHRMAHHRVFRR-----TQEQCKAFVSG---------------MQSIL---

--QPSWLS-LFAPNDLQCLISGVNSD-------------------------VDLSDLKRN

VQYFG---------------GFHGNHRLIKWLWDILENKFTSEE----RKLFLKFVTSCS

RPPVLGFSYLEPPF----SIRCVEVSDDQDQGDTLGSVVRGFLALRKGTA-------ATR

LP-TASTCFN-----LLKLPN-YNKKSL--LLEKLRYAIHAG-TGF

>Caenorhabditis_elegans_NM_066991.3_oxi1 .

ITVQRNRIIEDGFNHLSKLT----------------IPALK-STIRVKFVNEQGLDEAGI

---DQDGVFKEFLELTLKKVFDP-------------------------------------

------------------------------------------------------------

------------------------------------------------------------

------------------------------------------------------------

------------------------------------------------------------

--------------------QLNLFSTTST------------------------------

------------GVLYPSPTS---------SLHDDHLALFTFVG--RMLGKAVYEG----

-----IVVDVQLAP-VLLAAVLGSHRL---------------------------------

----------------------------------------CAFDELS---QLDPELY---

RSLTFVKRYEGE------------------------------------------MADLSL

TFSVDEDF-----------------------------MGKISTVDLVPSG----RTISVT

NENK--IDYVHRMAHHRVFRR-----TQEQCKAFVTG---------------MQSIL---

--QPTWLS-LFAPNDLQCLISGVNSD-------------------------IDLADLKRN

VQYFG---------------GFHGNHRLIKWLWDILENKFTSEE----RKLFLKFVTSCS

RPPVLGFSYLEPPF----SIRCVEVSDDQDQGDTLGSVVRGFLALRKGTA-------ATR

LP-TASTCFN-----LLKLPN-YNKKSL--LLEKLRYAIHAG-TGF

>Ciona_intestinalis_AK112274.1 .

IRVHRSRLVEDGFEKLGAMQ----------------GNKLK-GIIRVKFINDLGLDEAGI

---DQDGVFKEFLEEIIQKVFNP-------------------------------------

------------------------------------------------------------

------------------------------------------------------------

------------------------------------------------------------

------------------------------------------------------------

--------------------DMNLFKVTAGGD----------------------------

------------MKLYPSPLS---------YLQDHHLHLFTFVG--KMLGKAIYEG----

-----IVLDIPFAN-FFLRSILSKQQTAL-------------------------------

---------------------------------------YSPIDELP---ALDPEFY---

KNLTWIKRYEGD------------------------------------------ISELDL

RFMHEEDI-----------------------------MGKIQSHELIPGG----AGIPVT

NENR--IMYIHTLAHYRLHTQ-----ILHQTKAFVAG---------------FRSVI---

--PTNWIS-MFSSPELQRLISGDNVD-------------------------MDLTDLKKH

VVYYG---------------GFHSSHRVVRWLWDILEQDYSPSQ----RSAFLKFVTSCS

RPPLLGFANLQPQF----SIRCVEVPDDEDTGDTVATVLRGFLRLPSRSRRSSSH--NHR

LP-TASTCFN-----LLKLPN-YPSKVV--LKDKLKQAIANN-TGF

>Trichoplax_adhaerens_XM_002114119.1 .

ININRSRLLEDGYEQLTQLP----------------PEAIK-RTIKVQFVNEQGLTEAGI

---DESGVFKEFLEETIKKAFDP-------------------------------------

------------------------------------------------------------

------------------------------------------------------------

------------------------------------------------------------

------------------------------------------------------------

--------------------ALNLFKVTCGDE----------------------------

------------PRLYPSPTS---------YIHGNHLPLFEFVG--RMLGKAVYEG----

-----LVVEVPLAL-FFLNNVLGGQHSIL-------------------------------

---------------------------------------YSCIDELP---SLDPELY---

KNLNYVKNYEDD------------------------------------------VEDLAL

TFSFDEDV-----------------------------LGKIITHDLKYGG----STIPVT

NENK--LSYIHLMAHFRLRKQ-----LKKQTAAFIRG---------------FHSVI---

--KPEWLQ-VFSGPELQLLIAGENTT-------------------------ISILDLKKY

TKYLG---------------GYHSKHRVIQWLWDILKNDFGEED----RCKFLKFVTSCS

KPPLQGFSNLQPPF----TIRYVEAENEEDRESISRVLFRALSIPNRKSQS------TSR

LP-TSSTCFN-----LLKLPN-YHSKHV--LREKLRYAIQSN-SGF

>Monosiga_brevicollis_XM_001748764.1 .

---------------------------------------LK-DAVRIQFINELGMEEAGI

---DQEGLFKEFLEQTLKEGFNP-------------------------------------

------------------------------------------------------------

------------------------------------------------------------

------------------------------------------------------------

------------------------------------------------------------

--------------------DYGLFCLTAD------------------------------

------------NKLYPSSTS---------EVHQEHLRLFEYLG--RMLGKMLYEG----

-----IVVDIPLAH-FFLNALLARP-----------------------------------

----------------------------------------NTLDELS---TLDADLA---

RNLHMVKTYDGD------------------------------------------VEDLGL

VFAVDEEV-----------------------------LGERHTVPLRPGG----SAVDVT

NENR--VLYVHLMADYKLNQQ-----LRRQVQACRHG---------------FNEFV---

--HGSWLS-FFNAPELQRLVSGDDVP-------------------------LDVNDLRHH

ANYEA---------------GFHSSHRVIKWLFEVVEKDLSREE----QEQFLRFVTSCS

KPPVLGFAALQPRF----TVRALGEGQAEDQYTLGTVVRNFFTSGTD----------TTR

LP-TSSTCFN-----TLKLPM-YKSKRV--LREKLKAAISSS-SGF

>Caenorhabditis_elegans_NM_063441.3 .

--------------------------------------------------------ESGI

---DGGGIFREFLSELLKTAFNV-------------------------------------

------------------------------------------------------------

------------------------------------------------------------

------------------------------------------------------------

------------------------------------------------------------

--------------------ERGFFTFTES------------------------------

------------KLLYPNPTAPF-------LLGVDCLAHFQFIG--RMIGKLIYER----

-----QLQEVRFAE-FFIAQIFETDKNKD-------------------------------

----------------------------------------VDLQHMK---SFDPIIF---

KHLKALQKMNNRE-----------------------------------------LDELQL

DFSVVTSD-----------------------------MGLVRNVNLKPNG----SKFRVT

VENV--HEYVRLYVNYHLKQR-----IASMVDAVRKG---------------ISEII---

--SIEWMR-MFAPHELQIMIAGYEEV-------------------------FTAKELRKF

CELRF--------------AAGTQDINYEEMFWDVID-KLSNDD----KKALLKFVTGCS

RAPVDGFKSIQPRM----GVLVIPSS-------------------------------DDE

LP-TSATCMN-----MLRIPK-YSNRTK--LEEKLRYAINSG-AGF

>Caenorhabditis_briggsae_XM_001678710.1 .

--------------------------------------------------------EQGV

---DGGGIFREFLSELLKDAFSV-------------------------------------

------------------------------------------------------------

------------------------------------------------------------

------------------------------------------------------------

------------------------------------------------------------

--------------------NRGFFTETTES-----------------------------

------------KLLYPNPTAIY-------LLGADCLTHYQFIG--RMIGKLIYER----

-----QLQEVRFAE-FFIAQLFE-DRTKD-------------------------------

----------------------------------------VDLQHMK---SFDPLIF---

KNLKALQTTSEQE-----------------------------------------LDELQL

DFSVVTSD-----------------------------VGLVRTVNLKPNG----SNQRVT

VDNV--HEYIRLYVNYYLKQR-----IATMVGAIRRG---------------ISEVV---

--NIEWMS-MFAPHELQILIAGFEEV-------------------------FTIKELQQN

CQITF------------GMASSTTDQDFIEMIWDVID-KLSKDD----KMAFLRFVTGCS

RAPVDGLKSLYPKM----GILVISTS-------------------------------EEE

LP-TSATCMN-----LLRIPK-YSNRTK--LEEKLRYAINSG-AGF

>Brugia_malayi_XM_001895086.1 .

IAVHRATLYEDAFRALQPHL----------------VPDMK-STIRVQMVNWAGLEEAGV

---DGGGIFREFLFELLQTALDP-------------------------------------

------------------------------------------------------------

------------------------------------------------------------

------------------------------------------------------------

------------------------------------------------------------

--------------------SRGFFATTHE------------------------------

------------QLLYPNPLAPF-------LYPNNFVDHFYFIG--RIIAKLIYEG----

-----LLADIRFAN-FFLLQWMGNPDGTV-------------------------------

----------------------------------------LDLELVK---SYDPLLH---

KNLKFLKRCSTEE-----------------------------------------IENLDL

DFSVMTDN-----------------------------FGVTAKVELKKDG----SCIKVT

ADNR--MEYIQLYVNYYLSKR-----LSPMIAALRSG---------------LRNVI---

--DPEWLR-MFSPLEISMLVGGSDSE-------------------------IDFNELKKF

TTVHN--------------IKSEHDQQYMDLFWLVIN-GFSSGD----KKKLLKFITGCP

RPPIMGFKTLTPPM----GIQLVHD--------------------------------VDK

LP-TAATCMN-----LLKLPL-YDNAET--LRRKLIYAINCG-AGF

>Ciona_intestinalis_XM_002123338.1 .

VKVRRDFLYEDSFNDLSSHN----------------APDLH-RTLRVTFINQAGAEEAGY

----GAGVTREFYQQLVRTSFQP-------------------------------------

------------------------------------------------------------

------------------------------------------------------------

------------------------------------------------------------

------------------------------------------------------------

--------------------GRGLFKLTDD------------------------------

------------RELYPNPNAD--------HVVENMSQHFYFLG--RLLGKMIYEG----

-----MQIELPFAA-FFLCKLLQPKNAD--------------------------------

----------------------------------------VDINHLQ---SLDPDFY---

RNLMFLRSHEGN------------------------------------------VADLDL

NFTVVDDK-----------------------------FGATRVTELVPGG----HDLPVT

NENR--VRYIHLLSNYKLNVQ-----MRVAVDRFRDG---------------LSNVI---

--PLEWLR-MFDHRECQTIISGAEVP-------------------------INVNDMKDN

AAYSG---------------GYTPEHPTIEIFWNVLR-SFTEDE----KRKFLLFVTSCS

RPPLLGF-----------------------------------------------------

----------------------------------------------

>Hydra_magnipapillata_XM_002163757.1 .

ITVNRKYLYQDAYDQLSQDR----------------ADDIK-KVIRIHMINAQGLDEAGI

---DGGGVFREFMSQLLRSGFDP-------------------------------------

------------------------------------------------------------

------------------------------------------------------------

------------------------------------------------------------

------------------------------------------------------------

--------------------SIGFFKTTSQ------------------------------

------------QLLYPNPDIS--------LIYPDYLKHLHFLG--RMLGKVIYES----

-----MMVELPLAD-FFLCKLLNKGGSD--------------------------------

----------------------------------------VDIHHLE---SLDPELY---

KNLLYLKNYKED------------------------------------------VEELSL

SFTVANNE-----------------------------YGETKVLELKPGG----KDISVT

NSNK--IEYIHLMADYRLNKQ-----IRSHCNAFRSG---------------LSDVI---

--NIEWLQ-MFDQRELQILISGAQIP-------------------------IDIEDLRRN

TSYSG---------------SFTAEDPYIEEFWKILE-GLSDHQ----KRLFLKFVTSCS

RPPLLGFSELYPRF----CVHGGGE--------------------------------EDR

LP-TASTCMN-----FLKLPV-YKSIDL--LKTRLIYAIEAE-AGF

>Trichoplax_adhaerens_XM_002114833.1 .

--VKRGRMYEDAFNALTKEK----------------APNLK-KKIRVTILNEQGLEEAGI

---DGGGLFREFLTEVLKEGFNP-------------------------------------

------------------------------------------------------------

------------------------------------------------------------

------------------------------------------------------------

------------------------------------------------------------

--------------------NYGLFKLTQD------------------------------

------------GCLYPNSNVP--------VIISNFRDHYYFLG--RILGKAIFEK----

-----HLVELPLAG-FFLSKILSDNSE---------------------------------

----------------------------------------VGIHHLA---SLDPVMY---

KNLLSLKSYQGD------------------------------------------IADLEL

NFTVVNEN-----------------------------LGEAEVVDLKPGG----QNIAVT

PENL--IEYIHKLADYKLNK----------------------------------------

----------------EVLISGAEVP-------------------------IDLQDLRAN

TNYSG---------------GYSDNDEYINMFWDVAE-DFTDKQ----RRKLLKFVTSCS

RPPLLGFKELFPAF----CIHGSGESND-----------------------------ETR

LP-SASTCMN-----LLKLPR-FRSRDI--LKEKLTYAIESN-SGF

>Tribolium_castaneum_XM_963266.2 .

VVIRRTHLYEDAFEKLSVKN----------------ESDLR-HKVRIQFINSVGLEEAGI

---DGGGIFKEFINEVLKTAFDP-------------------------------------

------------------------------------------------------------

------------------------------------------------------------

------------------------------------------------------------

------------------------------------------------------------

--------------------NRGFFLLTAD------------------------------

------------NTLYPNPNVH--------LIVENFMEHYYFIG--RLVGKAIFEN----

-----ILVDLPLAE-FFLAKLLVDR-----------------------------------

----------------------------------------ASAHYLK---SLDPVLY---

RNLLYLRDYPGD------------------------------------------VSDLGL

DFTTVNND-----------------------------LGETRVVELKPHG----SNIQVT

NENR--LEYIQRLADLKLNVQ-----LKKQCMAFREG---------------LDSVV---

--PLLWLK-LFNHNELQVIIGGDTQE-------------------------IDLSDLKAH

TVYGG---------------EFTADHPTVNLFWKILN-TFTDTQ----KKMLLKFVTSCS

RPPLLGFKELNPQF----CIQSSGS--------------------------------EDR

MP-TASTCLN-----LLKIPI-IKEEEV--LRNKLLQAIEQQ-AGF

>Gallus_gallus_BX931003.1 .

VTIRRNYIYEDAYDKLSPEN----------------EPDLK-KRIRVHLLNAHGLDEAGI

---DGGGIFREFLNELLKSGFNP-------------------------------------

------------------------------------------------------------

------------------------------------------------------------

------------------------------------------------------------

------------------------------------------------------------

--------------------NQGFFKTTNE------------------------------

------------GLLYPNPAAQM-------LVGDSYARHYYFLG--RMLGKALYEN----

-----MLVELPFAS-FFLSKLLGTSAD---------------------------------

----------------------------------------VDIHHLA---SLDPEMY---

KNLLFLKSYEGD------------------------------------------VEELGL

NFTVVNND-----------------------------LGEAQVVELKPGG----KDIPVT

SANR--IAYIHLVADYRLNKQ-----IRQHCLAFRQG---------------LANVV---

--NLEWLR-MFDQQEIQVLISGAQVP-------------------------ISLDDLKSF

TNYSG---------------GYAADHPVIKTFWRVVE-RFTDEE----KRKLLKFVTSCS

RPPLLGFKELYPAF----CIHNGGSD-------------------------------LDR

LP-TASTCMN-----LLKLPE-FYDENL--MRSKLLYAIECA-AGF

>Taeniopygia_guttata_XM_002190902.1 .

VTIRRNYIYEDAYDKLSYEN----------------EPDLK-KRIRVHLLNAHGLDEAGI

---DGGGIFREFLNELLKSGFNP-------------------------------------

------------------------------------------------------------

------------------------------------------------------------

------------------------------------------------------------

------------------------------------------------------------

--------------------NQGFFKTTNE------------------------------

------------GLLYPNPAAQM-------LVGDSFARHYYFLG--RMLGKALYEN----

-----MLVELPFAS-FFLSKLLGTSAD---------------------------------

----------------------------------------VDIHHLA---SLDPEMY---

KNLLFLKSYEGD------------------------------------------VEELGL

NFTVVNND-----------------------------LGEAQVVELKPGG----KDIPVT

SANR--IAYIHLVADYRLNKQ-----IRHHCLAFRQG---------------LANVV---

--NLEWLR-MFDQQEIQVLTSGAQVP-------------------------ISLDDLKSF

TNYSG---------------GYTADHPVIKIFWRVVE-SFTDEE----KRKLLKFVTSCS

RPPLLGFKELYPAF----CIHNGGSD-------------------------------LDR

LP-TASTCMN-----LLKLPE-FYDENL--MRSKLLYAIECA-AGF

>Ornithorhynchus_anatinus_XM_001510706.1 .

VTIRRNYIYEDAYDKLSPEN----------------EPDLK-KRIRVHLLNAHGLDEAGI

---DGGGIFREFLNELLKSGFNP-------------------------------------

------------------------------------------------------------

------------------------------------------------------------

------------------------------------------------------------

------------------------------------------------------------

--------------------NQGFFKTTNE------------------------------

------------GLLYPNPAAQM-------LVGDSFARHYYFLG--RMLGKALYEN----

-----MLVELPFAS-FFLSKLLGTSAD---------------------------------

----------------------------------------VDIHHLA---SLDPEMY---

KNLLFLKSYEDD------------------------------------------VEELGL

NFTVVNND-----------------------------LGEAQVVELKPGG----KDIPVT

SANR--IAYIHLVADYRLNKQ-----IRQHCLAFRQG---------------LANVV---

--NLEWLR-MFDQQEIQVLISGAQVP-------------------------ISLDDLKSF

TNYSG---------------GYSADHPVIKIFWRVVE-SFTDEE----KRKLLKFVTSCS

RPPLLGFKELYPAF----CIHNGGSD-------------------------------LDR

LP-TASTCMN-----LLKLPE-FYDENL--LRSKLLYAIECA-AGF

>Monodelphis_domestica_XM_001364910.1 .

VTIRRNYIYEDAYDKLSPEN----------------EPDLK-KRIRVHLLNAHGLDEAGI

---DGGGIFREFLNELLKSGFNP-------------------------------------

------------------------------------------------------------

------------------------------------------------------------

------------------------------------------------------------

------------------------------------------------------------

--------------------NQGFFKTTNE------------------------------

------------GLLYPNPAAQM-------LVGDSFARHYYFLG--RMLGKALYEN----

-----MLVELPFAG-FFLSKLLGTSAD---------------------------------

----------------------------------------VDIHHLA---SLDPEMY---

KNLLFLKSYEGD------------------------------------------VEELGL

NFTVVNND-----------------------------LGEAQVVELKLGG----KDIPVT

SANR--IAYIHLVADYRLNKQ-----IRQHCLAFRQG---------------LANVV---

--NLEWLR-MFDQQEIQVLISGAQVP-------------------------ISLEDLKSF

TNYSG---------------GYSADHPVIKIFWKVVE-SFTDEE----KRKLLKFVTSCS

RPPLLGFKELYPAF----CIHNGGSD-------------------------------LDR

LP-TASTCMN-----LLKLPE-FYDENL--LRSKLLYAIECA-AGF

>Xenopus_tropicalis_NM_001079050.1 .

VTIRRNYIYEDAYDKLSPEN----------------EPDLK-KRIRVHLLNTHGLDEAGI

---DGGGIFREFLNELLKSGFNP-------------------------------------

------------------------------------------------------------

------------------------------------------------------------

------------------------------------------------------------

------------------------------------------------------------

--------------------NQGFFKTTNE------------------------------

------------GLLYPNPAAQM-------LVGDSFARHYYFLG--RMLGKALYEN----

-----MLVELPFAS-FFLSKLLGTSAD---------------------------------

----------------------------------------VDIHHLA---SLDPEMY---

RNLLFLKSYEDD------------------------------------------VEELGL

NFTVVNND-----------------------------LGEAQVVELKAGG----KDIPVT

SANR--IAYIHLVADYRLNKQ-----IRPHCLAFRQG---------------LANVV---

--NLEWLR-MFDQQELQVLISGAQVP-------------------------ICLDDLKSF

TNYSG---------------GYSADHAVIKIFWRVVE-NFTDEE----KRKLLKFVTSCS

RPPLLGFKELYPAF----CIHNGGSD-------------------------------LER

LP-TASTCMN-----LLKLPE-FYDEHL--MKSKLLYAIECA-AGF

>Xenopus_laevis_NM_001087330.1 .

VTIRRNYIYEDAYDKLSPEN----------------EPDLK-KRIRVHLLNAHGLDEAGI

---DGGGIFREFLNELLKSGFNP-------------------------------------

------------------------------------------------------------

------------------------------------------------------------

------------------------------------------------------------

------------------------------------------------------------

--------------------NQGFFKTTNE------------------------------

------------GLLYPNPAAQM-------LVGDSFARHYYFLG--RMLGKALYEN----

-----MLVELPFAS-FFLSKLLGTSAD---------------------------------

----------------------------------------VDIHHLA---SLDPEMY---

RNLLFLKSYEGD------------------------------------------VEELGL

NFTVVNND-----------------------------LGEAQVVELKAGG----KDIPVT

SANR--IAYIHLVADYRLNKQ-----IRPHCLAFRQG---------------LANVV---

--NLEWLR-MFDQQEIQVLISGAQVP-------------------------ICLDDLKSF

TNYSG---------------GYTADHPVIKIFWRVVE-NFTDEE----KRKLLKFVTSCS

RPPLLGFKELYPAF----CIHNGGSD-------------------------------LER

LP-TASTCMN-----LLKLPE-FYDEHL--MKSKLLYAIECA-AGF

>Bos_taurus_XM_585301.3 .

VTIRRNYIYEDAYDKLSPEN----------------EPDLK-KRIRVHLLNAHGLDEAGI

---DGGGIFREFLNELLKSGFNP-------------------------------------

------------------------------------------------------------

------------------------------------------------------------

------------------------------------------------------------

------------------------------------------------------------

--------------------NQGFFKTTNE------------------------------

------------GLLYPNPAAQM-------LVGDAFARHYYFLG--RMLGKALYEN----

-----MLVELPFAG-FFLSKLLGTSAD---------------------------------

----------------------------------------VDIHHLA---SLDPEVY---

RNLLFLKSYEGD------------------------------------------VEELGL

NFTVVNND-----------------------------LGEAQVVELKSGG----KDIPVT

SANR--IAYIHLVADYRLNRQ-----IRPHCLAFRQG---------------LANVV---

--SLEWLR-MFDQQEIQVLISGAQVP-------------------------ISLEDLKSF

TNYSG---------------GYSAEHPVIKVFWRVVE-GFTDEE----KRKLLKFVTSCS

RPPLLGFKELYPAF----CIHNGGSD-------------------------------LER

LP-TASTCMN-----LLKLPE-FQDEAL--LRSKLLYAIECA-AGF

>Mus_musculus_BC137626.1 .

VTIRRNYIYEDAYDKLSPEN----------------EPDLK-KRIRVHLLNAHGLDEAGI

---DGGGIFREFLNELLKSGFNP-------------------------------------

------------------------------------------------------------

------------------------------------------------------------

------------------------------------------------------------

------------------------------------------------------------

--------------------NQGFFKTTNE------------------------------

------------GLLYPNPAAQM-------LVGDSFARHYYFLG--RMLGKALYEN----

-----MLVELPFAG-FFLSKLLGTSAD---------------------------------

----------------------------------------VDIHHLA---SLDPEVY---

RNLLFLKSYEED------------------------------------------VEELGL

NFTVVNND-----------------------------LGEAQVVELKFGG----KDIPVT

GANR--IAYIHLVADYRLNKQ-----IRPHCLAFRQG---------------LANVV---

--SLEWLR-MFDQQEIQVLISGAQVP-------------------------VSLEDLKSF

TNYSG---------------GYSADHPVIKIFWRVVE-GFTDEE----KRKLLKFVTSCS

RPPLLGFKELYPAF----CIHNGGSD-------------------------------LER

LP-TASTCMN-----LLKLPE-FYDEAL--LRSKLLYAIECA-AGF

>Homo_sapiens_AK127280.1 .

VTIRRNYIYEDAYDKLSPEN----------------EPDLK-KRIRVHLLNAHGLDEAGI

---DGGGIFREFLNELLKSGFNP-------------------------------------

------------------------------------------------------------

------------------------------------------------------------

------------------------------------------------------------

------------------------------------------------------------

--------------------NQGFFKTTNE------------------------------

------------GLLYPNPAAQM-------LVGDSFARHYYFLG--RMLGKALYEN----

-----MLVELPFAG-FFLSKLLGTSAD---------------------------------

----------------------------------------VDIHHLA---SLDPEVY---

KNLLFLKSYEDD------------------------------------------VEELGL

NFTVVNND-----------------------------LGEAQVVELKFGG----KDIPVT

SANR--IAYIHLVADYRLNRQ-----IRQHCLAFRQG---------------LANVV---

--SLEWLR-MFDQQEIQVLISGAQVP-------------------------ISLEDLKSF

TNYSG---------------GYSADHPVIKVFWRVVE-GFTDEE----KRKLLKFVTSCS

RPPLLGFKELYPAF----CIHNGGSD-------------------------------LER

LP-TASTCMN-----LLKLPE-FYDETL--LRSKLLYAIECA-AGF

>Danio_rerio_NM_200473.1 .

VTIRRNYIYEDAYDKLSPEN----------------EPDLK-KRIRVHLLNAHGLDEAGI

---DGGGIFREFLNELLKSGFNP-------------------------------------

------------------------------------------------------------

------------------------------------------------------------

------------------------------------------------------------

------------------------------------------------------------

--------------------NQGFFKTTNE------------------------------

------------GLLYPSPAAEM-------LVGESFTRHYYSLG--RILGKALYEN----

-----MLVELPFAS-FFLSKLLGTSAD---------------------------------

----------------------------------------VDIHHLA---SLDPEMY---

RNLLFLKSYEGD------------------------------------------VEDLGL

NFTVVNND-----------------------------LGEAQVVELKPGG----KDIPVT

TANR--IAYIHLVADYRLNKQ-----IRAHCLAFRQG---------------LANVV---

--NLEWLR-MFDQQEIQVLVSGAHVP-------------------------ICLEDLKRF

TNYSG---------------GYSATHPVIKIFD-VVE-SFSDEE----KRKLLKFVTSCS

RPPLLGFKELYPAF----CIHNGGTD-------------------------------LER

LP-TASTCMN-----LLKLPE-FCDPQL--MRNKLLYAIESS-AGF

>Branchiostoma_floridae_XM_002238477.1 .

VMIRRNYIYEDAYEKLSPEN----------------EPNLK-KRMRVNLVNFQGLEEAGI

---DGGGIFREFLHELLKAGFDP-------------------------------------

------------------------------------------------------------

------------------------------------------------------------

------------------------------------------------------------

------------------------------------------------------------

--------------------NRGFFRTTND------------------------------

------------GQVYPNPASE--------LLVEHYARHYYFLG--RMLGKALYEN----

-----MLVEIPFAS-FFLSKILSKHNH---------------------------------

----------------------------------------VDIDQLQ---SLDPEVY---

RNLLFLKNYDGD------------------------------------------VSDLAL

NFTIVNND-----------------------------LGEAQVVELKPGG----QNIPVT

SENR--IQYIHLVADYRLNKQ-----IRAHCMSFRQG---------------LADVV---

--NLEWLR-MFDDQELQVVISGSQQP-------------------------VDVTDLRKH

TNYSG---------------GYTDEHPVIETFWKVVR-EFTNGQ----RRHLLKFVTSCS

RPPLLGFKELYPAF----CIHHGGPE-------------------------------EDR

LP-TASTCMN-----LLKLPE-FQDEET--MRSKLLYAVESN-AGF

>Strong._purpuratus_XM_001191849.1 .

IIIRRNYIYEDAYDRLRPEN----------------EPDLR-KKLRVTLKNAQGLDEAGI

---DGGGVTREFLSQLLMSAFDP-------------------------------------

------------------------------------------------------------

------------------------------------------------------------

------------------------------------------------------------

------------------------------------------------------------

--------------------NRGFFRTTTD------------------------------

------------ELLYPNPQSA--------MLMEDFTKHYYFIG--RILGKILYEN----

-----LLVEIPFAS-FFLSKILSRHTN---------------------------------

----------------------------------------VDIHHLA---SLDPLMY---

KD--YKSWSQ--------------------------------------------IYRIPT

EFTIFDSS-----------------------------LLKEQILSKDHNG----ESNHMV

TVNM--IFYCFDQPTYTCSKQ-----IRPHILAFRQG---------------LSDVI---

--DLEWLR-MFNHQELQVLISGASMP-------------------------IDVEDLRQH

TNYSG---------------GYKDDSPVIGNFWRVVE-RLTNAQ----RRHLLKFVTSCS

RPPLLGFKELYPAF----CIHHGGSE-------------------------------LDR

LP-TASTCMN-----LLKLPE-FSDEET--LREKLLYAIESG-AGF

>Apis_mellifera_XM_396547.3 .

ISVRRNYLYEDAFEKLSPEN----------------EPELR-LKMRVQLFNTAGLEEAGV

---DGGGLFREFLSELLKTSFDP-------------------------------------

------------------------------------------------------------

------------------------------------------------------------

------------------------------------------------------------

------------------------------------------------------------

--------------------NRGFFRLTKD------------------------------

------------NMLYPNPTVQ--------LLVDDFPKHYYFIG--RILGKALYEN----

-----LLVELPFAE-FFLSKIVGRQSD---------------------------------

----------------------------------------VDVHHLA---SLDPIMY---

RNLLYLKSYKGD------------------------------------------VADLGL

DFTVLSDE-----------------------------LGERRIDELKPGG----ANIPVT

NHNR--IEYIHLMADYKLNKQ-----IRAQCYAFKQG---------------IGSVI---

--PLDWLQ-MFNNKELQVLISGAQIP-------------------------VDVNDLKLH

TNYTG---------------GYAPDHPTITAFWKVVN-EFNDQQ----KGQLLKFVTSCS

RPPLLGFKELDPPF----CIQHAGS--------------------------------VDR

LP-TSSTCMN-----LLKLPE-FPDEKT--LREKLLYAIQAG-AGF

>Nasonia_vitripennis_XM_001607915.1 .

ISVRRNYLYEDAFDKLSPEN----------------EPEMR-LKMRVQLVNAVGLEEAGV

---DGGGLFREFLSELLKTSFDP-------------------------------------

------------------------------------------------------------

------------------------------------------------------------

------------------------------------------------------------

------------------------------------------------------------

--------------------NRGFFRLTKD------------------------------

------------NMLYPNPTVH--------LLVDNFPKHYYFIG--RILGKALYEN----

-----LLVELPFAE-FFLSKIVGTQSD---------------------------------

----------------------------------------VDVHHLA---SLDPIMY---

RNLLYLKSYKGD------------------------------------------VTDLGL

DFTILSDE-----------------------------LGERRVDELKPGG----ANIPVT

NHNR--IEYIHLMADYKLNKQ-----IRAQCNAFKQG---------------IGNVV---

--PLDWLQ-MFNNKELQVLISGAQIP-------------------------VDVSDLKQH

TNYTG---------------GYAPEHPTIIAFWKVVD-EFNDQQ----KSQLLKFVTSCS

RPPLLGFKELDPPF----CIQHAGS--------------------------------VDR

LP-TSSTCMN-----LLKLPQ-FPDEKT--LREKLLYAIQAG-AGF

>Acyrthosiphon_pisum_XM_001950751.1 .

ITVRRNYLYEDAFEKLSVEN----------------EPEIR-QTLRVHMKSAAGLDEAGV

---DGGGLLREFLSELLKTAFDP-------------------------------------

------------------------------------------------------------

------------------------------------------------------------

------------------------------------------------------------

------------------------------------------------------------

--------------------NRGFFRMTND------------------------------

------------NMLYPNPYAH--------LIQQNFAAHYFFIG--RMLGKALYEN----

-----LLVELPLAE-FFLSKIIGRQTE---------------------------------

----------------------------------------VDVHHLA---SLDPLMY---

RNLLSLKNYDGD------------------------------------------VVDLGL

DFTIVIDE-----------------------------FGQTRVEELKPNG----ANITVT

NQNR--IEYIHLMADYKLNTE-----IRKQCYFFKQG---------------LANVI---

--SLDWLR-MFSNHEVQVLISGAEVP-------------------------VDIEDLKKH

TNYVG---------------GYTPEDPAIDLFWTVVN-DFTDEQ----KTKLLKFVTSCS

RPPLLGFKELYPPF----CIQKVSS--------------------------------SDR

LP-TASTCMN-----LLKMPV-FKDYET--LKTKLLYAIQSG-AGF

>Aedes_aegypti_XM_001663593.1 .

LTVRRSHLYEDAFDKLSPLN----------------EPDLR-PKFRIEMVNSAGMREAGI

---DGGGVFREFLSELIKTAFDP-------------------------------------

------------------------------------------------------------

------------------------------------------------------------

------------------------------------------------------------

------------------------------------------------------------

--------------------HRGFFMISKD------------------------------

------------NMLYPNPSVG--------KIVEDYQRHYYFIG--RILGKALYEN----

-----LLVELPLAE-FFLSKLAGKHSD---------------------------------

----------------------------------------VDIHQLA---SLDPVLH---

RNLMSLKAYEGD------------------------------------------VADLGL

DFTIVCDE-----------------------------LGETRIEELKPNG----ANIMVS

SSNR--LEYIQLMADFKLNKQ-----IRSQCLAFRQG---------------LANVL---

--PIEWLY-MFSNKELQVLISGAEIP-------------------------VDVNDLRQN

TRYGG---------------DFTLEHQTIQLFWKVVE-EFDDIQ----RRQLLKFVTSCS

RPPLLGFKDLDPPF----CIQNAGD--------------------------------TDR

LP-SASTCMN-----LLKLPA-FEKEDV--LREKLIYAIQSG-AGF

>Culex_quinquefasciatus_XM_001844497.1 .

LTVRRSHLYEDAFDKLSPMN----------------EPDLR-PKFRIEMVNSAGMREAGI

---DGGGVFREFLSELLKTAFDP-------------------------------------

------------------------------------------------------------

------------------------------------------------------------

------------------------------------------------------------

------------------------------------------------------------

--------------------HRGFFMITKD------------------------------

------------NMLYPNPSVG--------KIVEDYQRHYYFIG--RILGKALYEN----

-----LLVELPLAE-FFLSKLAGKHSD---------------------------------

----------------------------------------VDIHQLA---SLDPVLH---

RNLMSLKAYEGD------------------------------------------VADLGL

DFTIVCDE-----------------------------LGETRVEELKPNG----ANITVS

SSNR--LEYIQLMADFKLNKQ-----IRSQCLAFRQG---------------LANVL---

--PIEWLY-MFSNKELQVLISGAEIP-------------------------VDVHDLRQN

TRYGG---------------DFTLEHNTIQLFWKVVE-EFDDIQ----RRQLLKFVTSCS

RPPLLGFKDLDPPF----CIQNAGD--------------------------------TDR

LP-SASTCMN-----LLKLPA-FEKEDM--LREKLLYAIQSG-AGF

>Anopheles_gambiae_XM_317832.4 .

LTVRRSHLYEDAFDKLSPTN----------------EPDLR-PKFRIEMVNSVGLREAGI

---DGGGVFREFLSELIKLAFDP-------------------------------------

------------------------------------------------------------

------------------------------------------------------------

------------------------------------------------------------

------------------------------------------------------------

--------------------HRGFFMITKD------------------------------

------------NMLYPNPCVG--------KIVEDFQRHYYFIG--RILGKALYEN----

-----LLVELPLAE-FFLSKLAGKHSD---------------------------------

----------------------------------------VDVHQLA---SLDPVLY---

RNLMSLKAYEGD------------------------------------------VADLGL

DFTIVCDA-----------------------------LGETKVEELKPNG----TNIIVN

STNR--IEYIQLMADFKLNQQ-----IRAQCAAFRQG---------------LANVL---

--PIEWLY-MFSNKELQVLISGAEIP-------------------------VDVHDLRQH

TRYGG---------------DFSLEHHTIQLFWKVVE-QFDDIQ----RRLLLKFVTSCS

RPPLLGFKDLDPPF----YIQNAGD--------------------------------TDR

LP-SASTCMN-----LLKLPA-FEEEDV--LREKLLYAIQSG-AGF

>Drosophila_mojavensis_XM_002006852.1 .

VTVRRTHLYEDAYDKLRPEN----------------EPDLR-LKFRIQFVSQLGLDEAGI

---DGGGVFREFLSELIKTSFDP-------------------------------------

------------------------------------------------------------

------------------------------------------------------------

------------------------------------------------------------

------------------------------------------------------------

--------------------NRGFFMVTTD------------------------------

------------NKLYPNPNVA--------DLFSDFEKHYYFIG--RILGKAIYEN----

-----LLVELPLAE-FFLTKLAGKYSD---------------------------------

----------------------------------------VDIHQLA---SLDPELY---

KNLLYLKDYPGD------------------------------------------VSELNL

DFTVASSS-----------------------------LGQTQVVELKPQG----QSTPVT

NSNR--IEYLQLIANYKLNVQ-----IKRHCKAFRKG---------------LSNVL---

--PIEWLY-MFSNKELQILISGAEIP-------------------------IDLEDLKKH

CKYGG---------------EYSPEHPSIVAFWEAVE-GFDDLQ----RRQLLKFVTSCS

RPPLLGFKDLDPPF----FIQNAGD--------------------------------MER

LP-TASTCTN-----LLKLPP-FKNAEQ--MREKLIYAIQSG-VGF

>Drosophila_virilis_XM_002049402.1 .

VTVRRTHLYEDAYDKLRPEN----------------EPDLR-LKFRIQFVSQLGLDEAGI

---DGGGVFREFLSELIKTAFDP-------------------------------------

------------------------------------------------------------

------------------------------------------------------------

------------------------------------------------------------

------------------------------------------------------------

--------------------NRGFFMVTTD------------------------------

------------NKLYPNPNVS--------DLFSDFEKHYYFIG--RILGKAIYEN----

-----LLVELPLAE-FFLTKLAGKYSD---------------------------------

----------------------------------------VDIHQLA---SLDPELY---

RNLLYLKDYPGD------------------------------------------VSELNL

DFTVASSS-----------------------------LGQTQVVELKPQG----QSTPVT

NSNR--IEYLQLIANFKLNVQ-----IKRHCKAFRKG---------------LSNVL---

--PIEWLY-MFSNKELQILISGAEIP-------------------------IDLEDLKKH

CKYGG---------------EYTPEHPSIVAFWEAVE-GFDDLQ----RRQLLKFVTSCS

RPPLLGFKDLDPPF----FIQNAGD--------------------------------MER

LP-TASTCTN-----LLKLPP-FKNAEQ--MREKLIYAIQSG-VGF

>Drosophila_grimshawi_XM_001985718.1 .

ITVRRSHLYEDAYDKLRPEN----------------EPDLR-LKFRIQFVSSLGLDEAGI

---DGGGVFREFLSELIKTAFDP-------------------------------------

------------------------------------------------------------

------------------------------------------------------------

------------------------------------------------------------

------------------------------------------------------------

--------------------NRGFFMVTTD------------------------------

------------NKLYPNPNVA--------DLFGDFEKHYYFIG--RILGKAIYEN----

-----LLVELPLAE-FFLTKLAGKYSD---------------------------------

----------------------------------------VDIHQLA---SLDPELY---

RNLLYLKDYTGD------------------------------------------VSELNL

DFTVASSS-----------------------------LGQTQVVELKPQG----QSTPVT

NSNR--IEYLQLIANYKLNVQ-----IKRHCKAFRKG---------------LSNVL---

--PIEWLY-MFSNKELQILISGAEIP-------------------------IDLEDLKKH

CKYGG---------------EYTPEHPSIVAFWAAVE-GFNDLQ----RRQLLKFVTSCS

RPPLLGFKDLDPPF----FIQNAGD--------------------------------MER

LP-TASTCTN-----LLKLPP-FNTIEQ--MREKLLYAIQSG-VGF

>Drosophila_pseudoobscura_XM_001361963.2 .

ITVRRTHLYEDAYDKLRPEN----------------EPDLR-PKFRIQFVSSLGLDEAGI

---DGGGVFREFLSELIKSAFDP-------------------------------------

------------------------------------------------------------

------------------------------------------------------------

------------------------------------------------------------

------------------------------------------------------------

--------------------NRGFFMVTTD------------------------------

------------NKLYPNPNVA--------DLFGDFEKHYYFIG--RILGKAIYEN----

-----LLVELPLAE-FFLTKLAGKYSD---------------------------------

----------------------------------------VDIHQLA---SLDPELY---

RNLLYLKDYTGD------------------------------------------VSELNL

DFTVASSS-----------------------------LGQTQIVELKPQG----QSTPVT

NSNR--IEYIQLIADYKLNVQ-----IRRHCNAFRKG---------------LSNVL---

--PIEWLY-MFSNKELQILISGAEIP-------------------------IDLEDLKKH

CKYGG---------------EYTPEHPSIVAFWEALE-GFDDLQ----RRQLLKFVTSCS

RPPLLGFKDLDPPF----FIQNAGD--------------------------------MER

LP-TASTCTN-----LLKLPP-FNTVEQ--MREKLLYAIQSG-VGF

>Drosophila_willistoni_XM_002063649.1 .

ITVRRSHLYEDAYDKLRPEN----------------EPDLR-LKFRIQFVSSLGLDEAGI

---DGGGVFREFLSELIKTAFDP-------------------------------------

------------------------------------------------------------

------------------------------------------------------------

------------------------------------------------------------

------------------------------------------------------------

--------------------NRGFFMVTTD------------------------------

------------NKLYPNPNVA--------DLFADYEKHYYFIG--RILGKAIYEN----

-----LLVELPLAE-FFLTKLAGKYSD---------------------------------

----------------------------------------VDIHQLA---SLDPELY---

RNLLYLKDYTGD------------------------------------------VSELNL

DFTVASSS-----------------------------LGQTQIVELKPQG----QSIPVT

NSNR--IEYLQLIADYKLNVQ-----IRRHCNAFRKG---------------LSNVL---

--PIEWLY-MFSNKELQILISGAEIP-------------------------IDLEDLKRH

CKYGG---------------EYTPEHPSIVAFWSALD-GFDDLQ----RRQLLKFVTSCS

RPPLLGFKDLDPPF----FIQNAGD--------------------------------MER

LP-TASTCTN-----LLKLPP-FKTIDQ--MREKLLYAIQSG-VGF

>Drosophila_ananassae_XM_001960632.1 .

LTVRRSHLYEDAYDKLRPEN----------------EPDLR-LKFRIQFVSSLGLDEAGI

---DGGGVFREFLSELIKTAFDP-------------------------------------

------------------------------------------------------------

------------------------------------------------------------

------------------------------------------------------------

------------------------------------------------------------

--------------------NRGFFMVTTD------------------------------

------------NKLYPNPNVA--------DLFEDYEKHYYFIG--RILGKAIYEN----

-----LLVELPLAE-FFLTKLAGKYSD---------------------------------

----------------------------------------VDIHQLA---SLDPELY---

RNLLYLKDYTGD------------------------------------------VSELNL

DFTVASSS-----------------------------LGQTQIVDLKPQG----QSIPVS

NSNR--IEYLQLIADYKLNVQ-----IRRHCNAFRKG---------------LSNVL---

--PIEWLY-MFSNKELQILISGAEIP-------------------------IDLEDLKKH

CEYGG---------------EFTPEHPSIVVFWAALE-GFDDLQ----RRQLLKFVTSCS

RPPLLGFKDLHPPF----FIQNAGD--------------------------------MER

LP-TASTCAN-----LLKLPP-FNNVEQ--MREKLLYAIQFG-AGF

>Drosophila_yakuba_XM_002092755.1 .

ITVRRSHLYEDAYDKLRPEN----------------EPDLR-FKFRIQFVSSLGLDEAGI

---DGGGVFREFLSELIKTAFDP-------------------------------------

------------------------------------------------------------

------------------------------------------------------------

------------------------------------------------------------

------------------------------------------------------------

--------------------NRGFFMVTTD------------------------------

------------NKLYPNPNVA--------DLFEDYEKHYYFIG--RILGKSIYEN----

-----LLVELPLAE-FFLTKLAGKYSD---------------------------------

----------------------------------------VDIHQLA---SLDPELY---

RNLLYLKDYSGD------------------------------------------VSELNL

DFTVASSS-----------------------------LGQTQIVELKPQG----QSIPVT

NSNR--IEYLQLIADYKLNVQ-----IRSHCNAFRKG---------------LSNVL---

--PIEWLY-MFSNKELQILISGAEIP-------------------------IDLEDLKRH

CEYGG---------------EFSPEHPSIVAFWEVLE-GFDDLQ----RRQLLKFVTSCS

RPPLLGFKDLDPPF----FIQNTGD--------------------------------MER

LP-TASTCTN-----LLKLPP-FKTVEQ--MREKLLYAIQSG-AGF

>Drosophila_erecta_XM_001976535.1 .

ITVRRSHLYEDAFDKLRPEN----------------EPDLR-VKFRIQFVSSLGLDEAGI

---DGGGVFREFLSELIKTAFDP-------------------------------------

------------------------------------------------------------

------------------------------------------------------------

------------------------------------------------------------

------------------------------------------------------------

--------------------NRGFFMVTTD------------------------------

------------NKLYPNPNVA--------DLFEDYEKHYYFIG--RILGKSIYEN----

-----LLVELPLAE-FFLTKLAGKYAD---------------------------------

----------------------------------------VDIHQLA---SLDPELY---

RNLLYLKDYSGD------------------------------------------VSELNL

DFTVASSS-----------------------------LGQTQIVELKPQG----QSIPVT

NSNR--IEYLQLIADYKLNVQ-----IRSHCNAFRKG---------------LSNVL---

--PIEWLY-MFSNKELQILISGAEIP-------------------------IDLEDLKKH

CKYGG---------------EFTPEHPSIVAFWEVLE-GFDDLQ----RRQLLKFVTSCS

RPPLLGFKDLDPPF----FIQNTGD--------------------------------MER

LP-TASTCTN-----LLKLPP-FKTVEQ--MREKLLYAIQSG-AGF

>Monosiga_brevicollis_XM_001743375.1 .

LKVRRTDILEDTMKALSMVS----------------PTQWT-KPLRIKFE-----DEEGV

---DEGGLVKEFFQLVMPKLYAH-------------------------------------

------------------------------------------------------------

------------------------------------------------------------

------------------------------------------------------------

------------------------------------------------------------

--------------------AFVPMESEGL------------------------------

------------RWFWFAGPT------------EDRLQAYYAMG--LLFGLALYNH----

-----VLVEPLFPS-VFYKMLLLADPEMS-------------------------------

---------------------------------------QLTLDDLA---SCAPDVA---

KGLRSLLDYDGHNE----------------------------------------KEVFGL

TFELSHKDVDGRVETMELKVCVCVCVC------LCVLVTPVLVSDLAQAN---RWEMDVT

ALNK--HEYVQLYVQYYLYER-----VAASVDAFRTG---------------FFMIVQ--

--KTPAFY-MLQPRELELLLCGNPE--------------------------YKLDQLKDN

ARYIN----------------YSAKDTVVQWFWDYVL-ALPQSK----RRRFLMFLTGSM

RVPVQGAKGIKVTI----QRVADTT----------------------------------R

LP-VAHTCFN-----VLDLPP-YETREE--LERKLGYALEET-EGF

>Drosophila_virilis_XM_002048516.1 .

LTVRRDQLINDALIGLELVAMSN-------------PKDLK-KQLVVEFV-----GEQGI

---DEGGVSKEFFQLIVEEIFNP-------------------------------------

------------------------------------------------------------

------------------------------------------------------------

------------------------------------------------------------

------------------------------------------------------------

--------------------AFGMFVQQEET-----------------------------

------------NNMWFNATP------------FENGAQFTLIG--IIIGLAIYNN----

-----VILAVNFPM-VVYRKLMGYR-----------------------------------

----------------------------------------GTFYDLS---DWSPTLY---

KSLKTMLDYQGQDM----------------------------------------EEVFEQ

TFKISYSN----------------------------VFGELVEHELVPHG----SEVLVG

QHNK--RLFVNLYSDFLLNVN-----IQQQFKAFRKG---------------FEMVT---

--DESPLKLLFRPEEIEKLVCGSRE--------------------------FDFVELEHS

TEYEG---------------GYTEETQIVRDFWSIVH-AMPHES----KRKLLEFTTGSA

RVPVGGLKCLRLLI----TRHGPD---------------------------------SDR

LP-TSHTCFN-----VLLLPE-YSSKEK--LEERLLKAINYS-KGF

>Drosophila_grimshawi_XM_001984742.1 .

LTVRRDQLINDALIGLELVAMSN-------------PKDLK-KQLVVEFV-----GEQGI

---DEGGVSKEFFQLIVEEIFNP-------------------------------------

------------------------------------------------------------

------------------------------------------------------------

------------------------------------------------------------

------------------------------------------------------------

--------------------AFGMFVQEEET-----------------------------

------------NNMWFNATP------------FENGAQFTLIG--IIIGLAIYNN----

-----VILAVNFPM-VVYRKLMGYR-----------------------------------

----------------------------------------GTFYDLS---DWSPTLY---

KSLNDMLDYQGHDM----------------------------------------EEVFEQ

TFKISYSN----------------------------VFGELVEHELVPHG----SEVLVG

QHNK--RLFVNLYSDFLLNVN-----IQQQFNAFRKG---------------FEMVT---

--DESPLKLLFRPEEIEKLVCGSRE--------------------------FDFVELEHS

TEYEG---------------GYTEETQIVQDFWSIVH-AMPHES----KRKLLEFTTGSD

RVPVGGLKCLRLMI----TRHGPD---------------------------------SDR

LP-TSHTCFN-----VLLLPE-YSSKEK--LEERLLKAINYS-KGF

>Drosophila_mojavensis_XM_002008761.1 .

LTVRRDQLINDALIGLELVAMSN-------------PKDLK-KQLVVEFV-----GEQGI

---DEGGVSKEFFQLIVEEIFNP-------------------------------------

------------------------------------------------------------

------------------------------------------------------------

------------------------------------------------------------

------------------------------------------------------------

--------------------AFGMFVQQEET-----------------------------

------------NNMWFNPTP------------FENGAQFTLIG--IIIGLAIYNN----

-----VILAVNFPM-VVYRKLMGYR-----------------------------------

----------------------------------------GTFYDLC---DWSPTLY---

KSLKAMLDYQGHDM----------------------------------------EEVFEQ

TFKISYSN----------------------------VFGELVEHELVPHG----SEVLVG

QHNK--RLFVNLYSDFLLNSN-----IEQQFKAFRKG---------------FEMVT---

--DESPLKLLFRPEEIEKLVCGSRE--------------------------FDFVELEHS

TEYEG---------------GYTEETQIVQDFWSIVH-AMPHEA----KRKLLEFTTGSA

RVPVGGLKCLRLLI----TRHGPD---------------------------------SDR

LP-SSHTCFN-----VLLLPE-YSSKEK--LEERLLKAINYS-KGF

>Drosophila_ananassae_XM_001956476.1 .

LTVRRDQLINDALIGLELVAMSN-------------PKDLK-KQLVVEFV-----GEQGI

---DEGGVSKEFFQLIVEEIFNP-------------------------------------

------------------------------------------------------------

------------------------------------------------------------

------------------------------------------------------------

------------------------------------------------------------

--------------------AFGMFVQQDET-----------------------------

------------NNMWFNATP------------FENGAQFTLIG--IIIGLAIYNN----

-----VILAVNFPM-VVYRKLMGYC-----------------------------------

----------------------------------------GTFADLA---DWSPTLH---

KSLQSLLDYQGQDM----------------------------------------EEVFDQ

TFRISYSN----------------------------VFGEMVEHELVPNG----KDLLVG

QHNK--QLFVNLYSDFLLNTN-----IQQQFNAFRKG---------------FEMVT---

--DESPLKLLFRPEDIEMLVCGSRE--------------------------FDFVELEHS

TEYEG---------------GYTEETKIIQDFWSIVH-AMPIES----KRKLLEFTTGSA

RVPVGGLKCLRLLI----TRHGPD---------------------------------SDR

LP-TSHTCFN-----VLLLPE-YSSREK--LEERLLKAINYS-KGF

>Drosophila_willistoni_XM_002062449.1 .

LNVRRDQLINDALVGLELVAMSN-------------PKDLK-KQLVVEFV-----GEQGI

---DEGGVSKEFFQLIVEEIFNP-------------------------------------

------------------------------------------------------------

------------------------------------------------------------

------------------------------------------------------------

------------------------------------------------------------

--------------------AFGMFVQQEET-----------------------------

------------NNMWFNATP------------FENGAQFTLIG--IIIGLAIYNN----

-----VILAVNFPM-VVYRKLMGYR-----------------------------------

----------------------------------------GTFYDLS---DWSPMLY---

KSLKSMLDYQGQDM----------------------------------------EEVFDQ

TFRISYSN----------------------------VFGEMVEHDLVPNG----KDLTVG

QHNK--KLFVNLYADFLLNTN-----IKQQFNSFRKG---------------FEMVT---

--DESPLKLLFRPEEIEMLVCGSQE--------------------------FDFVELEQS

TEYEG---------------GYTKETQIVQDFWSIVH-GMPNES----KRKLLEFTTGSA

RVPVGGLKCLRLLI----TRHGPD---------------------------------SDR

LP-TSHTCFN-----VLLLPE-YSSKEK--LEERLLKAINYS-KGF

>Drosophila_melanogaster_NM_140195.2 .

LTVRRDQLINDALIGLEMVAMSN-------------PKDLK-KQLVVEFV-----GEQGI

---DEGGVSKEFFQLIVEEIFNP-------------------------------------

------------------------------------------------------------

------------------------------------------------------------

------------------------------------------------------------

------------------------------------------------------------

--------------------AFGMFIQQEET-----------------------------

------------NNMWFNATP------------FENGAQFTLIG--IIIGLAIYNN----

-----VTLAVNFPM-VVYRKLIGYC-----------------------------------

----------------------------------------GTFADLS---DWSPALY---

KSLKSMLDYQGQDM----------------------------------------EEVFEQ

TFKISYSD----------------------------VFGDVVQHELVPNG----QDVLVG

QHNK--ELFVNLYSDFLLNTN-----IEQQFNAFRKG---------------FEMVT---

--DESPLKLLFRPEEIEMLVCGSRE--------------------------FDFVELENS

TVYEG---------------GYTEKSQYIQDFWSIVH-AMPSED----KHKLLEFTTGSA

RVPVGGLKCLRLLI----TRHGPD---------------------------------SDR

LP-TSHTCFN-----VLLLPE-YSSREK--LEERLMKAINYS-KGF

>Drosophila_sechellia_XM_002030050.1 .

LTVRRDHLINDALIGLEMVAMSN-------------PKDLK-KQLVVEFV-----GEQGI

---DEGGVSKEFFQLIVEEIFNP-------------------------------------

------------------------------------------------------------

------------------------------------------------------------

------------------------------------------------------------

------------------------------------------------------------

--------------------AFGMFIQQEET-----------------------------

------------NNMWFNATP------------FENGAQFTLIG--IIIGLAIYNN----

-----VTLAVNFPM-VVYRKLIGYR-----------------------------------

----------------------------------------GTFADLS---DWSPTLY---

KNLKSMLHYQGQDM----------------------------------------EEVFEQ

TFKISYSD----------------------------VFGDVVEHELLPNG----QDVLVG

QHNK--ELFVNLYSDFLLNTN-----IEQQFNAFRKG---------------FEMVT---

--DESPLKLLFRPEEIEMLVCGSRE--------------------------FDFVELENS

TVYEG---------------GYTENSQYIQDFWSIVH-AMPSED----KHKLLEFTTGSA

RVPVGGLKCLRLLI----TRHGPD---------------------------------SDR

LP-TSHTCFN-----VLLLPE-YSSREK--LEERLMKAINYS-KGF

>Drosophila_erecta_XM_001972329.1 .

LTVRRDQLINDALIGLEMVAMSN-------------PKDLK-KQLVVEFV-----GEQGI

---DEGGVSKEFFQLIVEEIFNP-------------------------------------

------------------------------------------------------------

------------------------------------------------------------

------------------------------------------------------------

------------------------------------------------------------

--------------------AFGMFIQQEET-----------------------------

------------NNMWFNATP------------FENGAQFTLIG--IIIGLAIYNN----

-----VILAVNFPM-VVYRKLMGYC-----------------------------------

----------------------------------------GTFADLS---DWSPALY---

KSLKSIMDYQGQDM----------------------------------------EEVFDQ

TFKISYSD----------------------------VFGDMVEHELVPNG----KDVLVG

QHNK--ELFVNLYSDFLLNTN-----IQQQFNAFRKG---------------FEMVT---

--DESPLKLLFRPEEIEMLVCGSRE--------------------------FDFVELENS

TVYEG---------------GYTEESQYIQDFWSIVH-AMPNEA----KHKLLEFTTGSA

RVPVGGLKCLRLLI----TRHGPD---------------------------------SDR

LP-TSHTCFN-----VLLLPE-YSSREK--LEERLMKAINYS-KGF

>Drosophila_yakuba_XM_002094356.1 .

LTVRRDQLINDALIGLEMVAMSN-------------PKDLK-KQLVVEFV-----GEQGI

---DEGGVSKEFFQLIVEEIFNP-------------------------------------

------------------------------------------------------------

------------------------------------------------------------

------------------------------------------------------------

------------------------------------------------------------

--------------------AFGMFIQQEET-----------------------------

------------NNMWFNATP------------FENGAQFTLIG--IIIGLAIYNN----

-----VILAVNFPM-VVYRKLMGYC-----------------------------------

----------------------------------------GTFADLS---DWSPTLH---

KSLKAMMDYQGQDM----------------------------------------EEVFDQ

TFKISYSD----------------------------VFGDMVEHELVPNG----KDVLVG

QHNK--ELFVNLYSDFLLNTN-----IQQQFNAFRKG---------------FEMVT---

--DESPLKLLFRPEEIEMLVCGSRE--------------------------FDFVELENS

TVYEG---------------GYTEESQYIQDFWSIVH-AMPNEA----KHKLLEFTTGSA

RVPVGGLKCLRLLI----TRHGPD---------------------------------SDR

LP-TSHTCFN-----VLLLPE-YSSRDK--LEERLMKAINYS-KGF

>Drosophila_pseudoobscura_XM_001353893.2 .

LTVRRDQLINDALVGLELVAMSN-------------PKDLK-KQLVVEFV-----GEQGI

---DEGGVSKEFFQLIVEEIFNP-------------------------------------

------------------------------------------------------------

------------------------------------------------------------

------------------------------------------------------------

------------------------------------------------------------

--------------------AFGMFLQQEET-----------------------------

------------NNMWFNATP------------FENGAQFTLIG--IIIGLAIYNN----

-----VILAVNFPM-VVYRKLLGYR-----------------------------------

----------------------------------------GTFCDLR---DYSPMLY---

SSLKSLLDFEGENM----------------------------------------EDVFDQ

TFKISYSN----------------------------VFGEMVEHELVPHG----DVVLVG

QHNK--QLFVDLYADFLLNKN-----IQQQFNSFRKG---------------FEMVT---

--DESPLKLLFRPEEIEMLVCGSRE--------------------------FDFVELEHS

TEYEG---------------GYSYETPIIKDFWKIVH-AMPHES----KRKLLEFATGSA

RVPVGGLKCLRLLI----TRHGPD---------------------------------SDR

LP-TSHTCFN-----VLLLPE-YSSKVK--LEERLLKAINYS-KGF

>Anopheles_gambiae_XM_551819.3 .

LKIRRDHIIDDALVELEIIAMSN-------------PKDLK-KQLVVEFT-----GEQGI

---DEGGVSKEFFQLIIEEIFNP-------------------------------------

------------------------------------------------------------

------------------------------------------------------------

------------------------------------------------------------

------------------------------------------------------------

--------------------DYGMFVTNEDS-----------------------------

------------NTVWFNSIS------------FENEAQFTLIG--IVLGLAIYNN----

-----IILAVNFPM-VVYRKLMGMK-----------------------------------

----------------------------------------GSFLDLK---DLNPVLF---

NSLKSLLDYTENDM----------------------------------------EEVFMQ

TFKIGYRD----------------------------VFGNLLEHELKPDG----DKIFVT

QDNK--QDFVELYSDFMLNKS-----VEKQFNAFRRG---------------FQMVT---

--DESPLHLLFRPEEVELIVCGSKE--------------------------FDFDELEQS

TEYEG---------------GFTAESQTIKDFWSIVH-GLSMEV----KRKLLQFTTGSD

RVPVGGLSRLKLVV----ARNGPD---------------------------------SDR

LP-TSHTCFN-----VLLLPE-YNSKEK--LEERLLKAINYS-KGF

>Aedes_aegypti_XM_001662572.1 .

LKIRRDHIIDDALVELEMIAMSN-------------PKDLK-KQLVVEFS-----GEQGI

---DEGGVSKEFFQLIIEEIFNP-------------------------------------

------------------------------------------------------------

------------------------------------------------------------

------------------------------------------------------------

------------------------------------------------------------

--------------------DYGMFINIEDT-----------------------------

------------NMVWFNSTS------------FENEAQFTLIG--IVLGLAIYNN----

-----IILAVNFPM-VVYRKLMGMK-----------------------------------

----------------------------------------GSFIDLR---DWNPVLY---

NSLKSILDHQENDM----------------------------------------EEVFMQ

TFKICYKD----------------------------VFGNTLDHELKPDG----DKIFVN

QDNK--QEFVELYADFLLNQN-----IEKQFMAFKRG---------------FQMVT---

--DESPLHLLFRPEEVELIVCGSKE--------------------------FDFNELEQS

TEYEG---------------GFTAESQTIKDFWDIVH-GLSMES----KLKLLQFTTGSD

RVPVGGLSRLKLVI----ARNGPD---------------------------------CDR

LP-TSHTCFN-----VLLLPE-YSTREK--LEERLLKAINYS-KGF

>Culex_quinquefasciatus_XM_001842960.1 .

LKIRRDHIIDDALVELEMIAMSN-------------PKDLK-KQLVVEFS-----GEQGI

---DEGGVSKEFFQLIIEEIFNP-------------------------------------

------------------------------------------------------------

------------------------------------------------------------

------------------------------------------------------------

------------------------------------------------------------

--------------------DYGMFINHEDT-----------------------------

------------NTVWFNSTS------------FENEAQFTLIG--IVLGLAIYNN----

-----IILAVNFPM-VVYRKLMGMK-----------------------------------

----------------------------------------GSFADLE---DWNPILF---

NSLKSMLDHQDNDM----------------------------------------EEVFMQ

TFKICYKD----------------------------VFGNAIDHELKPDG----DKIFVT

QDNK--HEFVELYTDFLLNQC-----CEKQFKAFKRG---------------FQMVT---

--DESPLHLLFRPEEIELIVCGSKK--------------------------FDFNELEQS

TEYEG---------------GFTAESQTIKDFWEIVH-GLSMES----KRKLLQFTTGSD

RVPVGGLSRLKLVI----ARNGPD---------------------------------CER

LP-TSHTCFN-----VLLLPE-YDSREK--LEERLLKAINYS-KGF

>Pediculus_humanus_EEB19773.1 .

LKVRRDHIIDDALVELEMVAMEN-------------PKDLK-KQLVVEFE-----GEQGI

---DEGGVSKEFFQLIVEEIFNP-------------------------------------

------------------------------------------------------------

------------------------------------------------------------

------------------------------------------------------------

------------------------------------------------------------

--------------------DYGMFVYQQET-----------------------------

------------ETVWFNPTS------------FESDAQFTLIG--IVLGLAIYNN----

-----IILDVRFPM-VVYKKLMGKR-----------------------------------

----------------------------------------GTFYDVQ---DWNPTLF---

AGLKELLDYEEDDI----------------------------------------EDVLIQ

TFRVCYTD----------------------------VFGNTLFHELKEGG----DQIYVN

QDNK--KEFVDLYSNFLLNKC-----VEKQFRAFRKG----------------FQMVT--

--DESPLIFLFRPEEVEQLVCGSKN--------------------------FDFNELEEA

TEYDG---------------GYTAETPIIKNFWKLVH-AMSLDD----KRKLLQFATGSD

RVPVGGLSRLKLVI----AKNGPD---------------------------------SDR

LP-TAHTCFN-----VLLLPE-YSSREK--LQDRLEKAINYS-KGF

>Tribolium_castaneum_XM_964003.1 .

LKVRRDHIIDDALVELEMISMEN-------------PNDLK-KQLVVEFE-----GEQGI

---DEGGVSKEFFQLVIEEIFNP-------------------------------------

------------------------------------------------------------

------------------------------------------------------------

------------------------------------------------------------

------------------------------------------------------------

--------------------DYAMFTSQSET-----------------------------

------------GTVWFNPTS------------FESDAQFTLIG--IVLGLAIYNN----

-----VILAVNFPM-VLYRKLLGKR-----------------------------------

----------------------------------------GSFEDLQ---DWNLTLY---

NSLKQLLEYNEPDV----------------------------------------EEVFMQ

TFRISYQD----------------------------VFGSIINYDLKDRG----DEINVT

QENK--YEFVDLYADFLLNKS-----VEKQFRAFYKG---------------FQMVV---

--DESPLELLFRPEEIEILICGSKN--------------------------FDFDELESS

TEYDG---------------GYTSESQIIKDFWSIVH-ALSLED----KRKLLQFTTGSD

RVPIGGLSRLKLVI----ARNGPD---------------------------------SDR

LP-TAHTCFN-----VLLLPE-YSSKEK--LKDRLIKAISYS-KGF

>Nasonia_vitripennis_XR_036787.1 .

LKVRRDHLIEDALVELEVVAMEN-------------PYDLK-KQLVVEFE-----GEQGV

---DEGGVSKEFFQLVVEEIFNP-------------------------------------

------------------------------------------------------------

------------------------------------------------------------

------------------------------------------------------------

------------------------------------------------------------

--------------------DYGMFTTQEDT-----------------------------

------------QTMWFNPTS------------FESDAQFTLIG--VVLGLAIYNN----

-----VILDVRFPM-VVYRKLLGRK-----------------------------------

----------------------------------------GSFSDLE---DWNPTLY---

RTMMEMLEYTGDDM----------------------------------------SDTFMQ

TFKVGYKD----------------------------VFGSLLFHELKEKG----DEIYVT

QENK--REFVDLYADFLLNQS-----VERQFKAFRRG---------------FQMVT---

--DESPLALLFRPEEIEQLVCGSKI--------------------------FDFAELEAA

TEYEG---------------GYSVESEAIRNFWQVVH-SLTPED----QRRLLQFTTGSD

RVPVGGLSRLKMVI----ARHGPD---------------------------------SDR

LP-IAHTCFN-----VLLLPD-YSTIEK--LQDRLLKAINYS-KGF

>Apis_mellifera_XM_394656.3 .

LKVRRDRLIDDALVELEMIAVEN-------------PSDLK-KQLVVEFE-----GEQGV

---DEGGVSKEFFQLIVEEIFNP-------------------------------------

------------------------------------------------------------

------------------------------------------------------------

------------------------------------------------------------

------------------------------------------------------------

--------------------DYGMFTTQEDT-----------------------------

------------QMTWFNPTS------------FESDAHFTLIG--VVLGLAIYNN----

-----VILDVRFSM-VVYRKLLGRK-----------------------------------

----------------------------------------GCFADLE---DWSPTLY---

RTLKELMDYTGDDM----------------------------------------PDTFMQ

TFRVAYKD----------------------------VFGSISFHDLKQNG----DELFVT

QENK--KEFVDLYADFLLNKS-----VERQFKAFRRG---------------FQMVT---

--DESPLALLFRPEEIEQLVCGSKV--------------------------FDFAELEAA

TEYEG---------------GYTVDSKAVRNFWRVAH-SLPPES----QRRLLQFTTGSD

RVPVGGLSRLKMVI----ARHGPD---------------------------------SDR

LP-IAHTCYN-----VLLLPD-YSTIEK--LQDRLLKAINYS-KGF

>Mya_arenaria_AF154109.2 .

IRVSRNRIIDDALVALEMVAMEN-------------PSDLR-KQLFVEFD-----GEQGV

---DEGGVSKEFFQLIVEEIFNV-------------------------------------

------------------------------------------------------------

------------------------------------------------------------

------------------------------------------------------------

------------------------------------------------------------

--------------------DFGMFTYNPDT-----------------------------

------------RQFWFNPMS------------FENEGQFTLCG--IVLGLAIYNS----

-----TIVDVHFPS-VVYRKLVGKL-----------------------------------

----------------------------------------GTFQDLK---GVDPTLA---

RTLQDLLDYEGTDM----------------------------------------EDVFMQ

TFQISYLD----------------------------VFGSTITHSLKDAG----ETIPVN

QLNK--QEFVDMYADFILNKS-----IEKQFRAFRRG---------------FSMVT---

--NESPLRALFRPEEIEMLICGSED--------------------------YDFNALEDA

CEYDG---------------GFDKKSQTIRDFWEVVH-GFDDEK----KCQLLQFTTGTD

RVPVGGLSKLKLII----ARNGPD---------------------------------SDR

LP-TAHTCFN-----VLLLPD-YLNKEK--LQERLLEAITYS-KGF

>Nematostella_vectensis_XM_001632475.1 .

LKVRRDHLIEDALVSLEMVAQDN-------------PLDLK-KQLFVEFE-----GEQGI

---DEGGVSKEFFQLIVEQIFNP-------------------------------------

------------------------------------------------------------

------------------------------------------------------------

------------------------------------------------------------

------------------------------------------------------------

--------------------DYGMFTYDEAT-----------------------------

------------RICWFNPTS------------FESDAQFCLIG--LVLGLAIYNN----

-----IILDIHFPM-VVYRKLFGKP-----------------------------------

----------------------------------------GTVEDLK---DSHPVLY---

MSLTELLTYEGNV-----------------------------------------EEDLMC

TFKIGYTD----------------------------VFGSDLTHELKANG----ESIPVT

KENR--QEYVNLHADFILNKS-----VEKQFTAFKRG---------------FDMVV---

--DESPLKIFFRPDEVEMLVCGSKD--------------------------FDFDALEKA

TEYDG---------------GLTKDSALIRWFWEIVH-SFDMEQ----KRQLLMFTTGSD

RIPVGGLAKLKLII----AKNGPD---------------------------------SPR

LP-TAHTCFN-----VLLLPD-YATKEK--LEERLLKAITHA-KGF

>Strong._purpuratus_XM_775717.2 .

LKVRRDHVVDDSLVRLEMIAVDN-------------PMDLK-KQLYVEFE-----GEQGV

---DEGGVSKEFFQLVIQEIFNP-------------------------------------

------------------------------------------------------------

------------------------------------------------------------

------------------------------------------------------------

------------------------------------------------------------

--------------------DIGMFTHNSDL-----------------------------

------------QTYWFNPTS------------FETNRQYTLIG--ILLGLAIYNN----

-----VILDVTFPM-VVYRKLMGRR-----------------------------------

----------------------------------------GVFADLH---DAQPVLY---

NSLKALLEHEDTV-----------------------------------------EETFMM

NFMISYID----------------------------VFGNTITHDLKENG----AQIPVT

VENR--KEFVDLYADFILNRS-----IEKQFREFRRG---------------FDMVT---

--DESPLRNWFRPDEVELLVCGSKN--------------------------FDFNELEKA

TEYDG---------------GYTSTSPTIRYFWEIVH-SMDEDQ----KRKLLMFTTGSD

RVPVGGLAKLRLIL----AKNGPD---------------------------------SDR

LP-TSHTCFN-----VLLLPE-YSSKAK--LEERLLKAITHA-KGF

>Branchiostoma_floridae_XM_002227105.1 .

--------------------------------------DLK-KQLYVEFE-----GEQGI

---DEGGVSKEFFQLVVEEIFNA-------------------------------------

------------------------------------------------------------

------------------------------------------------------------

------------------------------------------------------------

------------------------------------------------------------

--------------------DYGMFIYDEQT-----------------------------

------------HMYWFNPSS------------FETDAQFKLIG--IVLGLAIYNN----

-----VILDVHFPM-VVYRKLMAKK-----------------------------------

----------------------------------------GTFLDLQ---DSHPVLY---

QSLKSMLEYEGDV-----------------------------------------EEDXMA

TFCVGMSD----------------------------LFGGSSTHELKENG----NNVPVT

VDNR--QEYVDLYADYILNKS-----VEKQFNAFRQG---------------FHMVT---

--DESPLEEFFRPEEIELLICGCPD--------------------------FDFKALEES

TDYDG---------------GFTRDSQTVKDFWELVH-GFSEDE----KKQLLMFATGSD

RVPVGGLSKLKLVI----ARNGPD---------------------------------SDR

LP-TSHTCFN-----VLLLPE-YSSKDK--LKERLLKAITHA-KGF

>Tetraodon_nigroviridis_CAG05672.1 .

LKVRRDHIIDDALVQLELISTEK-------------PSDLK-KQLFIEFE-----GEQGF

---DEGGLSKEFFQLVLEEIFNP-------------------------------------

------------------------------------------------------------

------------------------------------------------------------

------------------------------------------------------------

------------------------------------------------------------

--------------------DIGMFTYDDDTR----------------------------

-------------VFWFNSSS------------LENEAQYTLIG--LVLGLAIYNN----

-----CILDLHFPM-IVYKKLMGKK-----------------------------------

----------------------------------------GTFSDLS---DSHPVLS---

RSLKSLLEYPGNV-----------------------------------------EEDMSL

SFQISHTD----------------------------MFGSPVFYDLKENG----EQIPVT

RDNR--QEFVNLYTDYMLNKS-----VETQFRAFKKG----------------FLMVT--

--QESPLKHLFRPEELELLICGSKVRITPSCPSPPFFSDSQRNPVGGFLQKLDFGALEET

TEYDG---------------GYSKDTPIIKDFWETIR-AFEEEQ----KRLFLQFISGTV

RAPVGGLGKLKMTI----AKNGSDTDSDVLYLP-------------------------TG

CP-TSHTCYN-----ILLLPE-YSSKAK--LRERLLKAITYA-KGF

>Salmo_salar_BT044049.1 .

LKVRRDHIIDDALVRLEMISMEN-------------PADLR-KQLFVEFE-----GEQGV

---DEGGVSKEFFQLVLEEMFNP-------------------------------------

------------------------------------------------------------

------------------------------------------------------------

------------------------------------------------------------

------------------------------------------------------------

--------------------DIGMFTYDEST-----------------------------

------------KLFWYNPSS------------LENEAQFTLIG--IVLGLAIYNN----

-----CILDVHFPM-VVYRKLMGKK-----------------------------------

----------------------------------------GTYLDLA---DSHPVLY---

QSLKELLDYEGDV-----------------------------------------EEDMMI

TFQISQTD----------------------------LFGEPITYDLKENG----DKIPVT

ADNR--KEFVSLYGDYILNKS-----VERQFKAFRRG---------------FQMVT---

--NESPLKCLFRPEEVELLICGSRN--------------------------LDFQALEET

TEYDG---------------GYSKDCRIIKDFWETVH-SFGEED----KRLFLQFTTGTD

RAPVGGLGKLKMII----AKNGPD---------------------------------TDR

LP-TSHTCFN-----ALLLPE-YDSKEK--LKERLLKAITYA-KGF

>Danio_rerio_BC155156.1 .

LKVRRDHIIDDALVRLEMIAMEN-------------PADLK-KQLYVEFE-----GEQGV

---DEGGVSKEFFQLVVEEIFNP-------------------------------------

------------------------------------------------------------

------------------------------------------------------------

------------------------------------------------------------

------------------------------------------------------------

--------------------DIGMFTYDEST-----------------------------

------------KLFWFNPSS------------FENEGQFTLIG--IVLGLAIYNN----

-----CILDVHFPM-VIYRKLMGKK-----------------------------------

----------------------------------------GTFRDLA---DSHPVLF---

QSLKDLMEYEGNV-----------------------------------------EEDMMI

TFQISQTD----------------------------LFGNPLMYDLKESG----DKIPVT

NENR--KDFVALYAEYMLNKS-----VEKQFKAFRRG---------------FHMVT---

--NESPLKYLFRPEEIELLICGSRN--------------------------LDFQALEES

TEYDG---------------GYNKDSRIIRDFWETVH-SFEQEK----KRLFLQFTTGTD

RAPVGGLGKLKMII----AKNGPD---------------------------------SDR

LP-TSHTCFN-----VLLLPE-YSTVEK--LKERLLKAITYA-KGF

>Tetraodon_nigroviridis_CAG06618.1 .

LKVRRDHIIDDALVRLEMTAMEN-------------PADLK-KQLYVEFE-----GEQGV

---DEGGVSKEFFQLVVEEIFNP-------------------------------------

------------------------------------------------------------

------------------------------------------------------------

------------------------------------------------------------

------------------------------------------------------------

--------------------DIGMFTYDERTK----------------------------

-------------LFWFNPSS------------FENEGQYTLIG--IVLGLAIYNN----

-----CILDVHFPM-VVYRKLMGKK-----------------------------------

----------------------------------------GTFRDLA---DANPVLY---

QSLKELLEYEGSV-----------------------------------------EEDMMI

TFQISQTD----------------------------LFGNPLMYDLRENG----DKIPVT

NENR--KEFVSQYADYMLNKS-----VEKQFKAFRRG---------------FHMVT---

--NESPLKYLFRPEEIELLICGSRK--------------------------LDFLALEET

TEYDG---------------GYNRDSRIIKEFWETLH-SFGEEQ----KRLFLQFTTGTD

RAPVGGLGKLKMII----AKNGPD---------------------------------TDR

LP-TSHTCFN-----VLLLPE-YSSKEK--LRERLLKAITYA-KGF

>Xenopus_tropicalis_NM_001001213.1 .

LKVRRDHIIDDALVRLEMIAMEN-------------PADLK-KQLYVEFE-----GEQGV

---DEGGVSKEFFQLVVEEIFNP-------------------------------------

------------------------------------------------------------

------------------------------------------------------------

------------------------------------------------------------

------------------------------------------------------------

--------------------DIGMFTYDEST-----------------------------

------------KHSWFNPSS------------FETEGQFTLIG--IVLGLAIYNN----

-----CILDVHFPM-VVYRKLMGKK-----------------------------------

----------------------------------------GTFRDLA---DSHPVLY---

QSLKELLEYEGNV-----------------------------------------EEDMMM

TFQISQTD----------------------------LFGNPLMHDLKENG----DKIPIT

NENR--KEFVSLYTDYILNKS-----VEKQFKAFRRG---------------FHMVT---

--NESPLKYLFRPEEIELLICGSRN--------------------------LDFQALKDT

TEYDG---------------GYTRDSNIIKEFWEIVN-SFTEEQ----KRLFLQFTTGTD

RAPVGGLGKLKMII----AKNGPD---------------------------------TDR

LP-TSHTCFN-----VLLLPE-YSNKEK--LKERLLKAITYA-KGF

>Xenopus_laevis_NM_001087224.1 .

LKVRRDYIIDDALVRLEMIAMEN-------------PADLK-KQLYVEFE-----GEQGV

---DEGGVSKEFFQLVVEEIFNP-------------------------------------

------------------------------------------------------------

------------------------------------------------------------

------------------------------------------------------------

------------------------------------------------------------

--------------------DIGMFTYDEST-----------------------------

------------KLFWFNTSS------------LETEGQFTLIG--IVLGLAIYNN----

-----CILDVHFPM-VVYRKLLGKK-----------------------------------

----------------------------------------GTFQDLA---DSHPVLY---

QSLKELLEYEGSV-----------------------------------------EDDMMM

TFQISQTD----------------------------LFGNPLMHDLKENG----DKIPIT

NENR--KEFVNLYTDFILNKS-----AEKQFKAFRRG---------------FHMVT---

--NESPLKYLFRPEEIELLICGSRN--------------------------VDFQALKDT

TEYDG---------------GYTRDSNIIKEFWEIVN-SFTEEQ----KRLFLQFTTGTD

RAPVGGLGKLKMII----AKNGPD---------------------------------TDR

LP-TSHTCFN-----VLLLPE-YSSKAK--LKERLLKAITYA-KGF

>Taeniopygia_guttata_XM_002197187.1 .

LKVRRDHIIDDALVRLEMIAMEN-------------PADLK-KQLYVEFE-----GEQGV

---DEGGVSKEFFQLVVEEIFNP-------------------------------------

------------------------------------------------------------

------------------------------------------------------------

------------------------------------------------------------

------------------------------------------------------------

--------------------DIGMFTYDEST-----------------------------

------------KLFWFNPSS------------FETEGQFTLIG--IVLGLAIYNN----

-----CILDVHFPM-VVYRKLMGKK-----------------------------------

----------------------------------------GTFRDLA---DSHPVLY---

QSLRDLLEYEGSV-----------------------------------------EDDMMI

TFQISHTD----------------------------LFGNPMMHDLKENG----DKIPIT

NENR--KEFVNLYADYILNKS-----VEKQFKAFRRG---------------FHMVT---

--NESPLKYLFRPEEIELLICGSRN--------------------------LDFQALEET

TEYDG---------------GYTRDSLIIREFWEIVH-SFTDEQ----KRLFLQFTTGTD

RAPVGGLGKLKMII----AKNGPD---------------------------------TER

LP-TSHTCFN-----VLLLPE-YSSKEK--LKERLLKAITYA-KGF

>Mus_musculus_U96636.1 .

LKVRRDHIIDDALVRLEMIAMEN-------------PADLK-KQLYVEFE-----GEQGV

---DEGGVSKEFFQLVVEEIFNP-------------------------------------

------------------------------------------------------------

------------------------------------------------------------

------------------------------------------------------------

------------------------------------------------------------

--------------------DIGMFTYDEAT-----------------------------

------------KLFWFNPSS------------FETEGQFTLIG---ILGLAIYNN----

-----CILDVHFPM-VVYRKLMGKK-----------------------------------

----------------------------------------GTFRDLG---DSHPVLY---

QSLKDLLEYEGSV-----------------------------------------EDDMMI

TFQISQTD----------------------------LFGNPMMYDLKENG----DKIPIT

NENR--KEFVISYSDYILNKS-----VEKQFKAFRRG---------------FHMVT---

--NESPLKYLFRPEEIELLICGSRN--------------------------LDFQALEET

TEYDG---------------GYTRESVVIREFWEIVH-SFTDEQ----KRLFLLFTTGTD

RAPVGGLGKLKMII----AKNGPD---------------------------------TER

LP-TSHTCFN-----VLLLPE-YSSKEK--LNVRLLKAITYA-KGF

>Rattus_norvegicus_XM_001055915.1 .

LKVRRDHIIDDALVRLEMIAMEN-------------PADLK-KQLYVEFE-----GEQGV

---DEGGVSKEFFQLVVEEIFNP-------------------------------------

------------------------------------------------------------

------------------------------------------------------------

------------------------------------------------------------

------------------------------------------------------------

--------------------DIGMFTYDEAT-----------------------------

------------RLFWFNPSS------------FETEGQFTLIG--IVLGLAIYNN----

-----CILDVHFPM-VVYRKLMGKK-----------------------------------

----------------------------------------GTFCDLG---DSHPILY---

QSLKDLLEYEGNV-----------------------------------------EDDMMI

TFQISQTD----------------------------LFGNPMMYDLKENG----DKIPIT

NENR--KEFVSLYSDYILNKS-----VEKQFKAFRRG---------------FHMVT---

--NESPLKYLFRPEEIELLICGSRN--------------------------LDFQALEET

TEYDG---------------GYTRESVVIREFWEIVH-SFTDEQ----KRLFLQFTTGTD

RAPVGGLGKLKMII----AKNGPD---------------------------------TER

LP-TSHTCFN-----VLLLPE-YSSKEK--LKERLLKAITYA-KGF

>Pan_troglodytes_DQ045240.1 .

LKVRRDHIIDDALXXLEMIAMEN-------------PADLK-KQLYVEFE-----GEQGV

---DEGGVSKEFFQLVVEEIFNP-------------------------------------

------------------------------------------------------------

------------------------------------------------------------

------------------------------------------------------------

------------------------------------------------------------

--------------------DIGMFTYDEST-----------------------------

------------KLFWFNPSS------------FETEGQFTLIG--IVLGLAIYNN----

-----CILDVHFPM-VVYRKLMGKK-----------------------------------

----------------------------------------GTFRDLG---DSHPVLY---

QSLKDLLEYEGNV-----------------------------------------EDDMMI

TFQISQTD----------------------------LFGNPMMYDLKENG----DKIPIT

NENR--KEFVNLYSDYILNKS-----VEKQFKAFRRG---------------FHMVT---

--NESPLKYLFRPEEIELLICGSRN--------------------------LDFQALEEX

TEYDG---------------G----------FWEIVH-SFTDEQ----KRLFLQFTTGTD

RAPVGGLGKLKMII----AKNGPD---------------------------------TER

LP-TSHTCFN-----VLLLPE-YSSKEK--LKXXXLKAI-------

>Bos_taurus_NM_001098462.1 .

LKVRRDHIIDDALVRLEMIAMEN-------------PADLK-KQLYVEFE-----GEQGV

---DEGGVSKEFFQLVVEEIFNP-------------------------------------

------------------------------------------------------------

------------------------------------------------------------

------------------------------------------------------------

------------------------------------------------------------

--------------------DIGMFTYDEST-----------------------------

------------KLFWFNPSS------------FETEGQFTLIG--IVLGLAIYNN----

-----CILDVHFPM-VVYRKLMGKK-----------------------------------

----------------------------------------GTFLDLG---DSHPVLY---

QSLKDLLEYEGNV-----------------------------------------EDDMMI

TFQISQTD----------------------------LFGNPMMYDLKENG----DKIPIT

NENR--KEFVNLYSDYILNKS-----VEKQFKAFRRG---------------FHMVT---

--NESPLKYLFRPEEIELLICGSRN--------------------------LDFQALEET

TEYDG---------------GYTRDSVVIREFWEIVH-SFTDEQ----KRLFLQFTTGTD

RAPVGGLGKLKMII----AKNGPD---------------------------------TER

LP-TSHTCFN-----VLLLPE-YSSKEK--LKERLLKAITYA-KGF

>Homo_sapiens_L07557.1 .

LKVRRDHIIDDALVRLEMIAMEN-------------PADLK-KQLYVEFE-----GEQGV

---DEGGVSKEFFQLVVEEIFNP-------------------------------------

------------------------------------------------------------

------------------------------------------------------------

------------------------------------------------------------

------------------------------------------------------------

--------------------DIGMFTYDEST-----------------------------

------------KLFWFNPSS------------FETEGQFTLIG--IVLGLAIYNN----

-----CILDVHFPM-VVYRKLMGKK-----------------------------------

----------------------------------------GLFVDLG---DSHPVLY---

QSLKDLLEYVGNV-----------------------------------------EDDMMI

TFQISQTN----------------------------LFGNPMMYDLKENG----DKIPIT

NENR--KEFVNLYSDYILNKS-----VEKQFKAFRRG---------------FHMVT---

--NESPLKYLFRPEEIELLICGSRN--------------------------LDFQALEET

TEYDG---------------GYTRDSVLIREFWEIVH-SFTDEQ----KRLFLQFTTGTD

RAPVGGLGKLKMII----AKNGPD---------------------------------TER

LP-TSHTCFN-----VLLLPE-YSSKEK--LKERLLKAITYA-KGF

>Equus_caballus_XM_001917171.1 .

LKVRRDHIIDDALVRLEMIAMEN-------------PADLK-KQLYVEFE-----GEQGV

---DEGGVSKEFFQLVVEEIFNP-------------------------------------

------------------------------------------------------------

------------------------------------------------------------

------------------------------------------------------------

------------------------------------------------------------

--------------------DIGMFTYDEST-----------------------------

------------KLFWFNPSS------------FETEGQFTLIG----------------

--------DVHFPM-VVYRKLMGKK-----------------------------------

----------------------------------------GTFRDLG---DSHPVLY---

QSLKDLLEYEGNV-----------------------------------------EDDMMI

TFQISQTD----------------------------LFGNPMMYDLKENG----DKIPIT

NENR--KEFVNLYSDYILNKS-----VEKQFKAFRRG---------------FHMVT---

--NESPLKYLFRPEEIELLICGSRN--------------------------LDFQALEET

TEYDG---------------GYTRDSVLIREFWEIVH-SFTDEQ----KRLFLQFTTGTD

RAPVGGLGKLKMII----AKNGPD---------------------------------TER

LP-TSHTCFN-----VLLLPE-YSSKEK--LKERLLKAITYA-KGF

>Macaca_fascicularis_AB179374.1 .

LKVRRDHIIDDALVRLEMIAMEN-------------PADLK-KQLYVEFE-----GEQGV

---DEGGVSKAFFQLVVEEISNP-------------------------------------

------------------------------------------------------------

------------------------------------------------------------

------------------------------------------------------------

------------------------------------------------------------

--------------------DIGMFTYDEST-----------------------------

------------KLFWFNPSS------------FETEGQFTLIG--IVLGLAIYNN----

-----CILDVHFPM-VVYRRLMGKK-----------------------------------

----------------------------------------GTFRDLG---DSHPVLY---

QSLKDLLEYEGNV-----------------------------------------EDDMMI

TFQISQTD----------------------------LFGNPMMYDLKENG----DKIPIT

NENR--KEFVNLYSDYILNKS-----VEKQFKAFRRG---------------FHMVT---

--NESPLKYLFRPEEIELLICGSRN--------------------------LDFQALEET

TEYDG---------------GYTRDSVLIREFWEIVH-SFTDEQ----KRLFLQFTTGTD

RAPVGGLGKLKMII----AKNGPD---------------------------------TER

LP-TSHTCFN-----VLLLPE-YSSKEK--LKERLLKAITYA-KGF

>Acyrthosiphon_pisum_XM_001948420.1 .

LKVRREHVVQDALVELEMVAMQN-------------PSDLK-KQLAVEFD-----GEQGV

---DEGGVSKEFFHLIVEELFNP-------------------------------------

------------------------------------------------------------

------------------------------------------------------------

------------------------------------------------------------

------------------------------------------------------------

--------------------DYGMFVIMNSDSGN--------------------------

------------PTYWFNSFS------------FETAAQYSLIG--LIVGLAIYNN----

-----VILNINLPM-VVYRKLLGKR-----------------------------------

----------------------------------------CTFNDLQ---DWNQELY---

NGLKNLLDYEEDDI----------------------------------------EEMFMQ

TFRICYKD----------------------------AFGEIVYHDLIDNG----DNITVN

HINK--REFVDKYADFLLNES-----IGVQFNAFYRG---------------FQNVM---

--EESPLQYLFWPEELEQIVCGSKV--------------------------FDMSELEET

TLYEG---------------GYHKETQVIKYFWDFAH-ALPRVS----QQKLLQFTTGSD

RVPVGGLGKLKLTI----TRNGSD---------------------------------SDR

LP-TAHTCFN-----VLLLPE-YSSKEK--LDERLSKAINYA-EGF

>Hydra_magnipapillata_XM_002161124.1 .

LVVRRDHLIQDSLVGLEMVAQDE-------------PEDFK-KQFFVEFE-----GEQGI

---DEGGVSKEFFQLIIEELFNP-------------------------------------

------------------------------------------------------------

------------------------------------------------------------

------------------------------------------------------------

------------------------------------------------------------

--------------------DFGMFTLNETT-----------------------------

------------RTYWFHPTS------------FEAASQFMLIG--VLFGIAIYNS----

-----IILDVRFPA-VLYRKLLGYS-----------------------------------

----------------------------------------GKFEDLS---SSHPEIY---

FSLNQLLSYNGNV-----------------------------------------KEDIMA

SFQISYSD----------------------------MFGSTVSHELIEGG----KDIEVT

NQNR--EEYVALYADFILNTS-----VKQQFDAFKKG---------------FDIVT---

--SHSILKKIFRPDELELLVCGSQI--------------------------YDINELENA

TEYDG---------------GYSKDTPVIRYFWQVIH-EMSSDQ----AKQFLNFTTGSD

RVPVGGLSKLKLII----AKNGED---------------------------------SDR

LP-TAHTCFN-----VLLLPK-YSSVDK--LRERLLKAISNA-KGF

>Trichoplax_adhaerens_XM_002117520.1 .

IRVRRDHLIEDALWQLEHVSDDN-------------PSALL-KQLRIKFV-----GEEGV

---DDGGLSKEFFVLINQEIFNP-------------------------------------

------------------------------------------------------------

------------------------------------------------------------

------------------------------------------------------------

------------------------------------------------------------

--------------------EYAMFRYNEKA-----------------------------

------------GTYWFTPDS------------YETDAQYKLVG--ILVGLAIYNG----

-----IILDVTFPM-ALYRKLYGQK-----------------------------------

----------------------------------------LILEDLS---DCFPEIW---

QSLVNLLNYEGDV-----------------------------------------ENDFMY

TFKISYTN----------------------------VFGDAVTVDLVENG----GEISVN

KNNR--EEFIRLYTEFLLTNS-----VKNQFDAFKKG---------------FNMVT---

--EDSLLKYLFRPDEVDMLVCGSKC--------------------------LDFHELERV

TYYDG---------------GLTKDSTLVKDLWEVVH-SFDEES----KRKFLKFTTGSD

RVPVGGLSKIMFRV----IKNGDD---------------------------------STR

LP-TSHTCFN-----VLMLCN-YSSKAK--LKERLLYAINNG-NA-

>Schistosoma_mansoni_CAZ35820.1 .

LRVHRDRIVEDALLILEIAFMEN-------------PGDFK-KQLLIEFD-----GEQGI

---DEGGLSKEFFQLIIERIFNP-------------------------------------

------------------------------------------------------------

------------------------------------------------------------

------------------------------------------------------------

------------------------------------------------------------

--------------------DYGMFVLDEET-----------------------------

------------QNYWFNPVP-----------LDDMEREYCLIG--TLLGLAIYND----

-----VILDVNFPS-VLYRKLVGKLG----------------------------------

-----------------------------------------TFEDLF---DARPSLA---

QGLKSLLEYEHDDI----------------------------------------ENVFGC

SFSVNYLD----------------------------PFGNVVTHELKPDG----ATIPVT

KENR--KEYVDLYSSFLLNDS-----VKKQFNAFRRG----------------FQMVV--

--DESPLTFLFRPDELELLVRGSPV--------------------------YDFNELERV

TTYEE----------------YTSDSAVIKNFWSVVH-SMTEEQ----KKQLLQFSTGSD

RVPVGGMSKMKFTI----ARQGAD---------------------------------TNR

LP-SAHTCFN-----ILLLPE-YQSLEK--LQQSLLLAITHC-KGF

>Ciona_intestinalis_XM_002128371.1 .

IQVRRAHIINDTLVQLEM-ATEN-------------LKNLR-KQLYVEFD-----GEEGA

---DEGGVSKEFFALMVEEIFNP-------------------------------------

------------------------------------------------------------

------------------------------------------------------------

------------------------------------------------------------

------------------------------------------------------------

--------------------ANGTFILVEES-----------------------------

------------QLFWFNPES------------FEGQMQYKLIG--IILGLAIYNN----

-----CILDIKFPA-FLYKKLLGRR-----------------------------------

----------------------------------------AKFRDIK---DIYPVVY---

RSLCDILSYSGNV-----------------------------------------EQDMML

TFRVEYSD----------------------------IYGCPHTHDLKKEG----NKIPVT

NSNR--QEYVDLYSDWLLNTS-----VEEQFNAFLGG---------------FELVL---

--SKSPLKYLFKPKEVELLLCGSEH--------------------------YDFKELEDS

ADYDG---------------GFTRDSQTVKDFWSVVH-ELSEEE----QKTLLQFSTGSD

RAPVGGLSKLKMIL----ARNGPD---------------------------------SDR

LP-TAHTCFN-----VILLPD-YKNKIK--LRERLLKAIKYS-KGF

>Brugia_malayi_XM_001896201.1 .

LNVRRDNIIRDALDSVRFFSLVSIKLAAIAIDN---SANFK-KQLRIQFD-----GEQAV

---DEGGVSKEFYQLITDELFCP-------------------------------------

------------------------------------------------------------

------------------------------------------------------------

------------------------------------------------------------

------------------------------------------------------------

--------------------DYGMFILNEKT-----------------------------

------------GLYWFNSQC------------NFCDDEFGLIG--LLFGLAIYNN----

-----ILIDVRFPT-LVYVKLLARP-----------------------------------

----------------------------------------AVFDELA---QIDSELY---

SGLRQLLECNDDV-----------------------------------------ENIYNY

TFQISYKD----------------------------VYGCSHDEELIPNG----ANIPVT

LANK--KKFVACYADFLLNRS-----VKRQFDAFSMG---------------FNKVV---

--NRGLLRRLFMPDEVEQLVCGVLD--------------------------LDFDILAQC

TKYQN---------------GFTETSQTIKDFWAVAK-AMNTEE----KKMLLQFITGSD

RVPVGGLAKLEVII----ARNGDN---------------------------------KER

LP-TAHTCYN-----VMLLPD-YGDLEI--TRERISKAISYS-RGF

>Monosiga_brevicollis_XM_001741995.1 .

-------------------------------------------------S-----GEQGL

---DLGGLQKELLKQVWEDVSNP-------------------------------------

------------------------------------------------------------

------------------------------------------------------------

------------------------------------------------------------

------------------------------------------------------------

--------------------EHGLFIMHDDS-----------------------------

------------RFLWFQTSL-----------PTVPARHFELCG--ILLGLALFNG----

-----IMLNLHFPA-VFFSLLLGHE-----------------------------------

----------------------------------------TGLEDFA---SLFPVQA---

QSLQMLLDYDGD------------------------------------------VSEWTQ

TFSIMRKT----------------------------LDESYEEVDIVPNG----RNIEVT

NANR--QQFVDAMVQYYLHDA-----IEHCLEPFRRG---------------FLGVC---

--GCPMLY-VLTAAELESLLVGART--------------------------WNIADLRAG

ATYED---------------QYTENHPTIVDFWEVIF-GMTQEE----QRQFVEFVTXSD

REPVGGLGSLRLCV----QRNGPD---------------------------------SDR

LP-TSLTCFN-----RLLLPE-YDNIDK--LRSRLLTAIQHS-KGF

>Hydra_magnipapillata_XM_002165082.1 .

LEIRRQNIVDDTLKQLRNK-----------------FSDFK-KPLKIKYTEG---GEQGL

---DMGGLQKEFFQVIIETMFDP-------------------------------------

------------------------------------------------------------

------------------------------------------------------------

------------------------------------------------------------

------------------------------------------------------------

--------------------NYGLFTLSEDS-----------------------------

------------NLMWFNALC------------LESVVMFNLVG--ILLGLAIYNG----

-----IILDVHFPL-LVYKKLLGYE-----------------------------------

----------------------------------------VGLEDLR---EIQPTLC---

KSLDELLSYEGDV-----------------------------------------EIDFGL

TFEVFHNL-----------------------------YGKEMRTELLTNG----SQISVT

NSNR--ESFVRLYVDLIINKS-----IENSFSAFKEG---------------FFQVC---

--HFPSI-SLFTAPELELLICGSPD--------------------------LDFKALEKV

TEYKD---------------GFSKSHPLMLEFWDIVH-RFTFKQ----KQALLMFVTGSY

RVPLKGLGSMSFYI----QRNGPD---------------------------------SLN

LP-TSMTCFN-----RLLIPE-YSSAAK--LEKMLLLAIENS-KGF

>Nematostella_vectensis_XM_001632315.1 .

LEVRRDHLIRDTLSQIHLK-----------------EYDLK-KPLKIKYVGG---GELGL

---DMGGLQKEFFHMIVDSIFEP-------------------------------------

------------------------------------------------------------

------------------------------------------------------------

------------------------------------------------------------

------------------------------------------------------------

--------------------GYGMFTYIEES-----------------------------

------------RTMWINGES------------FESLKEFELVG--IVLGLAIYNG----

-----IILDIHFPQ-VIYKKLQGQT-----------------------------------

----------------------------------------PSLEDLI---GVQPSLG---

NSLKQLLEYEGDV-----------------------------------------EDTFCY

TFQVSAMS-----------------------------YGKVCDRELIPNG----AEVPVN

NENR--EEFVHRYVKYLLVDS-----VAKQFDAFSAG---------------FHKVC---

--GGSAL-TLFCADELELLICGHHV--------------------------LDFSDLVLA

ASYDD---------------GYSAQHSTIKMFWNVFN-EMTNHQ----KKALLMFVTGSD

RVPLKGLSNLTFII----QKHGQD---------------------------------SDR

LP-TAMTCFN-----RLLLPE-YTSPKR--LKERLIVAIENS-KGF

>Trichoplax_adhaerens_XM_002112430.1 .

LEVRRDYLIEDTMQQIRSK-----------------ESDLK-KPLKIKYVGG---GEQGL

---DMGGLQKEFFHLIVDCIFDP-------------------------------------

------------------------------------------------------------

------------------------------------------------------------

------------------------------------------------------------

------------------------------------------------------------

--------------------SYGMFTYIEES-----------------------------

------------RKFWIDSAS------------LESEREFELVG--IILGLAIYNG----

-----VILDIHFPQ-SIYKKLQGEQ-----------------------------------

----------------------------------------LHLKDLI---DVQPTLG---

ASLQELLEYDGDV-----------------------------------------EDIFCF

TFEISYMS-----------------------------LGRMVDVELKPRG----SLIPVT

NENR--EEFVNLYIQHLLVNS-----IEKQFQPFSRG---------------FHKVC---

--GGDTL-TLFRAEELDLVICGSNE--------------------------LDFDSLERT

ANYDG----------------YTCTSVIIVHFWELVH-AMNEKH----KKLLLMFVTGSD

RAPLKGLGNLKIII----QRNGGD---------------------------------SNR

LP-TAMTCFN-----RLLLPD-YKNKNK--LEKLLLLAIENG-KGF

>Branchiostoma_floridae_XM_002214193.1 .

LEIRREQLIQDTLAEVQLK-----------------RDQLK-KPLKIKYIGG---GEQGL

---DMGGLQKEFFQMITESVFDP-------------------------------------

------------------------------------------------------------

------------------------------------------------------------

------------------------------------------------------------

------------------------------------------------------------

--------------------NYGMFVYLEES-----------------------------

------------RSLWINGES------------PESDGEFELVG--IILGLAIYNG----

-----VILDVHFPM-TVYKKLQGET-----------------------------------

----------------------------------------LELADLQ---DIQPILA---

SGLQELLDYEGDV-----------------------------------------EMDLCY

TFQVSYES-----------------------------FGHVKTVDLIENG----SEVPVN

NKNR--GEFVRRYVNFLLVDS-----VERQFEAFSRG---------------FHLVC---

--GGRVL-TLFRAEEIELLICGSTE--------------------------LDFDGLEAS

AVYED---------------GYSKDHKTVRALWSVVR-SLSHKH----KKMLLMFITGSD

RVPLKGLATLRITV----QRHGPD---------------------------------SER

LP-TAMTCFN-----TLLLPS-YKDIDK--LKNRLLTAIENC-KGF

>Strong._purpuratus_XM_001180785.1 .

LEVRRDYLISDTLAQIRLK-----------------KNDLK-KPLKIKYIGG---GEQGL

---DMGGLQKEFFQLISEAVFNP-------------------------------------

------------------------------------------------------------

------------------------------------------------------------

------------------------------------------------------------

------------------------------------------------------------

--------------------SYGMFVSSSEN-----------------------------

------------RTIWINGSE----------GSTDSDDEFELVG--TLLGLAIYNG----

-----IILDVNFPM-AIYKKLHEDP-----------------------------------

----------------------------------------MTLEDLI---GVQPSLG---

RGIREMLEYEGDV-----------------------------------------EDVFCQ

TFQVSYLS-----------------------------MGDILTVDLVLHG----SQIPVT

NQNQ--EEYARLYVKHLLIDS-----IARQFEAFARG---------------FHSVC---

--GGSAL-QLVQPSEIELLICGSPV--------------------------LDFHALETS

ATYED---------------FFSRKHPTVLSLWRLIH-SLSNEQ----KKKLLNFITGSD

RVPLKGLSSLPIVI----QRNGPD---------------------------------SER

LP-TAMTCFN-----RLLLPE-YKDEKK--LRERLLVAVQYG-KGF

>Hydra_magnipapillata_XM_002159674.1 .

LNVRRKFILEDSLSEISKN-----------------HGDLR-KKLRVIFQ-----GEPAV

---DLGGVSKEWFFLIIQKLFNE-------------------------------------

------------------------------------------------------------

------------------------------------------------------------

------------------------------------------------------------

------------------------------------------------------------

--------------------DYGMFKYNSET-----------------------------

------------KLWWFNASC------------KENYKEFNLCG--VLIGLAMYNG----

-----INLNIGFPP-CLYKKLLSPAVVPY-------------------------------

--------------------------------NNPHALVGVAPMDIEEFKQVYPDLA---

NGLKELLFYEGNV-----------------------------------------EEDLCQ

TFQVSFTE-----------------------------YGVVQTRILKPNG----ESIPVT

NDNK--KEYVSLYIDYFMNKC-----IYQQFYSFYHG---------------FHSVC---

--ASNAL-LLLRPDEVETLVIGDAD--------------------------FNIRDLEKV

TKYDG----------------YRYSDTVIRNLWDVLY-SYSQKQ----QRKFLFFCTGSD

RIPIGGVKEINFKV----TKVSGANA-------------------------------TQM

LP-VAHTCFN-----QLCLPM-YKGRKT--LAKKLTIAIENG-EGF

>Trichoplax_adhaerens_XM_002117456.1 .

LTVRRSHIVSDSLNEIANK-----------------QDQLK-KKITVTFI-----GEPGL

---DMGGLTKEWFLLLIRQIFRS-------------------------------------

------------------------------------------------------------

------------------------------------------------------------

------------------------------------------------------------

------------------------------------------------------------

--------------------EYGMFSYNKIT-----------------------------

------------QNFWFTMGR------------SGNLMEYNLIG--VLMGLAVYNS----

-----IVLDIRFPR-PCYKKLLALPSGHSNRP----------------------------

--------------------------------QTRVGMAHLNLNDLQ---QVFPDLA---

HGLQELLDYNGNV-----------------------------------------EEDFCY

TFQLSYQS-----------------------------FERVNTHILKPNG----NKIPVT

NKNR--YEYVQLYVDFMMNRI-----IYHQFSSFYRG---------------FHTVC---

--TSNSL-TFLKAEELEILICGYPN--------------------------FDITELMAI

TQYEG----------------YTKDNRVIKNFWDVAL-QLSKNK----QRQLLSFVTGSD

RIPVGGITEMNFKI----LYVEN----------------------------------TDY

LP-TSQTCFN-----QLCLPP-YKSKHI--LKKKLLIALANS-KGF

>Acyrthosiphon_pisum_XM_001945592.1 .

INVRRSHLVQDSLNEIAFK-----------------QKDLK-KKLKVTFA-----GEPGL

---DMGGLTKEWFLLLIREIFHA-------------------------------------

------------------------------------------------------------

------------------------------------------------------------

------------------------------------------------------------

------------------------------------------------------------

--------------------EYGMFVYYSHS-----------------------------

------------RCYWFSTGQ----------TDHTNLREYNLIG--VLMGLAVYNS----

-----IILDLSFPG-ICYRKLLSPPVVPTVND----------------------------

--------------------------------DRVGVVENPTLDDLN---EIMPDVA---

SGLKHLLEYEGNV-----------------------------------------EEDMGL

TFQVSLEE-----------------------------HRTPKTHKLKPNG----ENIPVT

NESR--EEYINLYLNWVLNLS-----IYEQFRAFYFG---------------FHSVC---

--ASNAL-IMLRPEEVELLVCGTDT--------------------------LDLHELRKA

TEYDG----------------YRPDDNIISIFWNVID-SLSDDQ----KRKFLLFVTGSD

RVPVGGMGDMNFKI----TRGPNR---------------------------------SDY

LP-EAHTCFN-----QLVLPQ-YPDHDQ--LKEKLVTAILNA-EGF

>Nematostella_vectensis_XM_001626176.1 .

IKVRRSHLITDSLNEIARK-----------------QKNLK-KKLKVTFA-----GEPGL

---DMGGLTKEWFLLLVRKIFKP-------------------------------------

------------------------------------------------------------

------------------------------------------------------------

------------------------------------------------------------

------------------------------------------------------------

--------------------EYGMFSCNDKS-----------------------------

------------RLYWFNKEC------------LDQDEEFNLVG--VLMGLAVYNS----

-----IILDIHFPS-CCYKKLLSPAVVPFHNP----------------------------

--------------------------------NARVGLANLGLEDLA---EVMPELA---

RGLKELLSYEGDV-----------------------------------------ENDLCQ

TFQVSFTS-----------------------------YGEVVTHNLKPKG----DTIPVT

NTNR--QEYVQLYVDYLLNSS-----IYKQFEAFYHG---------------FHSVC---

--ASNAL-IMLRPEEVEMLVCGNPE--------------------------LDMEALKKV

TVYDG----------------YSKNDNTIRYFWDTVM-NFNTDL----KKKMLLFATGSD

RIPIGGMAEMEFKI----VRMDTSHS-------------------------------TSM

LP-MAHTCFN-----QLCLPP-YKTRKQ--IKQKLTIAISNA-EGF

>Strong._purpuratus_XM_001183672.1 .

LKVRRSHLVSDSLHEVANK-----------------KQDLK-KKLRVTFA-----GEPGL

---DMGGLTKEWFLLLLRKVFRE-------------------------------------

------------------------------------------------------------

------------------------------------------------------------

------------------------------------------------------------

------------------------------------------------------------

--------------------EYGMFTYFKKT-----------------------------

------------HCFWFNPAC------------TDCNQEFNLVG--VLMGLAVYNS----

-----IILDIRFPA-ITYKKLLSPAVVPYNKP----------------------------

--------------------------------QASVGRCKVTMEDLE---QVNPDLC---

HGLKELLKYDGDV-----------------------------------------EEDLCT

TFQCSLPV-----------------------------YGTVMTYDLKPNG----GDILVT

NKNR--REYVELYVNFLMNNS-----VYEQFAAFYHG---------------FHSVC---

--ASNAL-IMLRPEEVEMLVCGSPV--------------------------VDYEELEKV

TLYDG----------------FDKDDVTIKYFWEVAK-GYPLPL----QKKLLLFATGSD

RVPIGGMGEMSFKI----IRVDTS---------------------------------SNM

LP-MSHTCFN-----QLILPP-YKSKRQ--LKQKLTIAISNA-EGF

>Danio_rerio_XM_686363.3 .

IKVRRLQLVSDSLDELSRK-----------------RADLK-KKLKVTFV-----GEAGL

---DMGGLTKEWFLLLIRQIFHT-------------------------------------

------------------------------------------------------------

------------------------------------------------------------

------------------------------------------------------------

------------------------------------------------------------

--------------------DYGMFTYVKES-----------------------------

------------QCYWFSSWK------------CDNYSEFRLVG--ALMGLAVYNS----

-----ITLDIRFPP-CVYKKLLTPPIVPCDL-----------------------------

--------------------------------DTPVGMATLTLDDLQ---QIMPDLA---

HGLGELLSYEGNV-----------------------------------------EEDFYT

TFQVFQEE-----------------------------LGVVKAYNLKPGG----DKIPVT

NLNR--KEYVQLYIDFLLNKS-----IYRQFAAFYHG---------------FHSVC---

--ASNAL-MLLRPEEVEILVCGSPN--------------------------LDMGSLQRV

VQYEG----------------YSKTDPTIRAFWDVVL-AFPLEL----QKKLLHFTTGSD

RVPVGGMADLNFKI----SKIDVS---------------------------------TDW

LP-VSHTCFN-----QICLPP-YKSKKE--LRQKLTIAISNA-EGF

>Xenopus_tropicalis_NM_001112937.1 .

IKVRRLQLVSDSLDELTRK-----------------RADLK-KKLKVTFM-----GEAGL

---DMGGLTKEWFLLLIRQIFHP-------------------------------------

------------------------------------------------------------

------------------------------------------------------------

------------------------------------------------------------

------------------------------------------------------------

--------------------DYGMFTYHKDS-----------------------------

------------NCHWFSSFK------------CDNYSEFRLVG--TLMGLAVYNS----

-----ITLDIRFPP-CCYKKLLSPPIVPCDQ-----------------------------

--------------------------------NMPVGIAPVTLDDLY---QIMPELA---

HGLNELLCYDGNV-----------------------------------------EEDFYS

TFQVFQEE-----------------------------FGTIKSYNLKPGG----DKIPVT

NQNR--KEYVQLYVDFLLNKS-----IYKQFSAFYYG---------------FHSVC---

--ASNAL-MLLRPEEVEILVCGSPE--------------------------LDMHALQRS

TQYDG----------------YLKTDVTVRYFWDVVL-GFPLEL----QKKLLHFATGSD

RVPVGGMADLNFKI----SKSETP---------------------------------SNW

LP-IAHTCFN-----QLCLPP-YKTKKE--LKQKLTIAISNA-EGF

>Gallus_gallus_XM_424632.2 .

VKVRRTHLVSDSLDELTRK-----------------RADLK-KKLKVTFV-----GEAGL

---DMGGLTKEWFLLLIRQIFHP-------------------------------------

------------------------------------------------------------

------------------------------------------------------------

------------------------------------------------------------

------------------------------------------------------------

--------------------DYGMFTYHKDS-----------------------------

------------HCHWFSSFK------------CDNYSEFRLVG--ALMGLAVYNS----

-----ITLDIRFPP-CCYKKLLSPPIVPCDH-----------------------------

--------------------------------NTLVGICDVTLDDLF---QIMPELA---

HGLSELLSYEGNV-----------------------------------------EEDFYS

TFQVFQEE-----------------------------FGVIKSYNLKPNG----DKIPVT

NQNR--KEYVQLYVDFLLNKS-----IYKQFAAFYYG---------------FHSVC---

--ASYAL-LLLRPEEVEILVCGSPE--------------------------LDMSALQRS

TQYEG----------------YQKTDVTIRYFWDVVL-GFSLDL----QKKLLHFATGSD

RVPVGGMADLNFKI----SKSEAS---------------------------------TSW

LP-VAHTCFN-----QLCLPP-YKNKKE--LKQKLIIGISNA-EGF

>Taeniopygia_guttata_XM_002188939.1 .

VKVRRTHLVSDSLDELARK-----------------KADLK-KKLKVTFV-----GEAGL

---DMGGLTKEWFLLLIRQIFHP-------------------------------------

------------------------------------------------------------

------------------------------------------------------------

------------------------------------------------------------

------------------------------------------------------------

--------------------DYGMFTYHKDS-----------------------------

------------QCHWFSSFK------------CDNYSEFRLVG--ALMGLAVYNS----

-----ITLDIRFPP-CCYKKLLSPPIVPCDH-----------------------------

--------------------------------NTLVGICGVTLDDLF---QIMPELA---

HGLSELLSYEGNV-----------------------------------------EEDFYS

TFQVFQEE-----------------------------FGVIKSYNLKPNG----DKIPVT

NRNR--KEYVQLYVDFLLNRS-----IYKQFAAFYYG---------------FHSVC---

--ASYAL-LLLRPEEVEILVCGSPE--------------------------LDMSALQRS

TQYEG----------------YQKTDMTIRYFWDVVL-GFSLDL----QKKLLHFATGSD

RVPVGGMADLNFKI----SRSETS---------------------------------TNW

LP-VAHTCFN-----QLCLPP-YKNKKE--LKQKLIIGISNA-EGF

>Ornithorhynchus_anatinus_XM_001506929.1 .

MKVRRTHLVSDSLDELTRK-----------------TADLK-KKLKVTFV-----GEAGL

---DMGGLTKEWFLLLIRQIFHP-------------------------------------

------------------------------------------------------------

------------------------------------------------------------

------------------------------------------------------------

------------------------------------------------------------

--------------------DYGMFAYHKDS-----------------------------

------------HSHWFSGFK------------CDNYSEFRLVG--ILMGLAVYNS----

-----NNLDIHFPL-CCYKKLLSPPVVPCDS-----------------------------

--------------------------------NSPVGICGVSIDDLY---QIMPELA---

HGLSELLSYEGNV-----------------------------------------EEDFYS

TFQVFQEE-----------------------------FGVIKSYSLKPGG----DKIPVT

NQNR--KEYVQLYIDFLLNKS-----IYKQFAAFYYG---------------FHSVC---

--ASNAL-MLLRPEEVEILVCGSPQ--------------------------LDMHALQRN

TQYEG----------------YVKTDLTIRYFWDVVL-EFSLDL----QKKLLHFTTGSD

RVPVGGMADLNFKI----SKNETS---------------------------------TNW

LP-VAHTCFN-----QLCLPP-YKNKKE--LRQKLIIGISNS-EGF

>Monodelphis_domestica_XM_001375675.1 .

MKVRRTHLVSDSLDELTRK-----------------RADLK-KKLKVTFV-----GEAGL

---DMGGLTKEWFLLLIRQIFHP-------------------------------------

------------------------------------------------------------

------------------------------------------------------------

------------------------------------------------------------

------------------------------------------------------------

--------------------DYGMFAYHKDS-----------------------------

------------RCHWFSSFK------------CENYSEFRLVG--ILMGLAVYNS----

-----ITLDIRFPP-CCYKKLLSPPIVPGDQ-----------------------------

--------------------------------NTPVGICNVTIDDLY---QIMPELA---

HGLNELLSYDGNV-----------------------------------------EEDFYS

TFQVFQEE-----------------------------FGIIKSYNLKSAG----DKIPVT

NQNR--KEYVQLYVDFLLNKS-----IYKQFAAFYYG---------------FHSVC---

--ASNAL-MLLRPEEVEILVCGSPE--------------------------LDMHALQRN

TQYDG----------------YVKTDLTIRYFWDVVL-GFPLDL----QKKLLHFTTGSD

RVPVGGMADLNFKI----SKHETS---------------------------------TNW

LP-VAHTCFN-----QLCLPP-YKNKKE--LKQKLIIGISNS-EGF

>Mus_musculus_AK142777.1 .

MKVRRTHLVSDSLDELTRK-----------------RADLK-KKLKVTFV-----GEAGL

---DMGGLTKEWFLLLIRQIFHP-------------------------------------

------------------------------------------------------------

------------------------------------------------------------

------------------------------------------------------------

------------------------------------------------------------

--------------------DYGMFTYHKDS-----------------------------

------------HCHWFSSFK------------CDNYSEFRLVGIYSLMGLAVYNS----

-----ITLDIRFPP-CCYKKLLSPPVVPSDQ-----------------------------

--------------------------------STPVGICSVTIDDLC---QVMPELA---

HGLKELLSYEGNV-----------------------------------------EEDFYS

TFQVFQEE-----------------------------FGVIKSYNLKPGG----DKIPVT

NQNR--REYVQLYTDFLLNKS-----IYKQFAAFYCG---------------FHSVC---

--ASNAL-MLLRPEEVEILVCGSPE--------------------------LDMHALQRS

TQYDG----------------YAKTDLTIRYFWDVVL-GFPLEL----QKKLLHFTTGSD

RVPVGGMADLNFKI----SKNETS---------------------------------TNW

LP-VAHTCFN-----QLCLPP-YKSKKD--LKQKLIIGISNS-EGF

>Macaca_mulatta_XP_001088322.1 .

MKVRRTHLVSDSLDELTRK-----------------RADLK-KKLKVTFVG-----EAGL

---DMGGLTKEWFLLLIRQIFHP-------------------------------------

------------------------------------------------------------

------------------------------------------------------------

------------------------------------------------------------

------------------------------------------------------------

--------------------DYGMFTYHKDS-----------------------------

------------HCHWFSSFK------------CDNYSEFRLVG--ILMGLAVYNS----

-----ITLDIRFPP-CCYKKLLSPPIIPSDQ-----------------------------

--------------------------------NIPVGICSVTVDDLC---QIMPELA---

HGLSELLSHEGNV-----------------------------------------EEDFYS

TFQVFQEE-----------------------------FGIIKSYNLKPGG----DKISVT

NQNR--KEYVQLYTDFLLNKS-----IYKQFAAFYYG---------------FHSVCA--

--SNALM--LLRPEEVEILVCGSPD--------------------------LDMHALQRS

TQYDG----------------YAKTDLTIKYFWDVVL-GFPLDL----QKKLLHFTTGSD

RVPVGGMADLNFKI----SKNETS---------------------------------TNW

LP-VAHTCFN-----QLCLPP-YKSKKD--LKQKLIIGISNS-EGF

>Homo_sapiens_BX537856.1 .

MKVRRTHLVSDSLDELTRK-----------------RADLK-KKLKVTFV-----GEAGL

---DMGGLTKEWFLLLIRQIFHP-------------------------------------

------------------------------------------------------------

------------------------------------------------------------

------------------------------------------------------------

------------------------------------------------------------

--------------------DYGMFTYHKDS-----------------------------

------------HCHWFSSFK------------CDNYSEFRLVGIYALMGLAVYNS----

-----ITLDIRFPP-CCYKKLLSPPIIPSDQ-----------------------------

--------------------------------NIPVGICNVTVDDLC---QIMPELA---

HGLSELLSHEGNV-----------------------------------------EEDFYS

TFQVFQEE-----------------------------FGIIKSYNLKPGG----DKISVT

NQNR--KEYVQLYTDFLLNKS-----IYKQFAAFYYG---------------FHSVR---

--ASNAL-MLLRPEEVEILVCGSPD--------------------------LDMHALQRS

TQYDG----------------YAKTDLTIKYFWDVVL-GFPLDL----QKKLLHFTTGSD

RVPVGGMADLNFKI----SKNETS---------------------------------TNC

LP-VAHTCFN-----QLCLPP-YKSKKD--LKQKLIIGISNS-EGF

>Tursiops_truncatus_ABRN01156618.1 .

MKVRWTYLVSNSLDELTRK-----------------RADLQ-KKLKVTFV-----GEVGL

---GTGGLTKEWFLLLIRQIFHP-------------------------------------

------------------------------------------------------------

------------------------------------------------------------

------------------------------------------------------------

------------------------------------------------------------

--------------------DYGMFTYHKDS-----------------------------

------------YCHWFSSFK------------CDNYSGFRLVG--ILMGLAVYNS----

-----IALDIRFPR-CCYKKLLSPPIIPNDQ-----------------------------

--------------------------------NMPVGICSVTTDDLS---QIMPELA---

HGLSELLSYEGNV-----------------------------------------EEDFYS

IFQVFQEE-----------------------------FRIIKSYNLKPGG----DKIPVT

NQNR--KEYVQLYIDFLLNKS-----IYKQFAAFYYG---------------FHSVC---

--ASNFL-MLLHPEEVEILVCGSPE--------------------------LDMHALQRS

TQYDG----------------YAKTDLTIQYFWDIVL-GFPLDL----QKKLLHFTTGSD

RVPVEGMADLNFKI----SKNETS---------------------------------TNW

LP-VAHTCFN-----QLCLPP-YKNKKD--LKQKLIIGISNS-EGF

>Equus_caballus_XM_001501193.2 .

MKVRRTHLVSDSLDELTRK-----------------RADLK-KKLKVTFV-----GEAGL

---DMGGLTKEWFLLLIRQIFHP-------------------------------------

------------------------------------------------------------

------------------------------------------------------------

------------------------------------------------------------

------------------------------------------------------------

--------------------DYGMFTYHKDS-----------------------------

------------YCHWFSSFK------------CDNYSEFRLVG--ILMGLAVYNS----

-----ITLDIRFPP-CCYKKLLSPPIVPSDQ-----------------------------

--------------------------------NTPVGICSVTTDDLC---QIMPELA---

HGLSELLSYEGNV-----------------------------------------EEDFYS

TFQVFQEE-----------------------------FGIIKSYNLKPGG----DKIPVT

NQNR--KEYVQLYIDFLLNKS-----IYKQFAAFYYG---------------FHSVC---

--ASNAL-MLLRPEEVEILVCGSPE--------------------------LDMHALQRS

TQYDG----------------YAKTDLTIRYFWDVVL-GFPLDL----QKKLLHFTTGSD

RVPVGGMADLNFKI----SKNETS---------------------------------TNW

LP-IAHTCFN-----QLCLPP-YKSKKD--LKQKLIIGISNS-EGF

>Canis_familiaris_XM_543924.2 .

MKVRRTHLVSDSLDELTRK-----------------RADLK-KKLKVTFV-----GEAGL

---DMGGLTKEWFLLLIRQIFHP-------------------------------------

------------------------------------------------------------

------------------------------------------------------------

------------------------------------------------------------

------------------------------------------------------------

--------------------DYGMFTYHKDS-----------------------------

------------HCHWFSSFK------------CDNYSEFRLVG--ILMGLAVYNS----

-----ITLDIRFPP-CCYKKLLSPPIIPSDQ-----------------------------

--------------------------------NIPVGICSVTTDDLC---QIMPELA---

HGLSELLSYEGNV-----------------------------------------EEDFYS

TFQVFQEE-----------------------------FGIIKSYNLKPGG----DKIPVT

NQNR--KEYVQLYIDFLLNKS-----IYKQFAAFYYG---------------FHSVC---

--ASNAL-MLLRPEEVEILVCGSPE--------------------------LDMYALQRS

TQYDG----------------YAKTDLTIRYFWDVVL-GFPLDL----QKKLLHFTTGSD

RVPVGGMADLNFKI----SKNETS---------------------------------TNW

LP-VAHTCFN-----QLCLPP-YKSKKD--LKQKLIIGISNS-EGF

>Strong._purpuratus_XM_001194948.1 .

--------------------------------------------LRIRFM-----GEMGI

---DAGALSMDFFRLVFEELFDPG------------------------------------

------------------------------------------------------------

------------------------------------------------------------

------------------------------------------------------------

------------------------------------------------------------

--------------------KNGLFRYVDELDPTH-------------------------

------------SPVWFRKDC-------------EDSKLAEICG--LLCGFSLHNK----

-----AIVPLP-FPQLLYAKLLGKGPN---------------------------------

----------------------------------------SDMEEVA---ELDKEFA---

DQMSKLRTGTE-------------------------------------------DYVMGC

CYGE---------------------------------------KVELKNG----RSVDVT

KENV--HLYVDNMVRDYL--------VPSQFAAFQRG---------------FNRVF---

-NPAAFD--MFRPIELEALVMGEKH--------------------------FDWKALEQS

TQYKQ---------------PYTDSHPTIKMFWTVFH-ALDNDM----KRKFLVFVAGSD

RVPVGGLRNLGLHI----DHLQVPMDDSLDEQPCDTEDRL-----------------TRL

LP-VGHGCDNHGSRRTLRLPM-YTILEL--MEDRLTVALS------

>Strong._purpuratus_XM_001185007.1 .

IDNDKEKVVESAISEVKGKDET--------------SLLLP-LAVHFKDN-----ESWGV

---DAGGPMKEFFSRLFEELFNVE------------------------------------

------------------------------------------------------------

------------------------------------------------------------

------------------------------------------------------------

------------------------------------------------------------

--------------------KHSIFKKLKDSPSC--------------------------

------------TTLWFNKAD-------------KDLDKLRSVG--KLFALMFYNK----

-----VIVTMP-FPLLFYKKLLETSQSQPGQ-----------------------------

----------------------------------------SSFKDLE---LLDPGMA---

KSMETLLEASD-------------------------------------------DELSDY

RFTVDGPV--------------------------------NEPDIELKPG---GRDEVVT

TANV--REYIDLYAKHYT--------ASTQFDVFSES---------------FRSMF---

--GRFGLHQRFAPQELMSLFQG-KE--------------------------YDWKAFQKS

FGYDI-----VPTENASNEKGYTANDRVVKMFWSVFH-DLTEDD----KRDFLRILTGAD

HVPIGGFQEISPKM----WPMGGDGDCRGNPP-------------------------KDM

CP-EVNTCHD---YVVLHLPM-YSKREH--LKERLMKLIE------

>Homo_sapiens_AK295832.1 .

LRVRRSRLVKDALRQLSQAE----------------ATDFC-KVLVVEFI-----NEICP

---ESGGVSSEFFHCMFEEMTKP-------------------------------------

------------------------------------------------------------

------------------------------------------------------------

------------------------------------------------------------

------------------------------------------------------------

--------------------EYGMFMYPEMG-----------------------------

------------SCMWFPAKP------------KPEKKRYFLFG--MLCGLSLFNL----

-----NVANLPFPL-ALYKKLLDQK-----------------------------------

----------------------------------------PSLEDLK---ELSPRLG---

KSLQEVLDDAADDI----------------------------------------GDALCI

RFSIHWDQ---------------------------------NDVDLIPNG----ISIPVD

QTNK--RDYVSKYIDYIFNVS-----VKAVYEEFQRG---------------FYRVC---

--EKEIL-RHFYPEELMTAIIGNTD--------------------------YDWKQFEQN

SKYEQ---------------GYQKSHPTIQLFWKAFH-KLTLDE----KKKFLFFLTGRD

RLHARGIQKMEIVF----RCPETFS--------------------------------ERD

HP-TSITCHN-----ILSLPK-YSTMER--MEEALQVAINNN-RGF

>Macaca_mulatta_XR_011454.1 .

LRVRRSRLVKDALRQLSQAE----------------ATDFC-KVLVVEFI-----EEIRP

---ESGGVSSEFFHCMFEEMTKP-------------------------------------

------------------------------------------------------------

------------------------------------------------------------

------------------------------------------------------------

------------------------------------------------------------

--------------------EYGMFMYPEMG-----------------------------

------------SCMWFPAKP------------KLEKKRYFLFG--MLCGLSLFNL----

-----NVANLPFPL-ALYKKLLDQK-----------------------------------

----------------------------------------PSLEDLK---ELSPRLG---

KSLQEVLNDDADDI----------------------------------------GDVLCI

RFSIHWDQ---------------------------------NDVDLIPNG----ISIPVD

QTNK--KDYVSKYIDYIFNVS-----VKAVYEEFQRG---------------FYRVC---

--EKEIL-RHFYPEELMTAIIGNTD--------------------------YDWKQFEQN

SKYEQ---------------GYQKSHPTIRLFWKAFH-KLTLDE----KKKFLLFLTGRD

RLHARGIQKMEIVF----RCPETFS--------------------------------ERD

HP-TSITCHH-----ILYLPK-YSTMER--MEEALQVAINNN-RGF

>Pan_troglodytes_XM_001160807.1 .

LKVRRSRLVKDALRQLSQAE----------------ATDFC-KVLVVEFI-----NEICP

---ESGGVSSEFFHCMFEEMTKP-------------------------------------

------------------------------------------------------------

------------------------------------------------------------

------------------------------------------------------------

------------------------------------------------------------

--------------------EYGMFMYPEMG-----------------------------

------------SCMWFPAKP------------KPEKKRYFLFG--ILCGLSLFNL----

-----NVANLPFPL-ALYKKLLDQK-----------------------------------

----------------------------------------PSLEDLK---ELSPRLG---

KSLQEVLDDDADDI----------------------------------------GDALCI

RFSIHWDQ---------------------------------NDVDLIPNG----ISIPVD

QTNK--RDYVSKYIDYIFNVS-----VKAVYEEFQRG---------------FYTVC---

--EKEIL-RHFYPEELMTAIIGNTD--------------------------YDWKQFEQN

SKYEQ---------------GYQKSHPTIQLFWKAFH-KLTLDE----KKKFLFFLTGRD

RLHARGIQKMEIVF----CCPETFS--------------------------------ERD

HP-TSLTCHN-----ILYLPK-YSTMER--MEEALQVAINNN-RGF

>Equus_caballus_XM_001494837.1 .

LKVRRSHLVEDALRQLSQAE----------------VTDLR-KQLVIEFI-----KEIRP

---ESGGVKSEFFHCIFEEMTKT-------------------------------------

------------------------------------------------------------

------------------------------------------------------------

------------------------------------------------------------

------------------------------------------------------------

--------------------EYGMFIYPEKG-----------------------------

------------SCMWFPANP------------KFEEKSYFLFG--MLCGLSLYNF----

-----NVVNLSFPL-ALFKKLLDQK-----------------------------------

----------------------------------------PSLEDFK---ELSPLWG---

KNLQEILNDQS-D-----------------------------------------IGEDYI

YFSIHWDH---------------------------------NDVDLIPDG----ISVLVN

QNNK--KDYVSKCVDYVFNTS-----VKAVYEEFQRG---------------FYKVC---

--DKEIL-RHFQPEELMTAIVGNTD--------------------------YDWEQFEKN

SKYTL---------------PYHKSHRTIVMFWKAFH-ALTLEE----KKKFLFFLTGND

RLPIRGLQEVGILF----RCPETFS--------------------------------ERD

HP-RSLTCHN-----ILDLPE-YSTMRR--MKEALQVAINSN-RGF

>Bos_taurus_XM_605913.4 .

LSVRRSHLVEDALCQLSQAE----------------DTDLQ-KILVVEFI-----KEIRS

---GGEGVKSEFFHCIFESMTKE-------------------------------------

------------------------------------------------------------

------------------------------------------------------------

------------------------------------------------------------

------------------------------------------------------------

--------------------EYGMFMYPEED-----------------------------

------------SYMWFPVKP------------KFEKKMYFLFG--MLCGLSLYNF----

-----NVVYLPFPL-ALFKKLLDQE-----------------------------------

----------------------------------------PSLEDLK---ELSPSFG---

KCLQEVLNDDANDI----------------------------------------KEEFGL

RFSIHWDQ---------------------------------NDVDLIPNG----ISVFVD

QSNK--KDYVSKCVDYVLNTS-----VKAVYEEFQRG---------------FYKLF---

--DKEIL-KHFKPEELMRAIIGNTD--------------------------YDWEQFEKN

SIYEQ---------------GYHESHPTILMFWKAFH-SLTLDE----KRKFLFFLTGND

RLHVKGIQKPGIRF----RCPETCS--------------------------------ESD

FP-KSLTCHN-----ILELPE-YSTMKK--VKEALQRAINSN-KGF

>Canis_familiaris_XM_846456.1 .

LRVRRRHLVEDALRQLSQAE----------------DTDLH-KAFMVEFI-----KEIRS

---IGYGVKSEFFYSIFEEMTNI-------------------------------------

------------------------------------------------------------

------------------------------------------------------------

------------------------------------------------------------

------------------------------------------------------------

--------------------EYGMFMYPEEG-----------------------------

------------SYMWFPAKS------------KFKKKRYFLFG--ILCGLSLYNL----

-----NPANLPFPL-ALFKKLLDQK-----------------------------------

----------------------------------------PSFQDLK---ELSPLLG---

KNLQEILDNEADD-----------------------------------------TEELYI

YFSIYWDK---------------------------------NNVNLIPNG----ISVPVD

QTNK--KEYVSACIDYIFNTS-----VKAVYEEFQRG---------------FYKVC---

--DKEILVKLFQPEELMTALVGNND--------------------------YDWKYFEEN

SQYGQ---------------GYHKSHPTILMFWKAFH-KLTLEE----KKKFLFFLRGND

RLPLSGKQEIGIKF----RCPETFS--------------------------------EAD

YP-RALTCHN-----ILDLPQ-YSTMEK--VEEALQVAINSN-KGF

>Mus_musculus_AK147960.1 .

LKVRRSHLVEDTLRQLRQVE----------------DFDLR-KQLSVGFI-----NEIRP

---EAGGVSSEFFHCIFEEMTDP-------------------------------------

------------------------------------------------------------

------------------------------------------------------------

------------------------------------------------------------

------------------------------------------------------------

--------------------KYEMFIYPEKG-----------------------------

------------SSMWFPVNP------------KFEKSSYFLFG--ILCGLSLHNL----

-----KVINLPFPL-ALYKKLLNQK-----------------------------------

----------------------------------------PSLEDLK---ELSLPLG---

RNLQEVLNCEAGD-----------------------------------------IEELHM

YFSIYWDQ---------------------------------KDVDLIPDG----ISVPVN

ETNK--RDYVSKYVDYIFNIS-----IKTIYEEFHRG---------------FYKVC---

--NWDIIR-QFQPEELMTAIIGNAT--------------------------CDWKQFENN

SKYKD---------------GYDKSHPTILLFWKAFH-DLTLDE----KKKFLLFLTGCD

RLHVKGLQNEGIIF----RCSETFS--------------------------------EED

NP-RSLTCHR-----MLDLPK-YSSMRR--MKEALQVAINNS-TGF

>Rattus_norvegicus_XM_342700.3 .

LKVRRSHLVEDTLRQLRQAE----------------DFDLR-KTLSVGFI-----NEIRP

---EGGGVSSEFFHCIFEEMTDP-------------------------------------

------------------------------------------------------------

------------------------------------------------------------

------------------------------------------------------------

------------------------------------------------------------

--------------------KYEMFMYPENG-----------------------------

------------SNMWFPVNP------------KFEKSRYFLFG--ILCGLSLNNL----

-----NVINLSFPL-ALYKKLLEQK-----------------------------------

----------------------------------------PSLEDLK---DLSLLLG---

RNLQEVLNCEAGV-----------------------------------------IEELHM

YFSIYWDQ---------------------------------RDVDLIPDG----ISVPVN

ETNK--RDYVSKCVDYIFNIS-----IKTIYDEFHRG---------------FYKVC---

--NRDSI-RHFQPEELMAAIIGNPT--------------------------CDWKQFENN

SKYEN---------------GYSKSHPTILLFWKAFH-ELTLDE----KKKFLLFLTGCD

RLHVKGLQNEGIRF----RCPEVFS--------------------------------ERD

NP-RSLTCHS-----ILDLPK-YSTMRR--MKEALQVAINNN-KGF

>Rattus_norvegicus_XM_001064216.1 .

LKVRRSHLVEDTLRQLRQAE----------------DFDLR-KTLSVGFI-----NEIRP

---EGGGVSSEFFHCIFEEMTDP-------------------------------------

------------------------------------------------------------

------------------------------------------------------------

------------------------------------------------------------

------------------------------------------------------------

--------------------KYEMFMYPENG-----------------------------

------------SNMWFPVNV---------SYVFPNKMHYMQTRV-GLCGLRARRI----

-----HII-VTKPL-LTARKALEQQ-----------------------------------

----------------------------------------SWNVDIK-YIILNQFFN---

RNLQEVLNCEAGV-----------------------------------------IEELHM

YFSIYWDQ---------------------------------RDVDLIPDG----ISVPVN

ETNK--RDYVSKCVDYIFNIS-----IKTIYDEFHRG---------------FYKVC---

--NRDSIR-HFQPEELMAAIIGNPT--------------------------CDWKQFENN

SKYEN---------------GYSKSHPTILLFWKAFH-ELTLDE----KKKFLLFLTGCD

RLHVKGLQNEGIRF----RCPEVFS--------------------------------ERD

NP-RSLTCHS-----ILDLPK-YSTMRR--MKEALQVAINNN-KGF

>Homo_sapiens_AY337518.1 .

LTVRRNHLIEDVLNQLSQFE----------------NEDLR-KELWVSFS-----GEIGY

---DLGGVKKEFFYCLFAEMIQP-------------------------------------

------------------------------------------------------------

------------------------------------------------------------

------------------------------------------------------------

------------------------------------------------------------

--------------------EYGMFMYPEGA-----------------------------

------------SCMWFPVKP------------KFEKKRYFFFG--VLCGLSLFNC----

-----NVANLPFPL-ALFKKLLDQM-----------------------------------

----------------------------------------PSLEDLK---ELSPDLG---

KNLQTLLDDEGDNF----------------------------------------EEVFYI

HFNVHWDR---------------------------------NDTNLIPNG----SSITVN

QTNK--RDYVSKYINYIFNDS-----VKAVYEEFRRG---------------FYKMC---

--DEDII-KLFHPEELKDVIVGNTD--------------------------YDWKTFEKN

ARYEP---------------GYNSSHPTIVMFWKAFH-KLTLEE----KKKFLVFLTGTD

RLQMKDLNNMKITF----CCPESW---------------------------------NER

DPIRALTCFS-----VLFLPK-YSTMET--VEEALQEAINNN-RGF

>Macaca_fascicularis_AB168451.1 .

LTVRRNHLIEDVLNQLSQFE----------------NEDLR-KELWVSFS-----GEIGY

---DLVGVKREFFYCLFEEMIQP-------------------------------------

------------------------------------------------------------

------------------------------------------------------------

------------------------------------------------------------

------------------------------------------------------------

--------------------EYGMFMYPEGA-----------------------------

------------SCMWFPVRP------------KFEKKRYFFFG--LLCGLSLFNC----

-----NVANLPFPL-ALFKKLLDQM-----------------------------------

----------------------------------------PSLEDLK---ELSPDLG---

KNLQTLLDDEGDNF----------------------------------------EEVFYI

HFNVHWDR---------------------------------NDTNLIPNG----SSIIVN

QTNK--RDYVSKYIDYIFNDS-----VKAVYEEFRRG---------------FYKMC---

--DEDII-KLFHPEELKDVIVGHTD--------------------------YDWKTFEKN

ARYEP---------------GYNSSHPTIVMFWKAFH-KLTLEE----KKKFLVFLTGTD

RLQTKDLKNMKITF----CCPESW---------------------------------NER

DPMRALTCFS-----VLFLPK-YSTMET--VEEALQVAINNN-RGF

>Pteropus_vampyrus_ABRP01284462.1 .

----------------------------------------------LSFS-----GEIRH

---DFGGVKAEFFHCLFKKMNRS-------------------------------------

------------------------------------------------------------

------------------------------------------------------------

------------------------------------------------------------

------------------------------------------------------------

--------------------EYGMCMDPEEA-----------------------------

------------SNM----CP------------KFE-KRYF-FG--VLCGFSLFSC----

-----NVANILFPM-ALFKKLSDQT-----------------------------------

----------------------------------------LSLEDLK---KLTPVLG---

KSLQTLLDDESDDF----------------------------------------REVFYI

DFNMYWDK---------------------------------NDVNLISNR----SCIIVD

QTNKT-DCFIQL----ITSTS-----LRQLMKTYKED---------------FTSRLL--

--NFSILK----TRMLLEIIMTGKH--------------------------LKRMLITDK

----------------------DMTISTTVMFWKALH-KLTLVE----KKKFLAFFIGTD

RIQVKVSENMKITF----CYEFFFFTVEKFYKGKYTSRRGSSPEQGKHSSKGLSEILNEK

DPIRAQTCFC-----VLYLPK-YSTMKR--MEEVLQVTINNN-RGF

>Equus_caballus_XM_001915080.1 .

LTVRRSHLIEDVLNHLNRFE----------------NEDLR-RELLVSFS-----GEIPL

---DYGGVRAEFFHCLFEELTQP-------------------------------------

------------------------------------------------------------

------------------------------------------------------------

------------------------------------------------------------

------------------------------------------------------------

--------------------EYGLFTYPEEA-----------------------------

------------SYMWFPVRP------------KFEKKSYFFFG--VLCGLCLFNC----

-----NVANIPFPL-ALFKKLLDQT-----------------------------------

----------------------------------------PSLEDLK---ELSPVFG---

RSLQTLLDDEGEDF----------------------------------------GEVFYI

HFTVHWDR---------------------------------NAEELIPNG----SDIIVD

QTNK--RDYVSKCVNYIFNIS-----IKAVYEEFQRG---------------FYKVC---

--DKEII-GFFHPAELKDVIIGNTD--------------------------YDWETFEKN

ARYAG---------------GYDSSHPTIVMFWEALH-KLTLEE----KKKFLVFLTGTD

RLQVKGVKNMKITF----CCPEHL---------------------------------DEK

DPIRAQTCFS-----VLYLPK-YSTMER--VEEALQVAINNN-RGF

>Bos_taurus_NM_001101995.1 .

LIVRRSHLIEDVFSQLNQFE----------------NEDLR-RELMVSFS-----GEIGY

---NFGGVRAEFFYCLFQEMTRP-------------------------------------

------------------------------------------------------------

------------------------------------------------------------

------------------------------------------------------------

------------------------------------------------------------

--------------------EYGMFTYPEEA-----------------------------

------------SYMWFPVRP------------KFEKKSYFFFG--LLCGLSLFNC----

-----NVADIPFPL-ALFKKLLDQT-----------------------------------

----------------------------------------PSLEDLK---ELSPVLG---
[truncated: 239,393 more chars]
